# Global, Regional, and National Burden of Early‐Onset Alzheimer's Disease and Other Dementias in Young Adults Aged 40–64 Years, 1990–2021: A Population‐Based Study

**DOI:** 10.1111/ene.70116

**Published:** 2025-03-19

**Authors:** Zenghui Zhang, Shaojie Han, Huimin Zhu, Qianyun Wang, Siyuan Cheng, Yuchen Han, Fengjuan Li, Jun Guo

**Affiliations:** ^1^ Department of Cardiology Jinan University First Affiliated Hospital Guangzhou China; ^2^ The First Clinical Medical College Jinan University Guangzhou China

**Keywords:** early onset Alzheimer's dementia, early onset dementia, global burden, prevalence, young adults

## Abstract

**Background and Purpose:**

Early‐onset Alzheimer's disease and other dementias (EOAD) impose significant burdens on affected individuals and their families. However, the global burden of EOAD has not been fully investigated. We aimed to assess the global, regional, and national burden of EOAD using data from the Global Burden of Diseases, Injuries, and Risk Factors (GBD) study from 1990 to 2021.

**Methods:**

Data for adults aged 40–64 were extracted within the GBD 2021 framework. Primary outcomes included age‐standardized prevalence, incidence, mortality, and disability‐adjusted life years (DALYs) for EOAD, as well as average annual percentage change (AAPC) across 21 regions and 204 countries.

**Results:**

In 2021, EOAD cases reached 7.75 million (95% uncertainty interval [UI] 5.82–10.08), up from 3.67 million cases (95% UI 2.75–4.76) in 1990. The age‐standardized prevalence rate increased from 341.2 per 100,000 (95% UI 255.89–442.79) in 1990 to 363.5 per 100,000 in 2021, with an AAPC of 0.26% (*p* < 0.001). EOAD prevalence was higher in women than in men in 2021 (4.28 million, 95% UI 3.24–5.56, vs. 3.46 million, 95% UI 2.57–4.52). EOAD was associated with 0.07 million (95% UI 0.01–0.23) deaths and 3.77 million (95% UI 1.69–8.88) DALYs in 2021. Additionally, 1.06 million (95% UI 0.07–3.03) DALYs were attributable to smoking, elevated fasting plasma glucose, and high body mass index.

**Conclusions:**

The global number of EOAD cases among adults aged 40–64 years more than doubled from 1990 to 2021. Targeted strategies and interventions are urgently needed to address this growing public health issue.

## 1 Introduction

Early‐onset Alzheimer's disease and other dementias (EOAD) represent a significant global health challenge, particularly among adults aged 40–64 years [1, 2]. Unlike late‐onset dementias, which occur after age 65, EOAD affects individuals in their productive years, significantly impacting patients, families, and communities [3, 4, 5, 6, 7]. Earlier onset of EOAD is associated with more aggressive progression and atypical symptoms, complicating diagnosis and management.

Despite the growing recognition of EOAD as a critical public health issue, the global burden of this condition has not been comprehensively explored. Previous studies have often focused on regional or single‐country data [8], leading to a fragmented understanding of the global landscape of EOAD. Research from Girona shows that EOAD makes up about 6.9% of the total dementia population, with prevalence tripling and doubling every 5 years of age [9]. A modeling study has projected that the number of dementias was approximately 57.4 million cases globally in 2019, which will increase to 152.8 million cases by 2050 [10]. This growing prevalence reflects an increasing number of individuals living with EOAD during their early adult years.

EOAD patients face unique challenges due to earlier onset, often balancing additional familial, occupational, and community responsibilities. Most dementia care networks are designed for older patients, whose social and familial situations differ [11, 12, 13]. This discrepancy suggests that EOAD management may be less effective than that for late‐onset dementia. Additionally, delays in accurate diagnosis are common in EOAD, often due to late referrals and its atypical manifestations [4, 14].

Given the rising prevalence of EOAD and its poor outcomes, it is crucial to assess its global, regional, and national burden. This study aims to examine the prevalence, incidence, DALYs, and mortality of EOAD in young adults (40–64 years) from 1990 to 2021, considering socio‐demographic factors, age, and sex. We also evaluate the risk factors contributing to DALYs in this population.

## 2 Methods

### 2.1 Study Population and Data Collection

This study used data from the GBD 2021, which assesses the health impact of 371 diseases and injuries across 204 countries and territories [15]. The GBD 2021 offers a comprehensive assessment of disease burden, including prevalence, severity, and mortality, with our analysis focusing on individuals diagnosed with early‐onset Alzheimer's disease (EOAD) before the age 65.

The cross‐sectional data from the Global Health Data Exchange were obtained, covering the burden of 371 diseases and injuries, including EOAD, across 21 global regions from 1990 to 2021 [16, 17]. The study population included young adults aged 40–64 years diagnosed with EOAD [1, 2]. We extracted age‐, sex‐, and location‐specific data on EOAD prevalence, incidence, mortality, and DALYs, along with their 95% uncertainty intervals (UIs). Methodologies used in the GBD 2021 study are detailed in previous publications [16, 17, 18].

### 2.2 Definitions and Modeling

For the GBD analyses, EOAD definitions were based on criteria from the International Classification of Diseases (ICD; ICD‐8, ICD‐9, and ICD‐10) and the Diagnostic and Statistical Manual of Mental Disorders (DSM III, IV, or V), as previously reported [19, 20]. The relevant ICD‐9 codes for dementia included 290, 291.2, 291.8, 294, and 331, while the relevant ICD‐10 codes were F00, F01, F02, F03, G30, and G31 [18, 21]. The EOAD definition also includes self‐reports, healthcare provider diagnoses, and hospital records, with much data from epidemiological studies due to the lack of standardized dementia registries in addition to ICD code.

In this study, we used the Average Annual Percent Change (AAPC) to measure the annual rate of change in the target variable (e.g., disease incidence, mortality). AAPC is calculated by performing regression analysis and applying the formula AAPC = (exp(β1) − 1) × 100 [22], where β1 is the regression slope, converting the logarithmic values to the original scale.

The Socio‐Demographic Index (SDI) is a composite measure of a country's per capita income, average years of schooling, and fertility rate in females under 25, rescaled based on health indicators to produce a value between 0.005 and 1. The geometric mean of these rescaled values, multiplied by 100, reflects socio‐economic conditions influencing health outcomes, with higher values indicating more advanced development [23].

To estimate the nonfatal burden of EOAD, we used the Bayesian meta‐regression tool DisModMR 2.1, which models disease trends over time, geography, age, and sex using available data [15, 18]. The GBD Study models dementia data to create a comprehensive database for most countries, using spatiotemporal Gaussian process regression to smooth data across age, location, and time in regions with incomplete datasets.

### 2.3 Age Groups, Regional, and Risk Factor Analysis

Our analysis examined five age groups (40–44, 45–49, 50–54, 55–59, and 60–64) for both sexes, using data from 21 regions with similar epidemiological profiles. The SDI was categorized into five levels: low, low‐middle, middle, high‐middle, and high. Given that the GBD includes only high BMI, high fasting plasma glucose, and smoking as dementia risk factors, we primarily focused on these three factors in our analysis.

### 2.4 Statistical Analysis

A descriptive analysis was conducted to outline the global burden of EOAD among adults aged 40–64 years. We compared age‐standardized rates (per 100,000 population) for prevalence, incidence, mortality, and DALYs of EOAD across different groups stratified by age, sex, region, and country. Temporal trends were assessed by calculating AAPC, with corresponding 95% confidence intervals (CIs). Trends were classified as increasing, decreasing, or stable based on the statistical significance of the AAPC. The epidemiological measures used in this study—prevalence, incidence, mortality, and DALYs—are interrelated. All statistical analyses were performed using R (version 4.4.1), and the R packages we used include tidyverse, ggsci, dplyr, sf, patchwork, ggplot2, and reshape2.

## 3 Results

### 3.1 Global Trends

**FIGURE 1:**
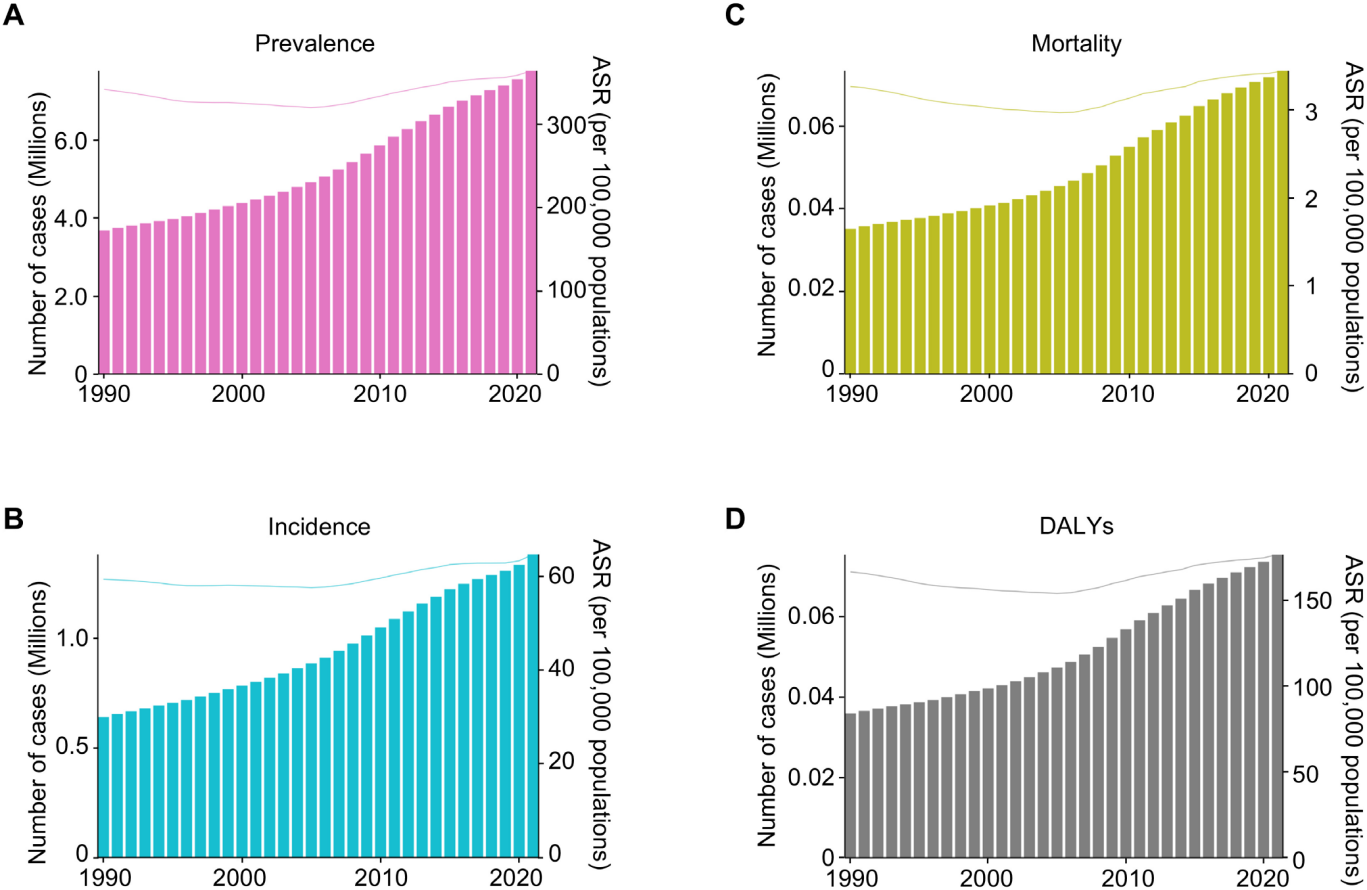
Global trends in the prevalence, incidence, mortality, and DALYs of early‐onset Alzheimer's disease and other dementias, 1990–2021. The age‐standardized numbers of (A) prevalence, (B) incidence, (C) mortality, and (D) disability‐adjusted life years (DALYs) associated with early‐onset Alzheimer's disease and other dementias (EOAD) in populations aged 40–64 years, spanning the period from 1990 to 2019. The bars represent the number of cases (left y‐axis), while the lines indicate the age‐standardized rate (ASR) per 100,000 population (right y‐axis).

Between 1990 and 2021, the global prevalence of EOAD in adults aged 40–64 years increased from 3.67 to 7.75 million cases, marking a 111% increase over the period (Figure 1, Table 1). The age‐standardized prevalence of EOAD increased from 341.2 per 100,000 in 1990 to 363.5 per 100,000 in 2021, with an AAPC of 0.26% (*p* < 0.001) (Table 1). The incidence followed a similar trend, rising from 0.63 million new cases in 1990 to 1.38 million in 2021, with the age‐standardized incidence rate increasing from 59.4 to 64.7 per 100,000, showing an AAPC of 0.29% (*p* < 0.001) (Table S1). The age‐standardized mortality rate for EOAD increased from 3.21 per 100,000 in 1990 to 3.44 per 100,000 in 2021, with an AAPC of 0.26% (*p* < 0.001). Similarly, the age‐standardized DALY rate rose from 166.7 per 100,000 in 1990 to 176.8 per 100,000 in 2021, showing an AAPC of 0.27% (*p* < 0.001) (Table S2).

**TABLE 1:** Age‐standardized prevalence and AAPC of EOAD in young adults aged 40–64 years at global and regional levels, 1990–2021.

	Prevalence					
	No of people with EOAD in 1990 (Thousands)	Age standardized rate in 1990 (per 100,000)	No of people with EOAD in 2021 (Thousands)	Age standardized rate in 2021 (per 100,000)	AAPC (95% CI)	*p*
Global	3675.35 (2756.03 to 4769.07)	341.24 (255.89 to 442.79)	7757.68 (5826.99 to 10080.81)	363.45 (273 to 472.29)	0.26 (0.14 to 0.38)	< 0.001
Sex						
Female	2026.76 (1526.63 to 2624.59)	380.03 (286.25 to 492.13)	4288.57 (3244.30 to 5561.42)	399.98 (302.59 to 518.7)	0.23 (0.11 to 0.35)	< 0.001
Male	1648.59 (1227.10 to 2152.82)	303.19 (225.68 to 395.93)	3469.11 (2576.63 to 4520.56)	326.58 (242.56 to 425.56)	0.28 (0.16 to 0.4)	< 0.001
Age group (years)						
40–44	52.56 (23.18 to 88.25)	18.35 (8.09 to 30.81)	86.22 (37.44 to 146.91)	17.23 (7.48 to 29.37)	‐0.22 (−0.24 to −0.2)	< 0.001
45–49	280.05 (178.92 to 415.8)	120.61 (77.05 to 179.07)	549.14 (346.66 to 817.09)	115.97 (73.21 to 172.56)	‐0.15 (−0.17 to −0.13)	< 0.001
50–54	630.63 (445.28 to 848.76)	296.67 (209.48 to 399.28)	1316.05 (930.38 to 1775.69)	295.79 (209.11 to 399.1)	−0.03 (−0.05 to −0.02)	0.001
55–59	1058.76 (826.97 to 1336.79)	571.69 (446.53 to 721.81)	2345.03 (1828.79 to 2960.59)	592.59 (462.13 to 748.14)	0.08 (0.06 to 0.09)	< 0.001
60–64	1653.35 (1281.68 to 2079.46)	1029.42 (798.01 to 1294.73)	3461.25 (2683.72 to 4380.53)	1081.48 (838.54 to 1368.71)	0.15 (0.13 to 0.17)	< 0.001
SDI level						
High	889.18 (669.24 to 1149.79)	366.28 (275.68 to 473.63)	1542.23 (1169.9 to 1988.7)	423.5 (321.26 to 546.1)	0.65 (0.56 to 0.74)	< 0.001
High‐middle	972.04 (728.55 to 1262.98)	382.12 (286.4 to 496.48)	1891.63 (1424.83 to 2467.55)	421.23 (317.29 to 549.48)	0.39 (0.19 to 0.6)	0.001
Middle	1089.96 (813.82 to 1424.19)	345.47 (257.95 to 451.41)	2733.46 (2045.33 to 3586.23)	377.52 (282.48 to 495.29)	0.27 (0.14 to 0.41)	< 0.001
Low‐middle	522.08 (385.71 to 684.21)	272.62 (201.41 to 357.29)	1160.26 (858.83 to 1518.81)	273.44 (202.4 to 357.94)	‐0.03 (−0.11 to 0.05)	0.39
Low	198.23 (146.93 to 259.15)	276.11 (204.66 to 360.97)	424.18 (312.3 to 558.53)	247.94 (182.54 to 326.46)	−0.38 (−0.42 to −0.33)	< 0.001

Abbreviations: AAPC, average annual percentage change; CI, confidence interval; EOAD, early‐onset Alzheimer's disease and other dementias.

### 3.2 Global Trends by Sex

From 1990 to 2021, both men and women saw increases in age‐standardized EOAD prevalence. The number of cases among men rose from 1.64 to 3.46 million, while women's cases grew from 2.02 to 4.28 million. Although women had higher prevalence, the relative increase was greater among men (AAPC 0.28% vs. 0.23%, *p* < 0.001) (Table 1). Similarly, the incidence of EOAD increased significantly in both sexes, with an AAPC of 0.28% for men and 0.26% for women (*p* < 0.001 for both) (Table S1). From 1990 to 2021, both mortality and DALY rates due to EOAD increased for both sexes. Men's mortality rose by 0.28% annually, while women's increased by 0.23%. The DALY rate for men rose from 145.2 to 155.2 per 100,000, and for women, it increased from 188.7 to 198.1 per 100,000, highlighting the ongoing burden of EOAD across both sexes (Table S2).

### 3.3 Global Trends by Age Group

From 1990 to 2021, EOAD prevalence at least doubled in individuals aged 50–64, with a more than two‐fold increase in the 50–54 years (0.63–1.31 million), 55–59 years (1.05–2.3 million), and 60–64 years (1.65–3.46 million) age groups. In individuals aged 40–49 years, prevalence nearly doubled, increasing from 0.05 to 0.08 million in those aged 40–44 years and from 0.28 to 0.54 million in those aged 45–49 years.

**FIGURE 2:**
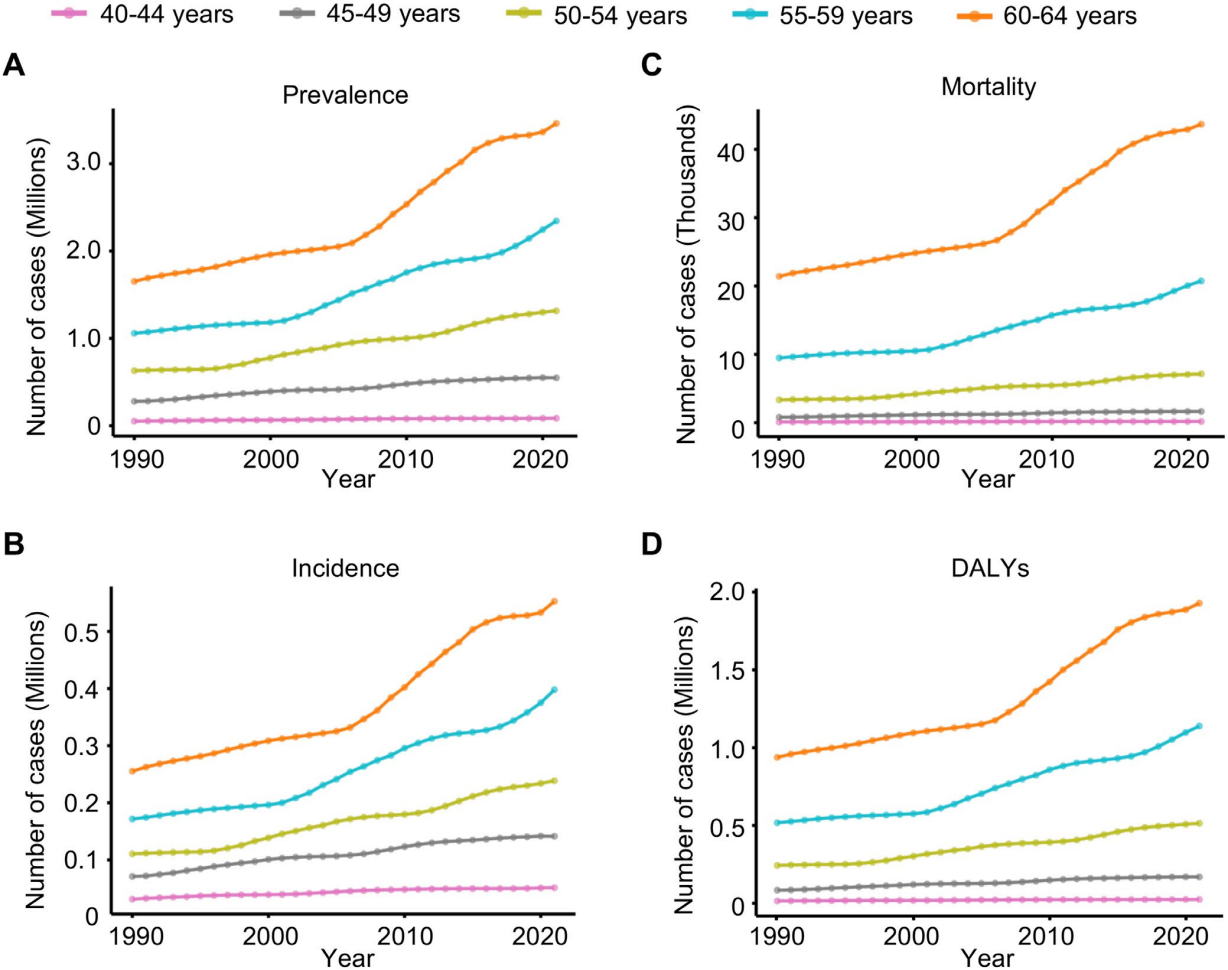
Global trends in the prevalence, incidence, mortality, and DALYs of early‐onset Alzheimer's disease and other dementias by age group, 1990–2021. The age‐standardized numbers of (A) prevalence, (B) incidence, (C) mortality, and (D) disability‐adjusted life years (DALYs) associated with early‐onset Alzheimer's disease and other dementias (EOAD) across five age groups: 40–44 years (pink line), 45–49 years (gray line), 50–54 years (green line), 55–59 years (blue line), and 60–64 years (orange line).

Among all age groups, individuals aged 60–64 years consistently exhibited the highest burden of EOAD. The prevalence in this group increased from 1029.4 per 100,000 in 1990 to 1081.5 per 100,000 in 2021, though with a modest AAPC of 0.15% (*p* < 0.001) (Table 1 and Figure 2). Conversely, a decreasing trend was observed in individuals aged 40–44 years (AAPC −0.22%), 45–49 years (AAPC −0.15%), and 50–54 years (AAPC −0.03%) (Table 1, Figure S1). Incidence rates in the 60–64 years age group also increased, albeit less dramatically, with an AAPC of 0.22% (*p* < 0.001) (Table S1, Figure S1). From 1990 to 2021, mortality rates and DALYs due to EOAD increased across all age subgroups in the 40–64 year range, with the highest DALYs in the 60–64 age group at 602 per 100,000 in 2021 (Table S2, Figure S1).

### 3.4 Regional Trends

**FIGURE 3:**
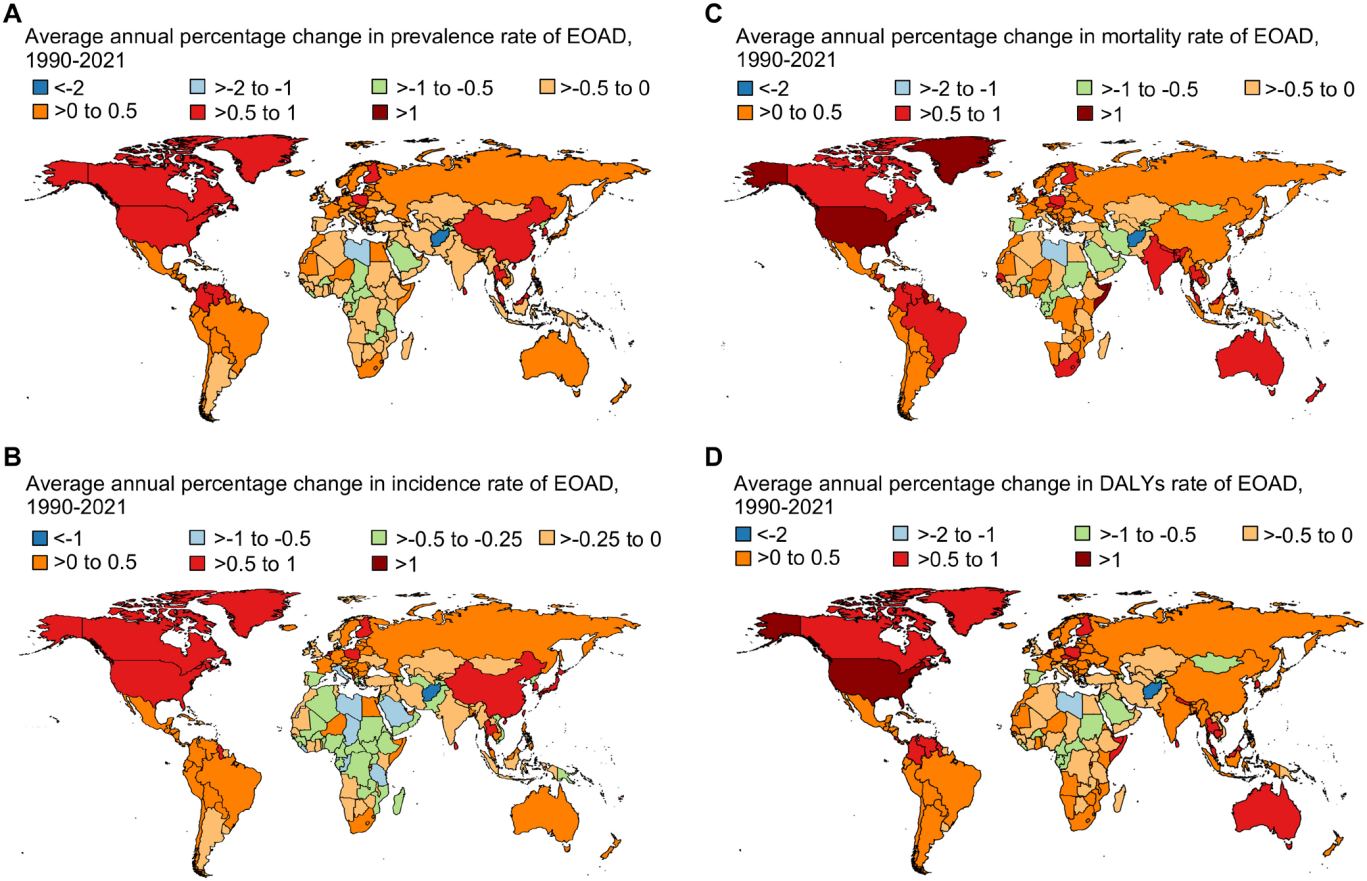
Average annual percentage change in the prevalence, incidence, mortality, and DALYs of early‐onset Alzheimer's disease and other dementias, 1990–2021. Average annual percentage change in rate of (A) prevalence, (B) incidence, (C) mortality, and (D) disability‐adjusted life years (DALYs) associated with early‐onset Alzheimer's disease and other dementias (EOAD) among populations aged 40–64 years, covering the period from 1990 to 2021.

Regional trends in EOAD from 1990 to 2021 showed significant variation in prevalence, incidence, mortality, and DALYs among those aged 40–64 (Figure 3, Figures S2 and S3). The largest increases in age‐standardized prevalence were seen in High‐income North America (AAPC 0.95%) and East Asia (AAPC 0.67%). In contrast, Central and Western Sub‐Saharan Africa showed declines in prevalence, with negative AAPCs of −0.38% and −0.35%, respectively. In 2021, High‐income North America had the highest prevalence (551.03 per 100,000), while Western Sub‐Saharan Africa had the lowest (212.55 per 100,000). In terms of incidence, High‐income North America experienced the most significant increases (AAPC 0.82%). Conversely, Central Sub‐Saharan Africa saw a marked decrease in incidence rates, with an AAPC of −0.37% (Table S3, Figure S4). From 1990 to 2021, mortality and DALYs generally increased across regions. Western Sub‐Saharan Africa saw the largest rise in mortality (AAPC 0.71%), followed by Western Europe (AAPC 0.52%). DALYs grew most notably in High‐income North America (AAPC 1.02%), Australasia (AAPC 0.61%), and Tropical Latin America (AAPC 0.47%), while North Africa and the Middle East experienced a decrease (AAPC −0.47%) (Table S4, Figure S4). After stratifying by sex, similar trends were generally observed (Tables S5–S8, Figures S5 and S6).

### 3.5 Country‐Level Trends

Canada had the highest age‐standardized prevalence rate, rising from 482.34 per 100,000 in 1990 to 606.41 per 100,000 in 2021, with an AAPC of 0.76% (*p* < 0.001). It also led in incidence rate in 2021, reaching 95.57 per 100,000, up from 80.10 per 100,000 in 1990, with an AAPC of 0.51% (*p* < 0.001). The Northern Mariana Islands had the highest AAPC for age‐standardized prevalence (1.93%, *p* < 0.001), followed by Taiwan (1.25%) and the US Virgin Islands (1.18%). Conversely, Afghanistan saw the largest decline in age‐standardized prevalence, with an AAPC of −2.77% (*p* < 0.001) (Table S9). Regarding mortality, the Northern Mariana Islands exhibited the highest increase in age‐standardized mortality with an AAPC of 2.23%, while the Islamic Republic of Afghanistan showed the most substantial decrease with an AAPC of −4.67%. For DALYs, the Northern Mariana Islands had the highest increase (AAPC 2.11%), whereas the Islamic Republic of Afghanistan saw the most significant decrease (AAPC −3.22%) (Table S10). Similar trends were observed after stratifying by sex (Tables S11–S14).

### 3.6 Global Trends by Socio‐Demographic Index

**FIGURE 4:**
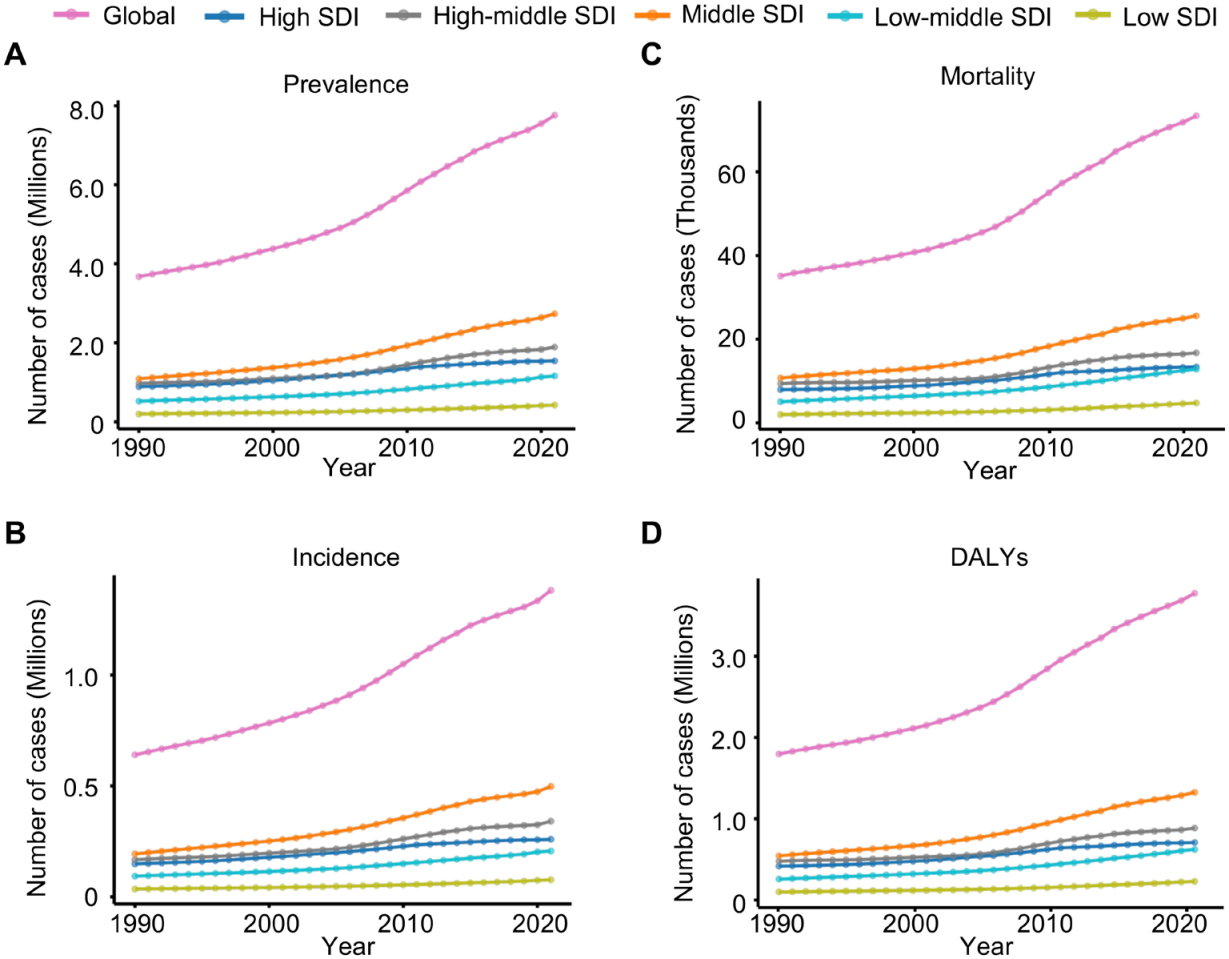
Global trends in the prevalence, incidence, mortality, and DALYs of early‐onset Alzheimer's disease and other dementias by SDI group, 1990–2021. The global trends in the age‐standardized numbers of (A) prevalence, (B) incidence, (C) mortality, and (D) disability‐adjusted life years (DALYs) associated with early‐onset Alzheimer's disease and other dementias (EOAD) in young adults aged 40–64 years across six Socio‐Demographic Index (SDI) groups: global (pink line), high SDI (dark blue line), high‐middle SDI (gray line), middle SDI (orange line), low‐middle SDI (blue line), and low SDI (green line).

From 1990 to 2021, the age‐standardized prevalence of EOAD in adults aged 40–64 years increased across all SDI subgroups (Figure 4), reaching 1.54 million cases in 2021, up from 0.88 million in 1990. However, the prevalence rate declined in low and low‐middle SDI countries, with AAPC of −0.38% (*p* < 0.001) and −0.03% (*p* = 0.390), respectively.

**FIGURE 5:**
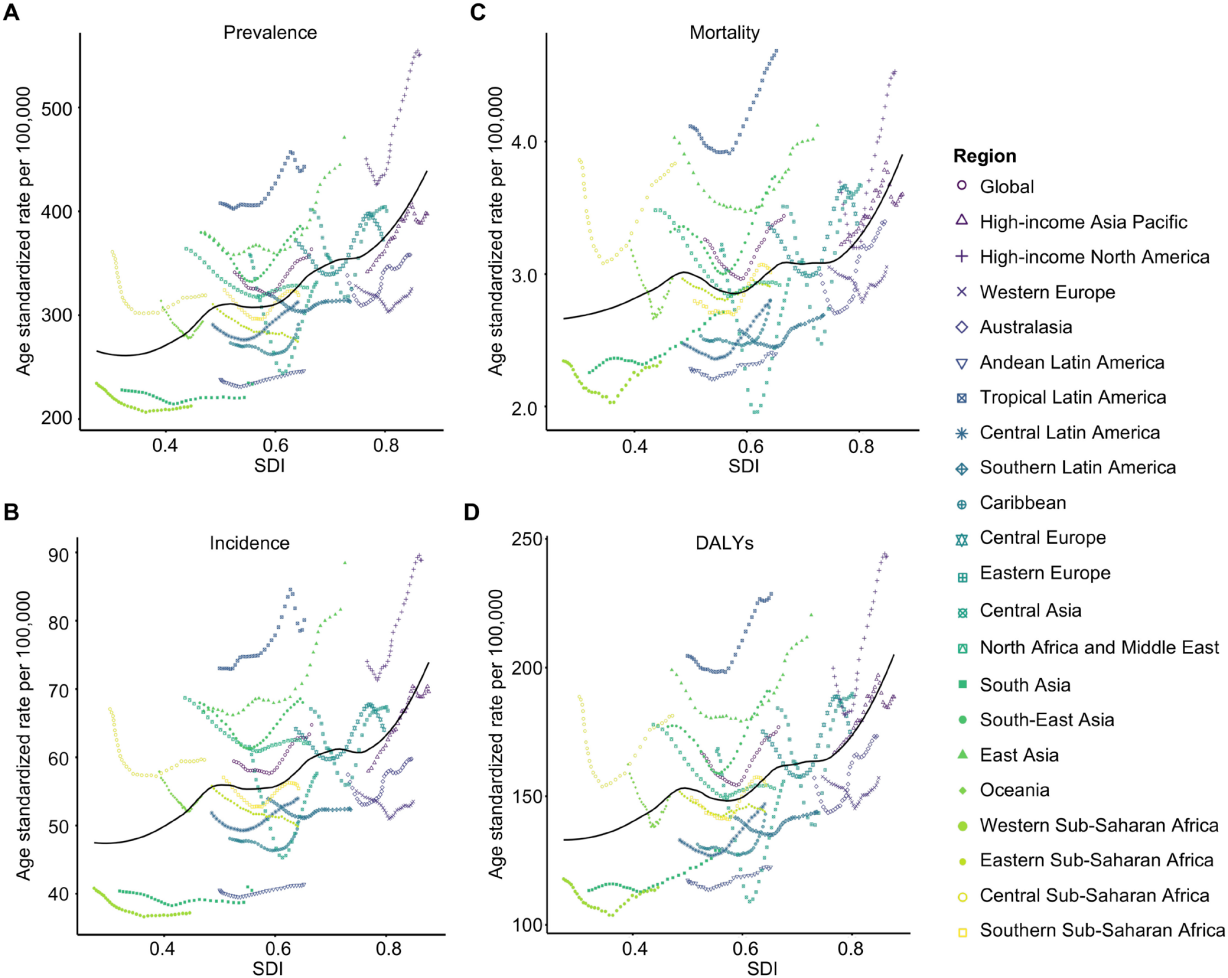
Age‐standardized prevalence, incidence, mortality, and DALY rates of early‐onset Alzheimer's disease and other dementias, globally and for 21 GBD Regions, 1990–2021. This figure depicts the age‐standardized rates (per 100,000 population) of prevalence, incidence, mortality, and disability‐adjusted life years (DALYs) for early‐onset Alzheimer's disease and other dementias (EOAD) in young adults aged 40–64 years across 21 GBD regions from 1990 to 2021. The solid black line represents the expected values, calculated based on the Socio‐Demographic Index (SDI) and disease rates across all locations. For each region, 31 points are plotted, showing the observed age‐standardized rates for prevalence, incidence, mortality, and DALYs of EOAD for each year from 1990 to 2021.

High SDI regions saw a significant increase in age‐standardized prevalence, from 366.3 per 100,000 in 1990 to 423.5 per 100,000 in 2021, with an AAPC of 0.65% (*p* < 0.001), and the incidence rate also rose significantly, with an AAPC of 0.63% (*p* < 0.001) (Table 1, Figure S7). DALYs trends mirrored those of prevalence and incidence, with high SDI regions showing the largest increase (AAPC = 0.61%, *p* < 0.001) and low SDI regions a decline (AAPC = −0.12%, *p* = 0.002). The relationship between SDI and the expected age‐standardized rates of EOAD, including prevalence, incidence, mortality, and DALYs, was predominantly positive, with the steepest increases observed in regions with higher SDI levels (Figure 5).

### 3.7 Risk Factors

A comprehensive analysis of global data from 1990 to 2021 identified three key risk factors contributing to the burden of DALYs associated with EOAD among individuals aged 40–64 years: high body mass index (BMI), elevated fasting plasma glucose levels, and smoking. Overall, these risk factors contributed to 106.87 million DALYs in 2021. The prevalence and impact of these risk factors varied across the 21 GBD regions, reflecting regional differences in health behaviors and outcomes (Figure S9).

## 4 Discussion

This study estimates that in 2021, there were 7.75 million prevalent cases and 1.38 million incident cases of EOAD across 204 countries and territories, representing more than double the figures reported in 1990. Our findings reveal modest yet consistent increases in the prevalence, incidence, mortality, and DALYs associated with EOAD over time, with significant disparities observed across countries with varying sociodemographic levels. Additionally, Slight differences in EOAD burden were observed between genders, with males having marginally higher prevalence and incidence rates.

The projected increase in dementia cases is mainly due to population aging, but our study shows a substantial global burden even in individuals under 65, with significant annual increases in prevalence and incidence. Previous studies report varying EOAD prevalence rates, ranging from 81 to 119 per 100,000 population in specific fields or regions [2, 24, 25]. In comparison, our study—marking the first to utilize the GBD dataset specifically for EOAD—reports a prevalence of 363.45 per 100,000 population in 2021. This variation may be due to differences in diagnostic criteria used in prevalence estimates [26]. Notably, the higher prevalence of EOAD observed in our study compared to previous reports may reflect improvements in EOAD identification and diagnosis over the past decade, driven by educational interventions aimed at enhancing primary care practices [2] and the broader availability of diagnostic biomarkers that were not widely used in clinical practice a decade ago. Additionally, the inclusion of more regions and populations in our study likely contributed to a more accurate representation of EOAD prevalence. In 2021, EOAD cases (7.75 million) accounted for 13.6% of global dementia cases (56.85 million), higher than previous estimates of 5.0%–7.0%, suggesting a greater, potentially underappreciated burden of EOAD [9, 27].

### 4.1 Age Differences in Burden of EOAD

Consistent with previous studies [2], our findings show that the EOAD burden increases with age, particularly in adults aged 50–64. While prevalence among those aged 40–64 rose, stratification revealed a decline in prevalence and incidence for 40–49 years old from 1990 to 2021. Despite this, mortality and DALYs significantly increased in this group. This disparity may be attributed to the underdiagnosis of EOAD in younger individuals, potentially due to the significant impact a diagnosis could have on their familial, occupational, and community responsibilities, or due to the atypical symptoms of EOAD in early life, as previously suggested [4, 28]. Underdiagnosis can delay intervention, resulting in more severe outcomes, including higher mortality and DALYs. Additionally, most dementia care networks are tailored to older patients, potentially overlooking the specific needs of individuals with EOAD [29, 30]. This inadequacy may contribute to the rising mortality and DALYs observed globally.

### 4.2 Sex Differences in Burden of EOAD

Our study found that women had a higher prevalence, incidence, mortality, and DALYs than men in 2021, consistent with trends observed in late‐onset dementias and previous studies [10, 31]. The gender disparity has been largely attributed to biological and socioeconomic differences between males and females [32, 33]. Although AAPC for prevalence, incidence, mortality, and DALYs from 1990 to 2021 was higher in men than women, indicating a greater increase in the global EOAD burden, this trend may not be solely due to gender differences. The higher rates of diabetes, cardiovascular disease, unhealthy lifestyles, and poor diets [34, 35, 36, 37], which are more prevalent among men, may partly contribute to the higher increase in EOAD burden. Additionally, women tend to engage more with healthcare services and adhere better to disease management protocols, such as for diabetes and cognitive decline, showing greater awareness and treatment adherence [38, 39]. This can help reduce EOAD risk in women and improve their prognosis.

### 4.3 Geographical Heterogeneity in Burden of EOAD

Geographical variations in dementia prevalence have been recognized, particularly the lower prevalence and incidence rates observed in regions with lower SDI levels [10]. Our study aligns with broader dementia research, showing that the EOAD burden correlates positively with SDI, with the highest burden in high SDI countries. Structural inequalities across nations likely contribute to disparities in prevalence. According to Alzheimer's Disease International, 60% of dementia cases are in low‐ and middle‐income countries, a figure expected to rise to 71% by 2050. Moreover, up to three‐quarters of those with dementia globally remain undiagnosed [40]. This trend is likely driven by lower diagnosis rates in low‐income settings compared to high‐income regions. Addressing the significant resource gaps in these countries, particularly in the management of EOAD, is crucial [41, 42]. However, the discussion of geographical differences needs caution because medical conditions vary greatly between regions and the prevalence may not be the true disease burden.

### 4.4 Risk Factors in Burden of EOAD

Analysis of the GBD dataset highlights high BMI, elevated fasting plasma glucose, and smoking as key contributors to EOAD in adults aged 40–64, emphasizing the challenge of controlling these risk factors. Many individuals with diabetes also struggle to maintain consistent glucose levels [43], and a substantial proportion of young adults are affected by overweight or obesity and engage in unhealthy behaviors such as smoking [35]. With no effective treatments for EOAD, prioritizing modifiable risk factors for prevention is crucial. The 2024 Lancet Commission update suggests that up to 45% of dementia cases could be prevented through early adulthood interventions (under 65) [44]. This highlights the potential to reduce the future EOAD burden by managing modifiable risk factors.

### 4.5 The Impact of the COVID‐19 Pandemic on EOAD

As shown in Figure 1, EOAD mortality and diagnoses in 2020 and 2021 were higher than before, with the diagnosis rate unaffected by COVID‐19‐related resource allocation. Several factors may explain this unexpected result. Although many routine medical activities were paused during the pandemic, dementia diagnoses did not completely stop. While there were delays early on, as healthcare systems returned to normal, delayed diagnoses were “caught up.” [45] Therefore, even during the peak of the pandemic, the overall number of dementia diagnoses may not have fallen significantly, as the recovery occurred in the later stages. A 2020 study in Barcelona found that despite a 2.5‐month pause in normal activities, patient visits remained similar, with an increase in non‐neurodegenerative diseases, including cognitive disturbances [46]. In addition, unlike late‐onset dementia populations (often reliant on institutional care), younger EOAD patients (aged 40–64) may have maintained access to outpatient neurology services via telemedicine [47]. Increased public awareness of cognitive symptoms during the pandemic might have prompted earlier help‐seeking behavior in some individuals, offsetting reductions in new diagnoses [48].

### 4.6 Strengths and Limitations

This study highlights the growing global burden of EOAD in adults aged 40–64 and emphasizes the need for targeted prevention strategies, especially for younger individuals. Timely diagnosis is crucial for reducing mortality and DALYs, and preventive actions should begin early and continue throughout life. This information is vital for public health planning and resource allocation for young adults affected by dementia.

However, this study has several limitations. First, The GBD dataset includes dementia data only for adults aged 40–64, excluding those under 40, limiting the applicability of our findings to younger individuals. However, previous reports show that EOAD cases in adults under 40 account for less than 1% [2]. Second, the EOAD definition varies by ICD codes, self‐reports, diagnoses by healthcare providers, and hospital records, with much data coming from epidemiological studies due to the lack of standardized dementia registries. This contributes to heterogeneity in case identification. Third, the rising global prevalence of EOAD may partially reflect better diagnostic tools, increased awareness among medical staff, and improved registration systems, except for true variations in prevalence. For example, regions like Afghanistan and Sub‐Saharan Africa may have underdiagnosis due to a lack of standardized practices, leading to underreporting. Caution is needed when interpreting regional variations. Future studies with standardized criteria are needed to more accurately assess the global burden of EOAD. The study focuses on the overall burden of EOAD due to the lack of data on dementia subtypes. While subtypes like stroke‐related dementia have different risk factors, the high prevalence of mixed pathologies and regional variations make total dementia a more suitable focus for global studies [49].

## 5 Conclusions

The global burden of EOAD among adults aged 40–64 years has more than doubled from 1990 to 2021, with substantial increases observed in prevalence, incidence, mortality, and DALYs. These findings underscore the urgent need for targeted public health strategies, especially in high‐risk populations and regions, to mitigate the growing burden of EOAD.

## Author Contributions

**Zenghui Zhang:** conceptualization, methodology, software, data curation, investigation, writing – original draft, writing – review and editing, formal analysis. **Shaojie Han:** conceptualization, methodology, formal analysis, writing – original draft, writing – review and editing. **Huimin Zhu:** writing – original draft, writing – review and editing, formal analysis, visualization. **Qianyun Wang:** writing – review and editing, validation. **Siyuan Cheng:** writing – review and editing, software. **Yuchen Han:** conceptualization, writing – review and editing. **Fengjuan Li:** writing – review and editing, supervision, project administration, resources. **Jun Guo:** funding acquisition, project administration, resources.

## Ethics Statement

The authors have nothing to report.

## Consent

The authors have nothing to report.

## Conflicts of Interest

The authors declare no conflicts of interest.

## Supporting information


Appendix S1

## Data Availability

The datasets generated and/or analyzed during the current study are available from the website https://vizhub.healthdata.org/gbd‐results/. As this study did not involve patient‐specific data, informed consent was not required.
