# Supplementary material for: Global, Regional, and National Burden of Early‐Onset Alzheimer's Disease and Other Dementias in Young Adults Aged 40–64 Years, 1990–2021: A Population‐Based Study
Source: Eur J Neurol. 2025 Mar 19;32(3):e70116. doi: 10.1111/ene.70116 (PMC11921012; doi:10.1111/ene.70116)
Supplement: Supplementary file 1 — Appendix S1 [file ENE-32-e70116-s001.docx]

**Supplementary materials**


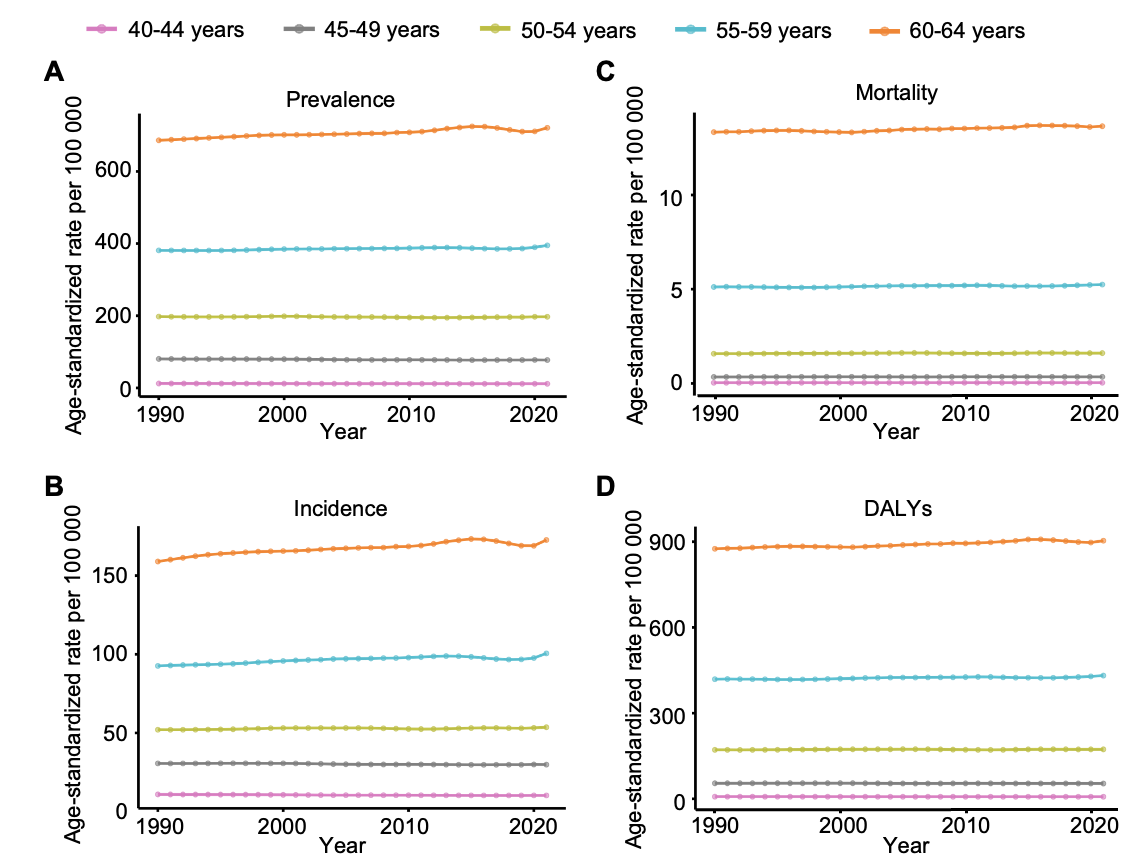


**Figure S1. Global Trends in the Age-Standardized Rate of Prevalence, Incidence, Mortality, and DALYs of Early-Onset Alzheimer's Disease and Other Dementias by Age Group, 1990–2021.**

The global age-standardized rates (per 100 000 population) of (A) prevalence, (B) incidence, (C) mortality, and (D) disability-adjusted life years (DALYs) associated with early-onset Alzheimer’s disease and other dementias (EOAD) across five age groups: 40–44 years (pink line), 45–49 years (grey line), 50–54 years (green line), 55–59 years (blue line), and 60–64 years (orange line).


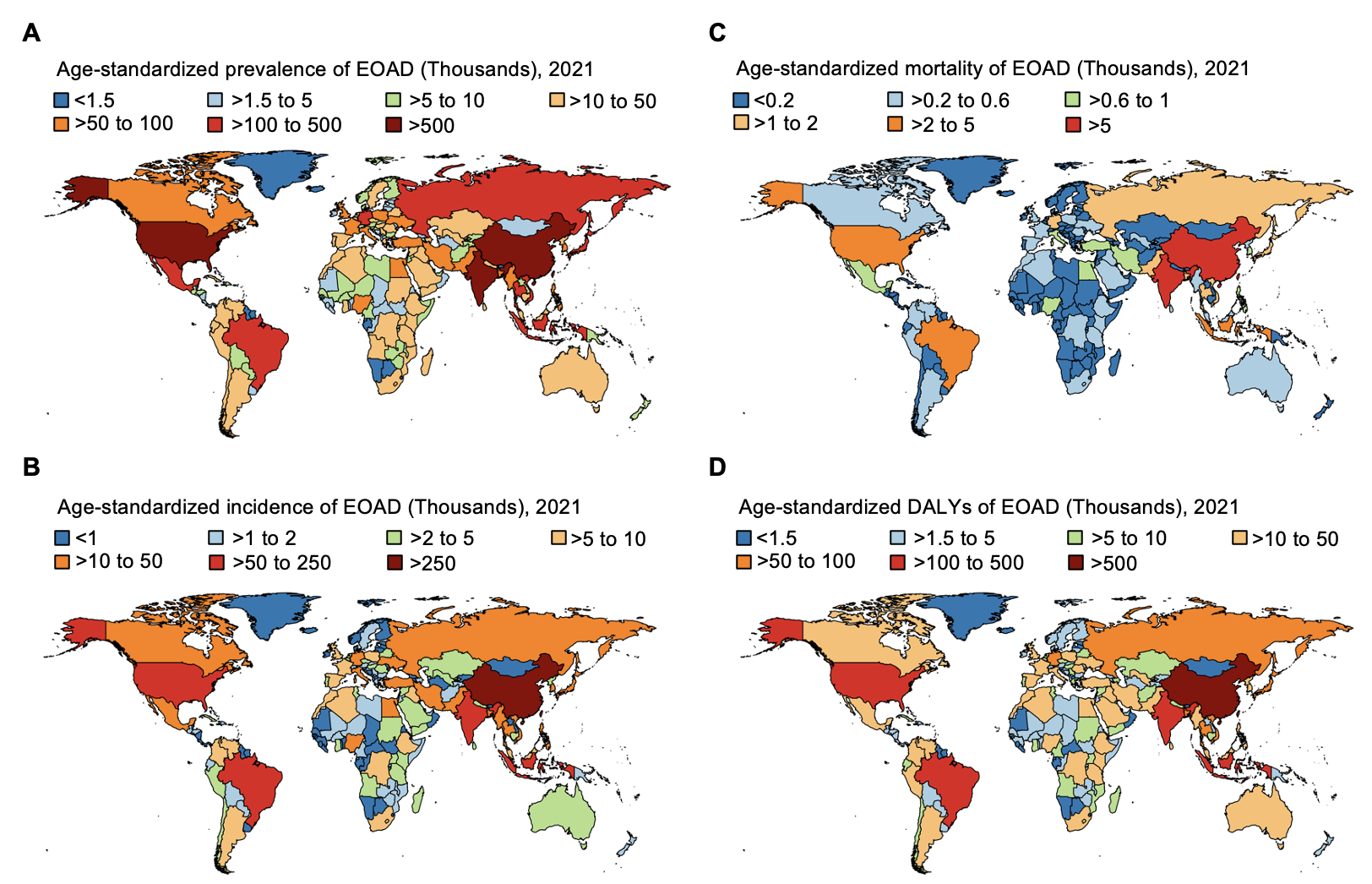


**Figure S2. Age-standardized Numbers of Prevalence, Incidence, Mortality, and DALYs of Early-Onset Alzheimer's Disease and Other Dementias in 2021.**

The age-standardized numbers of (A) prevalence, (B) incidence, (C) mortality, and (D) disability-adjusted life years (DALYs) associated with early-onset Alzheimer's disease and other dementias(EOAD) among populations aged 40-64 years in 2021.


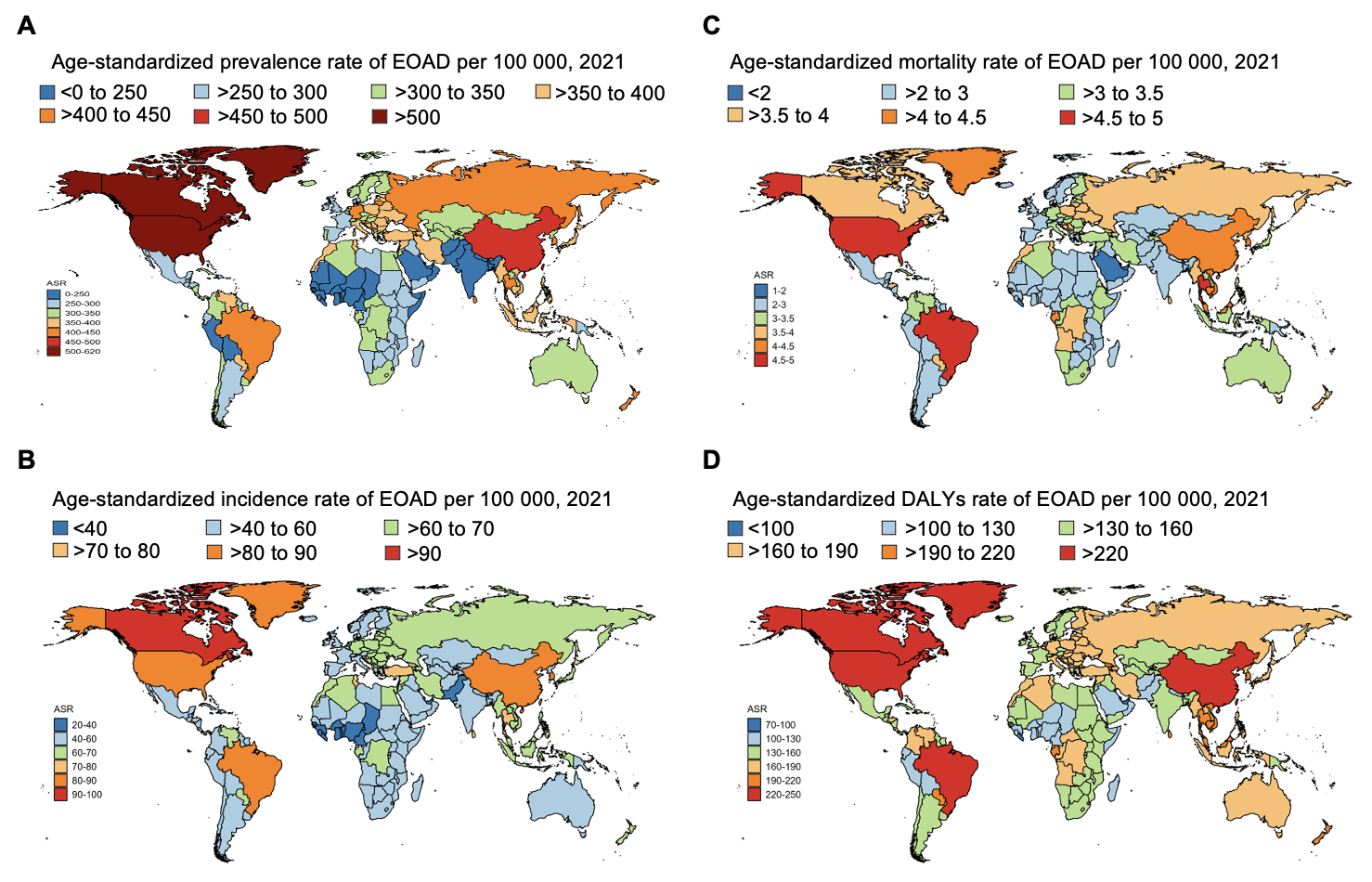


**Figure S3. Age-standardized Rates of Prevalence, Incidence, Mortality, and DALYs of Early-Onset Alzheimer's Disease and Other Dementias in 2021.**

The age-standardized rates (per 100000 population) of (A) prevalence, (B) incidence, (C) mortality, and (D) disability-adjusted life years (DALYs) associated with early-onset Alzheimer's disease and Other Dementias (EOAD) among populations aged 40-64 years in 2021.


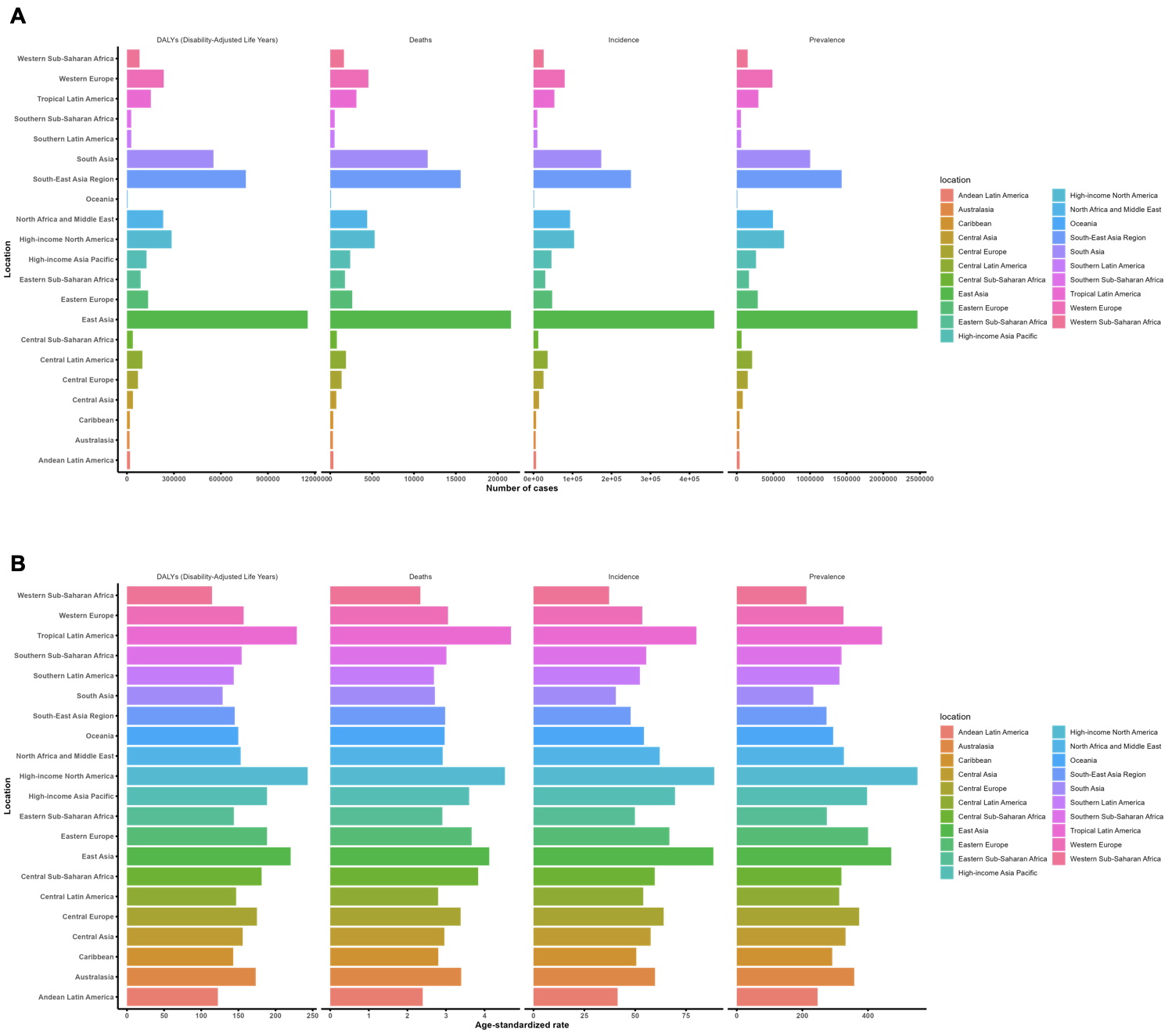


**Figure S4. Age-standardized Numbers and Rates of Prevalence, Incidence, Mortality, and DALYs of Early-Onset Alzheimer's Disease and Other Dementias in 2021.**

The age-standardized (A) number and (B) rates (per 100000 population) of prevalence, incidence, mortality, and disability-adjusted life years (DALYs) associated with early-onset Alzheimer's disease and other dementias (EOAD) among populations aged 40-64 years in 2021.


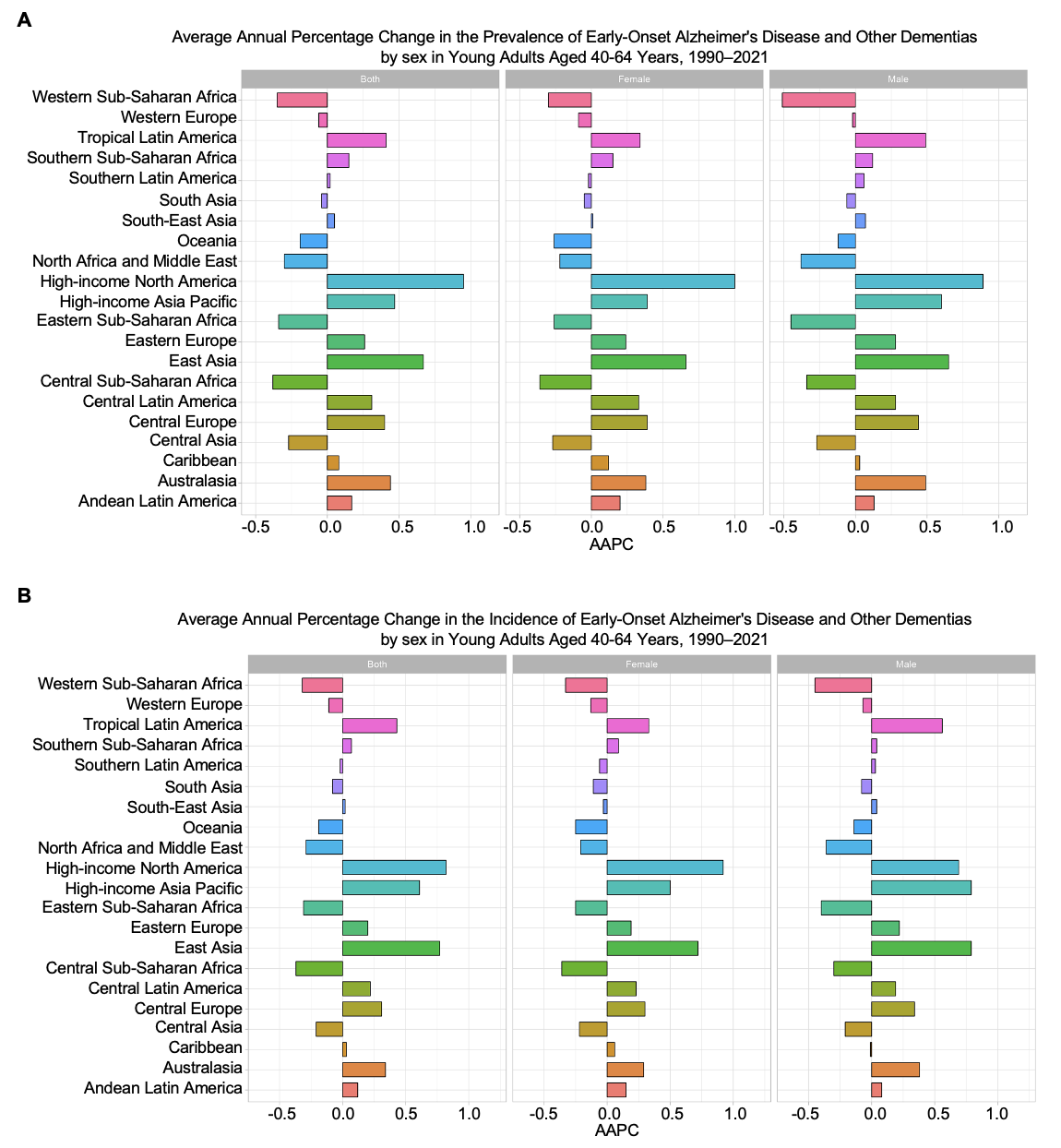


**Figure S5. Average Annual Percentage Change in the Prevalence and Incidence of Early-Onset Alzheimer's Disease and Other Dementias by Sex, 1990–2021.**

This figure illustrates the Average Annual Percentage Change (AAPC) in the prevalence and incidence of early-onset Alzheimer's disease and other dementias(EOAD) by sex in young adults aged 40-64 years, over the period from 1990 to 2021. The data are presented separately for males and females, providing a comparison between the years 1990 and 2021.


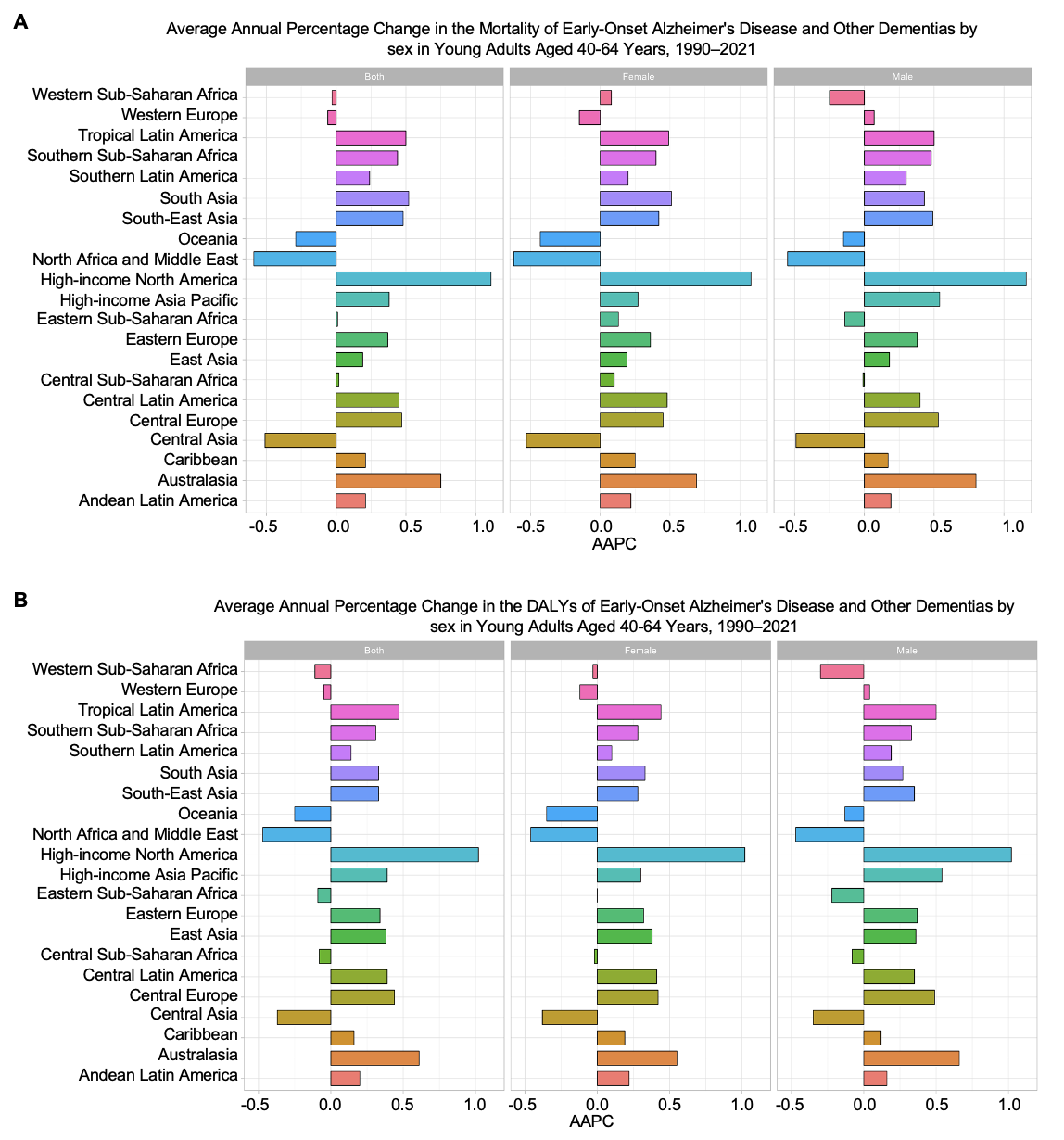


**Figure S6. Average Annual Percentage Change in Mortality and DALYs of Early-Onset Alzheimer's Disease and Other Dementias by Sex, 1990–2021.**

This figure illustrates the Average Annual Percentage Change (AAPC) in mortality and disability-adjusted life years (DALYs) associated with early-onset Alzheimer's disease and other dementias (EOAD) in young adults aged 40-64 years by sex, over the period from 1990 to 2021. The data are presented separately for males and females, offering a comparison between the years 1990 and 2021.


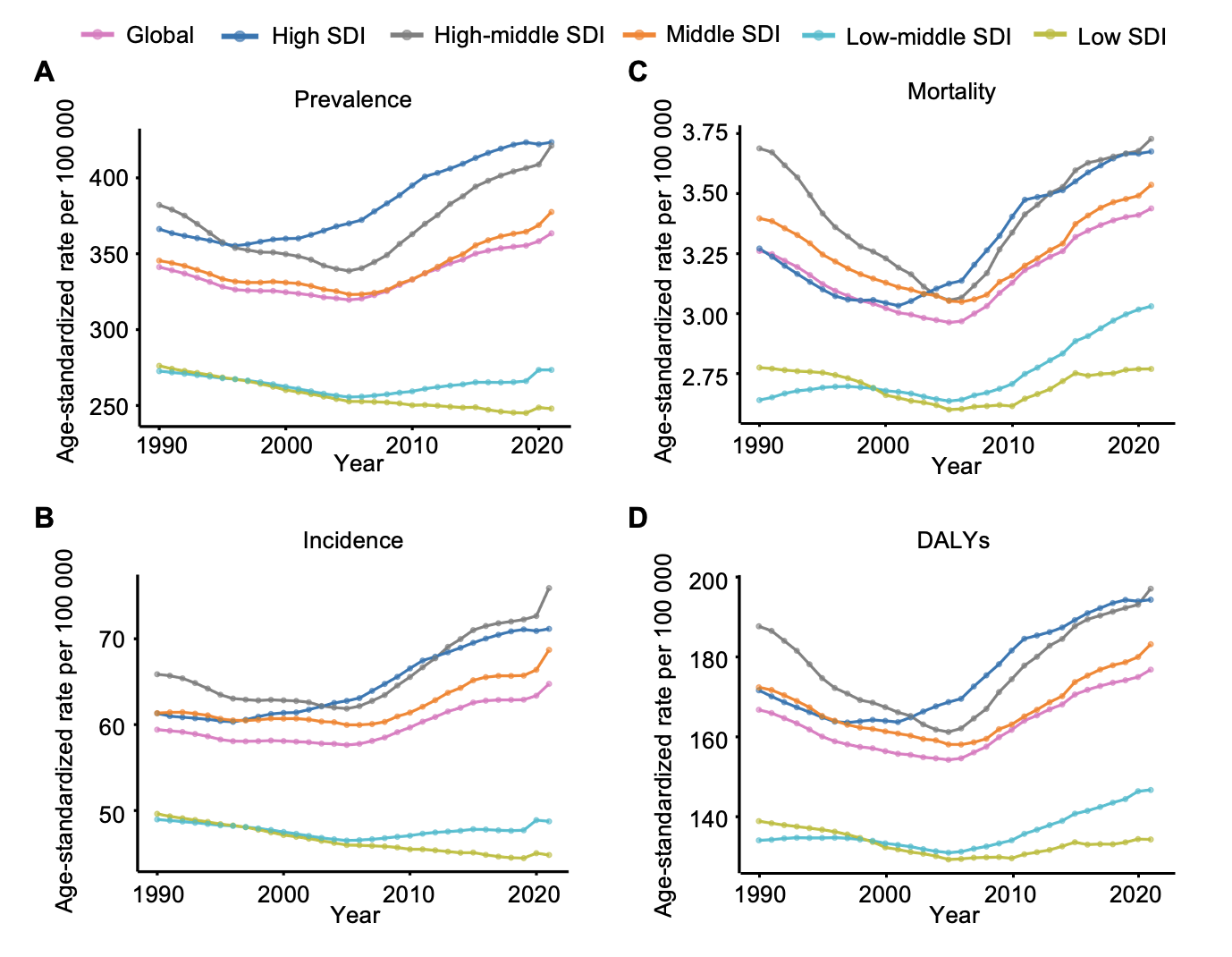


**Figure S7. Global Trends in the Age-Standardized Rates of Prevalence, Incidence, Mortality, and DALYs of Early-Onset Alzheimer's Disease and Other Dementias by SDI Group, 1990–2021.**

The global trends in the age-standardized rates (per 100 000 population) of (A) prevalence, (B) incidence, (C) mortality, and (D) disability-adjusted life years (DALYs) associated with early-onset Alzheimer's disease and other dementias (EOAD) in Young Adults Aged 40-64 Years across six Socio-Demographic Index (SDI) groups: global (pink line), high SDI (dark blue line), high-middle SDI (grey line), middle SDI (orange line), low-middle SDI (blue line), and low SDI (green line).


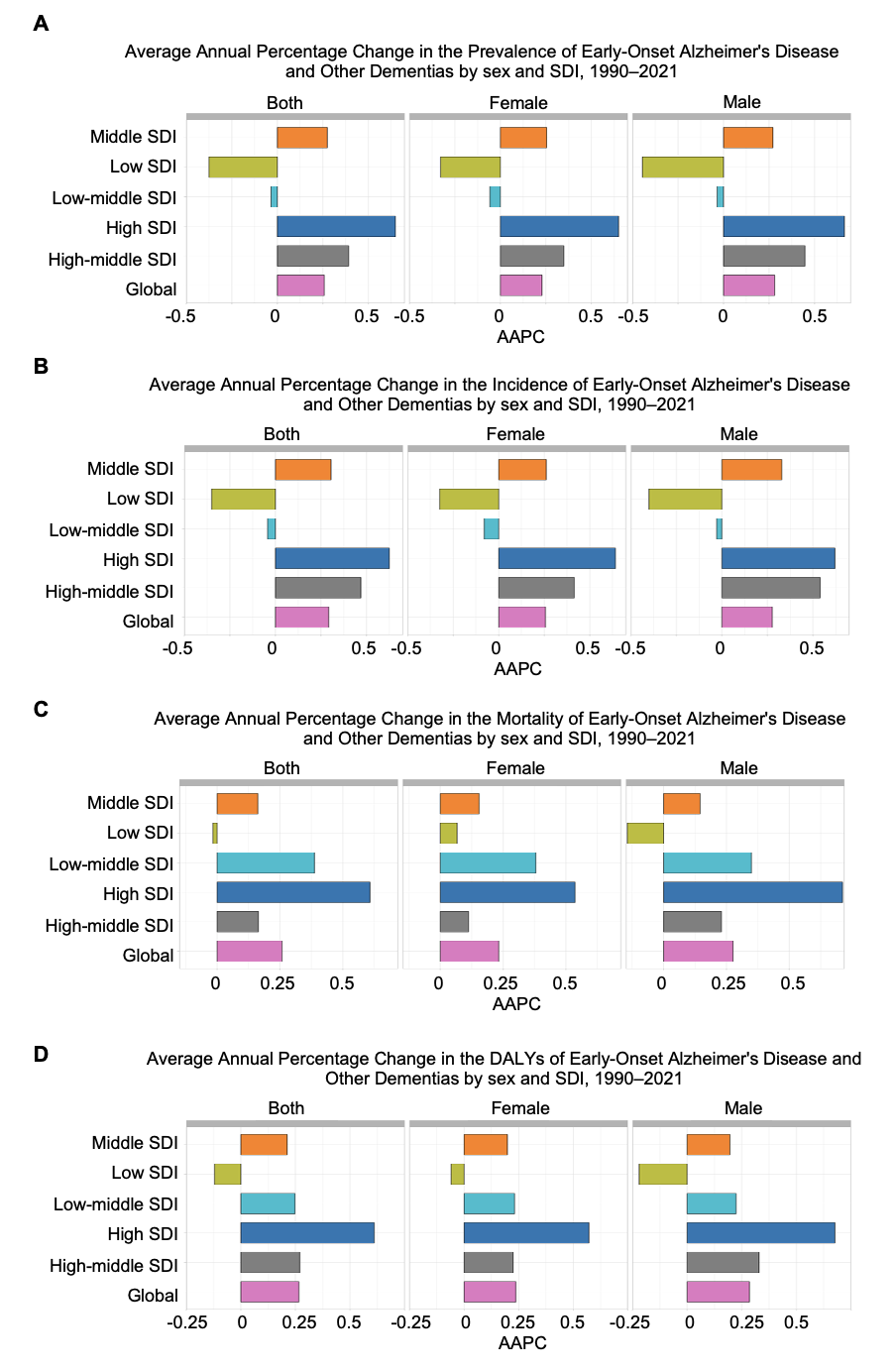


**Figure S8. Average Annual Percentage Change in the Prevalence, Incidence, Mortality, and DALYs of Early-Onset Alzheimer's Disease and Other Dementias by Sex and SDI, 1990–2021.**

The global trends in the average annual percentage change of the age-standardized rates of (A) prevalence, (B) incidence, (C) mortality, and (D) disability-adjusted life years (DALYs) associated with early-onset Alzheimer's disease and other dementias (EOAD) in Young Adults Aged 40-64 Years across six Socio-Demographic Index (SDI) groups. The data are presented separately for males and females, with trends shown for each SDI group: global, high SDI, high-middle SDI, middle SDI, low-middle SDI, and low SDI.


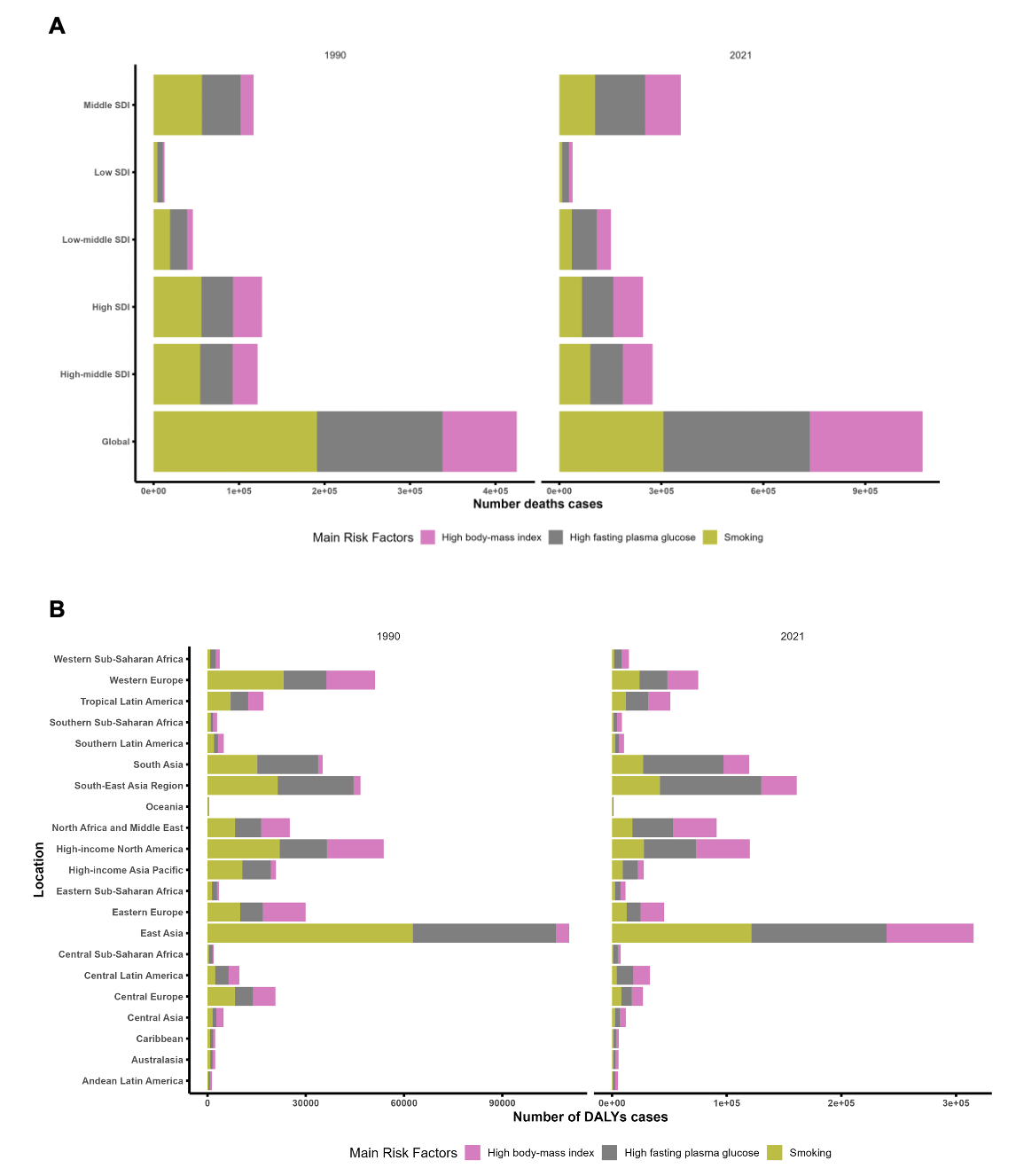


**Figure S9. Main Risk Factors for Age-Standardized EOAD-Related DALYs Among Populations aged 40-64 years by SDI and Region, 1990–2021.**

This figure illustrates the main risk factors—high body mass index, high fasting plasma glucose, and smoking—contributing to age-standardized disability-adjusted life years (DALYs) related to early-onset Alzheimer’s disease and other dementias (EOAD) among populations aged 40-64 years. The data are presented by Socio-Demographic Index (SDI) and region, comparing the years 1990 and 2021.

**Supplementary Table S1. Age-standardized incidence of EOAD in people aged 40-64 years and their AAPCs from 1990 to 2021 at the global and SDI levels.**

|  | Incidence | | | | | |
| --- | --- | --- | --- | --- | --- | --- |
|  | No of people with EOAD in 1990 (000s) | Age standardised rate  in 1990 (per 100 000) | No of people with  EOAD in 2021 (000s) | Age standardised rate  in 2021 (per 100 000) | AAPC (95% CI) | *p* value |
| Global | 639.95 (413.42 to 912.20) | 59.42 (38.38 to 84.69) | 1381.91 (894.77 to 1963.45) | 64.74 (41.92 to 91.99) | 0.29 (0.21 to 0.38) | <0.001 |
| Sex: |  |  |  |  |  |  |
| Female | 354.36 (229.55 to 504.86) | 66.44 (43.04 to 94.66) | 764.45 (495.45 to 1084.96) | 71.3 (46.21 to 101.19) | 0.26 (0.17 to 0.34) | <0.001 |
| Male | 285.60 (183.47 to 408.05) | 52.52 (33.74 to 75.04) | 617.46 (399.55 to 878.80) | 58.13 (37.61 to 82.73) | 0.28 (0.12 to 0.44) | 0.001 |
| Age group (years): |  |  |  |  |  |  |
| 40-44 | 31.32 (13.81 to 52.56) | 10.93 (4.82 to 18.35) | 51.4 (22.31 to 87.52) | 10.28 (4.46 to 17.5) | -0.22 (-0.24 to -0.2) | <0.001 |
| 45-49 | 71.13 (44.76 to 104.26) | 30.63 (19.28 to 44.9) | 141.41 (88.18 to 208.69) | 29.87 (18.62 to 44.07) | -0.12 (-0.14 to -0.1) | <0.001 |
| 50-54 | 110.65 (71.88 to 158.45) | 52.05 (33.82 to 74.54) | 238.61 (153.1 to 340.78) | 53.63 (34.41 to 76.59) | 0.06 (0.04 to 0.08) | <0.001 |
| 55-59 | 171.52 (113.3 to 242.17) | 92.61 (61.18 to 130.76) | 397.94 (263.07 to 565.03) | 100.56 (66.48 to 142.78) | 0.2 (0.16 to 0.24) | <0.001 |
| 60-64 | 255.34 (169.66 to 354.75) | 10.93 (4.82 to 18.35) | 552.54 (368.11 to 761.43) | 172.64 (115.02 to 237.91) | 0.22 (0.19 to 0.26) | <0.001 |
| SDI level: |  |  |  |  |  |  |
| High | 148.84 (95.95 to 213.04) | 61.31 (39.52 to 87.76) | 259.12 (168.88 to 368.63) | 71.16 (46.37 to 101.23) | 0.63 (0.55 to 0.7) | <0.001 |
| High-middle | 167.55 (108.5 to 238.56) | 65.86 (42.65 to 93.78) | 340.84 (221.48 to 484.22) | 75.9 (49.32 to 107.83) | 0.47 (0.31 to 0.63) | <0.001 |
| Middle | 193.52 (124.9 to 275.05) | 61.34 (39.59 to 87.18) | 497.45 (321.11 to 706.02) | 68.7 (44.35 to 97.51) | 0.31 (0.21 to 0.4) | <0.001 |
| Low-middle | 93.75 (59.93 to 134.23) | 48.96 (31.3 to 70.09) | 206.78 (132.06 to 297.03) | 48.73 (31.12 to 70) | -0.04 (-0.1 to 0.02) | 0.149 |
| Low | 35.62 (22.75 to 51.03) | 49.61 (31.69 to 71.08) | 76.67 (48.48 to 110.6) | 44.82 (28.33 to 64.64) | -0.35 (-0.38 to -0.32) | <0.001 |

Note: SDI, Socio-Demographic Index; CI, confidence interval; AAPC, average annual percentage change; EOAD, early-onset Alzheimer’s disease and other dementias.

**Supplementary Table S2. Age-standardized mortality and DALYs of EOAD in people aged 40-64 years and their AAPCs from 1990 to 2021 at the global and SDI levels.**

|  | Mortality | | | | | | DALYs | | | | | |
| --- | --- | --- | --- | --- | --- | --- | --- | --- | --- | --- | --- | --- |
|  | No of people with EOAD in 1990 (000s) | Age standardised rate  in 1990 (per 100 000) | No of people with  EOAD in 2021 (000s) | Age standardised rate  in 2021 (per 100 000) | AAPC (95% CI) | *p* value | No of people with EOAD in 1990 (000s) | Age standardised rate  in 1990 (per 100 000) | No of people with  EOAD in 2021 (000s) | Age standardised rate  in 2021 (per 100 000) | AAPC (95% CI) | *p* value |
| Global | 35.12 (6.72 to 110.34) | 3.26 (0.62 to 10.24) | 73.39 (14.06 to 232.17) | 3.44 (0.66 to 10.88) | 0.26 (0.1 to 0.42) | 0.003 | 1796.30 (814.02 to 4150.46) | 166.78 (175.58 to 385.35) | 3774.12 (1696.44 to 8881.15) | 176.82 (79.48 to 416.09) | 0.27 (0.13 to 0.4) | <0.001 |
| Sex: |  |  |  |  |  |  |  |  |  |  |  |  |
| Female | 19.44 (3.77 to 60.34) | 3.64 (0.71 to 11.31) | 40.89 (7.98 to 127.32) | 3.81 (0.74 to 11.87) | 0.23 (0.07 to 0.39) | 0.006 | 1006.49 (458.25 to 2285.45) | 188.72 (85.92 to 428.53) | 2124.79 (954.09 to 4885.44) | 198.17 (88.98 to 455.65) | 0.24 (0.1 to 0.37) | 0.001 |
| Male | 15.69 (2.97 to 49.41) | 2.88 (0.55 to 9.09) | 32.50 (6.16 to 102.45) | 3.06 (0.58 to 9.64) | 0.28 (0.12 to 0.44) | 0.001 | 789.81 (344.43 to 1874.26) | 145.25 (63.34 to 344.7) | 1649.33 (725.79 to 3926.95) | 155.27 (68.32 to 369.68) | 0.28 (0.15 to 0.42) | <0.001 |
| Age group (years): |  |  |  |  |  |  |  |  |  |  |  |  |
| 40-44 | 0.1 (0.01 to 0.34) | 0.03 (0 to 0.12) | 0.17 (0.02 to 0.63) | 0.03 (0 to 0.13) | 0.06 (0.04 to 0.08) | <0.001 | 14.42 (6.28 to 28.47) | 5.03 (2.19 to 9.94) | 24.37 (10.53 to 49.69) | 4.87 (2.1 to 9.93) | -0.13 (-0.14 to -0.11) | <0.001 |
| 45-49 | 0.79 (0.11 to 2.68) | 0.34 (0.05 to 1.16) | 1.64 (0.22 to 5.74) | 0.35 (0.05 to 1.21) | 0.04 (0.02 to 0.07) | 0.001 | 84.2 (43.6 to 165.77) | 36.26 (18.78 to 71.39) | 169.08 (86.41 to 347.56) | 35.71 (18.25 to 73.4) | -0.07 (-0.08 to -0.05) | <0.001 |
| 50-54 | 3.35 (0.53 to 11.31) | 1.57 (0.25 to 5.32) | 7.14 (1.14 to 24.13) | 1.61 (0.26 to 5.42) | 0.06 (0.04 to 0.08) | <0.001 | 243.1 (111.93 to 552.87) | 114.36 (52.65 to 260.09) | 513.73 (232.66 to 1160.02) | 115.47 (52.29 to 260.72) | 0.02 (0 to 0.04) | 0.02 |
| 55-59 | 9.48 (1.76 to 29.62) | 5.12 (0.95 to 16) | 20.75 (3.81 to 65.58) | 5.24 (0.96 to 16.57) | 0.07 (0.05 to 0.09) | <0.001 | 517.15 (239.55 to 1195.85) | 279.24 (129.35 to 645.71) | 1138.56 (515.36 to 2753.87) | 287.71 (130.23 to 695.9) | 0.08 (0.06 to 0.1) | <0.001 |
| 60-64 | 21.42 (4.32 to 66.38) | 13.33 (2.69 to 41.33) | 43.68 (8.88 to 136.09) | 13.65 (2.77 to 42.52) | 0.09 (0.07 to 0.1) | <0.001 | 937.43 (412.66 to 2207.49) | 583.67 (256.93 to 1374.45) | 1928.37 (851.49 to 4570.02) | 602.52 (266.05 to 1427.92) | 0.11 (0.1 to 0.13) | <0.001 |
| SDI level: |  |  |  |  |  |  |  |  |  |  |  |  |
| High | 7.94 (1.5 to 25.06) | 3.27 (0.62 to 10.32) | 13.38 (2.6 to 41.84) | 3.68 (0.72 to 11.49) | 0.19 (-0.02 to 0.4) | 0.069 | 416.7 (193.68 to 950.1) | 171.65 (79.78 to 391.38) | 707.68 (330.33 to 1596.86) | 194.33 (90.71 to 438.5) | 0.61 (0.49 to 0.73) | <0.001 |
| High-middle | 9.38 (1.82 to 28.85) | 3.69 (0.72 to 11.34) | 16.74 (3.24 to 52.9) | 3.73 (0.72 to 11.78) | 0.23 (0.08 to 0.37) | 0.004 | 477.42 (217.4 to 1090.82) | 187.68 (85.46 to 428.81) | 885.05 (403.13 to 2060.91) | 197.09 (89.77 to 458.93) | 0.27 (0.05 to 0.49) | 0.019 |
| Middle | 10.72 (2.04 to 33.34) | 3.4 (0.65 to 10.57) | 25.61 (4.9 to 80.94) | 3.54 (0.68 to 11.18) | 0.33 (0.24 to 0.43) | <0.001 | 543.85 (243.7 to 1258.69) | 172.38 (77.24 to 398.96) | 1326.32 (596.33 to 3126.02) | 183.18 (82.36 to 431.73) | 0.21 (0.06 to 0.36) | 0.007 |
| Low-middle | 5.05 (0.96 to 16.08) | 2.64 (0.5 to 8.39) | 12.86 (2.5 to 39.99) | 3.03 (0.59 to 9.42) | 0.3 (0.15 to 0.46) | <0.001 | 256.77 (114.11 to 609.73) | 134.08 (59.59 to 318.39) | 622.43 (269.35 to 1482.1) | 146.69 (63.48 to 349.29) | 0.25 (0.16 to 0.34) | <0.001 |
| Low | 1.99 (0.38 to 6.36) | 2.78 (0.53 to 8.86) | 4.74 (0.92 to 14.75) | 2.77 (0.54 to 8.62) | 0.25 (0.04 to 0.47) | 0.022 | 99.72 (43.66 to 239.21) | 138.9 (60.82 to 333.2) | 229.77 (97.62 to 553.08) | 134.3 (57.06 to 323.28) | -0.12 (-0.19 to -0.05) | 0.002 |

Note: SDI, Socio-Demographic Index; CI, confidence interval; AAPC, average annual percentage change; EOAD, early-onset Alzheimer’s disease and other dementias; DALYs, disability-adjusted life years.

**Supplementary Table S3. Age-standardized prevalence and incidence of EOAD in people aged 40-64 years and their AAPCs from 1990 to 2021 at the regional levels.**

|  | Prevalence | | | | | | Incidence | | | | | |
| --- | --- | --- | --- | --- | --- | --- | --- | --- | --- | --- | --- | --- |
| Region | No of people with EOAD in 1990 (000s) | Age standardised rate in 1990 (per 100 000) | No of people with EOAD in 2021 (000s) | Age standardised rate in 2021 (per 100 000) | AAPC (95% CI) | *p* value | No of people with EOAD in 1990 (000s) | Age standardised rate in 1990 (per 100 000) | No of people with EOAD in 2021 (000s) | Age standardised rate in 2021 (per 100 000) | AAPC (95% CI) | *p* value |
| Western Sub-Saharan Africa | 63.29 (46.26 to 83.7) | 234.38 (171.29 to 309.93) | 149.18 (108.24 to 197.24) | 212.55 (154.22 to 281.03) | -0.35 (-0.43 to -0.27) | <0.001 | 11.02 (6.93 to 16.02) | 40.81 (25.67 to 59.33) | 26.11 (16.23 to 38.13) | 37.2 (23.13 to 54.33) | -0.32 (-0.39 to -0.26) | <0.001 |
| Western Europe | 372.69 (279 to 482.89) | 328.26 (245.74 to 425.32) | 486.53 (357.91 to 636.58) | 325.39 (239.37 to 425.75) | -0.06 (-0.15 to 0.02) | 0.131 | 62.5 (39.51 to 90.16) | 55.05 (34.8 to 79.41) | 80 (50.15 to 117.31) | 53.5 (33.54 to 78.46) | -0.11 (-0.18 to -0.04) | 0.003 |
| Tropical Latin America | 112.22 (84.12 to 146.19) | 407.89 (305.74 to 531.33) | 295.82 (222.27 to 385.83) | 443 (332.86 to 577.79) | 0.41 (0.32 to 0.5) | <0.001 | 20.09 (13.04 to 28.39) | 73.03 (47.4 to 103.18) | 53.5 (34.73 to 76.29) | 80.12 (52 to 114.25) | 0.43 (0.34 to 0.52) | <0.001 |
| Southern Sub-Saharan Africa | 25.93 (19.26 to 34.31) | 324.21 (240.75 to 428.93) | 56.56 (41.86 to 74.9) | 319.32 (236.3 to 422.85) | 0.15 (0.02 to 0.27) | 0.021 | 4.56 (2.92 to 6.6) | 57.04 (36.5 to 82.49) | 9.82 (6.27 to 14.15) | 55.43 (35.39 to 79.89) | 0.07 (-0.02 to 0.17) | 0.134 |
| Southern Latin America | 36.63 (27.19 to 47.74) | 320.4 (237.77 to 417.52) | 60.4 (45.04 to 78.98) | 313.03 (233.45 to 409.34) | 0.02 (-0.05 to 0.08) | 0.612 | 6.17 (3.89 to 8.94) | 53.92 (34.01 to 78.19) | 10.1 (6.37 to 14.63) | 52.32 (33 to 75.81) | -0.02 (-0.08 to 0.03) | 0.392 |
| South Asia | 428.8 (310.22 to 567.8) | 228 (164.95 to 301.9) | 1002.29 (724.36 to 1327.98) | 233.62 (168.84 to 309.54) | -0.04 (-0.12 to 0.05) | 0.408 | 75.99 (47.75 to 110.26) | 40.4 (25.39 to 58.63) | 173.75 (108.11 to 254.07) | 40.5 (25.2 to 59.22) | -0.08 (-0.14 to -0.01) | 0.031 |
| South-East Asia Region | 607.88 (448.08 to 799.84) | 263.25 (194.04 to 346.38) | 1431.71 (1050.52 to 1885.89) | 273.68 (200.81 to 360.5) | 0.05 (-0.05 to 0.16) | 0.313 | 108.16 (69.08 to 155.04) | 46.84 (29.91 to 67.14) | 249.85 (159.35 to 360.14) | 47.76 (30.46 to 68.84) | 0.02 (-0.06 to 0.1) | 0.605 |
| Oceania | 3.19 (2.38 to 4.19) | 313.81 (233.52 to 411.87) | 8 (5.99 to 10.58) | 293.9 (220.1 to 388.56) | -0.19 (-0.29 to -0.09) | 0.001 | 0.59 (0.37 to 0.84) | 57.88 (36.62 to 82.28) | 1.48 (0.95 to 2.13) | 54.31 (34.83 to 78.05) | -0.19 (-0.28 to -0.11) | <0.001 |
| North Africa and Middle East | 191.19 (144.05 to 249.04) | 364.11 (274.34 to 474.29) | 494.03 (372.1 to 645.57) | 326.63 (246.01 to 426.83) | -0.3 (-0.41 to -0.19) | <0.001 | 36.08 (23.3 to 51.23) | 68.71 (44.37 to 97.57) | 93.87 (60.53 to 133.95) | 62.06 (40.02 to 88.57) | -0.29 (-0.38 to -0.19) | <0.001 |
| High-income North America | 324.59 (246.01 to 418.39) | 450.47 (341.42 to 580.65) | 645.9 (494.05 to 824.78) | 551.03 (421.47 to 703.63) | 0.95 (0.81 to 1.09) | <0.001 | 53.34 (34.72 to 76.11) | 74.03 (48.18 to 105.62) | 104.17 (68.41 to 146.65) | 88.87 (58.36 to 125.11) | 0.82 (0.72 to 0.92) | <0.001 |
| High-income Asia Pacific | 182.28 (135.98 to 238.32) | 341.91 (255.06 to 447.03) | 263.64 (197.77 to 341.48) | 397.39 (298.11 to 514.72) | 0.47 (0.37 to 0.57) | <0.001 | 30.91 (19.91 to 44.14) | 57.99 (37.34 to 82.8) | 46.18 (29.94 to 65.27) | 69.6 (45.13 to 98.38) | 0.61 (0.53 to 0.69) | <0.001 |
| Eastern Sub-Saharan Africa | 74.41 (55.46 to 97.56) | 310.59 (231.5 to 407.23) | 166.54 (123.34 to 219.63) | 274.64 (203.4 to 362.18) | -0.34 (-0.38 to -0.3) | <0.001 | 13.37 (8.62 to 19.23) | 55.81 (35.99 to 80.28) | 30.24 (19.18 to 43.5) | 49.87 (31.64 to 71.73) | -0.31 (-0.34 to -0.28) | <0.001 |
| Eastern Europe | 264.58 (197.54 to 346.18) | 401.65 (299.88 to 525.51) | 287.06 (214.42 to 375.08) | 400.46 (299.12 to 523.24) | 0.26 (-0.05 to 0.56) | 0.097 | 44.2 (28.58 to 62.98) | 67.09 (43.39 to 95.61) | 47.9 (31.04 to 68.22) | 66.82 (43.3 to 95.17) | 0.2 (-0.03 to 0.44) | 0.085 |
| East Asia | 966.72 (720.05 to 1263.53) | 379.58 (282.73 to 496.12) | 2466.92 (1846.57 to 3230.44) | 471.07 (352.61 to 616.86) | 0.67 (0.48 to 0.86) | <0.001 | 169.98 (109.55 to 241.86) | 66.74 (43.02 to 94.97) | 463.26 (302.94 to 653.6) | 88.46 (57.85 to 124.81) | 0.77 (0.62 to 0.91) | <0.001 |
| Central Sub-Saharan Africa | 26.65 (19.8 to 34.96) | 360.98 (268.25 to 473.58) | 65.92 (48.94 to 86.57) | 319.06 (236.87 to 419.04) | -0.38 (-0.54 to -0.22) | <0.001 | 4.95 (3.17 to 7.18) | 67.06 (42.97 to 97.21) | 12.32 (7.87 to 17.75) | 59.62 (38.07 to 85.92) | -0.37 (-0.51 to -0.24) | <0.001 |
| Central Latin America | 73.69 (54.81 to 96.87) | 290.76 (216.3 to 382.27) | 209.72 (156.13 to 274.76) | 312.41 (232.58 to 409.29) | 0.31 (0.21 to 0.42) | <0.001 | 13.14 (8.33 to 18.95) | 51.83 (32.87 to 74.79) | 36.22 (23.03 to 52.23) | 53.95 (34.3 to 77.81) | 0.22 (0.14 to 0.3) | <0.001 |
| Central Europe | 132.5 (99.87 to 171.6) | 371.97 (280.38 to 481.74) | 150.08 (113.03 to 193.77) | 373.16 (281.04 to 481.8) | 0.4 (0.24 to 0.57) | <0.001 | 22.84 (14.74 to 32.77) | 64.13 (41.39 to 92) | 25.75 (16.73 to 36.72) | 64.02 (41.59 to 91.31) | 0.31 (0.18 to 0.44) | <0.001 |
| Central Asia | 44.34 (33.28 to 57.76) | 358.37 (268.96 to 466.82) | 82.01 (61.41 to 106.82) | 331.67 (248.37 to 432.02) | -0.27 (-0.72 to 0.18) | 0.229 | 7.61 (4.9 to 10.9) | 61.54 (39.63 to 88.13) | 14.25 (9.12 to 20.47) | 57.63 (36.89 to 82.8) | -0.21 (-0.57 to 0.14) | 0.23 |
| Caribbean | 18.46 (13.68 to 24.31) | 273.24 (202.42 to 359.8) | 37.83 (28.18 to 49.48) | 291.07 (216.81 to 380.72) | 0.08 (-0.02 to 0.17) | 0.109 | 3.25 (2.06 to 4.72) | 48.1 (30.5 to 69.84) | 6.57 (4.13 to 9.46) | 50.56 (31.79 to 72.78) | 0.03 (-0.05 to 0.11) | 0.468 |
| Australasia | 17.95 (13.24 to 23.43) | 338.31 (249.51 to 441.71) | 34.24 (25.45 to 44.53) | 358.08 (266.15 to 465.65) | 0.44 (0.33 to 0.55) | <0.001 | 3.05 (1.93 to 4.42) | 57.45 (36.4 to 83.36) | 5.71 (3.59 to 8.31) | 59.75 (37.55 to 86.9) | 0.34 (0.24 to 0.44) | <0.001 |
| Andean Latin America | 14.47 (10.65 to 19.11) | 238.11 (175.25 to 314.42) | 39.3 (29.01 to 51.69) | 246.51 (182 to 324.24) | 0.17 (0.13 to 0.22) | <0.001 | 2.46 (1.53 to 3.61) | 40.52 (25.19 to 59.45) | 6.6 (4.09 to 9.61) | 41.38 (25.64 to 60.27) | 0.12 (0.09 to 0.16) | <0.001 |

Note: CI, confidence interval; AAPC, average annual percentage change; EOAD, early-onset Alzheimer’s disease and other dementias.

**Supplementary Table S4. Age-standardized mortality and DALYs of AD in people aged 40-64 years and their AAPCs from 1990 to 2021 at the regional levels.**

|  | Mortality | | | | | | DALYs | | | | | |
| --- | --- | --- | --- | --- | --- | --- | --- | --- | --- | --- | --- | --- |
| Region | No of people with AD in 1990 (000s) | Age standardised rate in 1990 (per 100 000) | No of people with AD in 2021 (000s) | Age standardised rate in 2021 (per 100 000) | AAPC (95% CI) | *p* value | No of people with AD in 1990 (000s) | Age standardised rate in 1990 (per 100 000) | No of people with AD in 2021 (000s) | Age standardised rate in 2021 (per 100 000) | AAPC (95% CI) | *p* value |
| Western Sub-Saharan Africa | 0.63 (0.12 to 2) | 2.34 (0.44 to 7.41) | 1.64 (0.31 to 5.1) | 2.34 (0.44 to 7.27) | 0.71 (0.52 to 0.89) | <0.001 | 31.82 (13.8 to 75.38) | 117.83 (51.1 to 279.14) | 80.4 (33.93 to 196.02) | 114.55 (48.34 to 279.29) | -0.11 (-0.25 to 0.03) | 0.124 |
| Western Europe | 3.46 (0.66 to 10.82) | 3.05 (0.58 to 9.53) | 4.56 (0.89 to 13.95) | 3.05 (0.6 to 9.33) | 0.52 (0.41 to 0.62) | <0.001 | 178.88 (81.94 to 407.8) | 157.56 (72.17 to 359.19) | 234.9 (107.17 to 536.13) | 157.1 (71.68 to 358.57) | -0.05 (-0.14 to 0.03) | 0.201 |
| Tropical Latin America | 1.13 (0.22 to 3.54) | 4.11 (0.8 to 12.85) | 3.13 (0.61 to 9.64) | 4.68 (0.92 to 14.43) | 0.5 (0.35 to 0.65) | <0.001 | 56.28 (25.23 to 132.27) | 204.55 (91.71 to 480.76) | 152.67 (67.57 to 358.08) | 228.63 (101.19 to 536.24) | 0.47 (0.35 to 0.59) | <0.001 |
| Southern Sub-Saharan Africa | 0.22 (0.04 to 0.71) | 2.8 (0.53 to 8.82) | 0.53 (0.1 to 1.7) | 3.01 (0.56 to 9.59) | 0.47 (0.36 to 0.58) | <0.001 | 11.96 (5.51 to 27.29) | 149.49 (68.91 to 341.19) | 27.35 (12.18 to 64.19) | 154.41 (68.77 to 362.37) | 0.31 (0.19 to 0.42) | <0.001 |
| Southern Latin America | 0.29 (0.06 to 0.93) | 2.57 (0.49 to 8.14) | 0.52 (0.1 to 1.61) | 2.69 (0.52 to 8.36) | 0.47 (0.24 to 0.7) | <0.001 | 16.19 (7.54 to 36.48) | 141.64 (65.95 to 319.08) | 27.75 (12.71 to 62.08) | 143.81 (65.88 to 321.74) | 0.14 (0.07 to 0.21) | <0.001 |
| South Asia | 4.24 (0.8 to 13.72) | 2.26 (0.42 to 7.3) | 11.64 (2.2 to 35.99) | 2.71 (0.51 to 8.39) | 0.42 (0.29 to 0.56) | <0.001 | 213.16 (93.63 to 512.91) | 113.34 (49.78 to 272.72) | 552.7 (233.77 to 1330.81) | 128.83 (54.49 to 310.2) | 0.33 (0.24 to 0.43) | <0.001 |
| South-East Asia Region | 5.73 (1.08 to 18.4) | 2.48 (0.47 to 7.97) | 15.58 (3.02 to 48.16) | 2.98 (0.58 to 9.21) | 0.43 (0.31 to 0.56) | <0.001 | 294.38 (132.1 to 697.38) | 127.48 (57.21 to 302) | 759.18 (328.3 to 1780.25) | 145.12 (62.76 to 340.31) | 0.33 (0.22 to 0.45) | <0.001 |
| Oceania | 0.03 (0.01 to 0.11) | 3.25 (0.61 to 10.43) | 0.08 (0.01 to 0.25) | 2.97 (0.55 to 9.17) | 0.39 (0.29 to 0.49) | <0.001 | 1.65 (0.72 to 4.08) | 162.36 (70.37 to 401.03) | 4.08 (1.74 to 9.55) | 149.91 (63.86 to 350.71) | -0.25 (-0.4 to -0.09) | 0.002 |
| North Africa and Middle East | 1.83 (0.35 to 5.72) | 3.48 (0.67 to 10.9) | 4.41 (0.86 to 13.87) | 2.92 (0.57 to 9.17) | 0.37 (-0.09 to 0.83) | 0.11 | 93.38 (41.88 to 215.57) | 177.84 (79.77 to 410.54) | 231.53 (105.08 to 532.7) | 153.08 (69.47 to 352.2) | -0.47 (-0.6 to -0.34) | <0.001 |
| High-income North America | 2.66 (0.51 to 8.49) | 3.69 (0.7 to 11.78) | 5.31 (1.02 to 16.58) | 4.53 (0.87 to 14.14) | 0.21 (0.11 to 0.31) | <0.001 | 143.96 (68.65 to 323.93) | 199.79 (95.28 to 449.56) | 285.18 (137.29 to 636.69) | 243.29 (117.13 to 543.17) | 1.02 (0.82 to 1.22) | <0.001 |
| High-income Asia Pacific | 1.72 (0.33 to 5.38) | 3.22 (0.61 to 10.09) | 2.39 (0.47 to 7.33) | 3.6 (0.71 to 11.05) | 0.21 (0.14 to 0.27) | <0.001 | 88.98 (40.69 to 203.88) | 166.91 (76.32 to 382.42) | 125.09 (57.87 to 280.75) | 188.55 (87.23 to 423.17) | 0.39 (0.29 to 0.5) | <0.001 |
| Eastern Sub-Saharan Africa | 0.71 (0.14 to 2.24) | 2.96 (0.57 to 9.34) | 1.76 (0.34 to 5.33) | 2.91 (0.56 to 8.8) | 0.07 (-0.15 to 0.3) | 0.509 | 36.35 (16.24 to 85.62) | 151.74 (67.8 to 357.37) | 87.32 (37.54 to 202.44) | 144 (61.91 to 333.84) | -0.09 (-0.15 to -0.04) | 0.002 |
| Eastern Europe | 2.31 (0.44 to 7.25) | 3.51 (0.66 to 11.01) | 2.63 (0.51 to 8.16) | 3.67 (0.72 to 11.39) | 0.05 (-0.06 to 0.17) | 0.344 | 121.35 (56.24 to 274.87) | 184.21 (85.38 to 417.26) | 135.17 (61.59 to 310.76) | 188.57 (85.92 to 433.51) | 0.34 (-0.04 to 0.72) | 0.075 |
| East Asia | 10.26 (1.96 to 30.76) | 4.03 (0.77 to 12.08) | 21.59 (4.15 to 68.74) | 4.12 (0.79 to 13.13) | 0.03 (-0.13 to 0.18) | 0.734 | 507 (223.05 to 1175.67) | 199.07 (87.58 to 461.63) | 1154.07 (520.56 to 2685.54) | 220.37 (99.4 to 512.81) | 0.38 (0.19 to 0.57) | <0.001 |
| Central Sub-Saharan Africa | 0.28 (0.05 to 0.89) | 3.86 (0.73 to 12.07) | 0.79 (0.15 to 2.36) | 3.83 (0.7 to 11.43) | 0 (-0.15 to 0.15) | 0.998 | 13.93 (5.93 to 33.32) | 188.66 (80.29 to 451.39) | 37.45 (14.98 to 88.96) | 181.26 (72.52 to 430.59) | -0.08 (-0.33 to 0.17) | 0.511 |
| Central Latin America | 0.63 (0.12 to 1.98) | 2.48 (0.47 to 7.83) | 1.88 (0.36 to 5.83) | 2.8 (0.53 to 8.69) | 0 (-0.15 to 0.15) | 0.998 | 33.69 (15.61 to 76.1) | 132.95 (61.61 to 300.3) | 98.68 (44.88 to 224.19) | 146.99 (66.86 to 333.96) | 0.39 (0.27 to 0.51) | <0.001 |
| Central Europe | 1.2 (0.23 to 3.8) | 3.38 (0.64 to 10.66) | 1.36 (0.26 to 4.26) | 3.38 (0.64 to 10.59) | -0.02 (-0.11 to 0.08) | 0.721 | 62.38 (28.32 to 143.49) | 175.11 (79.5 to 402.83) | 70.39 (32.36 to 158.43) | 175.03 (80.46 to 393.93) | 0.44 (0.24 to 0.63) | <0.001 |
| Central Asia | 0.39 (0.07 to 1.22) | 3.18 (0.6 to 9.85) | 0.73 (0.14 to 2.27) | 2.96 (0.58 to 9.18) | -0.03 (-0.22 to 0.15) | 0.706 | 20.72 (9.59 to 47.16) | 167.48 (77.54 to 381.17) | 38.52 (17.64 to 86.63) | 155.78 (71.34 to 350.36) | -0.37 (-0.87 to 0.14) | 0.153 |
| Caribbean | 0.17 (0.03 to 0.53) | 2.53 (0.48 to 7.91) | 0.36 (0.07 to 1.12) | 2.8 (0.54 to 8.6) | -0.09 (-0.16 to -0.02) | 0.012 | 8.87 (4.06 to 20.4) | 131.34 (60.07 to 301.89) | 18.59 (8.39 to 43.23) | 143.04 (64.54 to 332.62) | 0.16 (0.06 to 0.26) | 0.002 |
| Australasia | 0.16 (0.03 to 0.49) | 2.99 (0.58 to 9.32) | 0.32 (0.06 to 1.01) | 3.4 (0.67 to 10.58) | -0.12 (-0.41 to 0.17) | 0.403 | 8.33 (3.79 to 18.94) | 157.1 (71.42 to 356.98) | 16.58 (7.55 to 38.01) | 173.33 (78.97 to 397.41) | 0.61 (0.49 to 0.74) | <0.001 |
| Andean Latin America | 0.14 (0.03 to 0.43) | 2.29 (0.44 to 7.04) | 0.38 (0.07 to 1.2) | 2.4 (0.46 to 7.5) | -0.3 (-0.4 to -0.2) | <0.001 | 7.13 (3.18 to 16.39) | 117.35 (52.4 to 269.7) | 19.52 (8.62 to 45.46) | 122.47 (54.08 to 285.18) | 0.2 (0.14 to 0.25) | <0.001 |

Note: CI, confidence interval; AAPC, average annual percentage change; EOAD, early-onset Alzheimer’s disease and other dementias; DALYs, disability-adjusted life years.

**Supplementary Table S5. Age-standardized prevalence and incidence of EOAD in men aged 40-64 years and their AAPCs from 1990 to 2021 at the regional levels.**

|  | Prevalence | | | | | | Incidence | | | | | |
| --- | --- | --- | --- | --- | --- | --- | --- | --- | --- | --- | --- | --- |
| Region | No of people with EOAD in 1990 (000s) | Age standardised rate in 1990 (per 100 000) | No of people with EOAD in 2021 (000s) | Age standardised rate in 2021 (per 100 000) | AAPC (95% CI) | *p* value | No of people with EOAD in 1990 (000s) | Age standardised rate in 1990 (per 100 000) | No of people with EOAD in 2021 (000s) | Age standardised rate in 2021 (per 100 000) | AAPC (95% CI) | *p* value |
| Western Sub-Saharan Africa | 31.94 (23.16 to 42.41) | 217.55 (157.71 to 288.84) | 62.78 (44.94 to 84.13) | 187.5 (134.21 to 251.26) | -0.51 (-0.57 to -0.46) | <0.001 | 5.36 (3.31 to 7.88) | 36.53 (22.53 to 53.7) | 10.72 (6.49 to 15.86) | 32.01 (19.37 to 47.38) | -0.45 (-0.49 to -0.41) | <0.001 |
| Western Europe | 154.23 (113.82 to 202.53) | 274.99 (202.95 to 361.11) | 206.55 (148.83 to 272.51) | 278.73 (200.84 to 367.74) | -0.02 (-0.1 to 0.06) | 0.621 | 26.5 (16.59 to 38.52) | 47.26 (29.57 to 68.67) | 34.78 (21.58 to 51.24) | 46.94 (29.12 to 69.14) | -0.07 (-0.14 to -0.01) | 0.036 |
| Tropical Latin America | 50.33 (37.5 to 65.79) | 375.32 (279.63 to 490.57) | 132.46 (99.1 to 173.63) | 414.36 (310 to 543.16) | 0.49 (0.39 to 0.59) | <0.001 | 8.89 (5.78 to 12.62) | 66.31 (43.09 to 94.14) | 23.87 (15.46 to 34.18) | 74.68 (48.35 to 106.93) | 0.56 (0.46 to 0.66) | <0.001 |
| Southern Sub-Saharan Africa | 10.42 (7.62 to 13.87) | 275.7 (201.6 to 367.03) | 22.23 (16.17 to 29.83) | 265.94 (193.49 to 356.91) | 0.12 (0.03 to 0.22) | 0.016 | 1.77 (1.11 to 2.58) | 46.96 (29.47 to 68.26) | 3.76 (2.35 to 5.5) | 44.96 (28.06 to 65.86) | 0.04 (-0.03 to 0.12) | 0.265 |
| Southern Latin America | 15.07 (10.99 to 19.82) | 272.86 (198.91 to 358.82) | 25.3 (18.48 to 33.42) | 271.51 (198.26 to 358.61) | 0.06 (0.01 to 0.12) | 0.028 | 2.56 (1.59 to 3.77) | 46.33 (28.8 to 68.2) | 4.27 (2.65 to 6.29) | 45.83 (28.44 to 67.45) | 0.03 (-0.02 to 0.08) | 0.185 |
| Southeast Asia | 132.61 (99.42 to 173.76) | 343.6 (257.59 to 450.21) | 332.65 (247.89 to 436.45) | 342.26 (255.05 to 449.06) | 0.08 (-0.12 to 0.27) | 0.415 | 23.67 (15.19 to 33.73) | 61.33 (39.35 to 87.4) | 58.88 (38.01 to 83.68) | 60.58 (39.11 to 86.1) | 0.05 (-0.09 to 0.2) | 0.452 |
| South-East Asia Region | 282.51 (205.57 to 372.39) | 235.44 (171.32 to 310.34) | 638.69 (462.89 to 844.77) | 244.42 (177.15 to 323.29) | 0.07 (-0.05 to 0.2) | 0.252 | 50.16 (31.83 to 72.65) | 41.8 (26.53 to 60.55) | 111.95 (70.9 to 162.83) | 42.84 (27.13 to 62.32) | 0.04 (-0.05 to 0.14) | 0.335 |
| Oceania | 1.54 (1.13 to 2.02) | 289.76 (213.18 to 380.66) | 3.88 (2.85 to 5.16) | 276.8 (203.1 to 367.61) | -0.12 (-0.22 to -0.02) | 0.016 | 0.28 (0.18 to 0.4) | 52.95 (33.31 to 76.11) | 0.71 (0.45 to 1.03) | 50.44 (32.02 to 73.43) | -0.14 (-0.21 to -0.06) | 0.001 |
| North Africa and Middle East | 92.18 (68.76 to 119.92) | 341.28 (254.55 to 443.97) | 236.98 (177.08 to 310.01) | 297.73 (222.46 to 389.47) | -0.38 (-0.49 to -0.26) | <0.001 | 17.35 (11.24 to 24.58) | 64.21 (41.61 to 91) | 45.05 (29.08 to 64.38) | 56.6 (36.54 to 80.88) | -0.36 (-0.45 to -0.26) | <0.001 |
| High-income North America | 140.96 (105.34 to 182.53) | 402.35 (300.66 to 520.99) | 279.28 (212.34 to 357.85) | 486.31 (369.76 to 623.13) | 0.89 (0.75 to 1.04) | <0.001 | 23.44 (15.11 to 33.51) | 66.89 (43.12 to 95.64) | 44.62 (29.25 to 62.99) | 77.69 (50.93 to 109.68) | 0.69 (0.57 to 0.81) | <0.001 |
| High-income Asia Pacific | 75.17 (55.48 to 99.18) | 285.37 (210.64 to 376.54) | 115.55 (86.22 to 150.88) | 345.19 (257.57 to 450.73) | 0.6 (0.49 to 0.71) | <0.001 | 12.66 (8.07 to 18.3) | 48.07 (30.63 to 69.46) | 20.27 (13.1 to 28.99) | 60.57 (39.14 to 86.61) | 0.79 (0.69 to 0.88) | <0.001 |
| Eastern Sub-Saharan Africa | 33.67 (24.88 to 44.33) | 283.81 (209.73 to 373.62) | 72.72 (53.23 to 96.68) | 244.13 (178.69 to 324.55) | -0.45 (-0.49 to -0.4) | <0.001 | 5.81 (3.69 to 8.36) | 48.96 (31.09 to 70.45) | 12.74 (7.92 to 18.53) | 42.78 (26.6 to 62.19) | -0.4 (-0.43 to -0.37) | <0.001 |
| Eastern Europe | 102.32 (75.58 to 135.02) | 341.21 (252.04 to 450.29) | 114.1 (84.1 to 149.42) | 345.05 (254.31 to 451.87) | 0.28 (-0.03 to 0.6) | 0.075 | 16.96 (10.74 to 24.33) | 56.55 (35.81 to 81.15) | 18.91 (12.09 to 27.17) | 57.19 (36.57 to 82.17) | 0.22 (-0.01 to 0.46) | 0.061 |
| East Asia | 460.4 (340.71 to 606.68) | 345.4 (255.6 to 455.14) | 1142.12 (847.03 to 1494.1) | 431.75 (320.2 to 564.8) | 0.65 (0.47 to 0.84) | <0.001 | 79.36 (51.09 to 113.48) | 59.54 (38.33 to 85.13) | 213.27 (139.73 to 300.54) | 80.62 (52.82 to 113.61) | 0.79 (0.66 to 0.93) | <0.001 |
| Central Sub-Saharan Africa | 10.81 (7.97 to 14.28) | 312.71 (230.39 to 412.92) | 28.05 (20.62 to 37.08) | 274.85 (202.13 to 363.39) | -0.34 (-0.53 to -0.15) | 0.001 | 1.89 (1.2 to 2.75) | 54.59 (34.71 to 79.42) | 4.98 (3.07 to 7.22) | 48.83 (30.11 to 70.78) | -0.3 (-0.45 to -0.16) | <0.001 |
| Central Latin America | 33.63 (24.81 to 44.26) | 270.97 (199.94 to 356.62) | 91.84 (67.81 to 120.73) | 288.76 (213.22 to 379.62) | 0.28 (0.18 to 0.38) | <0.001 | 5.99 (3.77 to 8.65) | 48.28 (30.37 to 69.69) | 15.95 (10.1 to 23.17) | 50.16 (31.76 to 72.85) | 0.19 (0.12 to 0.27) | <0.001 |
| Central Europe | 56.11 (41.72 to 72.95) | 325.66 (242.12 to 423.39) | 66.1 (49.25 to 85.8) | 330.88 (246.54 to 429.45) | 0.44 (0.27 to 0.6) | <0.001 | 9.64 (6.16 to 13.89) | 55.97 (35.78 to 80.62) | 11.3 (7.24 to 16.25) | 56.55 (36.25 to 81.33) | 0.34 (0.21 to 0.47) | <0.001 |
| Central Asia | 18.65 (13.78 to 24.49) | 315.6 (233.11 to 414.4) | 34.9 (25.71 to 45.89) | 294.59 (217.06 to 387.37) | -0.27 (-0.71 to 0.18) | 0.231 | 3.19 (2.03 to 4.6) | 54.05 (34.27 to 77.86) | 6.05 (3.85 to 8.77) | 51.08 (32.46 to 74.01) | -0.21 (-0.55 to 0.14) | 0.235 |
| Caribbean | 8.61 (6.31 to 11.4) | 259.47 (189.96 to 343.4) | 17.26 (12.72 to 22.79) | 272.08 (200.48 to 359.13) | 0.03 (-0.07 to 0.13) | 0.558 | 1.52 (0.96 to 2.21) | 45.69 (28.88 to 66.52) | 2.99 (1.86 to 4.35) | 47.14 (29.33 to 68.62) | -0.01 (-0.1 to 0.07) | 0.723 |
| Australasia | 7.68 (5.59 to 10.2) | 286.46 (208.53 to 380.46) | 14.6 (10.73 to 19.08) | 311.56 (228.91 to 407.26) | 0.49 (0.4 to 0.58) | <0.001 | 1.29 (0.8 to 1.9) | 48.06 (29.74 to 71.04) | 2.4 (1.49 to 3.5) | 51.19 (31.73 to 74.65) | 0.38 (0.31 to 0.46) | <0.001 |
| Andean Latin America | 6.61 (4.81 to 8.73) | 219.71 (159.96 to 289.98) | 17.45 (12.64 to 23.23) | 224.07 (162.32 to 298.21) | 0.13 (0.09 to 0.17) | <0.001 | 1.11 (0.68 to 1.65) | 37.04 (22.63 to 54.67) | 2.91 (1.76 to 4.28) | 37.34 (22.64 to 54.9) | 0.08 (0.05 to 0.12) | <0.001 |

Note: CI, confidence interval; AAPC, average annual percentage change; EOAD, early-onset Alzheimer’s disease and other dementias.

**Supplementary Table S6. Age-standardized mortality and DALYs of EOAD in men aged 40-64 years and their AAPCs from 1990 to 2021 at the regional levels.**

|  | Mortality | | | | | | DALYs | | | | | |
| --- | --- | --- | --- | --- | --- | --- | --- | --- | --- | --- | --- | --- |
| Region | No of people with EOAD in 1990 (000s) | Age standardised rate in 1990 (per 100 000) | No of people with EOAD in 2021 (000s) | Age standardised rate in 2021 (per 100 000) | AAPC (95% CI) | *p* value | No of people with EOAD in 1990 (000s) | Age standardised rate in 1990 (per 100 000) | No of people with EOAD in 2021 (000s) | Age standardised rate in 2021 (per 100 000) | AAPC (95% CI) | *p* value |
| Western Sub-Saharan Africa | 0.32 (0.06 to 1.03) | 2.2 (0.42 to 6.99) | 0.69 (0.13 to 2.19) | 2.05 (0.39 to 6.55) | -0.25 (-0.39 to -0.1) | 0.001 | 15.98 (6.73 to 38.57) | 108.86 (45.85 to 262.7) | 33.38 (13.77 to 81.41) | 99.69 (41.14 to 243.14) | -0.3 (-0.41 to -0.19) | <0.001 |
| Western Europe | 1.44 (0.27 to 4.52) | 2.57 (0.49 to 8.06) | 1.98 (0.38 to 6.1) | 2.68 (0.52 to 8.23) | 0.07 (-0.01 to 0.15) | 0.094 | 72.88 (32.15 to 170.68) | 129.95 (57.33 to 304.34) | 99.37 (43.62 to 228.93) | 134.09 (58.86 to 308.93) | 0.04 (-0.04 to 0.12) | 0.302 |
| Tropical Latin America | 0.49 (0.09 to 1.54) | 3.64 (0.7 to 11.48) | 1.32 (0.26 to 4.14) | 4.13 (0.8 to 12.94) | 0.5 (0.36 to 0.65) | <0.001 | 24.22 (10.49 to 57.79) | 180.63 (78.2 to 430.98) | 64.78 (27.92 to 154.7) | 202.63 (87.32 to 483.95) | 0.5 (0.38 to 0.62) | <0.001 |
| Southern Sub-Saharan Africa | 0.09 (0.02 to 0.27) | 2.28 (0.43 to 7.1) | 0.21 (0.04 to 0.66) | 2.46 (0.45 to 7.89) | 0.48 (0.41 to 0.56) | <0.001 | 4.59 (2.02 to 10.52) | 121.52 (53.5 to 278.56) | 10.48 (4.47 to 25.16) | 125.38 (53.53 to 301.06) | 0.33 (0.25 to 0.41) | <0.001 |
| Southern Latin America | 0.12 (0.02 to 0.38) | 2.16 (0.41 to 6.97) | 0.22 (0.04 to 0.68) | 2.31 (0.44 to 7.3) | 0.3 (0.22 to 0.38) | <0.001 | 6.48 (2.9 to 14.76) | 117.36 (52.45 to 267.35) | 11.34 (5.03 to 26.15) | 121.68 (53.95 to 280.56) | 0.19 (0.13 to 0.26) | <0.001 |
| Southeast Asia | 1.11 (0.21 to 3.56) | 2.87 (0.55 to 9.23) | 3.18 (0.6 to 9.88) | 3.28 (0.61 to 10.17) | 0.4 (0.15 to 0.66) | 0.003 | 58.95 (26.25 to 136.95) | 152.73 (68 to 354.83) | 160.9 (68.4 to 379.6) | 165.55 (70.37 to 390.56) | 0.3 (0.08 to 0.52) | 0.01 |
| South-East Asia Region | 2.48 (0.47 to 8) | 2.06 (0.39 to 6.66) | 6.37 (1.21 to 19.86) | 2.44 (0.46 to 7.6) | 0.49 (0.35 to 0.63) | <0.001 | 128.64 (56.36 to 306.62) | 107.21 (46.97 to 255.53) | 315.56 (133.59 to 741.69) | 120.77 (51.13 to 283.84) | 0.35 (0.22 to 0.48) | <0.001 |
| Oceania | 0.02 (0 to 0.05) | 2.88 (0.54 to 9.15) | 0.04 (0.01 to 0.12) | 2.75 (0.5 to 8.7) | -0.15 (-0.34 to 0.03) | 0.107 | 0.76 (0.32 to 1.87) | 143.56 (60.52 to 351.92) | 1.93 (0.8 to 4.58) | 137.57 (57.04 to 326.64) | -0.13 (-0.28 to 0.02) | 0.082 |
| North Africa and Middle East | 0.85 (0.16 to 2.7) | 3.16 (0.61 to 10.01) | 2.11 (0.41 to 6.67) | 2.65 (0.51 to 8.38) | -0.55 (-0.71 to -0.39) | <0.001 | 43.42 (18.9 to 100.36) | 160.74 (69.96 to 371.53) | 109.2 (48.55 to 254.94) | 137.2 (61 to 320.29) | -0.47 (-0.6 to -0.34) | <0.001 |
| High-income North America | 1.18 (0.22 to 3.78) | 3.37 (0.64 to 10.78) | 2.41 (0.46 to 7.59) | 4.19 (0.8 to 13.21) | 1.16 (0.89 to 1.42) | <0.001 | 62.01 (28.36 to 142.8) | 176.99 (80.94 to 407.59) | 124.3 (56.85 to 284.82) | 216.44 (99 to 495.97) | 1.02 (0.82 to 1.22) | <0.001 |
| High-income Asia Pacific | 0.71 (0.13 to 2.26) | 2.71 (0.51 to 8.59) | 1.07 (0.21 to 3.3) | 3.19 (0.62 to 9.85) | 0.54 (0.43 to 0.66) | <0.001 | 36.14 (15.59 to 85.56) | 137.19 (59.21 to 324.84) | 54.36 (24.17 to 126.38) | 162.39 (72.19 to 377.55) | 0.54 (0.43 to 0.65) | <0.001 |
| Eastern Sub-Saharan Africa | 0.32 (0.06 to 1.02) | 2.71 (0.5 to 8.6) | 0.76 (0.14 to 2.32) | 2.55 (0.48 to 7.79) | -0.14 (-0.2 to -0.09) | <0.001 | 16.2 (6.94 to 38.97) | 136.55 (58.51 to 328.42) | 37.35 (15.63 to 88.21) | 125.38 (52.47 to 296.12) | -0.22 (-0.28 to -0.17) | <0.001 |
| Eastern Europe | 0.87 (0.16 to 2.78) | 2.9 (0.55 to 9.27) | 1.03 (0.2 to 3.25) | 3.11 (0.59 to 9.83) | 0.38 (-0.09 to 0.86) | 0.11 | 45.27 (20.22 to 104.89) | 150.96 (67.42 to 349.79) | 52.22 (22.64 to 121.55) | 157.91 (68.47 to 367.57) | 0.37 (-0.03 to 0.76) | 0.066 |
| East Asia | 5.01 (0.94 to 15.17) | 3.76 (0.7 to 11.38) | 10.28 (1.88 to 33.56) | 3.89 (0.71 to 12.69) | 0.18 (-0.02 to 0.39) | 0.078 | 241.57 (100.43 to 578.09) | 181.23 (75.35 to 433.69) | 533.28 (234.21 to 1281.88) | 201.59 (88.54 to 484.58) | 0.36 (0.17 to 0.54) | 0.001 |
| Central Sub-Saharan Africa | 0.11 (0.02 to 0.33) | 3.05 (0.57 to 9.65) | 0.3 (0.06 to 0.94) | 2.97 (0.56 to 9.24) | -0.01 (-0.37 to 0.35) | 0.966 | 5.25 (2.19 to 12.56) | 151.81 (63.31 to 363.09) | 14.69 (5.73 to 36) | 143.93 (56.17 to 352.83) | -0.08 (-0.37 to 0.22) | 0.599 |
| Central Latin America | 0.29 (0.06 to 0.93) | 2.37 (0.45 to 7.51) | 0.84 (0.16 to 2.67) | 2.65 (0.51 to 8.4) | 0.4 (0.28 to 0.52) | <0.001 | 15.29 (6.75 to 35.53) | 123.21 (54.37 to 286.29) | 42.98 (18.79 to 101.16) | 135.13 (59.09 to 318.09) | 0.35 (0.24 to 0.45) | <0.001 |
| Central Europe | 0.5 (0.09 to 1.59) | 2.92 (0.55 to 9.25) | 0.59 (0.11 to 1.86) | 2.97 (0.56 to 9.29) | 0.53 (0.28 to 0.77) | <0.001 | 25.68 (11.35 to 60.18) | 149.06 (65.89 to 349.3) | 30.26 (13.57 to 69.32) | 151.46 (67.92 to 346.98) | 0.49 (0.29 to 0.69) | <0.001 |
| Central Asia | 0.16 (0.03 to 0.52) | 2.77 (0.52 to 8.81) | 0.31 (0.06 to 0.98) | 2.63 (0.51 to 8.3) | -0.49 (-1.07 to 0.1) | 0.101 | 8.51 (3.78 to 19.66) | 143.99 (63.98 to 332.62) | 16.11 (7.09 to 37.03) | 135.98 (59.88 to 312.59) | -0.35 (-0.86 to 0.16) | 0.173 |
| Caribbean | 0.08 (0.02 to 0.25) | 2.47 (0.47 to 7.65) | 0.17 (0.03 to 0.53) | 2.71 (0.51 to 8.38) | 0.17 (0.07 to 0.28) | 0.002 | 4.13 (1.81 to 9.62) | 124.5 (54.54 to 289.69) | 8.51 (3.62 to 19.99) | 134.05 (57.08 to 315) | 0.12 (0.02 to 0.22) | 0.025 |
| Australasia | 0.07 (0.01 to 0.22) | 2.65 (0.51 to 8.14) | 0.14 (0.03 to 0.45) | 3.06 (0.6 to 9.62) | 0.8 (0.66 to 0.93) | <0.001 | 3.59 (1.55 to 8.26) | 133.9 (57.82 to 308.07) | 7.09 (3.02 to 16.72) | 151.32 (64.45 to 356.83) | 0.66 (0.56 to 0.77) | <0.001 |
| Andean Latin America | 0.07 (0.01 to 0.2) | 2.18 (0.41 to 6.62) | 0.17 (0.03 to 0.54) | 2.25 (0.43 to 6.97) | 0.19 (0.12 to 0.25) | <0.001 | 3.26 (1.39 to 7.58) | 108.42 (46.09 to 251.72) | 8.69 (3.68 to 20.45) | 111.57 (47.26 to 262.53) | 0.16 (0.11 to 0.22) | <0.001 |

Note: CI, confidence interval; AAPC, average annual percentage change; EOAD, early-onset Alzheimer’s disease and other dementias; DALYs, disability-adjusted life years.

**Supplementary Table S7. Age-standardized prevalence and incidence of EOAD in women aged 40-64 years and their AAPCs from 1990 to 2021 at the regional levels.**

|  | Prevalence | | | | | | Incidence | | | | | |
| --- | --- | --- | --- | --- | --- | --- | --- | --- | --- | --- | --- | --- |
| Region | No of people with EOAD in 1990 (000s) | Age standardised rate in 1990 (per 100 000) | No of people with EOAD in 2021 (000s) | Age standardised rate in 2021 (per 100 000) | AAPC (95% CI) | *p* value | No of people with EOAD in 1990 (000s) | Age standardised rate in 1990 (per 100 000) | No of people with EOAD in 2021 (000s) | Age standardised rate in 2021 (per 100 000) | AAPC (95% CI) | *p* value |
| Western Sub-Saharan Africa | 31.35 (23.06 to 41.5) | 254.44 (187.16 to 336.78) | 86.4 (63.25 to 114.73) | 235.41 (172.35 to 312.6) | -0.3 (-0.39 to -0.2) | <0.001 | 5.66 (3.57 to 8.19) | 45.91 (28.96 to 66.5) | 15.39 (9.62 to 22.32) | 41.93 (26.22 to 60.81) | -0.33 (-0.4 to -0.25) | <0.001 |
| Western Europe | 218.46 (165.04 to 282.5) | 380.26 (287.28 to 491.74) | 279.98 (207.07 to 365.06) | 371.25 (274.58 to 484.07) | -0.09 (-0.18 to -0.01) | 0.031 | 36 (22.86 to 52.02) | 62.66 (39.79 to 90.54) | 45.21 (28.36 to 66.31) | 59.95 (37.61 to 87.93) | -0.13 (-0.2 to -0.06) | 0.001 |
| Tropical Latin America | 61.89 (46.35 to 80.46) | 438.86 (328.63 to 570.46) | 163.36 (123.02 to 212.71) | 469.3 (353.4 to 611.09) | 0.34 (0.26 to 0.42) | <0.001 | 11.2 (7.31 to 15.79) | 79.41 (51.82 to 111.99) | 29.63 (19.24 to 42.08) | 85.11 (55.28 to 120.88) | 0.33 (0.24 to 0.41) | <0.001 |
| Southern Sub-Saharan Africa | 15.51 (11.5 to 20.36) | 367.65 (272.45 to 482.4) | 34.33 (25.45 to 45.35) | 367.01 (272.09 to 484.75) | 0.15 (0 to 0.31) | 0.05 | 2.79 (1.79 to 4.02) | 66.06 (42.42 to 95.16) | 6.06 (3.88 to 8.74) | 64.78 (41.43 to 93.44) | 0.09 (-0.04 to 0.21) | 0.178 |
| Southern Latin America | 21.56 (16.15 to 27.88) | 364.8 (273.15 to 471.63) | 35.09 (26.17 to 45.94) | 351.84 (262.39 to 460.59) | -0.02 (-0.08 to 0.05) | 0.581 | 3.61 (2.27 to 5.19) | 61.01 (38.45 to 87.75) | 5.82 (3.64 to 8.46) | 58.39 (36.47 to 84.85) | -0.06 (-0.12 to 0) | 0.035 |
| South Asia | 223.16 (162.91 to 294.54) | 252.19 (184.11 to 332.85) | 549.99 (402.08 to 729.2) | 258.06 (188.66 to 342.14) | -0.05 (-0.12 to 0.02) | 0.131 | 39.64 (24.99 to 57.34) | 44.8 (28.24 to 64.8) | 94.74 (59.23 to 137.81) | 44.45 (27.79 to 64.66) | -0.11 (-0.16 to -0.05) | <0.001 |
| South-East Asia Region | 325.38 (243.02 to 426.08) | 293.33 (219.09 to 384.11) | 793.03 (589.65 to 1039.11) | 302.88 (225.2 to 396.86) | 0.01 (-0.08 to 0.1) | 0.839 | 58 (37.09 to 82.73) | 52.29 (33.44 to 74.59) | 137.89 (88.04 to 197.28) | 52.66 (33.62 to 75.35) | -0.03 (-0.1 to 0.04) | 0.411 |
| Oceania | 1.65 (1.23 to 2.18) | 340.07 (253.58 to 447.35) | 4.12 (3.07 to 5.44) | 312.07 (232.43 to 411.74) | -0.26 (-0.36 to -0.15) | <0.001 | 0.31 (0.2 to 0.44) | 63.26 (40.47 to 90.47) | 0.77 (0.49 to 1.11) | 58.42 (37.38 to 83.97) | -0.25 (-0.34 to -0.16) | <0.001 |
| North Africa and Middle East | 99 (74.61 to 128.76) | 388.3 (292.63 to 505.02) | 257.04 (193.47 to 335.88) | 358.74 (270.01 to 468.78) | -0.22 (-0.33 to -0.1) | 0.001 | 18.73 (12.08 to 26.77) | 73.46 (47.36 to 104.99) | 48.82 (31.41 to 69.64) | 68.14 (43.84 to 97.19) | -0.21 (-0.31 to -0.12) | <0.001 |
| High-income North America | 183.63 (139.84 to 236) | 496.01 (377.74 to 637.48) | 366.63 (280.57 to 468.09) | 613.18 (469.26 to 782.88) | 1 (0.87 to 1.13) | <0.001 | 29.91 (19.59 to 42.67) | 80.78 (52.92 to 115.26) | 59.55 (39.43 to 83.91) | 99.6 (65.95 to 140.34) | 0.92 (0.83 to 1.01) | <0.001 |
| High-income Asia Pacific | 107.11 (80.48 to 140.02) | 397.13 (298.4 to 519.13) | 148.09 (111.4 to 192.14) | 450.55 (338.91 to 584.55) | 0.39 (0.29 to 0.48) | <0.001 | 18.25 (11.81 to 25.97) | 67.67 (43.77 to 96.3) | 25.9 (17.05 to 36.72) | 78.81 (51.87 to 111.73) | 0.5 (0.42 to 0.57) | <0.001 |
| Eastern Sub-Saharan Africa | 40.74 (30.4 to 53.54) | 336.87 (251.35 to 442.75) | 93.82 (69.94 to 123.31) | 304.1 (226.69 to 399.67) | -0.26 (-0.29 to -0.22) | <0.001 | 7.56 (4.89 to 10.86) | 62.52 (40.47 to 89.79) | 17.49 (11.17 to 25.13) | 56.71 (36.21 to 81.44) | -0.25 (-0.28 to -0.21) | <0.001 |
| Eastern Europe | 162.27 (121.88 to 211.53) | 452.15 (339.62 to 589.41) | 172.96 (130.22 to 225.36) | 447.9 (337.21 to 583.6) | 0.24 (-0.07 to 0.54) | 0.121 | 27.24 (17.67 to 38.74) | 75.9 (49.23 to 107.95) | 28.99 (18.83 to 41.07) | 75.07 (48.76 to 106.35) | 0.19 (-0.05 to 0.43) | 0.112 |
| East Asia | 506.32 (379.26 to 661.51) | 417.12 (312.45 to 544.97) | 1324.79 (997.79 to 1738.18) | 511.2 (385.02 to 670.72) | 0.66 (0.46 to 0.86) | <0.001 | 90.61 (58.79 to 128.65) | 74.65 (48.43 to 105.99) | 249.99 (162.18 to 354.75) | 96.46 (62.58 to 136.89) | 0.72 (0.57 to 0.87) | <0.001 |
| Central Sub-Saharan Africa | 15.83 (11.76 to 20.77) | 403.52 (299.8 to 529.39) | 37.87 (28.09 to 49.68) | 362.2 (268.63 to 475.14) | -0.36 (-0.5 to -0.22) | <0.001 | 3.06 (1.95 to 4.42) | 78.06 (49.65 to 112.71) | 7.34 (4.68 to 10.52) | 70.16 (44.8 to 100.6) | -0.36 (-0.48 to -0.24) | <0.001 |
| Central Latin America | 40.05 (29.85 to 52.6) | 309.76 (230.88 to 406.8) | 117.89 (87.78 to 154.67) | 333.7 (248.48 to 437.83) | 0.33 (0.22 to 0.44) | <0.001 | 7.14 (4.55 to 10.32) | 55.25 (35.21 to 79.77) | 20.27 (12.94 to 29.2) | 57.37 (36.63 to 82.65) | 0.23 (0.14 to 0.32) | <0.001 |
| Central Europe | 76.39 (57.76 to 98.75) | 415.35 (314.07 to 536.97) | 83.97 (63.76 to 108.7) | 414.89 (315 to 537.05) | 0.39 (0.23 to 0.56) | <0.001 | 13.2 (8.57 to 18.84) | 71.76 (46.58 to 102.43) | 14.45 (9.4 to 20.53) | 71.39 (46.46 to 101.44) | 0.3 (0.17 to 0.43) | <0.001 |
| Central Asia | 25.69 (19.32 to 33.36) | 397.48 (298.94 to 516.14) | 47.11 (35.38 to 61.58) | 365.78 (274.73 to 478.16) | -0.27 (-0.73 to 0.18) | 0.227 | 4.42 (2.85 to 6.32) | 68.39 (44.09 to 97.7) | 8.2 (5.27 to 11.79) | 63.65 (40.94 to 91.53) | -0.22 (-0.58 to 0.14) | 0.226 |
| Caribbean | 9.85 (7.33 to 12.95) | 286.53 (213.37 to 376.86) | 20.56 (15.35 to 26.95) | 309.19 (230.82 to 405.16) | 0.12 (0.03 to 0.21) | 0.012 | 1.73 (1.1 to 2.52) | 50.43 (32.04 to 73.37) | 3.58 (2.26 to 5.18) | 53.82 (33.99 to 77.96) | 0.06 (-0.01 to 0.14) | 0.096 |
| Australasia | 10.27 (7.61 to 13.45) | 391.27 (289.83 to 512.48) | 19.65 (14.58 to 25.87) | 402.77 (298.95 to 530.46) | 0.38 (0.25 to 0.51) | <0.001 | 1.76 (1.1 to 2.55) | 67.04 (41.96 to 97.05) | 3.32 (2.05 to 4.87) | 67.96 (42 to 99.8) | 0.29 (0.17 to 0.41) | <0.001 |
| Andean Latin America | 7.86 (5.79 to 10.37) | 256.17 (188.65 to 337.98) | 21.84 (16.12 to 28.8) | 267.96 (197.81 to 353.31) | 0.2 (0.15 to 0.26) | <0.001 | 1.35 (0.84 to 1.97) | 43.94 (27.35 to 64.21) | 3.69 (2.29 to 5.36) | 45.24 (28.07 to 65.76) | 0.15 (0.11 to 0.19) | <0.001 |

Note: CI, confidence interval; AAPC, average annual percentage change; EOAD, early-onset Alzheimer’s disease and other dementias.

**Supplementary Table S8. Age-standardized mortality and DAYLs of EOAD in women aged 40-64 years and their AAPCs from 1990 to 2021 at the regional levels.**

|  | Mortality | | | | | | DALYs | | | | | |
| --- | --- | --- | --- | --- | --- | --- | --- | --- | --- | --- | --- | --- |
| Region | No of people with EOAD in 1990 (000s) | Age standardised rate in 1990 (per 100 000) | No of people with EOAD in 2021 (000s) | Age standardised rate in 2021 (per 100 000) | AAPC (95% CI) | *p* value | No of people with EOAD in 1990 (000s) | Age standardised rate in 1990 (per 100 000) | No of people with EOAD in 2021 (000s) | Age standardised rate in 2021 (per 100 000) | AAPC (95% CI) | *p* value |
| Western Sub-Saharan Africa | 0.31 (0.06 to 0.97) | 2.51 (0.49 to 7.85) | 0.95 (0.18 to 2.95) | 2.59 (0.48 to 8.03) | 0.08 (-0.13 to 0.29) | 0.446 | 15.84 (6.98 to 37.08) | 128.53 (56.63 to 300.95) | 47.02 (19.85 to 111.14) | 128.11 (54.08 to 302.82) | -0.03 (-0.2 to 0.13) | 0.68 |
| Western Europe | 2.02 (0.39 to 6.22) | 3.52 (0.68 to 10.83) | 2.58 (0.51 to 7.9) | 3.42 (0.68 to 10.47) | -0.15 (-0.25 to -0.05) | 0.005 | 106 (49.03 to 238.11) | 184.5 (85.34 to 414.46) | 135.53 (63.01 to 302.49) | 179.71 (83.55 to 401.09) | -0.12 (-0.21 to -0.03) | 0.011 |
| Tropical Latin America | 0.64 (0.13 to 2) | 4.57 (0.9 to 14.2) | 1.81 (0.36 to 5.57) | 5.2 (1.03 to 16.01) | 0.49 (0.33 to 0.65) | <0.001 | 32.06 (14.31 to 74.34) | 227.3 (101.5 to 527.13) | 87.89 (38.68 to 205.94) | 252.5 (111.13 to 591.62) | 0.44 (0.32 to 0.55) | <0.001 |
| Southern Sub-Saharan Africa | 0.14 (0.03 to 0.43) | 3.26 (0.62 to 10.15) | 0.33 (0.06 to 1.02) | 3.5 (0.65 to 10.93) | 0.4 (0.24 to 0.56) | <0.001 | 7.36 (3.41 to 16.68) | 174.53 (80.78 to 395.21) | 16.87 (7.58 to 39.47) | 180.36 (81.05 to 421.93) | 0.28 (0.13 to 0.44) | 0.001 |
| Southern Latin America | 0.17 (0.03 to 0.54) | 2.96 (0.57 to 9.06) | 0.3 (0.06 to 0.93) | 3.05 (0.59 to 9.36) | 0.2 (0.11 to 0.3) | <0.001 | 9.71 (4.47 to 21.98) | 164.33 (75.7 to 371.79) | 16.41 (7.52 to 36.71) | 164.49 (75.4 to 368.06) | 0.1 (0.02 to 0.18) | 0.011 |
| South Asia | 2.37 (0.45 to 7.52) | 2.68 (0.5 to 8.5) | 6.98 (1.33 to 21.51) | 3.27 (0.62 to 10.09) | 0.51 (0.41 to 0.61) | <0.001 | 117.54 (50.77 to 284.52) | 132.83 (57.38 to 321.53) | 324.58 (135.02 to 793.29) | 152.29 (63.35 to 372.21) | 0.33 (0.24 to 0.41) | <0.001 |
| South-East Asia Region | 3.25 (0.62 to 10.34) | 2.93 (0.56 to 9.32) | 9.22 (1.8 to 28.53) | 3.52 (0.69 to 10.9) | 0.42 (0.29 to 0.55) | <0.001 | 165.74 (73.03 to 392.94) | 149.42 (65.84 to 354.24) | 443.62 (191.22 to 1054.94) | 169.43 (73.03 to 402.91) | 0.28 (0.17 to 0.39) | <0.001 |
| Oceania | 0.02 (0 to 0.06) | 3.65 (0.68 to 11.7) | 0.04 (0.01 to 0.13) | 3.19 (0.59 to 9.87) | -0.43 (-0.62 to -0.24) | <0.001 | 0.89 (0.38 to 2.17) | 182.88 (78.18 to 445.65) | 2.15 (0.91 to 5) | 163.02 (69.12 to 378.66) | -0.35 (-0.5 to -0.2) | <0.001 |
| North Africa and Middle East | 0.97 (0.19 to 3) | 3.82 (0.74 to 11.78) | 2.3 (0.45 to 7.22) | 3.21 (0.63 to 10.08) | -0.62 (-0.77 to -0.46) | <0.001 | 49.96 (22.32 to 114.4) | 195.96 (87.56 to 448.69) | 122.32 (55.31 to 280.45) | 170.72 (77.19 to 391.41) | -0.46 (-0.59 to -0.32) | <0.001 |
| High-income North America | 1.48 (0.28 to 4.64) | 4 (0.77 to 12.55) | 2.9 (0.56 to 8.95) | 4.85 (0.94 to 14.97) | 1.08 (0.81 to 1.35) | <0.001 | 81.95 (39.41 to 179.87) | 221.37 (106.44 to 485.86) | 160.89 (78.23 to 350.98) | 269.08 (130.85 to 587) | 1.02 (0.83 to 1.22) | <0.001 |
| High-income Asia Pacific | 1 (0.19 to 3.11) | 3.72 (0.72 to 11.54) | 1.32 (0.26 to 3.98) | 4.02 (0.8 to 12.1) | 0.27 (0.15 to 0.4) | <0.001 | 52.85 (24.28 to 119.64) | 195.93 (90.03 to 443.58) | 70.73 (33.2 to 155.98) | 215.19 (101.02 to 474.55) | 0.3 (0.19 to 0.41) | <0.001 |
| Eastern Sub-Saharan Africa | 0.39 (0.08 to 1.22) | 3.21 (0.64 to 10.07) | 1 (0.19 to 3.03) | 3.25 (0.62 to 9.81) | 0.13 (0.06 to 0.2) | 0.001 | 20.15 (9.11 to 46.69) | 166.64 (75.33 to 386.08) | 49.97 (21.41 to 115.21) | 161.97 (69.39 to 373.42) | 0 (-0.06 to 0.06) | 0.966 |
| Eastern Europe | 1.44 (0.28 to 4.47) | 4.02 (0.77 to 12.46) | 1.6 (0.32 to 4.8) | 4.14 (0.83 to 12.43) | 0.36 (-0.09 to 0.81) | 0.114 | 76.08 (35.15 to 171.19) | 211.99 (97.94 to 477.01) | 82.96 (38.23 to 183.81) | 214.82 (99.01 to 476.01) | 0.32 (-0.05 to 0.7) | 0.085 |
| East Asia | 5.25 (1 to 15.45) | 4.33 (0.83 to 12.73) | 11.31 (2.12 to 34.88) | 4.36 (0.82 to 13.46) | 0.19 (-0.03 to 0.41) | 0.085 | 265.43 (117.81 to 598.72) | 218.67 (97.05 to 493.23) | 620.79 (279.76 to 1382.99) | 239.55 (107.95 to 533.66) | 0.38 (0.18 to 0.58) | <0.001 |
| Central Sub-Saharan Africa | 0.18 (0.03 to 0.55) | 4.57 (0.87 to 13.9) | 0.49 (0.09 to 1.47) | 4.68 (0.83 to 14.09) | 0.1 (-0.17 to 0.37) | 0.443 | 8.68 (3.58 to 20.78) | 221.14 (91.37 to 529.65) | 22.76 (9.13 to 54.86) | 217.69 (87.32 to 524.7) | -0.02 (-0.25 to 0.2) | 0.851 |
| Central Latin America | 0.33 (0.06 to 1.05) | 2.58 (0.49 to 8.1) | 1.04 (0.2 to 3.16) | 2.93 (0.56 to 8.95) | 0.48 (0.33 to 0.62) | <0.001 | 18.4 (8.54 to 40.93) | 142.29 (66.02 to 316.5) | 55.7 (25.5 to 124.12) | 157.67 (72.18 to 351.34) | 0.41 (0.28 to 0.53) | <0.001 |
| Central Europe | 0.7 (0.13 to 2.2) | 3.81 (0.73 to 11.98) | 0.77 (0.15 to 2.39) | 3.78 (0.72 to 11.83) | 0.45 (0.22 to 0.68) | <0.001 | 36.69 (17.03 to 83.31) | 199.53 (92.6 to 453.02) | 40.13 (18.64 to 89.36) | 198.28 (92.11 to 441.48) | 0.42 (0.23 to 0.62) | <0.001 |
| Central Asia | 0.23 (0.04 to 0.7) | 3.55 (0.68 to 10.81) | 0.42 (0.08 to 1.29) | 3.26 (0.64 to 9.99) | -0.53 (-1.1 to 0.06) | 0.074 | 12.21 (5.69 to 27.53) | 188.95 (88.07 to 425.98) | 22.41 (10.27 to 49.55) | 173.99 (79.77 to 384.75) | -0.38 (-0.89 to 0.13) | 0.14 |
| Caribbean | 0.09 (0.02 to 0.28) | 2.59 (0.5 to 8.04) | 0.19 (0.04 to 0.6) | 2.89 (0.56 to 8.96) | 0.25 (0.15 to 0.35) | <0.001 | 4.74 (2.15 to 10.76) | 137.94 (62.64 to 313.04) | 10.08 (4.64 to 23.26) | 151.61 (69.69 to 349.7) | 0.19 (0.1 to 0.29) | <0.001 |
| Australasia | 0.09 (0.02 to 0.27) | 3.35 (0.66 to 10.26) | 0.18 (0.04 to 0.55) | 3.71 (0.74 to 11.29) | 0.69 (0.5 to 0.87) | <0.001 | 4.74 (2.2 to 10.49) | 180.8 (83.88 to 399.86) | 9.49 (4.32 to 21.15) | 194.48 (88.54 to 433.59) | 0.55 (0.4 to 0.7) | <0.001 |
| Andean Latin America | 0.07 (0.01 to 0.23) | 2.4 (0.46 to 7.34) | 0.21 (0.04 to 0.65) | 2.54 (0.5 to 7.99) | 0.22 (0.15 to 0.29) | <0.001 | 3.87 (1.74 to 8.85) | 126.12 (56.65 to 288.6) | 10.83 (4.76 to 24.73) | 132.87 (58.35 to 303.34) | 0.22 (0.16 to 0.27) | <0.001 |

Note: CI, confidence interval; AAPC, average annual percentage change; EOAD, early-onset Alzheimer’s disease and other dementias; DALYs, disability-adjusted life years.

**Supplementary Table S9. Age-standardized prevalence and incidence of EOAD in people aged 40-64 years and their AAPCs from 1990 to 2021 at the country levels.**

|  | Prevalence | | | | | | Incidence | | | | | |
| --- | --- | --- | --- | --- | --- | --- | --- | --- | --- | --- | --- | --- |
| Country | No of people with EOAD in 1990 (000s) | Age standardised rate in 1990 (per 100 000) | No of people with EOAD in 2021 (000s) | Age standardised rate in 2021 (per 100 000) | AAPC (95% CI) | *p* value | No of people with EOAD in 1990 (000s) | Age standardised rate in 1990 (per 100 000) | No of people with EOAD in 2021 (000s) | Age standardised rate in 2021 (per 100 000) | AAPC (95% CI) | *p* value |
| American Samoa | 22.65 (16.79 to 29.85) | 294.75 (218.49 to 388.37) | 48.73 (36.22 to 63.65) | 337.63 (250.92 to 441) | 0.47 (0.3 to 0.65) | <0.001 | 4.18 (2.65 to 6.01) | 54.41 (34.54 to 78.24) | 8.76 (5.66 to 12.58) | 60.72 (39.18 to 87.17) | 0.39 (0.25 to 0.53) | <0.001 |
| Antigua and Barbuda | 30.6 (22.63 to 40.36) | 276.64 (204.54 to 364.85) | 86.13 (63.59 to 113.75) | 295.18 (217.92 to 389.81) | 0.33 (0.08 to 0.59) | 0.013 | 5.36 (3.32 to 7.85) | 48.45 (30 to 71.01) | 14.87 (9.36 to 21.75) | 50.95 (32.08 to 74.55) | 0.27 (0.06 to 0.47) | 0.014 |
| Arab Republic of Egypt | 29928.73 (22572.41 to 38916.32) | 318.2 (239.99 to 413.76) | 69894.68 (52330.38 to 90653.54) | 315.05 (235.88 to 408.62) | 0.12 (0.04 to 0.2) | 0.005 | 5720.36 (3686.18 to 8076.92) | 60.82 (39.19 to 85.87) | 13519.72 (8685.69 to 19063.54) | 60.94 (39.15 to 85.93) | 0.12 (0.06 to 0.18) | <0.001 |
| Argentine Republic | 25438.18 (18757.86 to 33125.13) | 323.25 (238.36 to 420.93) | 37537.63 (27888.55 to 49316.94) | 299.59 (222.58 to 393.6) | -0.11 (-0.17 to -0.05) | 0.001 | 4292.24 (2701.21 to 6236.39) | 54.54 (34.33 to 79.25) | 6313.52 (3952.19 to 9224.07) | 50.39 (31.54 to 73.62) | -0.15 (-0.2 to -0.1) | <0.001 |
| Australia | 14591.88 (10745.34 to 19165.89) | 328.34 (241.79 to 431.27) | 27613.29 (20361.96 to 35958.68) | 345.75 (254.95 to 450.24) | 0.45 (0.34 to 0.57) | <0.001 | 2496.93 (1576.44 to 3631.54) | 56.19 (35.47 to 81.72) | 4654.17 (2917.01 to 6769.2) | 58.28 (36.52 to 84.76) | 0.36 (0.26 to 0.46) | <0.001 |
| Barbados | 154.54 (114.01 to 201.94) | 294.83 (217.5 to 385.25) | 349.76 (262.62 to 453.97) | 336.29 (252.51 to 436.49) | 0.69 (0.46 to 0.92) | <0.001 | 26.89 (16.9 to 39.26) | 51.31 (32.24 to 74.9) | 59.19 (37.43 to 85.17) | 56.91 (35.99 to 81.89) | 0.54 (0.36 to 0.73) | <0.001 |
| Belize | 67.97 (50.21 to 89.48) | 284.1 (209.87 to 374.01) | 251.37 (185.02 to 331.03) | 265.48 (195.41 to 349.61) | -0.13 (-0.37 to 0.11) | 0.275 | 12.02 (7.6 to 17.71) | 50.23 (31.75 to 74.03) | 44.66 (27.99 to 65.12) | 47.17 (29.56 to 68.77) | -0.13 (-0.32 to 0.07) | 0.185 |
| Bermuda | 47.38 (35.25 to 62.11) | 289.44 (215.34 to 379.44) | 87.53 (64.81 to 114.23) | 357.85 (264.96 to 466.98) | 0.83 (0.67 to 1) | <0.001 | 8.26 (5.15 to 12.08) | 50.44 (31.49 to 73.82) | 14.7 (9.45 to 21.6) | 60.08 (38.63 to 88.32) | 0.69 (0.55 to 0.82) | <0.001 |
| Bolivarian Republic of Venezuela | 9838.77 (7374.55 to 12758.35) | 331.91 (248.78 to 430.4) | 29309.85 (21888.51 to 38011.08) | 363.48 (271.44 to 471.38) | 0.57 (0.41 to 0.73) | <0.001 | 1766.08 (1130.55 to 2529.38) | 59.58 (38.14 to 85.33) | 5100.81 (3229.61 to 7350.9) | 63.26 (40.05 to 91.16) | 0.43 (0.31 to 0.56) | <0.001 |
| Bosnia and Herzegovina | 4349.89 (3256.42 to 5681.27) | 358.8 (268.6 to 468.61) | 4705.51 (3530.64 to 6068.08) | 391.93 (294.07 to 505.42) | 0.36 (0.12 to 0.59) | 0.004 | 751.16 (475.91 to 1086.2) | 61.96 (39.25 to 89.59) | 804.88 (516.86 to 1159.6) | 67.04 (43.05 to 96.58) | 0.32 (0.14 to 0.5) | 0.001 |
| Brunei Darussalam | 83.31 (60.36 to 110.99) | 218.18 (158.08 to 290.67) | 336.79 (248.37 to 442.27) | 263.16 (194.07 to 345.58) | 0.91 (0.67 to 1.15) | <0.001 | 15.84 (9.8 to 23.14) | 41.49 (25.67 to 60.6) | 61.05 (38.62 to 88.65) | 47.71 (30.17 to 69.27) | 0.71 (0.51 to 0.91) | <0.001 |
| Burkina Faso | 3326.8 (2443.54 to 4366.99) | 258.79 (190.08 to 339.7) | 6742.32 (4889.1 to 8953.29) | 221.48 (160.61 to 294.11) | -0.51 (-0.53 to -0.48) | <0.001 | 594.77 (370.89 to 863.92) | 46.27 (28.85 to 67.2) | 1215.73 (756.48 to 1784.94) | 39.94 (24.85 to 58.63) | -0.47 (-0.49 to -0.46) | <0.001 |
| Canada | 35285.73 (27702.09 to 43935.27) | 482.34 (378.67 to 600.57) | 75198.85 (60453.64 to 91057.39) | 606.41 (487.5 to 734.29) | 0.76 (0.64 to 0.87) | <0.001 | 5859.76 (3989.89 to 8075.82) | 80.1 (54.54 to 110.39) | 11851.5 (8455.41 to 15825.45) | 95.57 (68.18 to 127.62) | 0.51 (0.41 to 0.62) | <0.001 |
| Central African Republic | 1383.76 (1021.84 to 1814.31) | 351.16 (259.32 to 460.43) | 2778.84 (2034.42 to 3650.34) | 311.57 (228.1 to 409.29) | -0.51 (-0.64 to -0.37) | <0.001 | 259.4 (165.97 to 377.37) | 65.83 (42.12 to 95.77) | 531 (330.48 to 767.99) | 59.54 (37.05 to 86.11) | -0.47 (-0.58 to -0.36) | <0.001 |
| Commonwealth of Dominica | 37.04 (27.34 to 48.81) | 291.45 (215.1 to 384.06) | 65 (48.01 to 85.01) | 321.26 (237.28 to 420.18) | 0.25 (0 to 0.51) | 0.05 | 6.48 (4.04 to 9.43) | 50.99 (31.79 to 74.18) | 11.1 (6.97 to 16.2) | 54.87 (34.43 to 80.06) | 0.19 (-0.03 to 0.4) | 0.082 |
| Commonwealth of the Bahamas | 112.06 (81.98 to 148.7) | 244.75 (179.07 to 324.78) | 341.15 (251.15 to 448.28) | 283.64 (208.81 to 372.71) | 0.37 (0.18 to 0.55) | <0.001 | 20.18 (12.49 to 29.76) | 44.09 (27.29 to 65) | 59.69 (37.18 to 87.32) | 49.63 (30.91 to 72.6) | 0.29 (0.14 to 0.44) | <0.001 |
| Cook Islands | 12.21 (9.1 to 15.94) | 334.97 (249.81 to 437.48) | 23.1 (17.33 to 30.05) | 402.38 (301.89 to 523.54) | 0.31 (0.13 to 0.49) | 0.001 | 2.21 (1.41 to 3.18) | 60.58 (38.72 to 87.36) | 4.02 (2.6 to 5.76) | 70.06 (45.29 to 100.32) | 0.25 (0.1 to 0.39) | 0.002 |
| Czech Republic | 10529.26 (7912.23 to 13728.62) | 341.61 (256.7 to 445.41) | 12966.15 (9695.03 to 16997.6) | 345.29 (258.18 to 452.65) | 0.46 (0.21 to 0.71) | 0.001 | 1841.76 (1183.1 to 2640.39) | 59.75 (38.38 to 85.66) | 2260.35 (1433.73 to 3267.24) | 60.19 (38.18 to 87.01) | 0.35 (0.14 to 0.56) | 0.002 |
| Democratic People's Republic of Korea | 15042.27 (10975.55 to 19853.48) | 290.97 (212.31 to 384.04) | 27151.22 (20178.23 to 35768.92) | 305.47 (227.02 to 402.43) | -0.53 (-0.81 to -0.25) | 0.001 | 2786.6 (1760.38 to 4017.07) | 53.9 (34.05 to 77.71) | 4955.18 (3146.32 to 7152.64) | 55.75 (35.4 to 80.47) | -0.47 (-0.71 to -0.23) | <0.001 |
| Democratic Republic of Sao Tome and Principe | 42.8 (31.59 to 56.17) | 262.19 (193.54 to 344.08) | 79.8 (57.54 to 106.43) | 198.83 (143.36 to 265.17) | -1.04 (-1.24 to -0.85) | <0.001 | 7.39 (4.61 to 10.74) | 45.3 (28.23 to 65.79) | 14.32 (8.66 to 20.93) | 35.69 (21.58 to 52.15) | -0.89 (-1.04 to -0.74) | <0.001 |
| Democratic Republic of Timor-Leste | 372.25 (278.47 to 489.83) | 325.65 (243.61 to 428.51) | 839.49 (631.94 to 1098.04) | 362.79 (273.1 to 474.52) | 0.65 (0.42 to 0.87) | <0.001 | 70.71 (45.42 to 101.38) | 61.86 (39.73 to 88.69) | 154.08 (100.19 to 220.85) | 66.59 (43.3 to 95.44) | 0.48 (0.3 to 0.66) | <0.001 |
| Democratic Republic of the Congo | 18675.98 (13878.65 to 24625.05) | 369.79 (274.8 to 487.59) | 43972.68 (32603.33 to 57837.02) | 323.59 (239.92 to 425.61) | -0.4 (-0.59 to -0.22) | <0.001 | 3462.75 (2205.13 to 5024.63) | 68.56 (43.66 to 99.49) | 8189.64 (5221.97 to 11853.19) | 60.27 (38.43 to 87.23) | -0.41 (-0.55 to -0.26) | <0.001 |
| Democratic Socialist Republic of Sri Lanka | 12334.17 (9244.35 to 16235.67) | 364.07 (272.87 to 479.24) | 27587.64 (20904.89 to 35914.96) | 414.85 (314.35 to 540.07) | 0.69 (0.58 to 0.81) | <0.001 | 2248.97 (1453.75 to 3206.49) | 66.38 (42.91 to 94.65) | 4856.68 (3177.17 to 6917.1) | 73.03 (47.78 to 104.01) | 0.53 (0.43 to 0.62) | <0.001 |
| Dominican Republic | 2927.97 (2159.15 to 3858.77) | 267.72 (197.42 to 352.83) | 7174.77 (5269.07 to 9467.81) | 267.31 (196.31 to 352.75) | -0.02 (-0.09 to 0.06) | 0.673 | 521.34 (322.62 to 756.73) | 47.67 (29.5 to 69.19) | 1275.83 (797.36 to 1857.03) | 47.53 (29.71 to 69.19) | -0.05 (-0.11 to 0.01) | 0.077 |
| Eastern Republic of Uruguay | 2838.04 (2132.41 to 3686.38) | 347.81 (261.33 to 451.78) | 3313.61 (2465.56 to 4329.22) | 325.34 (242.07 to 425.05) | -0.18 (-0.27 to -0.1) | <0.001 | 472.73 (298.49 to 682.73) | 57.93 (36.58 to 83.67) | 549.89 (343.7 to 805.55) | 53.99 (33.74 to 79.09) | -0.21 (-0.28 to -0.14) | <0.001 |
| Federal Democratic Republic of Ethiopia | 21905.58 (16013.46 to 28989.56) | 334.3 (244.38 to 442.41) | 43331.99 (31531.61 to 58051.55) | 288.71 (210.09 to 386.78) | -0.33 (-0.38 to -0.28) | <0.001 | 3793.48 (2391.72 to 5464.51) | 57.89 (36.5 to 83.39) | 7626.26 (4790.36 to 11144.52) | 50.81 (31.92 to 74.25) | -0.3 (-0.34 to -0.25) | <0.001 |
| Federal Democratic Republic of Nepal | 7548.3 (5529.85 to 9970.04) | 245.03 (179.51 to 323.65) | 15721.13 (11479.95 to 20696.86) | 238.91 (174.46 to 314.53) | -0.07 (-0.08 to -0.06) | <0.001 | 1380.38 (867.13 to 2022.52) | 44.81 (28.15 to 65.65) | 2818.96 (1761.07 to 4102.58) | 42.84 (26.76 to 62.35) | -0.16 (-0.16 to -0.15) | <0.001 |
| Federal Republic of Germany | 94038.68 (70206.83 to 122179.92) | 370.86 (276.87 to 481.84) | 121056.68 (90960.63 to 157551.86) | 409.62 (307.78 to 533.11) | 0.1 (-0.14 to 0.34) | 0.418 | 15975.15 (9949.39 to 23285.74) | 63 (39.24 to 91.83) | 20467.99 (12790.02 to 29390.52) | 69.26 (43.28 to 99.45) | 0.12 (-0.09 to 0.33) | 0.26 |
| Federal Republic of Nigeria | 31218.92 (22591.02 to 41567.37) | 234.18 (169.46 to 311.8) | 71497.03 (51219.08 to 95488.97) | 213.82 (153.17 to 285.57) | -0.38 (-0.5 to -0.27) | <0.001 | 5250.63 (3262.24 to 7666.59) | 39.39 (24.47 to 57.51) | 12064.52 (7401.73 to 17646.35) | 36.08 (22.14 to 52.77) | -0.35 (-0.44 to -0.25) | <0.001 |
| Federal Republic of Somalia | 2559.06 (1889.84 to 3373.94) | 252.24 (186.28 to 332.56) | 6088.6 (4516.72 to 8039.66) | 234.18 (173.72 to 309.22) | 0.33 (0.03 to 0.64) | 0.034 | 485.92 (309.29 to 704.29) | 47.9 (30.49 to 69.42) | 1165.26 (740.25 to 1701.56) | 44.82 (28.47 to 65.45) | 0.25 (0 to 0.5) | 0.054 |
| Federated States of Micronesia | 42.75 (31.92 to 56.1) | 315.87 (235.81 to 414.5) | 88.99 (66.5 to 117.58) | 360.17 (269.17 to 475.9) | 0.82 (0.56 to 1.09) | <0.001 | 7.87 (5.04 to 11.25) | 58.16 (37.23 to 83.14) | 16.16 (10.41 to 23.24) | 65.41 (42.14 to 94.07) | 0.71 (0.49 to 0.92) | <0.001 |
| Federative Republic of Brazil | 109936.46 (82376.35 to 143210.91) | 408.85 (306.36 to 532.6) | 289786.2 (217698.92 to 377988.55) | 444.76 (334.12 to 580.13) | 0.41 (0.33 to 0.5) | <0.001 | 19672.97 (12765.74 to 27793.99) | 73.16 (47.48 to 103.37) | 52408.87 (34010.3 to 74716.82) | 80.44 (52.2 to 114.67) | 0.44 (0.35 to 0.53) | <0.001 |
| French Republic | 42028.7 (30676.44 to 55378.39) | 260.64 (190.24 to 343.42) | 57441.64 (41561.04 to 75898.24) | 271.53 (196.46 to 358.77) | 0.38 (0.23 to 0.53) | <0.001 | 6975.92 (4190.53 to 10274.19) | 43.26 (25.99 to 63.71) | 9460.04 (5739.66 to 14067.07) | 44.72 (27.13 to 66.49) | 0.32 (0.19 to 0.45) | <0.001 |
| Gabonese Republic | 580.65 (431.85 to 754.28) | 400.37 (297.77 to 520.1) | 1196.85 (887.78 to 1566.99) | 339.37 (251.73 to 444.33) | -0.47 (-0.72 to -0.22) | 0.001 | 106.08 (68.2 to 152.37) | 73.14 (47.02 to 105.06) | 220.4 (141.43 to 318.86) | 62.5 (40.1 to 90.41) | -0.47 (-0.68 to -0.26) | <0.001 |
| Georgia | 5947.79 (4449.07 to 7756.99) | 391.46 (292.82 to 510.53) | 4600.06 (3457.32 to 6023.25) | 394.98 (296.86 to 517.18) | 0 (-0.33 to 0.32) | 0.979 | 1011.12 (652.82 to 1458.73) | 66.55 (42.97 to 96.01) | 784.8 (504.5 to 1123.63) | 67.39 (43.32 to 96.48) | 0.01 (-0.25 to 0.28) | 0.914 |
| Grand Duchy of Luxembourg | 291.16 (211.69 to 385.1) | 248.37 (180.58 to 328.51) | 523.67 (373.04 to 701.76) | 231.27 (164.74 to 309.92) | -0.26 (-0.42 to -0.1) | 0.002 | 49.48 (30.39 to 74.41) | 42.21 (25.92 to 63.47) | 86.86 (52.74 to 130.08) | 38.36 (23.29 to 57.45) | -0.35 (-0.5 to -0.21) | <0.001 |
| Greenland | 46.2 (34.84 to 60.59) | 361.18 (272.4 to 473.73) | 96.76 (73.95 to 125.16) | 518.38 (396.15 to 670.5) | 0.97 (0.67 to 1.27) | <0.001 | 8.24 (5.36 to 11.85) | 64.41 (41.91 to 92.68) | 16.03 (10.53 to 22.98) | 85.86 (56.42 to 123.11) | 0.78 (0.53 to 1.03) | <0.001 |
| Grenada | 43.32 (32.45 to 56.31) | 324.3 (242.9 to 421.54) | 92.33 (68.01 to 122.09) | 297.41 (219.07 to 393.24) | -0.13 (-0.28 to 0.02) | 0.096 | 7.46 (4.73 to 10.88) | 55.82 (35.42 to 81.47) | 15.99 (10.06 to 23.49) | 51.51 (32.39 to 75.67) | -0.13 (-0.26 to -0.01) | 0.041 |
| Guam | 81.64 (60.6 to 107.73) | 308.81 (229.23 to 407.49) | 182.86 (136.81 to 238.99) | 380.63 (284.77 to 497.49) | 0.75 (0.57 to 0.93) | <0.001 | 14.8 (9.3 to 21.34) | 55.98 (35.19 to 80.72) | 31.93 (20.42 to 45.76) | 66.47 (42.52 to 95.26) | 0.6 (0.45 to 0.74) | <0.001 |
| Hashemite Kingdom of Jordan | 1548.44 (1154.76 to 2036.14) | 324.05 (241.66 to 426.11) | 9133.05 (6755.08 to 11940.02) | 324.81 (240.24 to 424.64) | -0.38 (-0.55 to -0.22) | <0.001 | 296.95 (189.19 to 426.65) | 62.14 (39.59 to 89.29) | 1750.11 (1116.33 to 2525.72) | 62.24 (39.7 to 89.83) | -0.32 (-0.45 to -0.18) | <0.001 |
| Hellenic Republic | 10829.15 (7926.58 to 14216.33) | 338.53 (247.79 to 444.42) | 11517.78 (8372.49 to 15186.54) | 312.52 (227.17 to 412.06) | -0.34 (-0.42 to -0.27) | <0.001 | 1816.43 (1120.19 to 2705.32) | 56.78 (35.02 to 84.57) | 1929.03 (1183.46 to 2873.32) | 52.34 (32.11 to 77.96) | -0.35 (-0.41 to -0.28) | <0.001 |
| Hungary | 11424.34 (8529.89 to 14825.41) | 358.24 (267.48 to 464.89) | 11936.64 (8976.14 to 15515.97) | 346.23 (260.36 to 450.05) | 0.36 (0.18 to 0.55) | <0.001 | 1997.72 (1286.02 to 2901.84) | 62.64 (40.33 to 91) | 2093.76 (1361.54 to 3002.16) | 60.73 (39.49 to 87.08) | 0.29 (0.13 to 0.45) | 0.001 |
| Independent State of Papua New Guinea | 2033.85 (1510.57 to 2668.2) | 322.87 (239.8 to 423.57) | 5552.94 (4138.35 to 7364.92) | 284.66 (212.14 to 377.54) | -0.4 (-0.51 to -0.29) | <0.001 | 374.73 (235.75 to 533.56) | 59.49 (37.42 to 84.7) | 1033.17 (659.8 to 1495.04) | 52.96 (33.82 to 76.64) | -0.38 (-0.47 to -0.29) | <0.001 |
| Independent State of Samoa | 80.47 (59.5 to 105.32) | 333.45 (246.55 to 436.46) | 137.32 (102.02 to 181.07) | 324.59 (241.17 to 428.01) | -0.14 (-0.33 to 0.05) | 0.138 | 14.47 (9.21 to 20.76) | 59.97 (38.16 to 86.04) | 24.71 (15.6 to 35.74) | 58.41 (36.87 to 84.47) | -0.13 (-0.28 to 0.02) | 0.085 |
| Ireland | 2346.59 (1685.76 to 3127.77) | 277.43 (199.3 to 369.78) | 4411.79 (3130.85 to 5902.01) | 270.99 (192.31 to 362.52) | 0.03 (-0.02 to 0.07) | 0.23 | 398.74 (241.75 to 593.3) | 47.14 (28.58 to 70.14) | 739.8 (437.41 to 1108.57) | 45.44 (26.87 to 68.09) | -0.05 (-0.08 to -0.01) | 0.022 |
| Islamic Republic of Afghanistan | 7635.34 (5741.04 to 9964.08) | 392.39 (295.04 to 512.06) | 9286.08 (6620.16 to 12605.41) | 222.29 (158.47 to 301.75) | -2.77 (-3.38 to -2.15) | <0.001 | 1437.28 (932.68 to 2046.42) | 73.86 (47.93 to 105.17) | 1899.33 (1172.25 to 2774.64) | 45.47 (28.06 to 66.42) | -2.33 (-2.86 to -1.81) | <0.001 |
| Islamic Republic of Iran | 33235.6 (24826.27 to 43457.66) | 409.61 (305.97 to 535.6) | 88970.86 (65938.17 to 116901.69) | 363.68 (269.53 to 477.84) | -0.22 (-0.45 to 0.02) | 0.067 | 6006.9 (3880.87 to 8594.99) | 74.03 (47.83 to 105.93) | 16249.46 (10421.85 to 23250.25) | 66.42 (42.6 to 95.04) | -0.21 (-0.4 to -0.01) | 0.037 |
| Islamic Republic of Mauritania | 645.78 (466.72 to 859.3) | 232.31 (167.9 to 309.12) | 1519.35 (1118.24 to 2014.71) | 227.62 (167.53 to 301.83) | 0.01 (-0.12 to 0.15) | 0.864 | 116.38 (72.31 to 169.08) | 41.87 (26.01 to 60.82) | 271.63 (169.28 to 396.45) | 40.69 (25.36 to 59.39) | -0.03 (-0.14 to 0.08) | 0.555 |
| Islamic Republic of Pakistan | 39274.73 (28355.4 to 52200.28) | 237.2 (171.25 to 315.26) | 91092.99 (65270.04 to 121747.28) | 213.54 (153 to 285.39) | -0.38 (-0.41 to -0.34) | <0.001 | 6889.17 (4330.07 to 10030.49) | 41.61 (26.15 to 60.58) | 15996.28 (9922.19 to 23387.22) | 37.5 (23.26 to 54.82) | -0.37 (-0.39 to -0.35) | <0.001 |
| Jamaica | 1264.35 (946.06 to 1662.94) | 334.19 (250.06 to 439.54) | 2500.52 (1869.2 to 3272.58) | 331.94 (248.13 to 434.43) | -0.06 (-0.25 to 0.13) | 0.53 | 220.72 (139.55 to 321.79) | 58.34 (36.89 to 85.05) | 430.91 (271.6 to 623.67) | 57.2 (36.05 to 82.79) | -0.1 (-0.25 to 0.05) | 0.192 |
| Japan | 149674.3 (111536.4 to 196437.77) | 348.86 (259.97 to 457.86) | 163139.13 (121532 to 212972.77) | 379.45 (282.67 to 495.35) | 0.36 (0.13 to 0.59) | 0.003 | 25008.5 (16035.24 to 35923.3) | 58.29 (37.38 to 83.73) | 27990.49 (17986.99 to 40165.78) | 65.1 (41.84 to 93.42) | 0.52 (0.33 to 0.71) | <0.001 |
| Kingdom of Bahrain | 218.2 (161.8 to 289.86) | 285.69 (211.85 to 379.52) | 1355.13 (995.54 to 1805.38) | 285.4 (209.67 to 380.23) | 0.21 (-0.18 to 0.59) | 0.288 | 42.42 (26.34 to 61.44) | 55.54 (34.49 to 80.45) | 261.92 (164.37 to 379.58) | 55.16 (34.62 to 79.94) | 0.14 (-0.18 to 0.45) | 0.384 |
| Kingdom of Belgium | 10625.45 (7865.55 to 13866.26) | 357.2 (264.42 to 466.15) | 13077.49 (9627.29 to 17200.96) | 343.08 (252.57 to 451.26) | -0.03 (-0.2 to 0.14) | 0.734 | 1771.41 (1102.05 to 2590.14) | 59.55 (37.05 to 87.07) | 2164.92 (1331.06 to 3169.23) | 56.8 (34.92 to 83.14) | -0.08 (-0.23 to 0.07) | 0.263 |
| Kingdom of Bhutan | 188.94 (136.75 to 250.42) | 229.58 (166.17 to 304.29) | 373.7 (272.52 to 496.79) | 215.95 (157.48 to 287.08) | -0.14 (-0.19 to -0.1) | <0.001 | 34 (20.84 to 49.89) | 41.32 (25.32 to 60.62) | 67.19 (41.25 to 98.92) | 38.83 (23.84 to 57.16) | -0.15 (-0.19 to -0.12) | <0.001 |
| Kingdom of Cambodia | 5119.1 (3839.1 to 6677.84) | 355.46 (266.58 to 463.69) | 14176.87 (10716.28 to 18561.53) | 377.67 (285.48 to 494.47) | 0.23 (0.05 to 0.41) | 0.013 | 946.12 (613.78 to 1357.79) | 65.7 (42.62 to 94.28) | 2579.39 (1678.73 to 3682.44) | 68.71 (44.72 to 98.1) | 0.16 (0.02 to 0.31) | 0.025 |
| Kingdom of Denmark | 4026.55 (2930.07 to 5344.78) | 259.99 (189.19 to 345.1) | 5111.39 (3673.03 to 6819.04) | 269.68 (193.79 to 359.78) | 0.12 (0.02 to 0.22) | 0.022 | 694.59 (421.3 to 1024.36) | 44.85 (27.2 to 66.14) | 816.7 (486.41 to 1234.28) | 43.09 (25.66 to 65.12) | -0.13 (-0.25 to -0.02) | 0.028 |
| Kingdom of Eswatini | 260.77 (191.31 to 343.9) | 263.21 (193.1 to 347.12) | 504.15 (372.22 to 666.98) | 260.63 (192.42 to 344.8) | 0.07 (0 to 0.13) | 0.046 | 48.24 (29.78 to 70.23) | 48.69 (30.06 to 70.89) | 92.42 (57.86 to 135.1) | 47.78 (29.91 to 69.84) | 0.03 (-0.02 to 0.08) | 0.271 |
| Kingdom of Lesotho | 735.78 (544.57 to 961.3) | 304.81 (225.59 to 398.23) | 966.1 (714.33 to 1265.71) | 294.4 (217.67 to 385.69) | 0.24 (0.11 to 0.36) | 0.001 | 134.68 (84.54 to 194.3) | 55.79 (35.02 to 80.49) | 175.91 (110.67 to 254.81) | 53.6 (33.72 to 77.65) | 0.17 (0.07 to 0.28) | 0.002 |
| Kingdom of Morocco | 14555.78 (10881.02 to 18965.46) | 360.67 (269.62 to 469.94) | 35672.46 (26801.04 to 46368.7) | 357.93 (268.92 to 465.26) | 0.06 (-0.15 to 0.26) | 0.577 | 2770.79 (1787.98 to 3982.19) | 68.66 (44.3 to 98.67) | 6693.93 (4308.24 to 9676.47) | 67.17 (43.23 to 97.09) | -0.01 (-0.18 to 0.17) | 0.932 |
| Kingdom of Norway | 3755.74 (2758.96 to 4957.37) | 327.12 (240.3 to 431.78) | 5662.07 (4063.7 to 7546.68) | 323.97 (232.51 to 431.8) | 0.09 (0.01 to 0.18) | 0.034 | 632.92 (393.78 to 931.75) | 55.13 (34.3 to 81.15) | 921 (563.11 to 1374.42) | 52.7 (32.22 to 78.64) | -0.05 (-0.13 to 0.03) | 0.211 |
| Kingdom of Saudi Arabia | 6006.46 (4409.16 to 7923.96) | 265.54 (194.93 to 350.31) | 23707.86 (17125.56 to 31594.84) | 223.77 (161.64 to 298.21) | -0.6 (-0.65 to -0.55) | <0.001 | 1184.91 (745.35 to 1724.42) | 52.38 (32.95 to 76.24) | 4761.41 (2972.41 to 6906.06) | 44.94 (28.06 to 65.18) | -0.53 (-0.57 to -0.49) | <0.001 |
| Kingdom of Spain | 34811.24 (26175.17 to 45005.57) | 320.01 (240.62 to 413.73) | 49437.19 (36034.94 to 65391.56) | 283.18 (206.41 to 374.57) | -0.43 (-0.54 to -0.31) | <0.001 | 5858.49 (3821.7 to 8184.89) | 53.86 (35.13 to 75.24) | 8116.89 (4945.83 to 12026.8) | 46.49 (28.33 to 68.89) | -0.42 (-0.52 to -0.32) | <0.001 |
| Kingdom of Sweden | 7891.45 (5735.5 to 10449.51) | 306.69 (222.9 to 406.1) | 10771.03 (7809.31 to 14447.55) | 339.18 (245.92 to 454.96) | 0.49 (0.38 to 0.6) | <0.001 | 1307.03 (806.61 to 1926.86) | 50.8 (31.35 to 74.88) | 1726.88 (1067.4 to 2543.89) | 54.38 (33.61 to 80.11) | 0.43 (0.33 to 0.53) | <0.001 |
| Kingdom of Thailand | 40574.08 (30629.07 to 52178.95) | 354.07 (267.28 to 455.34) | 104852.9 (79140.66 to 136229.75) | 402.13 (303.52 to 522.46) | 0.71 (0.51 to 0.9) | <0.001 | 7282.43 (4724.8 to 10309.65) | 63.55 (41.23 to 89.97) | 18489.37 (12081.94 to 26154.2) | 70.91 (46.34 to 100.31) | 0.68 (0.52 to 0.84) | <0.001 |
| Kingdom of Tonga | 55.79 (41.94 to 73.04) | 352.19 (264.71 to 461.06) | 71.97 (53.45 to 94.18) | 331.66 (246.29 to 433.99) | -0.44 (-0.55 to -0.33) | <0.001 | 10.05 (6.4 to 14.37) | 63.41 (40.39 to 90.69) | 12.98 (8.24 to 18.5) | 59.81 (37.97 to 85.27) | -0.4 (-0.49 to -0.31) | <0.001 |
| Kingdom of the Netherlands | 11999.04 (8930.88 to 15539.11) | 282.01 (209.9 to 365.22) | 19498.55 (14171.85 to 25595.73) | 336.79 (244.79 to 442.11) | 0.71 (0.58 to 0.83) | <0.001 | 1969.91 (1223.73 to 2857.33) | 46.3 (28.76 to 67.16) | 3247.66 (2025.81 to 4775.13) | 56.1 (34.99 to 82.48) | 0.82 (0.66 to 0.97) | <0.001 |
| Kyrgyz Republic | 2764.65 (2080.3 to 3601.29) | 366.59 (275.85 to 477.53) | 5069.41 (3788.59 to 6575.49) | 331.91 (248.05 to 430.52) | -0.28 (-0.83 to 0.27) | 0.304 | 474.19 (304.67 to 681.78) | 62.88 (40.4 to 90.4) | 888.81 (563.76 to 1278.45) | 58.19 (36.91 to 83.71) | -0.19 (-0.63 to 0.25) | 0.378 |
| Lao People's Democratic Republic | 2217.41 (1663.9 to 2923.49) | 354.3 (265.86 to 467.12) | 5294.41 (3935.04 to 6971.86) | 342.43 (254.51 to 450.92) | -0.13 (-0.28 to 0.01) | 0.071 | 408.1 (262.28 to 586.37) | 65.21 (41.91 to 93.69) | 981.76 (628.8 to 1397.42) | 63.5 (40.67 to 90.38) | -0.09 (-0.21 to 0.02) | 0.111 |
| Lebanese Republic | 2550.09 (1905.58 to 3305.21) | 406.59 (303.83 to 526.99) | 5320.74 (3989.67 to 6952.06) | 381.14 (285.79 to 498) | -0.28 (-0.34 to -0.22) | <0.001 | 477.71 (306.3 to 681.9) | 76.17 (48.84 to 108.72) | 1004.26 (648.85 to 1439.63) | 71.94 (46.48 to 103.13) | -0.25 (-0.3 to -0.2) | <0.001 |
| Malaysia | 10769.6 (8010.49 to 14054.39) | 359.39 (267.32 to 469.01) | 30784.18 (23113.74 to 40213.83) | 387.02 (290.59 to 505.57) | 0.59 (0.44 to 0.75) | <0.001 | 2008.25 (1282.65 to 2867.87) | 67.02 (42.8 to 95.7) | 5543.19 (3567.67 to 7951.62) | 69.69 (44.85 to 99.97) | 0.48 (0.35 to 0.61) | <0.001 |
| Mongolia | 863.13 (634.91 to 1132.22) | 297.9 (219.13 to 390.78) | 2542.8 (1892.03 to 3334.29) | 301.63 (224.43 to 395.52) | -0.24 (-0.62 to 0.13) | 0.193 | 154.62 (97.47 to 224.02) | 53.37 (33.64 to 77.32) | 455.75 (286.18 to 656.72) | 54.06 (33.95 to 77.9) | -0.16 (-0.46 to 0.14) | 0.273 |
| Montenegro | 595 (448.7 to 776.63) | 363.49 (274.12 to 474.45) | 783.14 (594.46 to 1015.86) | 377.99 (286.93 to 490.32) | 0.25 (0.08 to 0.43) | 0.006 | 103.58 (66.79 to 148.31) | 63.28 (40.8 to 90.6) | 135.07 (87.09 to 194.32) | 65.19 (42.03 to 93.79) | 0.2 (0.07 to 0.33) | 0.004 |
| New Zealand | 3355.97 (2494.61 to 4391.32) | 389.71 (289.69 to 509.94) | 6630.85 (4891.53 to 8731.59) | 420.57 (310.25 to 553.81) | 0.38 (0.27 to 0.49) | <0.001 | 550.93 (345.44 to 804.43) | 63.98 (40.11 to 93.41) | 1059.39 (668.69 to 1561.57) | 67.19 (42.41 to 99.04) | 0.27 (0.17 to 0.37) | <0.001 |
| North Macedonia | 1788.05 (1333.3 to 2332.78) | 341.25 (254.46 to 445.22) | 2813.66 (2118.95 to 3672.32) | 362.39 (272.91 to 472.98) | 0.3 (0.16 to 0.44) | <0.001 | 314.52 (201.35 to 454.55) | 60.03 (38.43 to 86.75) | 489.62 (316.78 to 699.08) | 63.06 (40.8 to 90.04) | 0.24 (0.13 to 0.36) | <0.001 |
| Northern Mariana Islands | 18.7 (13.51 to 25.12) | 215.43 (155.58 to 289.31) | 61.01 (45.36 to 80.3) | 355.62 (264.37 to 468.06) | 1.93 (1.53 to 2.34) | <0.001 | 3.66 (2.28 to 5.33) | 42.21 (26.21 to 61.35) | 10.85 (6.99 to 15.57) | 63.26 (40.76 to 90.78) | 1.55 (1.24 to 1.86) | <0.001 |
| Palestine | 905.89 (679.26 to 1177.03) | 374.03 (280.45 to 485.97) | 2970.14 (2207.69 to 3896.11) | 326.72 (242.85 to 428.58) | -0.63 (-0.82 to -0.44) | <0.001 | 170.82 (110.84 to 244.66) | 70.53 (45.76 to 101.02) | 564.93 (359.23 to 814.23) | 62.14 (39.52 to 89.57) | -0.58 (-0.75 to -0.42) | <0.001 |
| People's Democratic Republic of Algeria | 12966.04 (9752.62 to 17000.93) | 375.2 (282.21 to 491.96) | 37645.16 (28002.17 to 49532.15) | 341.38 (253.93 to 449.17) | -0.28 (-0.47 to -0.09) | 0.004 | 2437.81 (1572.85 to 3497.6) | 70.54 (45.51 to 101.21) | 7114.75 (4577.74 to 10201.2) | 64.52 (41.51 to 92.51) | -0.27 (-0.43 to -0.11) | 0.002 |
| People's Republic of Bangladesh | 32088.47 (23323.38 to 42693.76) | 221.23 (160.8 to 294.34) | 87935.92 (63929.37 to 116176.35) | 228.65 (166.23 to 302.08) | 0.17 (0.04 to 0.31) | 0.013 | 5848.47 (3640.82 to 8591.28) | 40.32 (25.1 to 59.23) | 15795.34 (9840.19 to 22863.34) | 41.07 (25.59 to 59.45) | 0.12 (0.01 to 0.24) | 0.036 |
| People's Republic of China | 940327.77 (700534.39 to 1229246.9) | 383.6 (285.78 to 501.47) | 2410831.98 (1803713.69 to 3158248.28) | 476.77 (356.7 to 624.58) | 0.67 (0.48 to 0.87) | <0.001 | 165221.96 (106448.02 to 235056.08) | 67.4 (43.43 to 95.89) | 453091.54 (296301.05 to 639314) | 89.6 (58.6 to 126.43) | 0.77 (0.63 to 0.91) | <0.001 |
| Plurinational State of Bolivia | 2301.73 (1682.55 to 3045.32) | 233.86 (170.95 to 309.42) | 6445.39 (4725.62 to 8509.92) | 243.52 (178.54 to 321.52) | 0.31 (0.24 to 0.37) | <0.001 | 397.56 (245.86 to 583.53) | 40.39 (24.98 to 59.29) | 1102.26 (683.77 to 1605.26) | 41.65 (25.83 to 60.65) | 0.25 (0.19 to 0.3) | <0.001 |
| Portuguese Republic | 9247.99 (6785.32 to 12216.41) | 318.41 (233.62 to 420.61) | 12146.65 (8869.59 to 16008.86) | 313.55 (228.96 to 413.25) | -0.01 (-0.09 to 0.06) | 0.771 | 1550.28 (963.45 to 2301.07) | 53.38 (33.17 to 79.23) | 2035.24 (1251.96 to 3013.52) | 52.54 (32.32 to 77.79) | -0.02 (-0.09 to 0.04) | 0.499 |
| Principality of Andorra | 40.82 (29.61 to 54.42) | 277.71 (201.4 to 370.19) | 102.74 (73.68 to 136.61) | 283.45 (203.28 to 376.9) | 0.04 (-0.11 to 0.18) | 0.605 | 7.02 (4.25 to 10.48) | 47.75 (28.9 to 71.31) | 17.22 (10.55 to 25.51) | 47.52 (29.09 to 70.38) | -0.05 (-0.17 to 0.08) | 0.456 |
| Principality of Monaco | 34.06 (25 to 44.62) | 322.23 (236.55 to 422.14) | 46.57 (34.07 to 61.12) | 335.13 (245.2 to 439.79) | 0.02 (-0.06 to 0.11) | 0.589 | 5.78 (3.59 to 8.47) | 54.71 (33.96 to 80.15) | 7.68 (4.71 to 11.42) | 55.24 (33.89 to 82.17) | -0.05 (-0.13 to 0.03) | 0.202 |
| Puerto Rico | 2500.37 (1847.24 to 3270.98) | 289.2 (213.65 to 378.33) | 3793.68 (2838.06 to 4955.2) | 347.86 (260.24 to 454.37) | 0.67 (0.64 to 0.7) | <0.001 | 438 (275.85 to 638.32) | 50.66 (31.91 to 73.83) | 640.18 (406.55 to 933.61) | 58.7 (37.28 to 85.61) | 0.53 (0.51 to 0.56) | <0.001 |
| Republic of Albania | 1968.23 (1462.97 to 2576.82) | 323.39 (240.37 to 423.39) | 3694.6 (2791.84 to 4785.66) | 429.45 (324.51 to 556.27) | 0.92 (0.72 to 1.12) | <0.001 | 343.23 (220.01 to 498.62) | 56.39 (36.15 to 81.93) | 619.85 (401.82 to 887.57) | 72.05 (46.71 to 103.17) | 0.8 (0.64 to 0.96) | <0.001 |
| Republic of Angola | 4646.68 (3440.44 to 6138.47) | 330.26 (244.53 to 436.29) | 14246.21 (10539.42 to 18755.95) | 312.85 (231.45 to 411.88) | -0.13 (-0.23 to -0.02) | 0.024 | 869.21 (552.13 to 1252.48) | 61.78 (39.24 to 89.02) | 2676.28 (1698.26 to 3887.06) | 58.77 (37.29 to 85.36) | -0.13 (-0.21 to -0.04) | 0.007 |
| Republic of Armenia | 3014.07 (2253.14 to 3922.59) | 398.98 (298.26 to 519.25) | 3829.53 (2909.13 to 5011.47) | 412.02 (313 to 539.19) | 0.33 (-0.22 to 0.88) | 0.233 | 509.85 (328.62 to 738.04) | 67.49 (43.5 to 97.7) | 645.37 (418.33 to 927.35) | 69.44 (45.01 to 99.77) | 0.29 (-0.16 to 0.73) | 0.196 |
| Republic of Austria | 6938.13 (5075.76 to 9146.66) | 306.71 (224.38 to 404.34) | 9921.19 (7211.22 to 13122.69) | 315.17 (229.08 to 416.87) | -0.06 (-0.2 to 0.09) | 0.428 | 1179.74 (730.94 to 1748.44) | 52.15 (32.31 to 77.29) | 1650.18 (1017.83 to 2452.37) | 52.42 (32.33 to 77.9) | -0.1 (-0.23 to 0.02) | 0.085 |
| Republic of Azerbaijan | 5151.56 (3834.26 to 6709.28) | 374.18 (278.5 to 487.32) | 11651.71 (8729.42 to 15170) | 363.59 (272.4 to 473.38) | -0.41 (-1.08 to 0.27) | 0.228 | 876.62 (562.01 to 1258.69) | 63.67 (40.82 to 91.42) | 1993.66 (1273.1 to 2887.32) | 62.21 (39.73 to 90.1) | -0.32 (-0.85 to 0.21) | 0.226 |
| Republic of Belarus | 11770.96 (8849.66 to 15335.43) | 392.67 (295.22 to 511.58) | 13244.83 (10005.11 to 17294.29) | 398.84 (301.28 to 520.78) | 0.16 (-0.24 to 0.57) | 0.424 | 2022.03 (1308.94 to 2897.07) | 67.45 (43.67 to 96.64) | 2274.01 (1456.72 to 3282.69) | 68.48 (43.87 to 98.85) | 0.15 (-0.19 to 0.49) | 0.369 |
| Republic of Benin | 1403.35 (1029.23 to 1869.95) | 248.6 (182.32 to 331.26) | 3933.35 (2873.34 to 5176.18) | 217.79 (159.1 to 286.61) | -0.41 (-0.46 to -0.35) | <0.001 | 252.21 (156.49 to 366.28) | 44.68 (27.72 to 64.89) | 708.14 (439.34 to 1019.31) | 39.21 (24.33 to 56.44) | -0.39 (-0.43 to -0.35) | <0.001 |
| Republic of Botswana | 526.08 (390.45 to 691.53) | 305.88 (227.02 to 402.07) | 1412.98 (1043.82 to 1855.52) | 269.12 (198.81 to 353.41) | -0.23 (-0.35 to -0.11) | 0.001 | 95.63 (60.69 to 138.81) | 55.6 (35.29 to 80.71) | 259.94 (163.47 to 374.85) | 49.51 (31.13 to 71.4) | -0.21 (-0.32 to -0.11) | <0.001 |
| Republic of Bulgaria | 10745.88 (8072.68 to 13965.36) | 379.98 (285.45 to 493.82) | 8876.16 (6615.66 to 11588.22) | 364.09 (271.37 to 475.34) | 0.06 (-0.04 to 0.17) | 0.229 | 1872.84 (1203.99 to 2679.59) | 66.22 (42.57 to 94.75) | 1547.65 (990.83 to 2217.09) | 63.48 (40.64 to 90.94) | 0.02 (-0.07 to 0.11) | 0.655 |
| Republic of Burundi | 2050.81 (1525.63 to 2695.36) | 302.08 (224.72 to 397.02) | 4848.26 (3590.13 to 6375.33) | 274.16 (203.01 to 360.51) | -0.05 (-0.27 to 0.16) | 0.622 | 378.54 (240.89 to 550.76) | 55.76 (35.48 to 81.13) | 886.57 (557.6 to 1279.85) | 50.13 (31.53 to 72.37) | -0.11 (-0.29 to 0.07) | 0.208 |
| Republic of Cabo Verde | 143.52 (106.35 to 187.67) | 320.98 (237.86 to 419.73) | 332.76 (245.06 to 441.53) | 252.6 (186.02 to 335.16) | -1.03 (-1.59 to -0.47) | 0.001 | 24.36 (15.44 to 35.35) | 54.49 (34.54 to 79.07) | 57.54 (36.04 to 84.37) | 43.68 (27.36 to 64.04) | -0.92 (-1.37 to -0.48) | <0.001 |
| Republic of Cameroon | 3441.8 (2516.03 to 4544.73) | 238.89 (174.63 to 315.44) | 9226.36 (6730.8 to 12324.37) | 202.43 (147.68 to 270.4) | -0.52 (-0.59 to -0.45) | <0.001 | 615.65 (383.9 to 900.44) | 42.73 (26.65 to 62.5) | 1677.76 (1036.87 to 2483.91) | 36.81 (22.75 to 54.5) | -0.47 (-0.53 to -0.41) | <0.001 |
| Republic of Chad | 1873.51 (1380.74 to 2468.36) | 245.82 (181.16 to 323.87) | 4242.89 (3079.88 to 5647.95) | 208.7 (151.5 to 277.82) | -0.52 (-0.57 to -0.47) | <0.001 | 337.54 (212.34 to 493.92) | 44.29 (27.86 to 64.81) | 768.87 (472.68 to 1119.81) | 37.82 (23.25 to 55.08) | -0.5 (-0.54 to -0.46) | <0.001 |
| Republic of Chile | 8354.64 (6171.32 to 11010.12) | 304.08 (224.61 to 400.73) | 19541.09 (14415.53 to 25525.59) | 340.17 (250.95 to 444.35) | 0.37 (0.19 to 0.56) | <0.001 | 1399.77 (873.07 to 2019.2) | 50.95 (31.78 to 73.49) | 3231.37 (2024.66 to 4693.26) | 56.25 (35.25 to 81.7) | 0.34 (0.18 to 0.51) | <0.001 |
| Republic of Colombia | 15822.67 (11668.37 to 20729.62) | 291.08 (214.66 to 381.36) | 46325.65 (34582.61 to 60647.37) | 342.76 (255.87 to 448.73) | 0.61 (0.47 to 0.75) | <0.001 | 2845.41 (1796.64 to 4131.94) | 52.35 (33.05 to 76.01) | 8067.59 (5099.61 to 11674.26) | 59.69 (37.73 to 86.38) | 0.5 (0.39 to 0.61) | <0.001 |
| Republic of Costa Rica | 1470.44 (1089.97 to 1936.07) | 300.61 (222.83 to 395.79) | 4633.18 (3472.82 to 6024.75) | 344.08 (257.91 to 447.42) | 0.61 (0.4 to 0.82) | <0.001 | 263.32 (167.32 to 382.24) | 53.83 (34.21 to 78.14) | 805.38 (506.89 to 1181.81) | 59.81 (37.64 to 87.77) | 0.49 (0.32 to 0.65) | <0.001 |
| Republic of Croatia | 6043.42 (4556.48 to 7858.72) | 389.25 (293.48 to 506.17) | 5930.08 (4488.02 to 7730.56) | 404.15 (305.87 to 526.86) | 0.24 (0.06 to 0.43) | 0.011 | 1042.82 (670.81 to 1498.21) | 67.17 (43.21 to 96.5) | 1011.83 (657.52 to 1451.93) | 68.96 (44.81 to 98.95) | 0.2 (0.05 to 0.35) | 0.009 |
| Republic of Cuba | 6646.62 (4896.76 to 8826.57) | 262.15 (193.13 to 348.13) | 12875.67 (9506.08 to 16970.1) | 311.31 (229.84 to 410.31) | 0.09 (-0.11 to 0.28) | 0.378 | 1155.24 (721.75 to 1678.34) | 45.56 (28.47 to 66.2) | 2184.47 (1373.15 to 3160.76) | 52.82 (33.2 to 76.42) | 0.03 (-0.14 to 0.2) | 0.701 |
| Republic of Cyprus | 543.76 (391.55 to 718.42) | 280.19 (201.76 to 370.2) | 1269.05 (917.71 to 1683.79) | 289.06 (209.03 to 383.53) | 0.25 (0.19 to 0.31) | <0.001 | 92.96 (56.1 to 137.64) | 47.9 (28.91 to 70.92) | 214.64 (130.74 to 319.37) | 48.89 (29.78 to 72.74) | 0.19 (0.14 to 0.25) | <0.001 |
| Population of Côte d ivoire | 3231.1 (2358.14 to 4306.49) | 212.78 (155.29 to 283.59) | 8583.85 (6181.35 to 11467.07) | 197.31 (142.09 to 263.59) | -0.24 (-0.3 to -0.19) | <0.001 | 590.01 (362.49 to 866.54) | 38.85 (23.87 to 57.06) | 1570.6 (951.88 to 2302.51) | 36.1 (21.88 to 52.93) | -0.23 (-0.27 to -0.2) | <0.001 |
| Republic of Djibouti | 150.64 (111.38 to 197.75) | 265.06 (195.97 to 347.95) | 697.47 (513.12 to 924.14) | 263.21 (193.64 to 348.75) | 0 (-0.03 to 0.04) | 0.868 | 28.22 (17.62 to 40.76) | 49.66 (31 to 71.71) | 129.4 (81.45 to 189.1) | 48.83 (30.74 to 71.36) | -0.03 (-0.06 to 0) | 0.035 |
| Republic of Ecuador | 3729.94 (2722.46 to 4963.27) | 237.87 (173.62 to 316.53) | 10771.19 (7889.24 to 14144.04) | 252.25 (184.76 to 331.24) | 0.3 (0.25 to 0.36) | <0.001 | 641.08 (394.87 to 942.11) | 40.88 (25.18 to 60.08) | 1817.41 (1124.93 to 2652.6) | 42.56 (26.34 to 62.12) | 0.23 (0.18 to 0.27) | <0.001 |
| Republic of El Salvador | 2433.87 (1786.3 to 3191.4) | 299.48 (219.8 to 392.69) | 4677.22 (3485.71 to 6130.19) | 315.5 (235.13 to 413.51) | 0.16 (0.14 to 0.18) | <0.001 | 436.49 (276.15 to 629.29) | 53.71 (33.98 to 77.43) | 832.71 (525.85 to 1207.46) | 56.17 (35.47 to 81.45) | 0.15 (0.13 to 0.16) | <0.001 |
| Republic of Equatorial Guinea | 219.61 (162.51 to 289.36) | 356.92 (264.11 to 470.28) | 572.71 (419.26 to 755.69) | 289.17 (211.69 to 381.56) | -0.66 (-0.71 to -0.61) | <0.001 | 40.83 (25.94 to 59.05) | 66.36 (42.16 to 95.97) | 109.47 (69 to 158.62) | 55.27 (34.84 to 80.09) | -0.57 (-0.61 to -0.52) | <0.001 |
| Republic of Estonia | 1707.89 (1282.02 to 2227.73) | 363.16 (272.6 to 473.7) | 1638.42 (1236.65 to 2134.72) | 381.25 (287.76 to 496.73) | 0.26 (0.17 to 0.35) | <0.001 | 295.56 (187.8 to 425.87) | 62.85 (39.93 to 90.56) | 280.25 (179.17 to 404.18) | 65.21 (41.69 to 94.05) | 0.21 (0.15 to 0.28) | <0.001 |
| Republic of Fiji | 365.77 (272.45 to 485.49) | 278.98 (207.8 to 370.29) | 800.27 (599.61 to 1050.62) | 335.13 (251.1 to 439.96) | 0.66 (0.57 to 0.75) | <0.001 | 68.62 (43.27 to 99.57) | 52.34 (33 to 75.94) | 144.06 (91.42 to 207.14) | 60.33 (38.28 to 86.74) | 0.51 (0.44 to 0.59) | <0.001 |
| Republic of Finland | 4418.04 (3207.19 to 5844.25) | 285.11 (206.97 to 377.15) | 5484.18 (3944.31 to 7237.26) | 316.65 (227.74 to 417.87) | 0.66 (0.53 to 0.79) | <0.001 | 742.63 (445.95 to 1116.88) | 47.92 (28.78 to 72.08) | 896.65 (538.91 to 1342.55) | 51.77 (31.12 to 77.52) | 0.51 (0.4 to 0.63) | <0.001 |
| Republic of Ghana | 4498.41 (3270.59 to 5990.98) | 216.83 (157.65 to 288.77) | 11823.84 (8556.98 to 15641.58) | 201.79 (146.04 to 266.95) | -0.18 (-0.26 to -0.11) | <0.001 | 809.85 (498.6 to 1184.26) | 39.04 (24.03 to 57.08) | 2142.28 (1318.26 to 3146.21) | 36.56 (22.5 to 53.69) | -0.16 (-0.22 to -0.1) | <0.001 |
| Republic of Guatemala | 3320.32 (2447.44 to 4372.6) | 297.17 (219.05 to 391.35) | 9301.48 (6926.09 to 12139.47) | 299.4 (222.94 to 390.75) | 0.21 (0.14 to 0.28) | <0.001 | 601.76 (378.02 to 878.34) | 53.86 (33.83 to 78.61) | 1675.58 (1051.97 to 2416.03) | 53.93 (33.86 to 77.77) | 0.17 (0.11 to 0.22) | <0.001 |
| Republic of Guinea | 2308.59 (1694.23 to 3046.01) | 253.77 (186.24 to 334.84) | 3959.17 (2876.47 to 5233.97) | 220.99 (160.56 to 292.15) | -0.41 (-0.43 to -0.39) | <0.001 | 412.05 (257.24 to 600.42) | 45.3 (28.28 to 66) | 713.9 (439.63 to 1036.08) | 39.85 (24.54 to 57.83) | -0.4 (-0.41 to -0.39) | <0.001 |
| Republic of Guinea-Bissau | 278.48 (200.93 to 369.13) | 222.74 (160.71 to 295.25) | 540.02 (390.77 to 721.58) | 195.95 (141.79 to 261.83) | -0.35 (-0.37 to -0.32) | <0.001 | 50.32 (30.64 to 74.1) | 40.25 (24.51 to 59.27) | 98.94 (60.22 to 144.39) | 35.9 (21.85 to 52.39) | -0.31 (-0.33 to -0.29) | <0.001 |
| Republic of Guyana | 281.18 (205.53 to 375.68) | 242.12 (176.97 to 323.49) | 531.99 (391.72 to 705.74) | 279.03 (205.46 to 370.17) | 0.65 (0.45 to 0.86) | <0.001 | 51.2 (31.91 to 74.66) | 44.08 (27.47 to 64.29) | 94.24 (58.59 to 137.86) | 49.43 (30.73 to 72.31) | 0.52 (0.36 to 0.69) | <0.001 |
| Republic of Haiti | 2728.37 (1990.71 to 3609.68) | 273.71 (199.71 to 362.13) | 5996.99 (4375.38 to 7946.73) | 238.37 (173.91 to 315.87) | -0.34 (-0.43 to -0.26) | <0.001 | 489.12 (303.19 to 715.62) | 49.07 (30.42 to 71.79) | 1087.46 (672.85 to 1593.66) | 43.22 (26.74 to 63.34) | -0.33 (-0.4 to -0.26) | <0.001 |
| Republic of Honduras | 1828.07 (1357.89 to 2395.37) | 296.48 (220.22 to 388.48) | 5628.75 (4173.04 to 7400.29) | 291.72 (216.28 to 383.54) | 0.09 (0.03 to 0.15) | 0.005 | 332.18 (210.59 to 480.62) | 53.87 (34.15 to 77.95) | 1021.81 (644.79 to 1475.62) | 52.96 (33.42 to 76.48) | 0.07 (0.02 to 0.11) | 0.006 |
| Republic of Iceland | 185.55 (136.29 to 245.64) | 311.33 (228.67 to 412.15) | 346.44 (251.44 to 461.33) | 320.93 (232.93 to 427.36) | 0.37 (0.16 to 0.58) | 0.001 | 31.99 (19.75 to 47.17) | 53.67 (33.14 to 79.14) | 58.33 (36.01 to 86.32) | 54.03 (33.36 to 79.96) | 0.22 (0.04 to 0.41) | 0.022 |
| Republic of India | 349695.68 (252492.86 to 464236.9) | 227.3 (164.12 to 301.75) | 807167.08 (583394.15 to 1070892.16) | 236.6 (171.01 to 313.91) | -0.02 (-0.11 to 0.08) | 0.741 | 61833.02 (38774.18 to 89692.34) | 40.19 (25.2 to 58.3) | 139069.24 (86240.12 to 203856.63) | 40.76 (25.28 to 59.76) | -0.06 (-0.14 to 0.02) | 0.113 |
| Republic of Indonesia | 122338.83 (90817.87 to 161500.17) | 376.95 (279.83 to 497.62) | 304547.75 (225787.95 to 401767.41) | 379.38 (281.26 to 500.48) | -0.05 (-0.21 to 0.1) | 0.482 | 21614.98 (13981.74 to 30730.24) | 66.6 (43.08 to 94.69) | 53508.47 (34600.19 to 76134.08) | 66.66 (43.1 to 94.84) | -0.05 (-0.17 to 0.06) | 0.371 |
| Republic of Iraq | 7623.69 (5640.16 to 9996.29) | 320.83 (237.36 to 420.68) | 25923.65 (19116.33 to 34183.69) | 299.87 (221.13 to 395.42) | -0.22 (-0.26 to -0.18) | <0.001 | 1475.89 (940.19 to 2116.09) | 62.11 (39.57 to 89.05) | 5009.82 (3177.9 to 7206.64) | 57.95 (36.76 to 83.36) | -0.23 (-0.27 to -0.19) | <0.001 |
| Republic of Italy | 72288.38 (53891.02 to 94326.75) | 408.93 (304.86 to 533.6) | 81289.79 (59360.82 to 107102.2) | 363.04 (265.1 to 478.31) | -0.49 (-0.57 to -0.41) | <0.001 | 12180.72 (7750.42 to 17498.3) | 68.9 (43.84 to 98.99) | 13034.33 (8019.89 to 19404.93) | 58.21 (35.82 to 86.66) | -0.61 (-0.71 to -0.51) | <0.001 |
| Republic of Kazakhstan | 11909.56 (8973.4 to 15568.45) | 343.24 (258.62 to 448.69) | 17107.5 (12790.63 to 22502.61) | 331.72 (248.02 to 436.34) | -0.06 (-0.38 to 0.26) | 0.698 | 2072.41 (1324.44 to 2980.91) | 59.73 (38.17 to 85.91) | 2991.02 (1906.18 to 4344.24) | 58 (36.96 to 84.24) | -0.04 (-0.29 to 0.21) | 0.735 |
| Republic of Kenya | 8292.74 (6104.83 to 10988.42) | 318.5 (234.47 to 422.03) | 23811.1 (17408.74 to 31612) | 294.3 (215.17 to 390.72) | -0.2 (-0.25 to -0.15) | <0.001 | 1455.31 (927.65 to 2095.12) | 55.89 (35.63 to 80.47) | 4182.1 (2658.92 to 6035.78) | 51.69 (32.86 to 74.6) | -0.22 (-0.25 to -0.18) | <0.001 |
| Republic of Kiribati | 35.57 (26.32 to 47.07) | 303.76 (224.77 to 401.89) | 82.02 (61.43 to 107.98) | 330.87 (247.81 to 435.58) | 0.3 (0.11 to 0.5) | 0.003 | 6.6 (4.21 to 9.44) | 56.34 (35.92 to 80.62) | 15.02 (9.59 to 21.39) | 60.58 (38.69 to 86.28) | 0.26 (0.11 to 0.41) | 0.001 |
| Republic of Korea | 30860.76 (22718.22 to 40175.25) | 319.78 (235.4 to 416.29) | 94177.86 (70796.12 to 122227.61) | 448.5 (337.15 to 582.09) | 0.84 (0.61 to 1.07) | <0.001 | 5579.07 (3542.93 to 7918.78) | 57.81 (36.71 to 82.05) | 17057.2 (11209.25 to 24063.52) | 81.23 (53.38 to 114.6) | 0.8 (0.6 to 1) | <0.001 |
| Republic of Latvia | 2989.43 (2228.83 to 3900.54) | 365.52 (272.52 to 476.92) | 2550.96 (1931.66 to 3325.58) | 397.2 (300.77 to 517.82) | 0.21 (0.09 to 0.33) | 0.001 | 519.96 (332.34 to 748.84) | 63.58 (40.64 to 91.56) | 437.85 (283.6 to 631.35) | 68.18 (44.16 to 98.31) | 0.19 (0.09 to 0.29) | <0.001 |
| Republic of Liberia | 730.74 (538.05 to 962.54) | 235.27 (173.23 to 309.9) | 1587.18 (1142.55 to 2136.57) | 178.33 (128.37 to 240.05) | -0.96 (-1.02 to -0.91) | <0.001 | 130.57 (81.95 to 189.16) | 42.04 (26.38 to 60.9) | 296.43 (178.98 to 437.58) | 33.31 (20.11 to 49.16) | -0.82 (-0.86 to -0.77) | <0.001 |
| Republic of Lithuania | 3810.73 (2865.33 to 4947.34) | 361 (271.44 to 468.68) | 3882.84 (2932.03 to 5044.08) | 405.13 (305.92 to 526.29) | 0.19 (-0.02 to 0.41) | 0.079 | 661.59 (429.88 to 952.94) | 62.67 (40.72 to 90.28) | 663.07 (426.3 to 949.2) | 69.18 (44.48 to 99.04) | 0.18 (0 to 0.36) | 0.052 |
| Republic of Madagascar | 4710.39 (3522.98 to 6196.92) | 305.64 (228.59 to 402.09) | 11641.57 (8592.77 to 15502.98) | 263.03 (194.15 to 350.28) | -0.39 (-0.58 to -0.2) | <0.001 | 859.33 (551.14 to 1230.81) | 55.76 (35.76 to 79.86) | 2143.33 (1352.29 to 3115.57) | 48.43 (30.55 to 70.39) | -0.36 (-0.52 to -0.2) | <0.001 |
| Republic of Malawi | 3627.42 (2692.66 to 4771.82) | 293.66 (217.98 to 386.3) | 6794.99 (4993.75 to 8991.82) | 261.13 (191.91 to 345.55) | -0.24 (-0.33 to -0.15) | <0.001 | 669.11 (425.58 to 970.19) | 54.17 (34.45 to 78.54) | 1268.01 (800.13 to 1850.9) | 48.73 (30.75 to 71.13) | -0.22 (-0.29 to -0.14) | <0.001 |
| Republic of Maldives | 119.34 (89.59 to 156.23) | 393.91 (295.7 to 515.63) | 420.46 (313.76 to 553.68) | 311.98 (232.81 to 410.83) | -1.03 (-1.23 to -0.82) | <0.001 | 21.55 (13.86 to 30.57) | 71.12 (45.74 to 100.89) | 78.7 (50.59 to 113.28) | 58.4 (37.54 to 84.05) | -0.83 (-1 to -0.67) | <0.001 |
| Republic of Mali | 3110.43 (2293.32 to 4089.78) | 247.21 (182.27 to 325.05) | 6712.05 (4898.99 to 8861.65) | 224.62 (163.94 to 296.55) | -0.33 (-0.36 to -0.31) | <0.001 | 554.28 (349.88 to 808.3) | 44.05 (27.81 to 64.24) | 1206.18 (745.85 to 1758.19) | 40.36 (24.96 to 58.84) | -0.3 (-0.32 to -0.28) | <0.001 |
| Republic of Malta | 287.45 (205.85 to 380.49) | 271.04 (194.1 to 358.77) | 445.49 (317.36 to 592.53) | 308.77 (219.96 to 410.68) | 0.8 (0.62 to 0.97) | <0.001 | 48.84 (29.65 to 72.61) | 46.05 (27.96 to 68.46) | 73.62 (44.31 to 110.95) | 51.02 (30.71 to 76.9) | 0.65 (0.49 to 0.82) | <0.001 |
| Republic of Mauritius | 762.32 (569.49 to 998.48) | 360.99 (269.68 to 472.82) | 1944.78 (1479.74 to 2538.42) | 437.18 (332.64 to 570.62) | 1.12 (0.81 to 1.43) | <0.001 | 138.75 (90.01 to 197.58) | 65.7 (42.62 to 93.56) | 342.82 (221.13 to 490.03) | 77.06 (49.71 to 110.16) | 0.94 (0.69 to 1.19) | <0.001 |
| Republic of Moldova | 3897.6 (2910.67 to 5087.93) | 352.75 (263.43 to 460.48) | 4722.5 (3545.31 to 6129.33) | 370.07 (277.82 to 480.31) | 0.46 (0.16 to 0.76) | 0.004 | 676.99 (440.04 to 974.7) | 61.27 (39.83 to 88.21) | 814.57 (521.45 to 1176.46) | 63.83 (40.86 to 92.19) | 0.4 (0.16 to 0.64) | 0.002 |
| Republic of Mozambique | 5875.9 (4364.89 to 7710.33) | 296.05 (219.92 to 388.48) | 10577.27 (7808.51 to 13994.07) | 263.38 (194.44 to 348.46) | -0.36 (-0.38 to -0.34) | <0.001 | 1083.66 (685.53 to 1562.98) | 54.6 (34.54 to 78.75) | 1965.78 (1248.48 to 2833.9) | 48.95 (31.09 to 70.57) | -0.33 (-0.35 to -0.31) | <0.001 |
| Republic of Namibia | 594.15 (443.47 to 779.12) | 306.12 (228.49 to 401.42) | 1281.55 (947.47 to 1678.01) | 278.96 (206.24 to 365.26) | -0.26 (-0.3 to -0.21) | <0.001 | 107.82 (68.73 to 155.69) | 55.55 (35.41 to 80.21) | 233.69 (148.32 to 340.46) | 50.87 (32.28 to 74.11) | -0.24 (-0.28 to -0.2) | <0.001 |
| Republic of Nauru | 4.59 (3.36 to 6.02) | 280.17 (205.03 to 367.88) | 5.68 (4.21 to 7.49) | 285.09 (211.1 to 375.81) | 0.28 (0.17 to 0.4) | <0.001 | 0.86 (0.55 to 1.24) | 52.77 (33.33 to 75.92) | 1.06 (0.68 to 1.53) | 53.39 (33.9 to 76.58) | 0.22 (0.13 to 0.31) | <0.001 |
| Republic of Nicaragua | 1377.18 (1021.12 to 1811.12) | 290.76 (215.59 to 382.38) | 4392.26 (3278.19 to 5783.19) | 302.56 (225.82 to 398.37) | 0.23 (0.13 to 0.33) | <0.001 | 249.14 (155.88 to 362.43) | 52.6 (32.91 to 76.52) | 786.47 (502.01 to 1140.98) | 54.18 (34.58 to 78.6) | 0.19 (0.11 to 0.27) | <0.001 |
| Republic of Niue | 1.65 (1.22 to 2.17) | 336.56 (248.02 to 442.31) | 1.98 (1.48 to 2.58) | 371.73 (278.96 to 486.13) | 0.21 (0.15 to 0.27) | <0.001 | 0.3 (0.19 to 0.43) | 60.33 (38.05 to 86.79) | 0.35 (0.22 to 0.5) | 65.18 (41.69 to 93.65) | 0.16 (0.11 to 0.2) | <0.001 |
| Republic of Palau | 8.57 (6.33 to 11.33) | 297.65 (219.88 to 393.56) | 24.88 (18.4 to 32.66) | 341.02 (252.23 to 447.66) | 0.85 (0.54 to 1.15) | <0.001 | 1.57 (1 to 2.29) | 54.43 (34.69 to 79.43) | 4.44 (2.84 to 6.35) | 60.81 (38.89 to 87.02) | 0.69 (0.45 to 0.93) | <0.001 |
| Republic of Panama | 1218.76 (899.37 to 1610.63) | 290.22 (214.16 to 383.53) | 3518.5 (2606.67 to 4604.67) | 320.14 (237.17 to 418.97) | 0.29 (0.23 to 0.35) | <0.001 | 218.52 (138.24 to 317.68) | 52.04 (32.92 to 75.65) | 616.81 (389.67 to 893.61) | 56.12 (35.46 to 81.31) | 0.22 (0.17 to 0.27) | <0.001 |
| Republic of Paraguay | 2288.29 (1718.7 to 3001.13) | 366.27 (275.1 to 480.37) | 6031.73 (4581.71 to 7843.54) | 372.21 (282.73 to 484.01) | 0.23 (0.1 to 0.35) | 0.001 | 419.69 (269.87 to 606.74) | 67.18 (43.2 to 97.12) | 1090.37 (707.37 to 1559.09) | 67.29 (43.65 to 96.21) | 0.15 (0.05 to 0.25) | 0.005 |
| Republic of Peru | 8437.21 (6181.7 to 11175.83) | 239.4 (175.4 to 317.11) | 22079.92 (16190.12 to 29266.44) | 244.67 (179.41 to 324.31) | 0.07 (0.03 to 0.12) | 0.003 | 1423.61 (877.8 to 2093.15) | 40.39 (24.91 to 59.39) | 3676.13 (2268.1 to 5370.44) | 40.74 (25.13 to 59.51) | 0.03 (0 to 0.07) | 0.04 |
| Republic of Poland | 39952.89 (29951.3 to 52227.1) | 388.92 (291.56 to 508.4) | 51448.06 (38612.97 to 67141.05) | 393.3 (295.18 to 513.26) | 0.66 (0.38 to 0.95) | <0.001 | 6766.44 (4346.48 to 9667.23) | 65.87 (42.31 to 94.1) | 8634.12 (5547.86 to 12374.19) | 66 (42.41 to 94.59) | 0.51 (0.28 to 0.73) | <0.001 |
| Republic of Rwanda | 2663.43 (1982.91 to 3481.26) | 312.92 (232.97 to 409) | 6372.18 (4717.54 to 8396.72) | 292.7 (216.7 to 385.69) | 0.25 (0 to 0.5) | 0.052 | 484.87 (310.97 to 696.18) | 56.97 (36.53 to 81.79) | 1169.1 (735.31 to 1682.85) | 53.7 (33.78 to 77.3) | 0.21 (0 to 0.41) | 0.05 |
| Republic of San Marino | 22.13 (16.14 to 29.25) | 322.12 (234.9 to 425.65) | 36.96 (26.35 to 49.15) | 299.78 (213.77 to 398.71) | -0.45 (-0.57 to -0.34) | <0.001 | 3.71 (2.29 to 5.51) | 53.98 (33.26 to 80.13) | 6.09 (3.65 to 9.14) | 49.39 (29.61 to 74.15) | -0.48 (-0.57 to -0.39) | <0.001 |
| Republic of Senegal | 2317.3 (1692.2 to 3079.23) | 238.71 (174.32 to 317.19) | 5572.34 (4049.36 to 7393.8) | 225.74 (164.04 to 299.53) | -0.14 (-0.19 to -0.08) | <0.001 | 415.05 (260.36 to 604.72) | 42.75 (26.82 to 62.29) | 990.95 (603.39 to 1456.93) | 40.14 (24.44 to 59.02) | -0.18 (-0.22 to -0.14) | <0.001 |
| Republic of Serbia | 11101.99 (8368 to 14600.58) | 376.68 (283.92 to 495.38) | 11253.57 (8435.91 to 14626.16) | 378.93 (284.05 to 492.49) | 0.25 (0.12 to 0.38) | <0.001 | 1915.38 (1225.84 to 2775) | 64.99 (41.59 to 94.15) | 1946.26 (1239.37 to 2788.03) | 65.53 (41.73 to 93.88) | 0.22 (0.11 to 0.33) | <0.001 |
| Republic of Seychelles | 51.81 (38.99 to 67.46) | 405.53 (305.22 to 528.05) | 133 (98.84 to 173.03) | 384.7 (285.91 to 500.49) | -0.28 (-0.67 to 0.11) | 0.149 | 9.28 (6.06 to 13.3) | 72.66 (47.41 to 104.07) | 23.94 (15.35 to 34.16) | 69.25 (44.4 to 98.81) | -0.24 (-0.56 to 0.08) | 0.14 |
| Republic of Sierra Leone | 1349.17 (990.23 to 1784.09) | 235.99 (173.21 to 312.07) | 2678.12 (1951.64 to 3571.79) | 208.38 (151.85 to 277.92) | -0.47 (-0.53 to -0.41) | <0.001 | 244.08 (152.9 to 352.81) | 42.69 (26.74 to 61.71) | 488.31 (300.32 to 714.37) | 37.99 (23.37 to 55.58) | -0.43 (-0.48 to -0.39) | <0.001 |
| Republic of Singapore | 1659.66 (1231.92 to 2158.98) | 230.66 (171.22 to 300.06) | 5987.7 (4509.82 to 7729.57) | 269.33 (202.85 to 347.68) | 0.7 (0.45 to 0.96) | <0.001 | 311.41 (204.3 to 438.15) | 43.28 (28.39 to 60.9) | 1069.34 (712.87 to 1491.56) | 48.1 (32.07 to 67.09) | 0.51 (0.28 to 0.75) | <0.001 |
| Republic of Slovenia | 2085.22 (1566.83 to 2694.7) | 360.61 (270.96 to 466.01) | 2894.17 (2191.04 to 3744.54) | 385.37 (291.75 to 498.6) | 0.46 (0.28 to 0.63) | <0.001 | 359.23 (228.53 to 516.45) | 62.12 (39.52 to 89.31) | 492.78 (322.71 to 709.69) | 65.62 (42.97 to 94.5) | 0.4 (0.26 to 0.54) | <0.001 |
| Republic of South Africa | 20148.61 (14823.58 to 26665.13) | 333.45 (245.33 to 441.3) | 45925.5 (33792.3 to 61143.37) | 334.33 (246 to 445.11) | 0.19 (0.04 to 0.35) | 0.017 | 3508.86 (2239.53 to 5083.31) | 58.07 (37.06 to 84.13) | 7857.21 (5015.56 to 11369.11) | 57.2 (36.51 to 82.76) | 0.11 (-0.02 to 0.23) | 0.087 |
| Republic of South Sudan | 2163.57 (1597.47 to 2846.49) | 295.54 (218.21 to 388.82) | 4083.38 (2984.03 to 5399.81) | 265.75 (194.2 to 351.42) | -0.49 (-0.62 to -0.36) | <0.001 | 395.77 (251.39 to 575.51) | 54.06 (34.34 to 78.61) | 755.73 (476.36 to 1096.16) | 49.18 (31 to 71.34) | -0.42 (-0.52 to -0.31) | <0.001 |
| Republic of Sudan | 8889.35 (6563.8 to 11742.73) | 322.14 (237.87 to 425.55) | 19945.88 (14763.31 to 26374.76) | 286.79 (212.27 to 379.22) | -0.49 (-0.54 to -0.44) | <0.001 | 1721.2 (1082.81 to 2465.84) | 62.37 (39.24 to 89.36) | 3876.24 (2449.48 to 5585.67) | 55.73 (35.22 to 80.31) | -0.46 (-0.51 to -0.42) | <0.001 |
| Republic of Suriname | 219.79 (162.97 to 286.35) | 297.04 (220.24 to 386.99) | 520.05 (383.48 to 682.85) | 306.81 (226.24 to 402.85) | -0.15 (-0.43 to 0.13) | 0.27 | 39.03 (24.66 to 56.5) | 52.75 (33.32 to 76.36) | 90.64 (57 to 131.26) | 53.47 (33.63 to 77.44) | -0.17 (-0.4 to 0.05) | 0.129 |
| Republic of Tajikistan | 2636.13 (1971.16 to 3435.64) | 361.47 (270.29 to 471.1) | 6469.67 (4806.96 to 8492.86) | 317.65 (236.01 to 416.98) | -0.58 (-1.13 to -0.02) | 0.042 | 451.21 (287.68 to 653.13) | 61.87 (39.45 to 89.56) | 1123.87 (712.71 to 1641.95) | 55.18 (34.99 to 80.62) | -0.48 (-0.91 to -0.04) | 0.032 |
| Republic of Trinidad and Tobago | 580.4 (423.74 to 768.66) | 260.32 (190.06 to 344.76) | 1415.38 (1066.22 to 1854.48) | 318.22 (239.72 to 416.94) | 0.94 (0.76 to 1.12) | <0.001 | 104.37 (65.48 to 152.6) | 46.81 (29.37 to 68.45) | 244.4 (154.7 to 355.27) | 54.95 (34.78 to 79.87) | 0.76 (0.62 to 0.91) | <0.001 |
| Republic of Tunisia | 5843.94 (4393.61 to 7660.68) | 415.92 (312.7 to 545.22) | 14076.45 (10594.43 to 18431.37) | 391.57 (294.71 to 512.71) | -0.13 (-0.42 to 0.17) | 0.386 | 1094.12 (705.37 to 1561.89) | 77.87 (50.2 to 111.16) | 2617.87 (1675.19 to 3736.87) | 72.82 (46.6 to 103.95) | -0.16 (-0.41 to 0.09) | 0.212 |
| Republic of Turkey | 43397.8 (32239.91 to 56470.38) | 410.95 (305.29 to 534.74) | 98392.49 (74337.17 to 128602.6) | 392.59 (296.61 to 513.13) | -0.05 (-0.23 to 0.12) | 0.529 | 8176.16 (5201.72 to 11640.88) | 77.42 (49.26 to 110.23) | 18437.99 (11770.34 to 26380.08) | 73.57 (46.96 to 105.26) | -0.09 (-0.24 to 0.06) | 0.226 |
| Republic of Uganda | 5853.09 (4345.9 to 7703.44) | 296.96 (220.5 to 390.84) | 14229.36 (10421.27 to 18889.58) | 270.16 (197.86 to 358.63) | -0.47 (-0.56 to -0.38) | <0.001 | 1073.39 (684.17 to 1551.26) | 54.46 (34.71 to 78.71) | 2630.75 (1659.54 to 3832.12) | 49.95 (31.51 to 72.76) | -0.4 (-0.47 to -0.33) | <0.001 |
| Republic of Uzbekistan | 10227.07 (7556.6 to 13412.4) | 346.4 (255.95 to 454.3) | 26771.48 (19816.66 to 35261.91) | 311.58 (230.63 to 410.39) | -0.24 (-0.72 to 0.24) | 0.317 | 1747.81 (1104.91 to 2537.01) | 59.2 (37.42 to 85.93) | 4673.04 (2959.46 to 6741.92) | 54.39 (34.44 to 78.47) | -0.17 (-0.54 to 0.21) | 0.374 |
| Republic of Vanuatu | 58.92 (42.91 to 78.21) | 277.64 (202.22 to 368.56) | 166.25 (122.68 to 218.76) | 279.06 (205.93 to 367.21) | 0.18 (0.12 to 0.24) | <0.001 | 10.91 (6.79 to 15.9) | 51.43 (31.99 to 74.95) | 30.62 (19.31 to 44.35) | 51.4 (32.41 to 74.44) | 0.13 (0.08 to 0.17) | <0.001 |
| Republic of Yemen | 5579.45 (4124.17 to 7289.55) | 349.98 (258.7 to 457.26) | 14791.67 (10985 to 19514.56) | 291.17 (216.24 to 384.14) | -0.4 (-0.47 to -0.33) | <0.001 | 1076.54 (691.56 to 1552.35) | 67.53 (43.38 to 97.38) | 2899.28 (1857.56 to 4169.82) | 57.07 (36.57 to 82.08) | -0.38 (-0.44 to -0.32) | <0.001 |
| Republic of Zambia | 2750.29 (2029.38 to 3647.6) | 289.16 (213.37 to 383.51) | 6785.34 (4983.12 to 9020.07) | 251.66 (184.82 to 334.55) | -0.55 (-0.58 to -0.52) | <0.001 | 507.12 (315.54 to 731.56) | 53.32 (33.18 to 76.92) | 1275.01 (799.04 to 1844.63) | 47.29 (29.64 to 68.42) | -0.46 (-0.48 to -0.44) | <0.001 |
| Republic of Zimbabwe | 3664.87 (2718.39 to 4843.67) | 293.43 (217.65 to 387.81) | 6468.37 (4783.08 to 8546.78) | 261.91 (193.67 to 346.06) | -0.1 (-0.2 to -0.01) | 0.04 | 666.62 (421.85 to 967.59) | 53.37 (33.78 to 77.47) | 1198.41 (746.78 to 1752.63) | 48.52 (30.24 to 70.96) | -0.08 (-0.17 to 0) | 0.05 |
| Republic of the Congo | 1139.47 (842.89 to 1498.19) | 351.96 (260.35 to 462.77) | 3149.64 (2332.16 to 4126.44) | 293.21 (217.11 to 384.15) | -0.65 (-0.77 to -0.53) | <0.001 | 211.98 (135.77 to 307.93) | 65.48 (41.94 to 95.11) | 590.77 (375.79 to 836.32) | 55 (34.98 to 77.86) | -0.63 (-0.73 to -0.52) | <0.001 |
| Republic of the Gambia | 253.13 (182.36 to 335.84) | 215.12 (154.98 to 285.42) | 667.49 (481.66 to 883.29) | 203.22 (146.64 to 268.92) | -0.3 (-0.37 to -0.23) | <0.001 | 46.23 (28.96 to 67.88) | 39.29 (24.61 to 57.69) | 121.57 (73.51 to 177.88) | 37.01 (22.38 to 54.16) | -0.29 (-0.35 to -0.24) | <0.001 |
| Republic of the Marshall Islands | 13.2 (9.7 to 17.55) | 259.42 (190.67 to 344.76) | 35.66 (26.38 to 47.13) | 278.33 (205.91 to 367.86) | 0.78 (0.57 to 0.99) | <0.001 | 2.46 (1.56 to 3.59) | 48.41 (30.72 to 70.59) | 6.58 (4.18 to 9.51) | 51.35 (32.61 to 74.21) | 0.64 (0.47 to 0.8) | <0.001 |
| Republic of the Niger | 2210.74 (1616.49 to 2925.14) | 221.77 (162.16 to 293.43) | 6512.84 (4789.39 to 8594.86) | 233.92 (172.02 to 308.7) | 0.15 (0.09 to 0.22) | <0.001 | 402.46 (250.92 to 589.43) | 40.37 (25.17 to 59.13) | 1159.97 (719.58 to 1699.77) | 41.66 (25.84 to 61.05) | 0.09 (0.04 to 0.14) | 0.001 |
| Republic of the Philippines | 35516.58 (26487.36 to 46716.06) | 364.57 (271.89 to 479.53) | 96726.29 (71976.84 to 127740.13) | 374.48 (278.66 to 494.55) | 0.12 (0.01 to 0.23) | 0.03 | 6427.47 (4150.7 to 9130.22) | 65.98 (42.61 to 93.72) | 17150.88 (11117.72 to 24541.38) | 66.4 (43.04 to 95.01) | 0.04 (-0.03 to 0.12) | 0.261 |
| Republic of the Union of Myanmar | 27582.26 (20702.61 to 35979.66) | 406.44 (305.06 to 530.18) | 55114.57 (41560.13 to 71993.5) | 379.71 (286.33 to 496) | -0.14 (-0.38 to 0.11) | 0.268 | 5042.41 (3248.99 to 7172.93) | 74.3 (47.88 to 105.7) | 10053.24 (6456.09 to 14366.81) | 69.26 (44.48 to 98.98) | -0.16 (-0.36 to 0.05) | 0.125 |
| Romania | 25031.46 (18680.78 to 32559.88) | 371.92 (277.56 to 483.77) | 23721.15 (17821.33 to 30784.98) | 351.25 (263.89 to 455.85) | 0.06 (-0.05 to 0.17) | 0.288 | 4336.13 (2770.16 to 6264.79) | 64.43 (41.16 to 93.08) | 4146.83 (2698.25 to 5945.91) | 61.4 (39.95 to 88.04) | 0.04 (-0.05 to 0.12) | 0.382 |
| Russian Federation | 174051.77 (129622.29 to 227910.88) | 401.77 (299.21 to 526.09) | 199539.43 (149158.34 to 261127.01) | 402.67 (301 to 526.96) | 0.35 (0.01 to 0.7) | 0.046 | 28945.18 (18646.52 to 41279.06) | 66.81 (43.04 to 95.29) | 33205.8 (21439.2 to 47394.45) | 67.01 (43.26 to 95.64) | 0.29 (0.02 to 0.55) | 0.034 |
| Saint Kitts and Nevis | 19.28 (14.38 to 24.95) | 316.78 (236.19 to 409.92) | 61.97 (45.64 to 81.32) | 299.44 (220.56 to 392.98) | 0.25 (-0.27 to 0.77) | 0.333 | 3.3 (2.06 to 4.8) | 54.29 (33.82 to 78.85) | 10.66 (6.69 to 15.38) | 51.53 (32.32 to 74.35) | 0.19 (-0.22 to 0.61) | 0.357 |
| Saint Lucia | 58.01 (42.76 to 76.7) | 280.72 (206.92 to 371.17) | 184.44 (136.18 to 241.96) | 301.57 (222.65 to 395.61) | 0.15 (-0.04 to 0.33) | 0.115 | 10.24 (6.5 to 15.01) | 49.56 (31.46 to 72.66) | 31.97 (20.31 to 46.72) | 52.28 (33.21 to 76.39) | 0.09 (-0.06 to 0.24) | 0.212 |
| Saint Vincent and the Grenadines | 48.12 (35.96 to 63.25) | 304.32 (227.41 to 400.03) | 109.42 (81.12 to 143.68) | 310.58 (230.25 to 407.83) | 0.12 (-0.19 to 0.43) | 0.428 | 8.41 (5.26 to 12.22) | 53.21 (33.26 to 77.29) | 18.94 (11.79 to 27.71) | 53.77 (33.46 to 78.65) | 0.07 (-0.18 to 0.33) | 0.565 |
| Slovak Republic | 4760.65 (3587.68 to 6234.65) | 349.77 (263.59 to 458.06) | 6869.48 (5150.35 to 8961.89) | 356.92 (267.6 to 465.63) | 0.59 (0.38 to 0.79) | <0.001 | 831.63 (531.22 to 1202.28) | 61.1 (39.03 to 88.33) | 1190.28 (758.53 to 1714.67) | 61.84 (39.41 to 89.09) | 0.46 (0.3 to 0.63) | <0.001 |
| Socialist Republic of Viet Nam | 42576.24 (32156.57 to 55551.8) | 431.66 (326.02 to 563.21) | 112240.69 (84554.14 to 146403.75) | 381.17 (287.14 to 497.18) | -0.33 (-0.8 to 0.14) | 0.159 | 7566.89 (4880.14 to 10830.5) | 76.72 (49.48 to 109.81) | 20175.14 (12972.42 to 28658.72) | 68.51 (44.05 to 97.32) | -0.29 (-0.67 to 0.09) | 0.124 |
| Solomon Islands | 144.2 (106.92 to 189.9) | 313.01 (232.09 to 412.2) | 350.31 (257.35 to 461.08) | 274.91 (201.96 to 361.84) | -0.6 (-0.65 to -0.54) | <0.001 | 26.49 (17 to 38.06) | 57.51 (36.9 to 82.63) | 65.97 (41.76 to 94.49) | 51.77 (32.77 to 74.15) | -0.48 (-0.53 to -0.43) | <0.001 |
| State of Eritrea | 1277.88 (943.65 to 1684.65) | 277.64 (205.02 to 366.01) | 2859.06 (2091.71 to 3794.24) | 258.97 (189.47 to 343.68) | -0.34 (-0.4 to -0.29) | <0.001 | 237.8 (149.96 to 342.68) | 51.67 (32.58 to 74.45) | 532.66 (332.74 to 775.58) | 48.25 (30.14 to 70.25) | -0.3 (-0.34 to -0.26) | <0.001 |
| State of Israel | 3022.49 (2207.13 to 3953.17) | 287.43 (209.89 to 375.93) | 6698.94 (4836.88 to 8899.52) | 273.85 (197.73 to 363.81) | 0.24 (0.08 to 0.4) | 0.005 | 514.07 (314.45 to 759.86) | 48.89 (29.9 to 72.26) | 1128.72 (689.99 to 1673.87) | 46.14 (28.21 to 68.43) | 0.13 (-0.01 to 0.27) | 0.06 |
| State of Kuwait | 763.31 (560.66 to 1006.9) | 267.88 (196.76 to 353.36) | 3950.8 (2920.35 to 5204.26) | 261.47 (193.28 to 344.43) | -0.36 (-0.61 to -0.11) | 0.006 | 150.47 (94.96 to 217.3) | 52.81 (33.33 to 76.26) | 774.1 (487.15 to 1120.69) | 51.23 (32.24 to 74.17) | -0.33 (-0.54 to -0.12) | 0.003 |
| State of Libya | 2073.92 (1542.17 to 2726.89) | 354.98 (263.96 to 466.74) | 5925.53 (4343.59 to 7786.28) | 292.06 (214.09 to 383.77) | -1.01 (-1.17 to -0.85) | <0.001 | 394.72 (253.62 to 562.91) | 67.56 (43.41 to 96.35) | 1146.32 (726.15 to 1643.29) | 56.5 (35.79 to 80.99) | -0.89 (-1.02 to -0.76) | <0.001 |
| State of Qatar | 156.05 (112.73 to 209.22) | 198.42 (143.34 to 266.03) | 1599.76 (1150.89 to 2139.37) | 201.58 (145.02 to 269.58) | 0.17 (0 to 0.33) | 0.046 | 32.17 (20.12 to 47.14) | 40.91 (25.58 to 59.94) | 326.51 (200.28 to 474.36) | 41.14 (25.24 to 59.77) | 0.11 (-0.02 to 0.24) | 0.101 |
| Sultanate of Oman | 717.63 (525.48 to 956.81) | 266.83 (195.38 to 355.76) | 2434.94 (1777.14 to 3234.64) | 231.62 (169.05 to 307.7) | -0.26 (-0.35 to -0.17) | <0.001 | 141.51 (89.53 to 207.46) | 52.62 (33.29 to 77.14) | 485.07 (305.33 to 705.66) | 46.14 (29.04 to 67.13) | -0.25 (-0.33 to -0.18) | <0.001 |
| Swiss Confederation | 6258.1 (4597.39 to 8260.36) | 300.88 (221.04 to 397.15) | 9550.55 (6942.93 to 12570.39) | 307.22 (223.34 to 404.37) | 0 (-0.06 to 0.07) | 0.913 | 1064.57 (663.31 to 1570.7) | 51.18 (31.89 to 75.52) | 1578.59 (973.51 to 2349.77) | 50.78 (31.32 to 75.59) | -0.09 (-0.14 to -0.03) | 0.002 |
| Syrian Arab Republic | 5977.37 (4491.26 to 7854.65) | 368.78 (277.1 to 484.61) | 15303.73 (11387.61 to 20109.89) | 358.67 (266.89 to 471.31) | -0.18 (-0.43 to 0.06) | 0.141 | 1138.97 (737.01 to 1623.51) | 70.27 (45.47 to 100.17) | 2884.43 (1861.37 to 4131.11) | 67.6 (43.62 to 96.82) | -0.21 (-0.42 to 0.01) | 0.056 |
| Taiwan (Province of China) | 11351.9 (8345.86 to 14836.85) | 259.17 (190.54 to 338.73) | 28934.27 (21454.85 to 37421.36) | 316.67 (234.81 to 409.56) | 1.25 (0.86 to 1.65) | <0.001 | 1966.94 (1241.11 to 2821.75) | 44.91 (28.33 to 64.42) | 5217.6 (3329.72 to 7390.71) | 57.1 (36.44 to 80.89) | 1.36 (1 to 1.72) | <0.001 |
| Togolese Republic | 906.71 (654.42 to 1214.14) | 212.47 (153.35 to 284.51) | 2968.27 (2154.01 to 3959.82) | 205.58 (149.18 to 274.25) | -0.17 (-0.21 to -0.13) | <0.001 | 166.27 (102.65 to 243.08) | 38.96 (24.05 to 56.96) | 539.95 (330.54 to 792.74) | 37.4 (22.89 to 54.9) | -0.19 (-0.22 to -0.16) | <0.001 |
| Tokelau | 1.28 (0.96 to 1.65) | 428.55 (322.52 to 552.44) | 1.2 (0.9 to 1.58) | 344.13 (257.79 to 451.61) | -0.19 (-0.4 to 0.02) | 0.068 | 0.22 (0.14 to 0.32) | 74.28 (48.29 to 106.17) | 0.21 (0.14 to 0.31) | 61.52 (39.08 to 88.56) | -0.18 (-0.35 to -0.01) | 0.043 |
| Turkmenistan | 1828.25 (1362.92 to 2372.37) | 347.39 (258.98 to 450.78) | 3965.39 (2938.01 to 5199.99) | 312.12 (231.25 to 409.29) | -0.28 (-0.73 to 0.16) | 0.207 | 316.63 (201.85 to 456.21) | 60.17 (38.35 to 86.69) | 692.13 (439.24 to 998.36) | 54.48 (34.57 to 78.58) | -0.25 (-0.6 to 0.09) | 0.144 |
| Tuvalu | 6.57 (4.89 to 8.65) | 347.58 (258.37 to 457.18) | 9.87 (7.36 to 12.93) | 352.18 (262.53 to 461.38) | 0.33 (0.17 to 0.48) | <0.001 | 1.19 (0.76 to 1.71) | 62.72 (40.11 to 90.2) | 1.77 (1.14 to 2.56) | 63.29 (40.83 to 91.49) | 0.27 (0.14 to 0.39) | <0.001 |
| Ukraine | 66355.79 (48900.6 to 87595.02) | 411.98 (303.61 to 543.85) | 61484.02 (45673.6 to 80874.03) | 396.59 (294.61 to 521.67) | 0 (-0.21 to 0.21) | 0.996 | 11074.63 (7074.37 to 15949.71) | 68.76 (43.92 to 99.03) | 10224.97 (6577.66 to 14563.71) | 65.95 (42.43 to 93.94) | -0.03 (-0.19 to 0.13) | 0.702 |
| Union of the Comoros | 187.41 (139.55 to 245.88) | 298.58 (222.32 to 391.72) | 450.41 (333.07 to 591.23) | 286.07 (211.54 to 375.51) | -0.29 (-0.35 to -0.23) | <0.001 | 34.13 (21.5 to 49.24) | 54.37 (34.25 to 78.45) | 81.93 (51.83 to 117.52) | 52.04 (32.92 to 74.64) | -0.25 (-0.3 to -0.21) | <0.001 |
| United Arab Emirates | 511.17 (359.07 to 690.65) | 167.89 (117.93 to 226.83) | 7266.01 (5153.3 to 9803.61) | 176.7 (125.32 to 238.41) | 0.11 (-0.06 to 0.27) | 0.185 | 108.93 (66.28 to 161.05) | 35.78 (21.77 to 52.9) | 1507.17 (921.02 to 2225.84) | 36.65 (22.4 to 54.13) | 0.04 (-0.09 to 0.16) | 0.568 |
| United Kingdom of Great Britain and Northern Ireland | 46449.9 (33716.64 to 61263.25) | 281.48 (204.32 to 371.25) | 60250.43 (41872.65 to 80598.77) | 276.29 (192.01 to 369.6) | -0.09 (-0.14 to -0.03) | 0.003 | 7576.24 (4658.43 to 11267.56) | 45.91 (28.23 to 68.28) | 9547.47 (5726.27 to 14346.55) | 43.78 (26.26 to 65.79) | -0.18 (-0.23 to -0.13) | <0.001 |
| United Mexican States | 36375.36 (26619.91 to 48224.39) | 279.54 (204.57 to 370.6) | 101936.11 (74600.94 to 135374.43) | 290.13 (212.33 to 385.3) | 0.13 (0.03 to 0.22) | 0.01 | 6422.93 (4062.17 to 9291.09) | 49.36 (31.22 to 71.4) | 17313 (10958.01 to 25237.23) | 49.28 (31.19 to 71.83) | 0.04 (-0.04 to 0.12) | 0.335 |
| United Republic of Tanzania | 10288.94 (7634.1 to 13470.38) | 317.13 (235.3 to 415.2) | 23826.9 (17479.96 to 31262.37) | 268.55 (197.02 to 352.36) | -0.65 (-0.71 to -0.6) | <0.001 | 1874.15 (1186.8 to 2701.48) | 57.77 (36.58 to 83.27) | 4401 (2760.47 to 6293.3) | 49.6 (31.11 to 70.93) | -0.6 (-0.64 to -0.55) | <0.001 |
| United States Virgin Islands | 69.63 (50.81 to 92.74) | 248.87 (181.6 to 331.45) | 109.95 (81.65 to 144.08) | 357.69 (265.63 to 468.73) | 1.19 (1.09 to 1.28) | <0.001 | 12.5 (7.82 to 18.53) | 44.69 (27.96 to 66.23) | 18.43 (11.61 to 26.75) | 59.97 (37.75 to 87.02) | 0.96 (0.88 to 1.03) | <0.001 |
| United States of America | 289252.66 (218178.9 to 376018.33) | 446.89 (337.08 to 580.94) | 570598.65 (431638.23 to 737065.55) | 544.48 (411.88 to 703.32) | 0.98 (0.82 to 1.14) | <0.001 | 47471.75 (30592.95 to 68208.28) | 73.34 (47.27 to 105.38) | 92300.76 (59852.25 to 131482.3) | 88.08 (57.11 to 125.46) | 0.86 (0.74 to 0.98) | <0.001 |

Note: CI, confidence interval; AAPC, average annual percentage change; EOAD, early-onset Alzheimer’s disease and other dementias.

**Supplementary Table S10. Age-standardized Mortality and DALYs of EOAD in people aged 40-64 years and their AAPCs from 1990 to 2021 at the country levels.**

|  | Mortality | | | | | | DALYs | | | | | |
| --- | --- | --- | --- | --- | --- | --- | --- | --- | --- | --- | --- | --- |
| Country | No of people with EOAD in 1990 (000s) | Age standardised rate in 1990 (per 100 000) | No of people with EOAD in 2021 (000s) | Age standardised rate in 2021 (per 100 000) | AAPC (95% CI) | *p* value | No of people with EOAD in 1990 (000s) | Age standardised rate in 1990 (per 100 000) | No of people with EOAD in 2021 (000s) | Age standardised rate in 2021 (per 100 000) | AAPC (95% CI) | *p* value |
| American Samoa | 0.25 (0.05 to 0.77) | 3.2 (0.61 to 9.96) | 0.48 (0.09 to 1.51) | 3.35 (0.62 to 10.48) | 0.21 (-0.06 to 0.49) | 0.129 | 12.11 (5.17 to 28.87) | 157.56 (67.32 to 375.66) | 24.4 (10.59 to 58.15) | 169.03 (73.34 to 402.87) | 0.29 (0.06 to 0.52) | 0.016 |
| Antigua and Barbuda | 0.29 (0.06 to 0.9) | 2.64 (0.52 to 8.18) | 0.79 (0.15 to 2.48) | 2.72 (0.51 to 8.49) | 0.26 (-0.08 to 0.6) | 0.126 | 14.92 (6.5 to 35.01) | 134.88 (58.73 to 316.47) | 41.15 (18.35 to 96.43) | 141.02 (62.88 to 330.46) | 0.29 (-0.01 to 0.58) | 0.055 |
| Arab Republic of Egypt | 293.42 (56.08 to 920.34) | 3.12 (0.6 to 9.79) | 657.89 (126.81 to 2063.52) | 2.97 (0.57 to 9.3) | -0.11 (-0.25 to 0.03) | 0.13 | 14864.44 (6492.71 to 35157.47) | 158.04 (69.03 to 373.79) | 33733.01 (14895 to 78519.95) | 152.05 (67.14 to 353.93) | -0.03 (-0.14 to 0.08) | 0.555 |
| Argentine Republic | 205.03 (39.48 to 639.65) | 2.61 (0.5 to 8.13) | 323.8 (61.65 to 1011.01) | 2.58 (0.49 to 8.07) | 0.17 (0.1 to 0.25) | <0.001 | 11261.62 (5198.83 to 25487.28) | 143.1 (66.06 to 323.87) | 17308.8 (7875.69 to 39220.62) | 138.14 (62.86 to 313.02) | 0.04 (-0.02 to 0.11) | 0.187 |
| Australia | 130.97 (25.52 to 408.28) | 2.95 (0.57 to 9.19) | 268.01 (52.7 to 834.21) | 3.36 (0.66 to 10.45) | 0.78 (0.62 to 0.94) | <0.001 | 6837.48 (3077.86 to 15570.25) | 153.86 (69.26 to 350.36) | 13556.38 (6043.1 to 31251.74) | 169.74 (75.67 to 391.31) | 0.64 (0.51 to 0.77) | <0.001 |
| Barbados | 1.44 (0.28 to 4.44) | 2.75 (0.53 to 8.47) | 3.37 (0.65 to 10.57) | 3.24 (0.63 to 10.17) | 0.9 (0.59 to 1.21) | <0.001 | 74.35 (32.93 to 170.44) | 141.84 (62.81 to 325.15) | 170.4 (76.08 to 393.37) | 163.84 (73.15 to 378.22) | 0.79 (0.53 to 1.06) | <0.001 |
| Belize | 0.64 (0.12 to 2.01) | 2.68 (0.51 to 8.39) | 2.35 (0.45 to 7.23) | 2.48 (0.47 to 7.64) | -0.14 (-0.4 to 0.13) | 0.299 | 32.99 (14.33 to 77.26) | 137.9 (59.91 to 322.91) | 121.62 (53.98 to 282.1) | 128.45 (57.01 to 297.93) | -0.12 (-0.37 to 0.13) | 0.323 |
| Bermuda | 0.41 (0.08 to 1.3) | 2.52 (0.49 to 7.91) | 0.77 (0.15 to 2.33) | 3.14 (0.61 to 9.51) | 0.81 (0.59 to 1.03) | <0.001 | 21.95 (10.02 to 50.11) | 134.07 (61.21 to 306.09) | 40.51 (18.23 to 89.09) | 165.61 (74.53 to 364.2) | 0.8 (0.61 to 0.99) | <0.001 |
| Bolivarian Republic of Venezuela | 85.44 (16.33 to 270.12) | 2.88 (0.55 to 9.11) | 276.81 (53.62 to 891.77) | 3.43 (0.66 to 11.06) | 0.83 (0.6 to 1.05) | <0.001 | 4550.33 (2086.49 to 10372.7) | 153.5 (70.39 to 349.92) | 14212.43 (6091.73 to 33395.64) | 176.25 (75.54 to 414.14) | 0.71 (0.52 to 0.9) | <0.001 |
| Bosnia and Herzegovina | 39.31 (7.52 to 121.2) | 3.24 (0.62 to 10) | 42.11 (8.27 to 127.03) | 3.51 (0.69 to 10.58) | 0.15 (-0.16 to 0.46) | 0.333 | 2057.65 (916.99 to 4641.18) | 169.72 (75.64 to 382.82) | 2188.95 (997.49 to 4841.9) | 182.32 (83.08 to 403.29) | 0.23 (-0.03 to 0.49) | 0.086 |
| Brunei Darussalam | 0.75 (0.14 to 2.41) | 1.97 (0.38 to 6.31) | 3.15 (0.58 to 9.76) | 2.46 (0.45 to 7.62) | 1.07 (0.71 to 1.42) | <0.001 | 39.92 (17.3 to 96.52) | 104.54 (45.32 to 252.76) | 164.16 (73.54 to 376.2) | 128.27 (57.46 to 293.95) | 0.99 (0.7 to 1.29) | <0.001 |
| Burkina Faso | 38.16 (7.41 to 115.8) | 2.97 (0.58 to 9.01) | 71.57 (13.29 to 221.35) | 2.35 (0.44 to 7.27) | -0.78 (-0.98 to -0.57) | <0.001 | 1829.79 (746.64 to 4372.59) | 142.34 (58.08 to 340.14) | 3555.13 (1487.77 to 8263.24) | 116.78 (48.87 to 271.45) | -0.65 (-0.79 to -0.51) | <0.001 |
| Canada | 213.66 (41.55 to 666.96) | 2.92 (0.57 to 9.12) | 442.33 (86.64 to 1368.99) | 3.57 (0.7 to 11.04) | 0.95 (0.7 to 1.21) | <0.001 | 13471.31 (6873.16 to 28067.43) | 184.15 (93.95 to 383.67) | 28085.22 (15090.39 to 57724.27) | 226.48 (121.69 to 465.49) | 0.82 (0.7 to 0.94) | <0.001 |
| Central African Republic | 14.88 (2.87 to 45.91) | 3.78 (0.73 to 11.65) | 28.95 (5.06 to 89.85) | 3.25 (0.57 to 10.07) | -0.69 (-0.92 to -0.46) | <0.001 | 725.64 (306.04 to 1738.16) | 184.15 (77.67 to 441.1) | 1435.79 (602.91 to 3442.42) | 160.98 (67.6 to 385.97) | -0.59 (-0.79 to -0.4) | <0.001 |
| Commonwealth of Dominica | 0.36 (0.07 to 1.1) | 2.82 (0.55 to 8.66) | 0.62 (0.12 to 1.95) | 3.07 (0.6 to 9.62) | 0.23 (-0.07 to 0.53) | 0.124 | 18.28 (7.95 to 42.06) | 143.8 (62.54 to 330.93) | 31.68 (13.97 to 74.63) | 156.56 (69.03 to 368.85) | 0.22 (-0.06 to 0.5) | 0.117 |
| Commonwealth of the Bahamas | 1.03 (0.19 to 3.26) | 2.26 (0.42 to 7.11) | 3.05 (0.57 to 9.5) | 2.54 (0.47 to 7.9) | 0.19 (-0.05 to 0.42) | 0.119 | 54.07 (24.04 to 124.67) | 118.1 (52.5 to 272.29) | 160.53 (70.99 to 369.58) | 133.47 (59.03 to 307.28) | 0.24 (0.03 to 0.45) | 0.025 |
| Cook Islands | 0.13 (0.03 to 0.42) | 3.66 (0.7 to 11.54) | 0.22 (0.04 to 0.7) | 3.85 (0.75 to 12.13) | -0.14 (-0.4 to 0.12) | 0.294 | 6.52 (2.76 to 15.67) | 178.93 (75.75 to 429.99) | 11.25 (4.92 to 26.6) | 195.99 (85.78 to 463.33) | 0.02 (-0.2 to 0.24) | 0.864 |
| Czech Republic | 96.19 (18.98 to 301.51) | 3.12 (0.62 to 9.78) | 114.05 (21.63 to 343.23) | 3.04 (0.58 to 9.14) | 0.62 (0.28 to 0.95) | 0.001 | 4969.78 (2219.33 to 11232.26) | 161.24 (72 to 364.42) | 5988.55 (2735.1 to 13308.3) | 159.48 (72.84 to 354.41) | 0.52 (0.23 to 0.81) | 0.001 |
| Democratic People's Republic of Korea | 159.1 (28.22 to 497.94) | 3.08 (0.55 to 9.63) | 324.13 (59.9 to 1008.78) | 3.65 (0.67 to 11.35) | -0.21 (-0.52 to 0.1) | 0.183 | 7964.3 (3382.64 to 18990.03) | 154.06 (65.43 to 367.34) | 15528.76 (6313.56 to 37701.46) | 174.71 (71.03 to 424.17) | -0.3 (-0.59 to -0.01) | 0.042 |
| Democratic Republic of Sao Tome and Principe | 0.47 (0.09 to 1.42) | 2.88 (0.55 to 8.73) | 0.92 (0.18 to 2.94) | 2.3 (0.44 to 7.33) | -1.03 (-1.29 to -0.77) | <0.001 | 22.87 (9.72 to 53.55) | 140.12 (59.52 to 328.06) | 44.48 (18.5 to 109.37) | 110.83 (46.09 to 272.5) | -0.98 (-1.21 to -0.76) | <0.001 |
| Democratic Republic of Timor-Leste | 2.79 (0.51 to 8.83) | 2.44 (0.44 to 7.72) | 7.72 (1.45 to 24.11) | 3.34 (0.63 to 10.42) | 1.48 (1.19 to 1.78) | <0.001 | 159.22 (73.9 to 365.67) | 139.29 (64.65 to 319.89) | 399.95 (175.17 to 919.26) | 172.84 (75.7 to 397.26) | 1.09 (0.84 to 1.35) | <0.001 |
| Democratic Republic of the Congo | 201.24 (38.27 to 622.56) | 3.98 (0.76 to 12.33) | 540.92 (97.07 to 1610.83) | 3.98 (0.71 to 11.85) | 0.06 (-0.29 to 0.41) | 0.746 | 9794.35 (4127.24 to 23414.56) | 193.93 (81.72 to 463.62) | 25353.96 (9805.9 to 60565.71) | 186.58 (72.16 to 445.69) | -0.06 (-0.35 to 0.23) | 0.66 |
| Democratic Socialist Republic of Sri Lanka | 99.18 (18.69 to 302.64) | 2.93 (0.55 to 8.93) | 238.06 (42.04 to 721.54) | 3.58 (0.63 to 10.85) | 0.98 (0.85 to 1.1) | <0.001 | 5469.93 (2567.21 to 12067.4) | 161.46 (75.78 to 356.2) | 12678.75 (5588.29 to 28150.45) | 190.66 (84.03 to 423.31) | 0.83 (0.72 to 0.95) | <0.001 |
| Dominican Republic | 27.02 (5.11 to 84.42) | 2.47 (0.47 to 7.72) | 68.12 (12.39 to 210.91) | 2.54 (0.46 to 7.86) | 0.12 (0.01 to 0.23) | 0.037 | 1410.36 (619.24 to 3211.81) | 128.96 (56.62 to 293.67) | 3494.29 (1537.52 to 8137.51) | 130.19 (57.28 to 303.18) | 0.06 (-0.04 to 0.15) | 0.229 |
| Eastern Republic of Uruguay | 23.11 (4.34 to 71.85) | 2.83 (0.53 to 8.81) | 29.1 (5.62 to 91.22) | 2.86 (0.55 to 8.96) | 0.1 (-0.03 to 0.23) | 0.126 | 1263.9 (585.76 to 2851.19) | 154.9 (71.79 to 349.42) | 1541.24 (701.87 to 3460.11) | 151.32 (68.91 to 339.72) | -0.02 (-0.13 to 0.09) | 0.698 |
| Federal Democratic Republic of Ethiopia | 224.68 (43.44 to 720.92) | 3.43 (0.66 to 11) | 468.24 (86.76 to 1429.72) | 3.12 (0.58 to 9.53) | -0.18 (-0.25 to -0.12) | <0.001 | 11154.13 (4871.98 to 26953.66) | 170.22 (74.35 to 411.34) | 22989.42 (9739.09 to 54187.03) | 153.17 (64.89 to 361.03) | -0.21 (-0.27 to -0.15) | <0.001 |
| Federal Democratic Republic of Nepal | 62.05 (11.53 to 196.92) | 2.01 (0.37 to 6.39) | 171.31 (30.91 to 523.38) | 2.6 (0.47 to 7.95) | 1 (0.9 to 1.1) | <0.001 | 3352.29 (1519.55 to 7719.03) | 108.82 (49.33 to 250.57) | 8310.07 (3391.56 to 19396.23) | 126.29 (51.54 to 294.76) | 0.58 (0.52 to 0.65) | <0.001 |
| Federal Republic of Germany | 776.55 (148.21 to 2391.4) | 3.06 (0.58 to 9.43) | 1027.37 (203.26 to 3192.13) | 3.48 (0.69 to 10.8) | 0.04 (-0.25 to 0.34) | 0.764 | 42181.87 (19591.36 to 93118.58) | 166.35 (77.26 to 367.23) | 54933.92 (25671.3 to 124383.52) | 185.88 (86.86 to 420.88) | 0.07 (-0.19 to 0.33) | 0.6 |
| Federal Republic of Nigeria | 287.76 (53.66 to 926.28) | 2.16 (0.4 to 6.95) | 751.06 (138.26 to 2439) | 2.25 (0.41 to 7.29) | 0.11 (-0.13 to 0.34) | 0.362 | 14895.28 (6640.16 to 35289.93) | 111.73 (49.81 to 264.71) | 37515.17 (16101.08 to 91356.01) | 112.19 (48.15 to 273.21) | -0.02 (-0.21 to 0.16) | 0.809 |
| Federal Republic of Somalia | 23.4 (4.45 to 75.91) | 2.31 (0.44 to 7.48) | 64.69 (12.15 to 197.29) | 2.49 (0.47 to 7.59) | 1.06 (0.69 to 1.43) | <0.001 | 1231.83 (530.53 to 2966.81) | 121.42 (52.29 to 292.43) | 3194.07 (1339.08 to 7481.93) | 122.85 (51.5 to 287.77) | 0.75 (0.4 to 1.1) | <0.001 |
| Federated States of Micronesia | 0.52 (0.1 to 1.69) | 3.84 (0.74 to 12.51) | 1 (0.19 to 3.16) | 4.05 (0.76 to 12.79) | 0.61 (0.18 to 1.05) | 0.007 | 24.37 (10.01 to 61.34) | 180.01 (73.93 to 453.14) | 48.19 (19.85 to 117.98) | 195.06 (80.36 to 477.51) | 0.68 (0.33 to 1.04) | <0.001 |
| Federative Republic of Brazil | 1109.26 (215.59 to 3464.45) | 4.13 (0.8 to 12.88) | 3065.29 (600.08 to 9436.95) | 4.7 (0.92 to 14.48) | 0.5 (0.35 to 0.65) | <0.001 | 55133.41 (24711.64 to 129636.07) | 205.04 (91.9 to 482.12) | 149561.87 (66169.6 to 351245.35) | 229.55 (101.56 to 539.09) | 0.47 (0.36 to 0.59) | <0.001 |
| French Republic | 454.15 (87.4 to 1418.92) | 2.82 (0.54 to 8.8) | 585.97 (112.19 to 1762.71) | 2.77 (0.53 to 8.33) | 0.28 (0.05 to 0.51) | 0.02 | 22162.51 (9588.97 to 52332.53) | 137.44 (59.46 to 324.53) | 29139.75 (12727.63 to 67145.33) | 137.74 (60.16 to 317.39) | 0.31 (0.12 to 0.5) | 0.002 |
| Gabonese Republic | 7.05 (1.32 to 21.65) | 4.86 (0.91 to 14.93) | 14.25 (2.5 to 44.67) | 4.04 (0.71 to 12.67) | -0.64 (-0.96 to -0.32) | <0.001 | 329.75 (135.22 to 792.38) | 227.38 (93.24 to 546.37) | 674.84 (272.11 to 1667.4) | 191.36 (77.16 to 472.8) | -0.55 (-0.84 to -0.26) | <0.001 |
| Georgia | 52.97 (10.31 to 161.11) | 3.49 (0.68 to 10.6) | 44.21 (8.57 to 134.11) | 3.8 (0.74 to 11.52) | 0.15 (-0.29 to 0.6) | 0.489 | 2774.55 (1266.75 to 6219.91) | 182.61 (83.37 to 409.37) | 2237.21 (1000.33 to 5133.71) | 192.1 (85.89 to 440.8) | 0.1 (-0.28 to 0.48) | 0.583 |
| Grand Duchy of Luxembourg | 2.74 (0.53 to 8.48) | 2.34 (0.45 to 7.23) | 4.68 (0.91 to 14.45) | 2.07 (0.4 to 6.38) | -0.46 (-0.65 to -0.27) | <0.001 | 141.14 (62.26 to 319.6) | 120.4 (53.11 to 272.63) | 246.08 (108.34 to 560.15) | 108.68 (47.85 to 247.38) | -0.37 (-0.54 to -0.19) | <0.001 |
| Greenland | 0.34 (0.06 to 1.07) | 2.69 (0.51 to 8.36) | 0.76 (0.15 to 2.31) | 4.07 (0.78 to 12.37) | 1.02 (0.62 to 1.43) | <0.001 | 19.61 (9.09 to 43.41) | 153.33 (71.05 to 339.41) | 42.09 (19.61 to 92.2) | 225.48 (105.08 to 493.95) | 1 (0.64 to 1.35) | <0.001 |
| Grenada | 0.42 (0.08 to 1.28) | 3.15 (0.6 to 9.6) | 0.85 (0.16 to 2.61) | 2.75 (0.52 to 8.41) | -0.18 (-0.39 to 0.02) | 0.081 | 21.3 (9.33 to 48.98) | 159.49 (69.83 to 366.7) | 44.26 (18.82 to 100.07) | 142.56 (60.62 to 322.32) | -0.15 (-0.33 to 0.02) | 0.08 |
| Guam | 0.86 (0.16 to 2.7) | 3.25 (0.62 to 10.22) | 1.86 (0.36 to 5.93) | 3.88 (0.74 to 12.34) | 0.69 (0.39 to 0.99) | <0.001 | 42.69 (18.42 to 101.71) | 161.49 (69.68 to 384.75) | 93.47 (40.92 to 223.62) | 194.56 (85.19 to 465.47) | 0.7 (0.45 to 0.94) | <0.001 |
| Hashemite Kingdom of Jordan | 13.7 (2.71 to 42.21) | 2.87 (0.57 to 8.83) | 70.07 (13.02 to 213.62) | 2.49 (0.46 to 7.6) | -1.1 (-1.37 to -0.84) | <0.001 | 726.55 (325.57 to 1684.58) | 152.05 (68.13 to 352.54) | 3929.45 (1812.18 to 8597.87) | 139.75 (64.45 to 305.78) | -0.8 (-1.01 to -0.59) | <0.001 |
| Hellenic Republic | 99.11 (19.08 to 307.71) | 3.1 (0.6 to 9.62) | 106.04 (20.61 to 319.46) | 2.88 (0.56 to 8.67) | -0.38 (-0.52 to -0.23) | <0.001 | 5151.06 (2348.61 to 11751.32) | 161.03 (73.42 to 367.36) | 5504 (2531.42 to 12391.55) | 149.34 (68.69 to 336.22) | -0.35 (-0.47 to -0.24) | <0.001 |
| Hungary | 104.67 (20.09 to 330.32) | 3.28 (0.63 to 10.36) | 106.36 (20.33 to 324.13) | 3.09 (0.59 to 9.4) | 0.46 (0.23 to 0.68) | <0.001 | 5403.37 (2393.23 to 12442.83) | 169.44 (75.05 to 390.18) | 5561.32 (2506.79 to 12570.23) | 161.31 (72.71 to 364.6) | 0.41 (0.21 to 0.61) | <0.001 |
| Independent State of Papua New Guinea | 20.67 (3.83 to 66.39) | 3.28 (0.61 to 10.54) | 55.51 (10.03 to 173.48) | 2.85 (0.51 to 8.89) | -0.48 (-0.67 to -0.29) | <0.001 | 1038.65 (448.17 to 2525.47) | 164.88 (71.15 to 400.91) | 2820.76 (1184.66 to 6608.59) | 144.6 (60.73 to 338.77) | -0.43 (-0.59 to -0.28) | <0.001 |
| Independent State of Samoa | 1.02 (0.19 to 3.14) | 4.23 (0.79 to 13.02) | 1.66 (0.31 to 5.25) | 3.93 (0.74 to 12.41) | -0.31 (-0.62 to -0.01) | 0.042 | 47.43 (19.31 to 114.93) | 196.55 (80.03 to 476.27) | 78.35 (31.46 to 194.19) | 185.21 (74.37 to 459.03) | -0.25 (-0.5 to 0.01) | 0.059 |
| Ireland | 21.77 (4.21 to 68.96) | 2.57 (0.5 to 8.15) | 41.33 (7.96 to 127.52) | 2.54 (0.49 to 7.83) | 0.13 (0.05 to 0.2) | 0.001 | 1125.16 (503.98 to 2597.66) | 133.02 (59.58 to 307.11) | 2132.69 (963.12 to 4827.54) | 131 (59.16 to 296.52) | 0.09 (0.03 to 0.14) | 0.004 |
| Islamic Republic of Afghanistan | 108.74 (19.85 to 324.71) | 5.59 (1.02 to 16.69) | 101.45 (17.95 to 316.84) | 2.43 (0.43 to 7.58) | -3.52 (-4.29 to -2.75) | <0.001 | 4834.99 (1858.26 to 11824.92) | 248.48 (95.5 to 607.7) | 5108.99 (2014.29 to 12365.66) | 122.3 (48.22 to 296.01) | -3.22 (-3.92 to -2.52) | <0.001 |
| Islamic Republic of Iran | 306.3 (58.72 to 946.35) | 3.77 (0.72 to 11.66) | 756.04 (141.17 to 2318.51) | 3.09 (0.58 to 9.48) | -0.59 (-0.89 to -0.29) | <0.001 | 15803.11 (7121.03 to 36185.51) | 194.77 (87.76 to 445.97) | 40366.59 (18597.02 to 91406.29) | 165 (76.02 to 373.63) | -0.42 (-0.68 to -0.16) | 0.003 |
| Islamic Republic of Mauritania | 6.91 (1.31 to 21.78) | 2.48 (0.47 to 7.83) | 18.41 (3.24 to 57.47) | 2.76 (0.49 to 8.61) | 0.25 (0.06 to 0.44) | 0.011 | 340.11 (144.12 to 812.09) | 122.35 (51.85 to 292.14) | 869.95 (356.48 to 2122.38) | 130.33 (53.4 to 317.96) | 0.19 (0.03 to 0.35) | 0.025 |
| Islamic Republic of Pakistan | 383.72 (70.79 to 1195.28) | 2.32 (0.43 to 7.22) | 1006.11 (186.64 to 3168.3) | 2.36 (0.44 to 7.43) | -0.06 (-0.11 to -0.01) | 0.012 | 19452.02 (8318.63 to 45186.66) | 117.48 (50.24 to 272.9) | 48915.52 (20380.87 to 118089.27) | 114.67 (47.78 to 276.82) | -0.17 (-0.2 to -0.13) | <0.001 |
| Jamaica | 10.35 (1.95 to 32.43) | 2.73 (0.52 to 8.57) | 20.99 (3.96 to 64.89) | 2.79 (0.53 to 8.61) | -0.01 (-0.21 to 0.19) | 0.92 | 563.5 (255.57 to 1257.66) | 148.94 (67.55 to 332.42) | 1130.2 (520.9 to 2559.42) | 150.03 (69.15 to 339.76) | -0.02 (-0.21 to 0.17) | 0.811 |
| Japan | 1385.29 (264.36 to 4328.37) | 3.23 (0.62 to 10.09) | 1481.91 (291.04 to 4586.62) | 3.45 (0.68 to 10.67) | 0.34 (0.07 to 0.6) | 0.015 | 72204.4 (33243.48 to 164969.91) | 168.29 (77.48 to 384.51) | 77626.7 (35854.09 to 174768.96) | 180.55 (83.39 to 406.5) | 0.32 (0.08 to 0.56) | 0.011 |
| Kingdom of Bahrain | 1.89 (0.37 to 6) | 2.48 (0.48 to 7.85) | 9.89 (1.82 to 29.81) | 2.08 (0.38 to 6.28) | -0.56 (-1.04 to -0.08) | 0.024 | 100.78 (44.41 to 232.83) | 131.94 (58.14 to 304.85) | 564.76 (257.28 to 1235.94) | 118.94 (54.19 to 260.3) | -0.22 (-0.64 to 0.2) | 0.289 |
| Kingdom of Belgium | 95.45 (17.94 to 301.19) | 3.21 (0.6 to 10.13) | 123.34 (24.06 to 376.29) | 3.24 (0.63 to 9.87) | 0.08 (-0.15 to 0.3) | 0.491 | 4985.62 (2299.21 to 11563.14) | 167.6 (77.29 to 388.72) | 6317.03 (2814.08 to 14371.87) | 165.72 (73.83 to 377.04) | 0.03 (-0.16 to 0.23) | 0.717 |
| Kingdom of Bhutan | 1.9 (0.34 to 6.03) | 2.31 (0.42 to 7.33) | 4.69 (0.84 to 14.59) | 2.71 (0.49 to 8.43) | 0.58 (0.51 to 0.65) | <0.001 | 95.59 (39.93 to 229.4) | 116.15 (48.52 to 278.74) | 217.4 (85.12 to 532) | 125.63 (49.19 to 307.43) | 0.33 (0.27 to 0.39) | <0.001 |
| Kingdom of Cambodia | 49.92 (9.59 to 158.75) | 3.47 (0.67 to 11.02) | 167.21 (30.17 to 528.65) | 4.45 (0.8 to 14.08) | 0.95 (0.71 to 1.2) | <0.001 | 2550.45 (1111.38 to 6087.04) | 177.1 (77.17 to 422.67) | 7964.61 (3277.94 to 19543.36) | 212.17 (87.32 to 520.63) | 0.7 (0.49 to 0.92) | <0.001 |
| Kingdom of Denmark | 40.84 (7.86 to 129.16) | 2.64 (0.51 to 8.34) | 55.67 (10.97 to 167.04) | 2.94 (0.58 to 8.81) | 0.54 (0.38 to 0.7) | <0.001 | 2031.51 (900.23 to 4781.02) | 131.17 (58.13 to 308.7) | 2710.08 (1132.92 to 6334.67) | 142.99 (59.77 to 334.22) | 0.37 (0.24 to 0.5) | <0.001 |
| Kingdom of Eswatini | 2.64 (0.49 to 8.04) | 2.66 (0.49 to 8.11) | 5.74 (0.99 to 17.34) | 2.96 (0.51 to 8.96) | 0.57 (0.43 to 0.72) | <0.001 | 133.34 (56.03 to 310.39) | 134.58 (56.56 to 313.3) | 275.88 (113.5 to 658) | 142.62 (58.67 to 340.16) | 0.36 (0.25 to 0.47) | <0.001 |
| Kingdom of Lesotho | 6.89 (1.31 to 20.87) | 2.86 (0.54 to 8.65) | 10.46 (1.88 to 31.6) | 3.19 (0.57 to 9.63) | 0.88 (0.67 to 1.09) | <0.001 | 358.45 (158.41 to 813.86) | 148.49 (65.62 to 337.15) | 508.92 (207.28 to 1198.73) | 155.08 (63.16 to 365.28) | 0.59 (0.41 to 0.77) | <0.001 |
| Kingdom of Morocco | 140.74 (26.96 to 419.09) | 3.49 (0.67 to 10.38) | 373.51 (66.35 to 1185.27) | 3.75 (0.67 to 11.89) | 0.34 (0.07 to 0.62) | 0.016 | 7140.91 (3121.83 to 16158.38) | 176.94 (77.36 to 400.38) | 18322.44 (7841.6 to 43810.02) | 183.85 (78.68 to 439.59) | 0.23 (-0.01 to 0.47) | 0.057 |
| Kingdom of Norway | 34.22 (6.54 to 105.91) | 2.98 (0.57 to 9.22) | 51.38 (10.13 to 156.46) | 2.94 (0.58 to 8.95) | 0.29 (0.13 to 0.46) | 0.001 | 1771.66 (810.17 to 4028.18) | 154.31 (70.57 to 350.85) | 2678.33 (1220.22 to 6007.3) | 153.25 (69.82 to 343.72) | 0.21 (0.09 to 0.33) | 0.001 |
| Kingdom of Saudi Arabia | 56.39 (10.26 to 175.97) | 2.49 (0.45 to 7.78) | 203.02 (38.06 to 638.25) | 1.92 (0.36 to 6.02) | -0.87 (-0.92 to -0.82) | <0.001 | 2900.81 (1225.68 to 6892.31) | 128.24 (54.19 to 304.7) | 10940.6 (4729.47 to 24933.1) | 103.26 (44.64 to 235.33) | -0.74 (-0.79 to -0.69) | <0.001 |
| Kingdom of Spain | 357.25 (68.57 to 1110.97) | 3.28 (0.63 to 10.21) | 498.22 (98.02 to 1499.25) | 2.85 (0.56 to 8.59) | -0.66 (-0.84 to -0.48) | <0.001 | 17794.55 (7886.06 to 41106.96) | 163.58 (72.5 to 377.89) | 25018.64 (10994.56 to 56961.95) | 143.31 (62.98 to 326.29) | -0.55 (-0.69 to -0.4) | <0.001 |
| Kingdom of Sweden | 65.01 (12.39 to 201.94) | 2.53 (0.48 to 7.85) | 85.42 (16.68 to 254.19) | 2.69 (0.53 to 8) | 0.36 (0.19 to 0.52) | <0.001 | 3539.7 (1635.85 to 7993.57) | 137.56 (63.57 to 310.65) | 4730.01 (2242.06 to 10151.24) | 148.95 (70.6 to 319.66) | 0.39 (0.25 to 0.53) | <0.001 |
| Kingdom of Thailand | 418.86 (81.19 to 1292.43) | 3.66 (0.71 to 11.28) | 1191.4 (229.13 to 3656.18) | 4.57 (0.88 to 14.02) | 0.51 (0.22 to 0.81) | 0.001 | 20977.78 (9181.65 to 49056.21) | 183.06 (80.12 to 428.09) | 57249.77 (24292.24 to 135603.66) | 219.56 (93.16 to 520.06) | 0.58 (0.34 to 0.82) | <0.001 |
| Kingdom of Tonga | 0.64 (0.12 to 1.97) | 4.01 (0.76 to 12.41) | 0.78 (0.15 to 2.45) | 3.58 (0.68 to 11.28) | -0.62 (-0.74 to -0.49) | <0.001 | 30.64 (13.09 to 73.47) | 193.42 (82.64 to 463.76) | 38.16 (15.92 to 91.2) | 175.86 (73.35 to 420.26) | -0.55 (-0.67 to -0.43) | <0.001 |
| Kingdom of the Netherlands | 114.89 (22.27 to 359.38) | 2.7 (0.52 to 8.45) | 187.53 (35.73 to 564.34) | 3.24 (0.62 to 9.75) | 0.69 (0.55 to 0.84) | <0.001 | 5893.81 (2627.42 to 13555.77) | 138.52 (61.75 to 318.6) | 9549.79 (4266.92 to 21552.89) | 164.95 (73.7 to 372.28) | 0.68 (0.55 to 0.81) | <0.001 |
| Kyrgyz Republic | 25.02 (4.74 to 79.05) | 3.32 (0.63 to 10.48) | 44.8 (8.49 to 142.78) | 2.93 (0.56 to 9.35) | -0.63 (-1.32 to 0.06) | 0.073 | 1304.89 (591.63 to 3025.75) | 173.03 (78.45 to 401.21) | 2373.12 (1083.35 to 5454.21) | 155.38 (70.93 to 357.11) | -0.42 (-1.03 to 0.19) | 0.166 |
| Lao People's Democratic Republic | 19.4 (3.59 to 62.23) | 3.1 (0.57 to 9.94) | 49.37 (9.1 to 151.28) | 3.19 (0.59 to 9.78) | 0.05 (-0.07 to 0.17) | 0.419 | 1033.84 (455.83 to 2457.8) | 165.19 (72.83 to 392.71) | 2577.78 (1135.42 to 5945.93) | 166.72 (73.44 to 384.56) | 0 (-0.13 to 0.12) | 0.987 |
| Lebanese Republic | 20.46 (3.96 to 64.45) | 3.26 (0.63 to 10.28) | 39.14 (7.64 to 121.3) | 2.8 (0.55 to 8.69) | -0.51 (-0.67 to -0.36) | <0.001 | 1120.08 (517.54 to 2516.14) | 178.59 (82.52 to 401.18) | 2216.6 (1041.07 to 4935.04) | 158.78 (74.58 to 353.51) | -0.42 (-0.52 to -0.31) | <0.001 |
| Malaysia | 106.15 (20.53 to 328.65) | 3.54 (0.69 to 10.97) | 325.75 (63.35 to 1002.54) | 4.1 (0.8 to 12.6) | 0.76 (0.55 to 0.98) | <0.001 | 5388.31 (2382.37 to 12615.37) | 179.81 (79.5 to 420.99) | 16055.5 (7021.44 to 37784.19) | 201.85 (88.27 to 475.02) | 0.69 (0.51 to 0.88) | <0.001 |
| Mongolia | 7.58 (1.37 to 23.67) | 2.62 (0.47 to 8.17) | 20.33 (3.83 to 64.04) | 2.41 (0.45 to 7.6) | -0.91 (-1.4 to -0.42) | 0.001 | 402.37 (177.98 to 920.67) | 138.88 (61.43 to 317.76) | 1126.22 (515.34 to 2535.72) | 133.59 (61.13 to 300.79) | -0.58 (-1.01 to -0.15) | 0.01 |
| Montenegro | 5.35 (1.01 to 16.03) | 3.27 (0.62 to 9.79) | 6.93 (1.39 to 21.95) | 3.34 (0.67 to 10.59) | 0.1 (-0.16 to 0.36) | 0.43 | 280.34 (126.42 to 627.18) | 171.26 (77.23 to 383.15) | 362.75 (164.27 to 825.18) | 175.09 (79.29 to 398.28) | 0.16 (-0.05 to 0.37) | 0.138 |
| New Zealand | 27.87 (5.36 to 85.89) | 3.24 (0.62 to 9.97) | 56.67 (11.16 to 172.66) | 3.59 (0.71 to 10.95) | 0.57 (0.42 to 0.72) | <0.001 | 1497.09 (687.38 to 3359.27) | 173.85 (79.82 to 390.1) | 3019.68 (1404.68 to 6730.14) | 191.53 (89.09 to 426.87) | 0.49 (0.37 to 0.61) | <0.001 |
| North Macedonia | 16 (3.05 to 49.55) | 3.05 (0.58 to 9.46) | 24.55 (4.62 to 75.1) | 3.16 (0.6 to 9.67) | 0.16 (0.01 to 0.31) | 0.037 | 837.62 (371.59 to 1896.66) | 159.86 (70.92 to 361.98) | 1293.99 (584.91 to 2899.32) | 166.66 (75.33 to 373.42) | 0.22 (0.08 to 0.36) | 0.003 |
| Northern Mariana Islands | 0.17 (0.03 to 0.55) | 1.96 (0.36 to 6.35) | 0.61 (0.12 to 1.91) | 3.54 (0.68 to 11.13) | 2.27 (1.66 to 2.89) | <0.001 | 9.06 (3.94 to 21.43) | 104.34 (45.41 to 246.8) | 30.92 (13.4 to 73.36) | 180.25 (78.1 to 427.62) | 2.11 (1.61 to 2.62) | <0.001 |
| Palestine | 8.22 (1.52 to 25.53) | 3.39 (0.63 to 10.54) | 23.76 (4.59 to 75.23) | 2.61 (0.51 to 8.28) | -1.16 (-1.35 to -0.97) | <0.001 | 430.16 (192.01 to 983.52) | 177.61 (79.28 to 406.07) | 1304.23 (590.87 to 2974.05) | 143.47 (65 to 327.15) | -0.93 (-1.12 to -0.75) | <0.001 |
| People's Democratic Republic of Algeria | 124.38 (24.06 to 382.6) | 3.6 (0.7 to 11.07) | 359.94 (69.52 to 1130.1) | 3.26 (0.63 to 10.25) | -0.31 (-0.53 to -0.09) | 0.008 | 6360.31 (2790.24 to 14563.07) | 184.05 (80.74 to 421.42) | 18425.65 (8021.69 to 43133.01) | 167.09 (72.74 to 391.14) | -0.28 (-0.48 to -0.08) | 0.007 |
| People's Republic of Bangladesh | 348.45 (65.67 to 1065.56) | 2.4 (0.45 to 7.35) | 1082.6 (202.39 to 3329.13) | 2.81 (0.53 to 8.66) | 0.55 (0.42 to 0.68) | <0.001 | 16947.03 (7012.79 to 40303.38) | 116.84 (48.35 to 277.86) | 50560.13 (19981.28 to 123218.74) | 131.47 (51.96 to 320.39) | 0.44 (0.31 to 0.56) | <0.001 |
| People's Republic of China | 9979.34 (1908.41 to 29882.84) | 4.07 (0.78 to 12.19) | 20967.32 (4026.78 to 66791.06) | 4.15 (0.8 to 13.21) | 0.19 (-0.02 to 0.4) | 0.074 | 493008.23 (216637.44 to 1141993.11) | 201.12 (88.38 to 465.87) | 1123825.79 (507111.29 to 2613285.53) | 222.25 (100.29 to 516.8) | 0.38 (0.19 to 0.58) | <0.001 |
| Plurinational State of Bolivia | 24.24 (4.47 to 76.82) | 2.46 (0.45 to 7.81) | 69.15 (13.01 to 215.35) | 2.61 (0.49 to 8.14) | 0.38 (0.31 to 0.45) | <0.001 | 1199.52 (509.64 to 2908.13) | 121.88 (51.78 to 295.48) | 3397.1 (1453.7 to 8026.81) | 128.35 (54.92 to 303.27) | 0.35 (0.28 to 0.42) | <0.001 |
| Portuguese Republic | 89.07 (17.22 to 275.38) | 3.07 (0.59 to 9.48) | 116.2 (22.98 to 350.03) | 3 (0.59 to 9.04) | -0.02 (-0.12 to 0.08) | 0.678 | 4528.48 (2000.56 to 10402.98) | 155.92 (68.88 to 358.18) | 5922.47 (2629.97 to 13501.47) | 152.88 (67.89 to 348.53) | -0.01 (-0.1 to 0.07) | 0.801 |
| Principality of Andorra | 0.39 (0.07 to 1.2) | 2.65 (0.51 to 8.18) | 0.92 (0.17 to 2.91) | 2.55 (0.47 to 8.03) | -0.15 (-0.34 to 0.04) | 0.128 | 20 (8.78 to 45.7) | 136.04 (59.74 to 310.9) | 48.57 (21.16 to 114.91) | 134.01 (58.38 to 317.02) | -0.07 (-0.23 to 0.1) | 0.415 |
| Principality of Monaco | 0.33 (0.06 to 1) | 3.08 (0.57 to 9.42) | 0.42 (0.08 to 1.25) | 2.99 (0.57 to 8.96) | -0.15 (-0.25 to -0.04) | 0.008 | 16.62 (7.49 to 38.32) | 157.25 (70.88 to 362.61) | 21.83 (9.67 to 47.99) | 157.1 (69.58 to 345.35) | -0.09 (-0.18 to 0) | 0.05 |
| Puerto Rico | 22.92 (4.39 to 71.43) | 2.65 (0.51 to 8.26) | 36.22 (6.91 to 112.78) | 3.32 (0.63 to 10.34) | 0.87 (0.8 to 0.94) | <0.001 | 1195.23 (526.41 to 2747.4) | 138.24 (60.89 to 317.77) | 1842.05 (806.29 to 4159.76) | 168.91 (73.93 to 381.43) | 0.75 (0.7 to 0.81) | <0.001 |
| Republic of Albania | 18.02 (3.35 to 55.13) | 2.96 (0.55 to 9.06) | 33.63 (6.43 to 106.73) | 3.91 (0.75 to 12.41) | 0.79 (0.54 to 1.03) | <0.001 | 937.93 (416.62 to 2139.5) | 154.11 (68.45 to 351.53) | 1747.46 (794.92 to 4077.16) | 203.12 (92.4 to 473.92) | 0.84 (0.63 to 1.06) | <0.001 |
| Republic of Angola | 46.06 (8.77 to 144.12) | 3.27 (0.62 to 10.24) | 164.97 (29.27 to 494.85) | 3.62 (0.64 to 10.87) | 0.4 (0.18 to 0.62) | 0.001 | 2327.83 (988.06 to 5551.51) | 165.45 (70.23 to 394.57) | 7912.74 (3251.96 to 18674.17) | 173.76 (71.41 to 410.08) | 0.23 (0.06 to 0.41) | 0.011 |
| Republic of Armenia | 25.75 (4.96 to 79.06) | 3.41 (0.66 to 10.47) | 34.48 (6.69 to 105.58) | 3.71 (0.72 to 11.36) | 0.41 (-0.32 to 1.14) | 0.266 | 1374.57 (625.9 to 3083.16) | 181.96 (82.85 to 408.13) | 1789.38 (801.43 to 4015.54) | 192.52 (86.23 to 432.03) | 0.4 (-0.23 to 1.04) | 0.202 |
| Republic of Austria | 65.05 (12.4 to 200.84) | 2.88 (0.55 to 8.88) | 92.52 (17.78 to 283.87) | 2.94 (0.56 to 9.02) | 0.02 (-0.14 to 0.18) | 0.8 | 3349.71 (1501.17 to 7622.38) | 148.08 (66.36 to 336.96) | 4789.75 (2165.97 to 11029.02) | 152.16 (68.81 to 350.36) | 0 (-0.15 to 0.15) | 0.984 |
| Republic of Azerbaijan | 44.31 (8.4 to 136.65) | 3.22 (0.61 to 9.93) | 100.35 (19.16 to 305.95) | 3.13 (0.6 to 9.55) | -0.82 (-1.67 to 0.04) | 0.061 | 2378.81 (1086.34 to 5336.47) | 172.78 (78.9 to 387.61) | 5359.73 (2389.83 to 12067.56) | 167.25 (74.57 to 376.57) | -0.59 (-1.34 to 0.16) | 0.119 |
| Republic of Belarus | 104.04 (19.97 to 323.34) | 3.47 (0.67 to 10.79) | 122.37 (23.52 to 378.74) | 3.68 (0.71 to 11.4) | 0.18 (-0.32 to 0.68) | 0.466 | 5455.18 (2482.72 to 12320.35) | 181.98 (82.82 to 411) | 6286.36 (2799.79 to 14341.67) | 189.3 (84.31 to 431.87) | 0.19 (-0.25 to 0.64) | 0.378 |
| Republic of Benin | 14.97 (2.86 to 46.94) | 2.65 (0.51 to 8.31) | 40.58 (7.71 to 126.88) | 2.25 (0.43 to 7.03) | -0.6 (-0.73 to -0.47) | <0.001 | 734.63 (312.74 to 1743.4) | 130.14 (55.4 to 308.84) | 2032.08 (830.6 to 4836.15) | 112.52 (45.99 to 267.78) | -0.5 (-0.59 to -0.4) | <0.001 |
| Republic of Botswana | 5.32 (0.95 to 16.54) | 3.09 (0.56 to 9.62) | 13.99 (2.53 to 44.13) | 2.67 (0.48 to 8.4) | -0.3 (-0.48 to -0.11) | 0.003 | 267.61 (113.27 to 637.27) | 155.6 (65.86 to 370.53) | 707.71 (296.99 to 1689.4) | 134.8 (56.57 to 321.77) | -0.27 (-0.43 to -0.11) | 0.002 |
| Republic of Bulgaria | 96.91 (18.48 to 299.44) | 3.43 (0.65 to 10.59) | 79.31 (15.76 to 242.72) | 3.25 (0.65 to 9.96) | 0.05 (-0.11 to 0.21) | 0.506 | 5033.86 (2303.67 to 11376.28) | 178 (81.46 to 402.27) | 4138.83 (1849.83 to 9323.97) | 169.77 (75.88 to 382.46) | 0.06 (-0.08 to 0.19) | 0.393 |
| Republic of Burundi | 18.27 (3.39 to 58.83) | 2.69 (0.5 to 8.67) | 49.74 (9.2 to 149.69) | 2.81 (0.52 to 8.46) | 0.42 (0.11 to 0.73) | 0.009 | 964.63 (424.08 to 2277.61) | 142.09 (62.47 to 335.49) | 2489.36 (1040.55 to 5867.74) | 140.77 (58.84 to 331.81) | 0.26 (-0.01 to 0.52) | 0.057 |
| Republic of Cabo Verde | 1.42 (0.26 to 4.2) | 3.17 (0.59 to 9.39) | 3.53 (0.67 to 11.39) | 2.68 (0.51 to 8.65) | -1.23 (-1.98 to -0.48) | 0.002 | 71.98 (31.56 to 165.71) | 160.99 (70.58 to 370.61) | 173.98 (73.59 to 416.18) | 132.07 (55.86 to 315.92) | -1.09 (-1.75 to -0.44) | 0.002 |
| Republic of Cameroon | 41.1 (8.16 to 131.21) | 2.85 (0.57 to 9.11) | 107.15 (18.55 to 342.47) | 2.35 (0.41 to 7.51) | -0.64 (-0.86 to -0.43) | <0.001 | 1945.23 (801.22 to 4770.74) | 135.01 (55.61 to 331.12) | 5159.52 (2070.25 to 12783.04) | 113.2 (45.42 to 280.47) | -0.57 (-0.74 to -0.4) | <0.001 |
| Republic of Chad | 18.45 (3.48 to 56.89) | 2.42 (0.46 to 7.46) | 47.97 (8.63 to 150.44) | 2.36 (0.42 to 7.4) | -0.06 (-0.13 to 0.01) | 0.078 | 935.61 (407.5 to 2207.68) | 122.76 (53.47 to 289.66) | 2322.3 (950.56 to 5659.93) | 114.23 (46.76 to 278.41) | -0.21 (-0.27 to -0.15) | <0.001 |
| Republic of Chile | 66.18 (12.67 to 206.39) | 2.41 (0.46 to 7.51) | 165.91 (32.09 to 515.24) | 2.89 (0.56 to 8.97) | 0.5 (0.24 to 0.76) | <0.001 | 3668.45 (1717.13 to 8214.76) | 133.52 (62.5 to 298.99) | 8894.65 (4061.79 to 19977.13) | 154.84 (70.71 to 347.76) | 0.44 (0.22 to 0.66) | <0.001 |
| Republic of Colombia | 138.2 (26.14 to 433.59) | 2.54 (0.48 to 7.98) | 433.83 (82.78 to 1332.26) | 3.21 (0.61 to 9.86) | 0.81 (0.6 to 1.03) | <0.001 | 7357.69 (3271.65 to 16696.07) | 135.36 (60.19 to 307.15) | 22449.18 (9799.54 to 50508.75) | 166.1 (72.51 to 373.71) | 0.73 (0.55 to 0.91) | <0.001 |
| Republic of Costa Rica | 13.01 (2.5 to 40.52) | 2.66 (0.51 to 8.28) | 42.86 (8.33 to 132.34) | 3.18 (0.62 to 9.83) | 0.73 (0.43 to 1.03) | <0.001 | 687.43 (302.78 to 1593.83) | 140.53 (61.9 to 325.83) | 2218.13 (993.48 to 5162.56) | 164.73 (73.78 to 383.39) | 0.68 (0.43 to 0.93) | <0.001 |
| Republic of Croatia | 53.42 (10.28 to 166.66) | 3.44 (0.66 to 10.73) | 51.75 (10.06 to 159.45) | 3.53 (0.69 to 10.87) | 0.11 (-0.11 to 0.32) | 0.322 | 2809.2 (1280.51 to 6350.14) | 180.94 (82.48 to 409) | 2720.65 (1235.49 to 6089.81) | 185.42 (84.2 to 415.04) | 0.17 (-0.02 to 0.36) | 0.085 |
| Republic of Cuba | 61.11 (11.49 to 189.75) | 2.41 (0.45 to 7.48) | 124.72 (24.55 to 391.69) | 3.02 (0.59 to 9.47) | 0.23 (0.03 to 0.43) | 0.023 | 3180.74 (1438.07 to 7290.02) | 125.45 (56.72 to 287.53) | 6382.24 (2807.29 to 14855.79) | 154.31 (67.88 to 359.19) | 0.18 (-0.01 to 0.38) | 0.068 |
| Republic of Cyprus | 6.2 (1.16 to 19.28) | 3.2 (0.6 to 9.94) | 12.11 (2.4 to 36.44) | 2.76 (0.55 to 8.3) | -0.42 (-0.53 to -0.32) | <0.001 | 298.43 (126.7 to 706.53) | 153.78 (65.29 to 364.07) | 619.95 (269.61 to 1427.1) | 141.21 (61.41 to 325.06) | -0.18 (-0.26 to -0.11) | <0.001 |
| Population of Côte d ivoire | 33.57 (6.36 to 108.46) | 2.21 (0.42 to 7.14) | 93.97 (16.4 to 298.4) | 2.16 (0.38 to 6.86) | -0.09 (-0.29 to 0.11) | 0.356 | 1664.82 (703.63 to 4104.62) | 109.63 (46.34 to 270.3) | 4611.71 (1882.1 to 11434.5) | 106.01 (43.26 to 262.84) | -0.11 (-0.26 to 0.03) | 0.111 |
| Republic of Djibouti | 1.45 (0.27 to 4.65) | 2.56 (0.48 to 8.17) | 7.73 (1.28 to 23.49) | 2.92 (0.48 to 8.86) | 0.43 (0.34 to 0.52) | <0.001 | 74.94 (32.31 to 179.76) | 131.86 (56.86 to 316.29) | 378.53 (155.98 to 891.54) | 142.85 (58.86 to 336.45) | 0.27 (0.21 to 0.34) | <0.001 |
| Republic of Ecuador | 34.14 (6.51 to 105.69) | 2.18 (0.41 to 6.74) | 99.77 (18.76 to 315.03) | 2.34 (0.44 to 7.38) | 0.37 (0.28 to 0.46) | <0.001 | 1785.13 (785.1 to 4140.35) | 113.84 (50.07 to 264.05) | 5186.36 (2273.95 to 12087.7) | 121.46 (53.25 to 283.08) | 0.34 (0.27 to 0.41) | <0.001 |
| Republic of El Salvador | 23.14 (4.3 to 70.4) | 2.85 (0.53 to 8.66) | 42.89 (7.95 to 133.77) | 2.89 (0.54 to 9.02) | 0 (-0.05 to 0.06) | 0.884 | 1188.62 (536.25 to 2737.13) | 146.26 (65.98 to 336.79) | 2233.53 (977.78 to 5202.1) | 150.66 (65.96 to 350.91) | 0.07 (0.03 to 0.1) | 0.001 |
| Republic of Equatorial Guinea | 2.33 (0.43 to 7.29) | 3.78 (0.7 to 11.84) | 6.21 (0.99 to 18.91) | 3.13 (0.5 to 9.55) | -0.61 (-0.67 to -0.55) | <0.001 | 114.29 (48.15 to 274.59) | 185.74 (78.26 to 446.28) | 305.59 (125.35 to 731.55) | 154.29 (63.29 to 369.37) | -0.59 (-0.63 to -0.54) | <0.001 |
| Republic of Estonia | 15.02 (2.87 to 47.49) | 3.19 (0.61 to 10.1) | 13.95 (2.81 to 41.25) | 3.25 (0.65 to 9.6) | 0.08 (-0.05 to 0.21) | 0.204 | 789.82 (362.24 to 1813.37) | 167.94 (77.03 to 385.59) | 740.79 (332.13 to 1597.92) | 172.38 (77.29 to 371.82) | 0.15 (0.05 to 0.26) | 0.005 |
| Republic of Fiji | 3.7 (0.69 to 11.44) | 2.82 (0.52 to 8.72) | 7.53 (1.35 to 23.24) | 3.15 (0.57 to 9.73) | 0.43 (0.28 to 0.59) | <0.001 | 187.69 (79.49 to 441.6) | 143.16 (60.63 to 336.81) | 387.51 (167.99 to 891.57) | 162.28 (70.35 to 373.36) | 0.49 (0.36 to 0.61) | <0.001 |
| Republic of Finland | 44.7 (8.69 to 139.99) | 2.88 (0.56 to 9.03) | 57.45 (11.24 to 173.88) | 3.32 (0.65 to 10.04) | 0.94 (0.74 to 1.14) | <0.001 | 2229.92 (969.43 to 5222.08) | 143.9 (62.56 to 337) | 2820.76 (1236.91 to 6584.87) | 162.87 (71.42 to 380.2) | 0.8 (0.64 to 0.96) | <0.001 |
| Republic of Ghana | 45.39 (8.24 to 145.2) | 2.19 (0.4 to 7) | 136.38 (25.27 to 411.21) | 2.33 (0.43 to 7.02) | 0.17 (-0.02 to 0.37) | 0.083 | 2286.21 (964.4 to 5495.33) | 110.2 (46.49 to 264.88) | 6573.82 (2683.43 to 15414.31) | 112.19 (45.8 to 263.07) | 0.07 (-0.08 to 0.22) | 0.318 |
| Republic of Guatemala | 30.44 (5.67 to 93.36) | 2.72 (0.51 to 8.36) | 84.67 (16.43 to 259.28) | 2.73 (0.53 to 8.35) | 0.2 (0.11 to 0.3) | <0.001 | 1579.9 (699.78 to 3627.24) | 141.4 (62.63 to 324.64) | 4405.3 (1927.34 to 10191.29) | 141.8 (62.04 to 328.04) | 0.2 (0.12 to 0.28) | <0.001 |
| Republic of Guinea | 23.71 (4.41 to 72.86) | 2.61 (0.49 to 8.01) | 44.27 (7.7 to 138.16) | 2.47 (0.43 to 7.71) | -0.16 (-0.19 to -0.13) | <0.001 | 1179.77 (496.91 to 2764.38) | 129.69 (54.62 to 303.88) | 2147.89 (881.12 to 5232.3) | 119.89 (49.18 to 292.05) | -0.23 (-0.25 to -0.21) | <0.001 |
| Republic of Guinea-Bissau | 3.37 (0.65 to 10.91) | 2.69 (0.52 to 8.73) | 6.93 (1.27 to 21.67) | 2.51 (0.46 to 7.86) | -0.14 (-0.18 to -0.1) | <0.001 | 158.78 (62.74 to 401.59) | 127 (50.18 to 321.21) | 322.8 (127.92 to 800.17) | 117.13 (46.42 to 290.35) | -0.18 (-0.22 to -0.15) | <0.001 |
| Republic of Guyana | 2.38 (0.48 to 7.43) | 2.05 (0.41 to 6.39) | 5.02 (0.95 to 15.89) | 2.63 (0.5 to 8.33) | 1.03 (0.79 to 1.27) | <0.001 | 127.15 (56.63 to 295.92) | 109.48 (48.76 to 254.81) | 256.5 (110.42 to 610.87) | 134.54 (57.91 to 320.4) | 0.87 (0.66 to 1.09) | <0.001 |
| Republic of Haiti | 28.17 (5.36 to 84.84) | 2.83 (0.54 to 8.51) | 62.06 (11.14 to 199.38) | 2.47 (0.44 to 7.92) | -0.29 (-0.41 to -0.17) | <0.001 | 1394.72 (596.9 to 3263.84) | 139.92 (59.88 to 327.43) | 3085.08 (1298.75 to 7547.98) | 122.63 (51.62 to 300.02) | -0.29 (-0.39 to -0.18) | <0.001 |
| Republic of Honduras | 19.98 (3.78 to 61.48) | 3.24 (0.61 to 9.97) | 70.3 (13.22 to 219.71) | 3.64 (0.69 to 11.39) | 0.53 (0.47 to 0.59) | <0.001 | 977.2 (408.51 to 2335.31) | 158.48 (66.25 to 378.74) | 3271.04 (1335.53 to 8084.34) | 169.53 (69.22 to 418.99) | 0.37 (0.32 to 0.42) | <0.001 |
| Republic of Iceland | 1.68 (0.32 to 5.08) | 2.82 (0.54 to 8.52) | 2.99 (0.59 to 8.93) | 2.77 (0.54 to 8.28) | 0.3 (0.01 to 0.59) | 0.046 | 87.72 (39.85 to 197.26) | 147.18 (66.87 to 330.99) | 158.77 (73.11 to 353.3) | 147.08 (67.73 to 327.28) | 0.32 (0.08 to 0.56) | 0.011 |
| Republic of India | 3447.38 (648.4 to 11180.89) | 2.24 (0.42 to 7.27) | 9377.68 (1754.41 to 29008.73) | 2.75 (0.51 to 8.5) | 0.57 (0.45 to 0.68) | <0.001 | 173311.09 (75874.53 to 418926.37) | 112.65 (49.32 to 272.3) | 444700.15 (187816.48 to 1080711.66) | 130.35 (55.05 to 316.78) | 0.37 (0.27 to 0.48) | <0.001 |
| Republic of Indonesia | 956.73 (181.55 to 3081.75) | 2.95 (0.56 to 9.5) | 2667.84 (505.37 to 8183.37) | 3.32 (0.63 to 10.19) | 0.17 (0.02 to 0.32) | 0.03 | 53571.79 (24859.7 to 122914.11) | 165.07 (76.6 to 378.72) | 142560.96 (65032.28 to 318496.01) | 177.59 (81.01 to 396.75) | 0.09 (-0.05 to 0.23) | 0.209 |
| Republic of Iraq | 72.83 (13.17 to 232.44) | 3.06 (0.55 to 9.78) | 232.59 (43.79 to 740.73) | 2.69 (0.51 to 8.57) | -0.47 (-0.53 to -0.42) | <0.001 | 3730.39 (1621.9 to 8872.85) | 156.99 (68.26 to 373.4) | 12133 (5265.57 to 28550.31) | 140.35 (60.91 to 330.25) | -0.4 (-0.44 to -0.35) | <0.001 |
| Republic of Italy | 665.27 (128.84 to 2106.36) | 3.76 (0.73 to 11.92) | 775.18 (151.58 to 2364.74) | 3.46 (0.68 to 10.56) | -0.45 (-0.51 to -0.38) | <0.001 | 34384.29 (15810.12 to 79045.22) | 194.51 (89.44 to 447.15) | 39733.97 (17936.63 to 91278.79) | 177.45 (80.1 to 407.65) | -0.44 (-0.5 to -0.38) | <0.001 |
| Republic of Kazakhstan | 102.38 (19.26 to 321.76) | 2.95 (0.56 to 9.27) | 145.83 (28.44 to 451.2) | 2.83 (0.55 to 8.75) | -0.44 (-0.85 to -0.02) | 0.04 | 5453.84 (2494.15 to 12460.39) | 157.18 (71.88 to 359.12) | 7806.55 (3516.02 to 17574.67) | 151.37 (68.18 to 340.78) | -0.25 (-0.61 to 0.1) | 0.157 |
| Republic of Kenya | 72.8 (13.36 to 220.4) | 2.8 (0.51 to 8.46) | 248.4 (45.28 to 718.68) | 3.07 (0.56 to 8.88) | 0.45 (0.36 to 0.54) | <0.001 | 3850.43 (1740.13 to 8566.43) | 147.88 (66.83 to 329.01) | 12337.78 (5296.17 to 27487.44) | 152.49 (65.46 to 339.74) | 0.21 (0.14 to 0.29) | <0.001 |
| Republic of Kiribati | 0.39 (0.07 to 1.18) | 3.3 (0.62 to 10.06) | 0.92 (0.16 to 2.8) | 3.71 (0.66 to 11.31) | 0.41 (0.12 to 0.7) | 0.008 | 18.89 (8.01 to 45.46) | 161.31 (68.38 to 388.23) | 44.41 (18.7 to 104.71) | 179.16 (75.43 to 422.4) | 0.37 (0.13 to 0.62) | 0.004 |
| Republic of Korea | 317.36 (62.68 to 984.57) | 3.29 (0.65 to 10.2) | 855.06 (170.69 to 2529.86) | 4.07 (0.81 to 12.05) | 0.56 (0.26 to 0.86) | 0.001 | 15942.89 (6916.61 to 37671.85) | 165.2 (71.67 to 390.35) | 44608.08 (20310.26 to 98079.57) | 212.44 (96.72 to 467.09) | 0.66 (0.4 to 0.93) | <0.001 |
| Republic of Latvia | 26.3 (5.01 to 79.96) | 3.22 (0.61 to 9.78) | 21.8 (4.25 to 64.47) | 3.39 (0.66 to 10.04) | -0.02 (-0.13 to 0.1) | 0.756 | 1381.39 (624.35 to 3082.68) | 168.9 (76.34 to 376.92) | 1157.73 (519.29 to 2493.93) | 180.27 (80.86 to 388.32) | 0.09 (-0.02 to 0.2) | 0.097 |
| Republic of Liberia | 7.68 (1.45 to 24.88) | 2.47 (0.47 to 8.01) | 16.51 (2.88 to 52.91) | 1.86 (0.32 to 5.94) | -0.95 (-1.13 to -0.76) | <0.001 | 376.37 (158.69 to 919.39) | 121.18 (51.09 to 296.01) | 821.09 (331.18 to 2051.28) | 92.25 (37.21 to 230.47) | -0.9 (-1.04 to -0.77) | <0.001 |
| Republic of Lithuania | 34.55 (6.64 to 105.81) | 3.27 (0.63 to 10.02) | 34.45 (6.66 to 103.96) | 3.59 (0.69 to 10.85) | -0.11 (-0.33 to 0.1) | 0.287 | 1796.67 (802.88 to 4057.4) | 170.2 (76.06 to 384.37) | 1801.31 (810.04 to 3960.58) | 187.95 (84.52 to 413.24) | 0.02 (-0.18 to 0.23) | 0.811 |
| Republic of Madagascar | 38.47 (7.11 to 119.96) | 2.5 (0.46 to 7.78) | 99.4 (18.79 to 315.81) | 2.25 (0.42 to 7.14) | -0.27 (-0.54 to 0) | 0.048 | 2101.6 (934.5 to 4669.86) | 136.36 (60.64 to 303.01) | 5361.17 (2343.37 to 12325.22) | 121.13 (52.95 to 278.48) | -0.29 (-0.51 to -0.06) | 0.016 |
| Republic of Malawi | 34.51 (6.71 to 109.02) | 2.79 (0.54 to 8.83) | 73.47 (13.59 to 225.18) | 2.82 (0.52 to 8.65) | 0.28 (0.17 to 0.4) | <0.001 | 1769.42 (786.87 to 4135.28) | 143.24 (63.7 to 334.77) | 3613.96 (1528.84 to 8572.63) | 138.88 (58.75 to 329.45) | 0.11 (0 to 0.21) | 0.044 |
| Republic of Maldives | 1.09 (0.21 to 3.48) | 3.6 (0.69 to 11.49) | 3.5 (0.67 to 10.42) | 2.59 (0.49 to 7.73) | -1.58 (-1.83 to -1.33) | <0.001 | 57.19 (25.49 to 135.35) | 188.76 (84.13 to 446.73) | 191.44 (86.33 to 419.49) | 142.05 (64.06 to 311.26) | -1.31 (-1.52 to -1.09) | <0.001 |
| Republic of Mali | 37.85 (7.27 to 118.43) | 3.01 (0.58 to 9.41) | 84.63 (15.54 to 254.24) | 2.83 (0.52 to 8.51) | -0.28 (-0.37 to -0.19) | <0.001 | 1774.74 (712.66 to 4334.12) | 141.05 (56.64 to 344.47) | 3952.31 (1544.46 to 9457.07) | 132.26 (51.68 to 316.48) | -0.27 (-0.33 to -0.2) | <0.001 |
| Republic of Malta | 2.73 (0.52 to 8.43) | 2.57 (0.49 to 7.94) | 4.62 (0.91 to 14.03) | 3.21 (0.63 to 9.72) | 1.11 (0.87 to 1.35) | <0.001 | 140.73 (61.29 to 318.97) | 132.7 (57.79 to 300.75) | 227.45 (98.11 to 533.09) | 157.65 (68 to 369.48) | 0.94 (0.73 to 1.15) | <0.001 |
| Republic of Mauritius | 6.95 (1.31 to 21.83) | 3.29 (0.62 to 10.34) | 17.41 (3.42 to 53.94) | 3.91 (0.77 to 12.13) | 1.1 (0.66 to 1.55) | <0.001 | 362.53 (160.52 to 831.06) | 171.67 (76.01 to 393.54) | 909.59 (415.28 to 2089.43) | 204.47 (93.35 to 469.69) | 1.09 (0.71 to 1.46) | <0.001 |
| Republic of Moldova | 35.01 (6.73 to 110.39) | 3.17 (0.61 to 9.99) | 43.89 (8.48 to 136.07) | 3.44 (0.66 to 10.66) | 0.44 (0.06 to 0.81) | 0.025 | 1827.76 (821.16 to 4182.69) | 165.42 (74.32 to 378.55) | 2252.41 (1015.45 to 5221.32) | 176.5 (79.57 to 409.16) | 0.45 (0.12 to 0.78) | 0.009 |
| Republic of Mozambique | 55.56 (10.74 to 173.77) | 2.8 (0.54 to 8.76) | 118.55 (21.71 to 369.89) | 2.95 (0.54 to 9.21) | 0.34 (0.28 to 0.41) | <0.001 | 2856.13 (1258.33 to 6758.2) | 143.9 (63.4 to 340.5) | 5747.62 (2338.95 to 13821.76) | 143.12 (58.24 to 344.17) | 0.1 (0.05 to 0.15) | <0.001 |
| Republic of Namibia | 5.83 (1.11 to 17.91) | 3 (0.57 to 9.23) | 14.3 (2.49 to 44.53) | 3.11 (0.54 to 9.69) | 0.14 (0.09 to 0.19) | <0.001 | 295.72 (128.61 to 681.38) | 152.36 (66.26 to 351.06) | 696.04 (286.26 to 1676.42) | 151.51 (62.31 to 364.92) | 0.01 (-0.03 to 0.06) | 0.561 |
| Republic of Nauru | 0.05 (0.01 to 0.17) | 3.32 (0.62 to 10.67) | 0.06 (0.01 to 0.2) | 3.19 (0.59 to 10.18) | 0.22 (0.02 to 0.42) | 0.036 | 2.59 (1.05 to 6.44) | 158.16 (64.09 to 393.44) | 3.09 (1.28 to 7.57) | 154.91 (64.11 to 379.69) | 0.24 (0.07 to 0.4) | 0.007 |
| Republic of Nicaragua | 12.08 (2.29 to 37.35) | 2.55 (0.48 to 7.89) | 37.8 (7.01 to 113.81) | 2.6 (0.48 to 7.84) | 0.11 (-0.02 to 0.23) | 0.096 | 642.61 (290.86 to 1463.27) | 135.67 (61.41 to 308.94) | 2017.39 (892.34 to 4538.52) | 138.97 (61.47 to 312.63) | 0.15 (0.04 to 0.26) | 0.011 |
| Republic of Niue | 0.02 (0 to 0.07) | 4.3 (0.81 to 13.42) | 0.02 (0 to 0.07) | 4.23 (0.82 to 13.54) | -0.24 (-0.32 to -0.16) | <0.001 | 0.98 (0.39 to 2.43) | 199.28 (79.29 to 495.58) | 1.08 (0.45 to 2.62) | 202.42 (84.55 to 493.75) | -0.11 (-0.18 to -0.04) | 0.004 |
| Republic of Palau | 0.1 (0.02 to 0.31) | 3.46 (0.65 to 10.93) | 0.25 (0.05 to 0.76) | 3.45 (0.63 to 10.37) | 0.47 (0.02 to 0.92) | 0.043 | 4.74 (2 to 11.67) | 164.72 (69.59 to 405.22) | 12.6 (5.39 to 29.13) | 172.75 (73.87 to 399.25) | 0.6 (0.22 to 0.98) | 0.003 |
| Republic of Panama | 10.61 (2.03 to 33.1) | 2.53 (0.48 to 7.88) | 31.59 (6 to 97.65) | 2.87 (0.55 to 8.88) | 0.41 (0.35 to 0.47) | <0.001 | 565.27 (253.81 to 1264.61) | 134.6 (60.44 to 301.14) | 1660.17 (728.41 to 3748.38) | 151.05 (66.28 to 341.06) | 0.35 (0.29 to 0.41) | <0.001 |
| Republic of Paraguay | 22.88 (4.56 to 70.2) | 3.66 (0.73 to 11.24) | 63.17 (12.02 to 194.76) | 3.9 (0.74 to 12.02) | 0.39 (0.24 to 0.53) | <0.001 | 1147.12 (502.76 to 2659.4) | 183.61 (80.47 to 425.67) | 3107.81 (1322.58 to 7258.03) | 191.78 (81.61 to 447.88) | 0.32 (0.19 to 0.45) | <0.001 |
| Republic of Peru | 80.59 (15.17 to 245.99) | 2.29 (0.43 to 6.98) | 213.21 (41.17 to 655.75) | 2.36 (0.46 to 7.27) | 0.08 (0 to 0.16) | 0.054 | 4146.25 (1804.8 to 9509.6) | 117.65 (51.21 to 269.83) | 10939.04 (4770.83 to 25202.75) | 121.22 (52.87 to 279.28) | 0.08 (0.02 to 0.14) | 0.011 |
| Republic of Poland | 355.16 (68.16 to 1133.87) | 3.46 (0.66 to 11.04) | 473.59 (92.23 to 1465.15) | 3.62 (0.71 to 11.2) | 0.89 (0.49 to 1.29) | <0.001 | 18507.65 (8520.92 to 42553.29) | 180.16 (82.95 to 414.23) | 24272.88 (11079.9 to 54898.22) | 185.55 (84.7 to 419.67) | 0.79 (0.46 to 1.13) | <0.001 |
| Republic of Rwanda | 26.98 (5.29 to 84.3) | 3.17 (0.62 to 9.9) | 73.76 (13.16 to 222.47) | 3.39 (0.6 to 10.22) | 0.61 (0.27 to 0.95) | 0.001 | 1354.44 (582.49 to 3234.04) | 159.13 (68.44 to 379.96) | 3538.32 (1425.14 to 8320.5) | 162.53 (65.46 to 382.19) | 0.5 (0.2 to 0.8) | 0.002 |
| Republic of San Marino | 0.18 (0.03 to 0.55) | 2.57 (0.5 to 7.99) | 0.31 (0.05 to 0.93) | 2.49 (0.43 to 7.54) | -0.27 (-0.4 to -0.13) | <0.001 | 9.73 (4.47 to 21.52) | 141.65 (65.12 to 313.18) | 16.68 (7.35 to 36.48) | 135.34 (59.63 to 295.9) | -0.34 (-0.47 to -0.21) | <0.001 |
| Republic of Senegal | 25.22 (4.84 to 78.81) | 2.6 (0.5 to 8.12) | 72.95 (12.83 to 230.12) | 2.96 (0.52 to 9.32) | 0.57 (0.39 to 0.75) | <0.001 | 1233.18 (516.54 to 2966.91) | 127.03 (53.21 to 305.62) | 3360.08 (1315.48 to 8553.53) | 136.12 (53.29 to 346.51) | 0.35 (0.21 to 0.48) | <0.001 |
| Republic of Serbia | 104.74 (20.22 to 324.98) | 3.55 (0.69 to 11.03) | 104.1 (20.27 to 317.26) | 3.51 (0.68 to 10.68) | 0.17 (0 to 0.33) | 0.048 | 5352.74 (2388.21 to 12438.67) | 181.61 (81.03 to 422.03) | 5346.73 (2414.26 to 11906.84) | 180.03 (81.29 to 400.93) | 0.19 (0.05 to 0.34) | 0.011 |
| Republic of Seychelles | 0.52 (0.1 to 1.63) | 4.06 (0.77 to 12.78) | 1.17 (0.22 to 3.65) | 3.37 (0.63 to 10.55) | -0.77 (-1.26 to -0.27) | 0.004 | 26.14 (11.33 to 61.3) | 204.64 (88.69 to 479.84) | 61.9 (27.75 to 139.82) | 179.03 (80.28 to 404.43) | -0.55 (-0.99 to -0.1) | 0.017 |
| Republic of Sierra Leone | 12.05 (2.26 to 39.08) | 2.11 (0.4 to 6.84) | 26.59 (4.85 to 81.68) | 2.07 (0.38 to 6.36) | -0.04 (-0.21 to 0.13) | 0.639 | 632.08 (281.95 to 1483.96) | 110.56 (49.32 to 259.57) | 1347.37 (565.36 to 3162.6) | 104.84 (43.99 to 246.08) | -0.18 (-0.31 to -0.06) | 0.006 |
| Republic of Singapore | 14.89 (2.84 to 47.06) | 2.07 (0.4 to 6.54) | 49.07 (9.59 to 149.5) | 2.21 (0.43 to 6.72) | 0.68 (0.43 to 0.92) | <0.001 | 794.22 (356.89 to 1841.52) | 110.38 (49.6 to 255.94) | 2689.78 (1268.28 to 5978.84) | 120.99 (57.05 to 268.93) | 0.66 (0.44 to 0.88) | <0.001 |
| Republic of Slovenia | 19.03 (3.64 to 57.71) | 3.29 (0.63 to 9.98) | 24.56 (4.81 to 72.34) | 3.27 (0.64 to 9.63) | 0.14 (-0.07 to 0.35) | 0.174 | 985.57 (450.69 to 2234.93) | 170.44 (77.94 to 386.5) | 1307.14 (608.89 to 2813.22) | 174.05 (81.08 to 374.59) | 0.27 (0.08 to 0.45) | 0.007 |
| Republic of South Africa | 166.63 (31.06 to 525.83) | 2.76 (0.51 to 8.7) | 420.17 (79.41 to 1323.74) | 3.06 (0.58 to 9.64) | 0.51 (0.35 to 0.66) | <0.001 | 9056.59 (4228.49 to 20520.5) | 149.88 (69.98 to 339.61) | 21749.6 (9807.11 to 50916.92) | 158.33 (71.39 to 370.67) | 0.36 (0.21 to 0.52) | <0.001 |
| Republic of South Sudan | 20.28 (3.79 to 62.64) | 2.77 (0.52 to 8.56) | 41.16 (7.05 to 125.41) | 2.68 (0.46 to 8.16) | -0.32 (-0.53 to -0.11) | 0.005 | 1039.18 (440.03 to 2435.44) | 141.95 (60.11 to 332.68) | 2077.22 (861.98 to 4820.59) | 135.19 (56.1 to 313.73) | -0.34 (-0.52 to -0.16) | 0.001 |
| Republic of Sudan | 81.24 (14.68 to 259.68) | 2.94 (0.53 to 9.41) | 178.85 (31.56 to 559.11) | 2.57 (0.45 to 8.04) | -0.6 (-0.66 to -0.54) | <0.001 | 4234.61 (1847.12 to 10118.01) | 153.46 (66.94 to 366.67) | 9448.49 (4136.25 to 21868.99) | 135.85 (59.47 to 314.44) | -0.54 (-0.59 to -0.48) | <0.001 |
| Republic of Suriname | 1.93 (0.38 to 5.89) | 2.61 (0.51 to 7.96) | 4.77 (0.91 to 13.99) | 2.82 (0.53 to 8.26) | -0.16 (-0.52 to 0.19) | 0.358 | 102.62 (46.43 to 229.03) | 138.68 (62.75 to 309.52) | 247.72 (110.78 to 543.1) | 146.15 (65.36 to 320.41) | -0.15 (-0.47 to 0.16) | 0.332 |
| Republic of Tajikistan | 24.13 (4.56 to 74.61) | 3.31 (0.62 to 10.23) | 62.5 (11.98 to 198.97) | 3.07 (0.59 to 9.77) | -0.67 (-1.37 to 0.04) | 0.065 | 1259.07 (568.32 to 2827.88) | 172.64 (77.93 to 387.76) | 3204.6 (1388.78 to 7530.62) | 157.34 (68.19 to 369.74) | -0.57 (-1.19 to 0.06) | 0.076 |
| Republic of Trinidad and Tobago | 4.96 (0.93 to 15.49) | 2.23 (0.42 to 6.95) | 13.59 (2.53 to 43.45) | 3.06 (0.57 to 9.77) | 1.42 (1.17 to 1.66) | <0.001 | 265.76 (120.44 to 607.36) | 119.2 (54.02 to 272.42) | 688.07 (296.68 to 1629.21) | 154.7 (66.7 to 366.29) | 1.19 (0.98 to 1.4) | <0.001 |
| Republic of Tunisia | 54.66 (10.09 to 165.69) | 3.89 (0.72 to 11.79) | 129.88 (23.82 to 408.5) | 3.61 (0.66 to 11.36) | -0.31 (-0.67 to 0.05) | 0.087 | 2817.76 (1258.17 to 6332.11) | 200.54 (89.55 to 450.67) | 6692.63 (3002.23 to 15305.08) | 186.17 (83.51 to 425.74) | -0.23 (-0.55 to 0.09) | 0.156 |
| Republic of Turkey | 399.29 (76.23 to 1204.51) | 3.78 (0.72 to 11.41) | 828.82 (159.71 to 2651.96) | 3.31 (0.64 to 10.58) | -0.42 (-0.7 to -0.15) | 0.003 | 20733.69 (9258.42 to 46821.02) | 196.34 (87.67 to 443.37) | 44520.66 (20273.64 to 102318.62) | 177.64 (80.89 to 408.26) | -0.28 (-0.5 to -0.06) | 0.015 |
| Republic of Uganda | 47.17 (8.96 to 143.93) | 2.39 (0.45 to 7.3) | 142.41 (25.69 to 437.47) | 2.7 (0.49 to 8.31) | 0.13 (0.01 to 0.25) | 0.032 | 2584.38 (1139.43 to 5719.84) | 131.12 (57.81 to 290.2) | 7221.22 (2990.62 to 16989.22) | 137.1 (56.78 to 322.55) | -0.07 (-0.17 to 0.03) | 0.185 |
| Republic of Uzbekistan | 94.81 (17.98 to 295.66) | 3.21 (0.61 to 10.01) | 243.58 (46.3 to 748.59) | 2.83 (0.54 to 8.71) | -0.47 (-1.11 to 0.17) | 0.141 | 4913.33 (2226.64 to 11200.82) | 166.42 (75.42 to 379.39) | 12743.03 (5518.22 to 28829.66) | 148.31 (64.22 to 335.53) | -0.33 (-0.89 to 0.23) | 0.236 |
| Republic of Vanuatu | 0.64 (0.11 to 1.96) | 2.99 (0.54 to 9.26) | 1.88 (0.35 to 5.81) | 3.15 (0.59 to 9.76) | 0.31 (0.1 to 0.51) | 0.005 | 31.29 (12.87 to 74.43) | 147.44 (60.67 to 350.73) | 91.29 (37.39 to 221.32) | 153.24 (62.76 to 371.5) | 0.26 (0.12 to 0.4) | 0.001 |
| Republic of Yemen | 52.68 (9.63 to 166.21) | 3.3 (0.6 to 10.43) | 138.07 (23.88 to 435.91) | 2.72 (0.47 to 8.58) | -0.4 (-0.5 to -0.3) | <0.001 | 2696.97 (1174.63 to 6434.51) | 169.17 (73.68 to 403.62) | 7135.02 (3083.83 to 16593.49) | 140.45 (60.71 to 326.64) | -0.39 (-0.48 to -0.31) | <0.001 |
| Republic of Zambia | 25.58 (4.91 to 80.87) | 2.69 (0.52 to 8.5) | 70.19 (11.77 to 215.65) | 2.6 (0.44 to 8) | -0.22 (-0.28 to -0.16) | <0.001 | 1328.15 (581.05 to 3160.19) | 139.64 (61.09 to 332.26) | 3518.35 (1462.41 to 8225.55) | 130.49 (54.24 to 305.08) | -0.32 (-0.36 to -0.27) | <0.001 |
| Republic of Zimbabwe | 36.35 (7.15 to 108.64) | 2.91 (0.57 to 8.7) | 68.86 (12.09 to 210.98) | 2.79 (0.49 to 8.54) | 0.18 (0.04 to 0.32) | 0.012 | 1844.3 (788.54 to 4310.3) | 147.67 (63.14 to 345.11) | 3412.08 (1409.7 to 8080.65) | 138.16 (57.08 to 327.19) | 0.07 (-0.05 to 0.2) | 0.234 |
| Republic of the Congo | 13.37 (2.56 to 40.93) | 4.13 (0.79 to 12.64) | 36.82 (6.5 to 116.11) | 3.43 (0.6 to 10.81) | -0.71 (-0.88 to -0.54) | <0.001 | 634.35 (260.45 to 1509.49) | 195.94 (80.45 to 466.26) | 1764.88 (712.95 to 4371.77) | 164.3 (66.37 to 406.98) | -0.65 (-0.8 to -0.5) | <0.001 |
| Republic of the Gambia | 2.63 (0.49 to 8.21) | 2.23 (0.42 to 6.98) | 8.22 (1.51 to 24.71) | 2.5 (0.46 to 7.52) | -0.08 (-0.34 to 0.19) | 0.571 | 130.8 (54.64 to 308.12) | 111.16 (46.44 to 261.86) | 386.24 (149.23 to 920.89) | 117.59 (45.43 to 280.37) | -0.13 (-0.32 to 0.05) | 0.145 |
| Republic of the Marshall Islands | 0.16 (0.03 to 0.5) | 3.19 (0.59 to 9.83) | 0.41 (0.07 to 1.28) | 3.19 (0.58 to 10.02) | 0.71 (0.39 to 1.03) | <0.001 | 7.62 (3.12 to 18.51) | 149.76 (61.29 to 363.78) | 19.65 (8.03 to 48.02) | 153.42 (62.69 to 374.84) | 0.7 (0.44 to 0.97) | <0.001 |
| Republic of the Niger | 22.76 (4.49 to 72.21) | 2.28 (0.45 to 7.24) | 72.71 (12.96 to 227.5) | 2.61 (0.47 to 8.17) | 0.4 (0.25 to 0.55) | <0.001 | 1133.19 (475.75 to 2714.21) | 113.67 (47.72 to 272.27) | 3530.21 (1475.33 to 8465.61) | 126.79 (52.99 to 304.05) | 0.32 (0.21 to 0.43) | <0.001 |
| Republic of the Philippines | 274.55 (51.51 to 865.58) | 2.82 (0.53 to 8.89) | 864.23 (164.2 to 2838.8) | 3.35 (0.64 to 10.99) | 0.62 (0.48 to 0.76) | <0.001 | 15438.82 (7206.05 to 34614.52) | 158.48 (73.97 to 355.31) | 45652.09 (20672.21 to 107838.9) | 176.74 (80.03 to 417.5) | 0.41 (0.29 to 0.53) | <0.001 |
| Republic of the Union of Myanmar | 229.32 (42.18 to 709.53) | 3.38 (0.62 to 10.46) | 515.83 (97.52 to 1601.5) | 3.55 (0.67 to 11.03) | 0.16 (-0.11 to 0.44) | 0.225 | 12476.47 (5647.13 to 27873.5) | 183.85 (83.21 to 410.73) | 26787.42 (11759.81 to 62075.24) | 184.55 (81.02 to 427.67) | 0.07 (-0.18 to 0.32) | 0.574 |
| Romania | 229.01 (43.91 to 708.59) | 3.4 (0.65 to 10.53) | 215.43 (42.25 to 691.94) | 3.19 (0.63 to 10.25) | 0.12 (-0.06 to 0.29) | 0.185 | 11865.5 (5382.36 to 27244.31) | 176.3 (79.97 to 404.79) | 11166.43 (5054.89 to 26123.9) | 165.35 (74.85 to 386.83) | 0.1 (-0.05 to 0.24) | 0.171 |
| Russian Federation | 1542.11 (291.2 to 4826.95) | 3.56 (0.67 to 11.14) | 1820.77 (352.74 to 5720.64) | 3.67 (0.71 to 11.54) | 0.43 (-0.1 to 0.96) | 0.111 | 80459.77 (36716.48 to 183208.06) | 185.73 (84.75 to 422.9) | 93695.94 (42891.3 to 215027.7) | 189.08 (86.56 to 433.93) | 0.41 (-0.02 to 0.85) | 0.062 |
| Saint Kitts and Nevis | 0.2 (0.04 to 0.63) | 3.27 (0.63 to 10.31) | 0.61 (0.12 to 1.93) | 2.95 (0.58 to 9.33) | 0.18 (-0.5 to 0.86) | 0.596 | 9.77 (4.23 to 23.25) | 160.47 (69.45 to 381.91) | 30.66 (13.19 to 72.41) | 148.17 (63.72 to 349.93) | 0.23 (-0.36 to 0.82) | 0.437 |
| Saint Lucia | 0.55 (0.1 to 1.66) | 2.64 (0.49 to 8.03) | 1.7 (0.33 to 5.16) | 2.77 (0.53 to 8.43) | 0.03 (-0.22 to 0.29) | 0.798 | 28.05 (12.33 to 62.96) | 135.73 (59.67 to 304.7) | 88.05 (38.67 to 200.44) | 143.97 (63.23 to 327.73) | 0.09 (-0.13 to 0.31) | 0.433 |
| Saint Vincent and the Grenadines | 0.48 (0.09 to 1.53) | 3.02 (0.58 to 9.66) | 1.05 (0.21 to 3.24) | 2.97 (0.58 to 9.2) | -0.03 (-0.43 to 0.38) | 0.889 | 23.99 (10.43 to 57.36) | 151.71 (65.94 to 362.76) | 53.24 (23.82 to 123) | 151.11 (67.61 to 349.11) | 0.04 (-0.32 to 0.4) | 0.817 |
| Slovak Republic | 46.19 (8.96 to 142.17) | 3.39 (0.66 to 10.44) | 63.57 (12.56 to 194.54) | 3.3 (0.65 to 10.11) | 0.62 (0.3 to 0.93) | <0.001 | 2337.34 (1036.14 to 5377.04) | 171.73 (76.13 to 395.05) | 3271.2 (1450.13 to 7437.2) | 169.96 (75.34 to 386.41) | 0.59 (0.33 to 0.85) | <0.001 |
| Socialist Republic of Viet Nam | 459.81 (82.33 to 1438.84) | 4.66 (0.83 to 14.59) | 1303.57 (245.92 to 4093.95) | 4.43 (0.84 to 13.9) | -0.21 (-0.79 to 0.36) | 0.451 | 22566.48 (9843.61 to 54260.12) | 228.79 (99.8 to 550.12) | 62568.81 (26202.43 to 152378.45) | 212.48 (88.98 to 517.47) | -0.21 (-0.72 to 0.31) | 0.417 |
| Solomon Islands | 1.54 (0.27 to 5.06) | 3.34 (0.58 to 10.98) | 3.8 (0.67 to 11.8) | 2.98 (0.53 to 9.26) | -0.62 (-0.85 to -0.38) | <0.001 | 76.18 (31.22 to 188.43) | 165.36 (67.77 to 409.01) | 188.18 (77.79 to 449.1) | 147.68 (61.04 to 352.44) | -0.57 (-0.73 to -0.41) | <0.001 |
| State of Eritrea | 12.34 (2.33 to 39.47) | 2.68 (0.51 to 8.57) | 33.83 (5.89 to 107.06) | 3.06 (0.53 to 9.7) | 0.26 (0.14 to 0.38) | <0.001 | 635.73 (274.37 to 1523.2) | 138.12 (59.61 to 330.94) | 1615.88 (641.75 to 3975.96) | 146.37 (58.13 to 360.14) | 0.04 (-0.05 to 0.13) | 0.341 |
| State of Israel | 27.36 (5.26 to 84.57) | 2.6 (0.5 to 8.04) | 61.82 (11.99 to 189.29) | 2.53 (0.49 to 7.74) | 0.49 (0.25 to 0.72) | <0.001 | 1432.21 (637.85 to 3243.93) | 136.2 (60.66 to 308.49) | 3202.54 (1424.16 to 7227.31) | 130.92 (58.22 to 295.45) | 0.38 (0.18 to 0.58) | 0.001 |
| State of Kuwait | 6.29 (1.18 to 19.42) | 2.21 (0.41 to 6.82) | 27.73 (5.42 to 85.1) | 1.83 (0.36 to 5.63) | -1.02 (-1.27 to -0.77) | <0.001 | 342.05 (153.78 to 774.11) | 120.04 (53.97 to 271.67) | 1621.72 (749.59 to 3493.3) | 107.33 (49.61 to 231.2) | -0.7 (-0.93 to -0.46) | <0.001 |
| State of Libya | 22.3 (4.15 to 69.28) | 3.82 (0.71 to 11.86) | 55.98 (10.09 to 173.73) | 2.76 (0.5 to 8.56) | -1.6 (-1.8 to -1.4) | <0.001 | 1091.98 (459.2 to 2577.86) | 186.9 (78.6 to 441.23) | 2882.65 (1249.17 to 6698.94) | 142.08 (61.57 to 330.18) | -1.35 (-1.52 to -1.17) | <0.001 |
| State of Qatar | 1.26 (0.22 to 3.91) | 1.6 (0.28 to 4.97) | 11.11 (1.97 to 34.19) | 1.4 (0.25 to 4.31) | -0.35 (-0.58 to -0.13) | 0.003 | 69.7 (29.97 to 158.15) | 88.62 (38.1 to 201.09) | 653.7 (290.32 to 1442.46) | 82.37 (36.58 to 181.76) | -0.14 (-0.32 to 0.05) | 0.14 |
| Sultanate of Oman | 6.85 (1.26 to 21.48) | 2.55 (0.47 to 7.99) | 21.29 (3.98 to 67.88) | 2.03 (0.38 to 6.46) | -0.59 (-0.7 to -0.47) | <0.001 | 351.59 (151.26 to 836.58) | 130.73 (56.24 to 311.05) | 1134.2 (498.04 to 2666.12) | 107.89 (47.38 to 253.61) | -0.44 (-0.54 to -0.34) | <0.001 |
| Swiss Confederation | 59.84 (11.59 to 188.39) | 2.88 (0.56 to 9.06) | 91.12 (17.63 to 274.14) | 2.93 (0.57 to 8.82) | 0.03 (-0.03 to 0.09) | 0.289 | 3061.99 (1351.78 to 7125.84) | 147.22 (64.99 to 342.6) | 4656.17 (2052.84 to 10460.21) | 149.78 (66.04 to 336.49) | 0.02 (-0.04 to 0.08) | 0.5 |
| Syrian Arab Republic | 51.14 (9.62 to 160.82) | 3.16 (0.59 to 9.92) | 131.58 (22.87 to 417.82) | 3.08 (0.54 to 9.79) | -0.27 (-0.6 to 0.06) | 0.1 | 2735 (1241.53 to 6290.19) | 168.74 (76.6 to 388.08) | 7010.17 (3137.76 to 16259.81) | 164.29 (73.54 to 381.07) | -0.23 (-0.52 to 0.06) | 0.109 |
| Taiwan (Province of China) | 122.74 (23.41 to 379.92) | 2.8 (0.53 to 8.67) | 293.88 (57.19 to 914.54) | 3.22 (0.63 to 10.01) | 0.68 (0.22 to 1.14) | 0.005 | 6024.49 (2591.23 to 14308.1) | 137.54 (59.16 to 326.66) | 14718.41 (6546.72 to 33877.14) | 161.09 (71.65 to 370.77) | 0.9 (0.5 to 1.31) | <0.001 |
| Togolese Republic | 9.5 (1.75 to 29.87) | 2.23 (0.41 to 7) | 34.64 (5.86 to 108.13) | 2.4 (0.41 to 7.49) | 0.1 (0 to 0.19) | 0.041 | 473.83 (202 to 1151.94) | 111.03 (47.33 to 269.93) | 1669.37 (680.42 to 4079.93) | 115.62 (47.12 to 282.57) | 0.02 (-0.05 to 0.09) | 0.59 |
| Tokelau | 0.02 (0 to 0.06) | 6.45 (1.26 to 19.72) | 0.01 (0 to 0.05) | 4.17 (0.82 to 13.13) | -0.83 (-1.11 to -0.54) | <0.001 | 0.83 (0.33 to 2.04) | 278.59 (109.28 to 683.34) | 0.68 (0.28 to 1.68) | 195.56 (80.37 to 482.42) | -0.6 (-0.84 to -0.36) | <0.001 |
| Turkmenistan | 16.37 (3.14 to 51.35) | 3.11 (0.6 to 9.76) | 35.63 (6.57 to 111.48) | 2.8 (0.52 to 8.77) | -0.49 (-1.09 to 0.11) | 0.108 | 861.03 (385.8 to 1958.75) | 163.61 (73.31 to 372.19) | 1878.11 (825.19 to 4286.8) | 147.83 (64.95 to 337.41) | -0.36 (-0.88 to 0.16) | 0.172 |
| Tuvalu | 0.08 (0.02 to 0.26) | 4.34 (0.86 to 13.65) | 0.12 (0.02 to 0.37) | 4.16 (0.8 to 13.17) | 0.18 (-0.02 to 0.38) | 0.079 | 3.83 (1.56 to 9.4) | 202.35 (82.68 to 496.85) | 5.53 (2.3 to 13.55) | 197.28 (81.93 to 483.74) | 0.22 (0.05 to 0.4) | 0.015 |
| Ukraine | 554.75 (105.67 to 1733.5) | 3.44 (0.66 to 10.76) | 570.62 (107.2 to 1748.7) | 3.68 (0.69 to 11.28) | 0.29 (-0.03 to 0.62) | 0.077 | 29635.21 (13625.02 to 67383.77) | 184 (84.59 to 418.36) | 29237.59 (12898.93 to 66168.88) | 188.59 (83.2 to 426.81) | 0.2 (-0.07 to 0.46) | 0.142 |
| Union of the Comoros | 1.93 (0.38 to 6.1) | 3.08 (0.6 to 9.71) | 5.35 (0.97 to 16.51) | 3.4 (0.62 to 10.49) | 0.08 (-0.03 to 0.19) | 0.14 | 96.24 (40.78 to 228.02) | 153.33 (64.97 to 363.27) | 255.49 (101.89 to 615.69) | 162.27 (64.72 to 391.05) | -0.03 (-0.11 to 0.06) | 0.567 |
| United Arab Emirates | 4.57 (0.81 to 14.65) | 1.5 (0.27 to 4.81) | 55.58 (10.06 to 172.74) | 1.35 (0.24 to 4.2) | -0.28 (-0.47 to -0.08) | 0.008 | 244.48 (103.23 to 590.54) | 80.3 (33.9 to 193.96) | 3166.16 (1361.73 to 7223.11) | 77 (33.12 to 175.66) | -0.14 (-0.31 to 0.03) | 0.11 |
| United Kingdom of Great Britain and Northern Ireland | 436.33 (83.37 to 1369.62) | 2.64 (0.51 to 8.3) | 578.17 (111.97 to 1778.01) | 2.65 (0.51 to 8.15) | 0.05 (-0.02 to 0.12) | 0.143 | 22394.53 (10081.73 to 51570.72) | 135.71 (61.09 to 312.51) | 29512.32 (13173.29 to 68515.75) | 135.33 (60.41 to 314.19) | 0 (-0.06 to 0.05) | 0.862 |
| United Mexican States | 294.91 (55.59 to 932.8) | 2.27 (0.43 to 7.17) | 856.92 (160.54 to 2657.08) | 2.44 (0.46 to 7.56) | 0.21 (0.11 to 0.31) | <0.001 | 16142.71 (7466.45 to 36241.84) | 124.05 (57.38 to 278.51) | 46209.82 (21275.29 to 104582.81) | 131.52 (60.55 to 297.66) | 0.18 (0.08 to 0.27) | 0.001 |
| United Republic of Tanzania | 106.22 (20.24 to 338.26) | 3.27 (0.62 to 10.43) | 264.17 (48.8 to 804.57) | 2.98 (0.55 to 9.07) | -0.43 (-0.49 to -0.37) | <0.001 | 5285.25 (2255.47 to 12779.48) | 162.91 (69.52 to 393.9) | 12906.17 (5251.39 to 30890.41) | 145.47 (59.19 to 348.17) | -0.48 (-0.53 to -0.42) | <0.001 |
| United States Virgin Islands | 0.66 (0.13 to 2.09) | 2.36 (0.45 to 7.48) | 1.01 (0.19 to 3.17) | 3.3 (0.63 to 10.3) | 1.18 (1.03 to 1.32) | <0.001 | 34.08 (14.65 to 82.25) | 121.81 (52.36 to 293.97) | 52.07 (22.9 to 117.83) | 169.39 (74.5 to 383.34) | 1.12 (1 to 1.23) | <0.001 |
| United States of America | 2447.45 (466.04 to 7792) | 3.78 (0.72 to 12.04) | 4864.92 (933.28 to 15175.38) | 4.64 (0.89 to 14.48) | 1.13 (0.86 to 1.4) | <0.001 | 130465.2 (61500.92 to 295793.17) | 201.56 (95.02 to 456.99) | 257051.42 (121365.21 to 579405.88) | 245.28 (115.81 to 552.88) | 1.04 (0.83 to 1.25) | <0.001 |

Note: CI, confidence interval; AAPC, average annual percentage change; EOAD, early-onset Alzheimer’s disease and other dementias; DALYs, disability-adjusted life years.

**Supplementary Table S11. Age-standardized prevalence and incidence of EOAD in men aged 40-64 years and their AAPCs from 1990 to 2021 at the country levels.**

|  | Prevalence | | | | | | Incidence | | | | | |
| --- | --- | --- | --- | --- | --- | --- | --- | --- | --- | --- | --- | --- |
| Country | No of people with EOAD in 1990 (000s) | Age standardised rate in 1990 (per 100 000) | No of people with EOAD in 2021 (000s) | Age standardised rate in 2021 (per 100 000) | AAPC (95% CI) | *p* value | No of people with EOAD in 1990 (000s) | Age standardised rate in 1990 (per 100 000) | No of people with EOAD in 2021 (000s) | Age standardised rate in 2021 (per 100 000) | AAPC (95% CI) | *p* value |
| American Samoa | 10.94 (7.89 to 14.57) | 265.57 (191.57 to 353.82) | 22.98 (16.75 to 30.4) | 309.27 (225.5 to 409.24) | 0.44 (0.32 to 0.57) | <0.001 | 2.01 (1.25 to 2.93) | 48.86 (30.33 to 71.25) | 4.1 (2.6 to 5.93) | 55.14 (34.95 to 79.86) | 0.29 (0.09 to 0.5) | 0.006 |
| Antigua and Barbuda | 13.36 (9.7 to 17.7) | 255.33 (185.46 to 338.25) | 38.84 (28.57 to 51.37) | 278.53 (204.91 to 368.39) | 0.43 (0.18 to 0.69) | 0.002 | 2.34 (1.43 to 3.45) | 44.75 (27.32 to 66) | 6.67 (4.12 to 9.8) | 47.82 (29.56 to 70.28) | 0.42 (0.1 to 0.75) | 0.013 |
| Arab Republic of Egypt | 14520.36 (10850.49 to 19009.41) | 304.99 (227.9 to 399.27) | 35034.56 (26056.3 to 45280.21) | 301.67 (224.36 to 389.9) | 0.15 (0.09 to 0.21) | <0.001 | 2788.74 (1775.71 to 3935.63) | 58.57 (37.3 to 82.66) | 6740.68 (4297.29 to 9472.81) | 58.04 (37 to 81.57) | 0.04 (-0.07 to 0.15) | 0.459 |
| Argentine Republic | 10468.33 (7622.24 to 13831.48) | 274.53 (199.9 to 362.73) | 15777.51 (11520.41 to 20986.59) | 260.14 (189.95 to 346.03) | -0.05 (-0.11 to 0) | 0.061 | 1782.82 (1104.13 to 2645.81) | 46.75 (28.96 to 69.39) | 2676.59 (1638.03 to 3960.25) | 44.13 (27.01 to 65.3) | 0.23 (0.16 to 0.31) | <0.001 |
| Australia | 6211.93 (4514.49 to 8265.32) | 276.25 (200.76 to 367.56) | 11782.6 (8617.5 to 15457.92) | 301.05 (220.18 to 394.96) | 0.52 (0.43 to 0.61) | <0.001 | 1050.78 (642.87 to 1552.67) | 46.73 (28.59 to 69.05) | 1954.56 (1209.61 to 2832.76) | 49.94 (30.91 to 72.38) | 0.83 (0.69 to 0.97) | <0.001 |
| Barbados | 64.13 (47.04 to 85.03) | 264.54 (194.05 to 350.73) | 153.49 (112.46 to 202.31) | 312.16 (228.72 to 411.46) | 0.83 (0.62 to 1.05) | <0.001 | 11.21 (6.88 to 16.54) | 46.23 (28.37 to 68.24) | 25.9 (16.05 to 38.1) | 52.69 (32.64 to 77.49) | 1.11 (0.81 to 1.41) | <0.001 |
| Belize | 33.76 (24.67 to 44.78) | 270.75 (197.86 to 359.12) | 118.62 (86.04 to 158.49) | 254.01 (184.25 to 339.41) | -0.1 (-0.33 to 0.13) | 0.379 | 5.94 (3.67 to 8.81) | 47.65 (29.46 to 70.64) | 20.89 (12.75 to 30.94) | 44.72 (27.31 to 66.25) | -0.09 (-0.32 to 0.14) | 0.423 |
| Bermuda | 20.84 (15.3 to 27.61) | 263.17 (193.21 to 348.6) | 39.39 (28.72 to 52.04) | 324.17 (236.4 to 428.3) | 0.79 (0.64 to 0.94) | <0.001 | 3.66 (2.28 to 5.36) | 46.16 (28.75 to 67.61) | 6.65 (4.11 to 9.79) | 54.76 (33.83 to 80.56) | 0.91 (0.72 to 1.09) | <0.001 |
| Bolivarian Republic of Venezuela | 4626.97 (3405.96 to 6078.56) | 316.73 (233.15 to 416.1) | 13460.17 (9962.11 to 17604.73) | 341.13 (252.48 to 446.17) | 0.52 (0.36 to 0.68) | <0.001 | 833.65 (522.37 to 1194.68) | 57.07 (35.76 to 81.78) | 2363.16 (1471.59 to 3401.67) | 59.89 (37.3 to 86.21) | 0.77 (0.55 to 0.98) | <0.001 |
| Bosnia and Herzegovina | 1880.58 (1377.7 to 2468.35) | 318.68 (233.46 to 418.28) | 2062.03 (1533.15 to 2708.84) | 348.87 (259.39 to 458.3) | 0.37 (0.12 to 0.61) | 0.005 | 323.91 (200.71 to 472.99) | 54.89 (34.01 to 80.15) | 352.23 (222.02 to 516.32) | 59.59 (37.56 to 87.35) | 0.2 (-0.15 to 0.55) | 0.248 |
| Brunei Darussalam | 39.7 (28.27 to 53.35) | 189.16 (134.71 to 254.17) | 152.99 (111.38 to 203.02) | 230.12 (167.53 to 305.37) | 1.02 (0.8 to 1.24) | <0.001 | 7.6 (4.59 to 11.29) | 36.2 (21.86 to 53.81) | 28 (17.43 to 41.01) | 42.12 (26.22 to 61.69) | 1.29 (0.96 to 1.62) | <0.001 |
| Burkina Faso | 1440.98 (1050.57 to 1907.38) | 239.66 (174.73 to 317.23) | 2886.31 (2047.97 to 3879.43) | 195.78 (138.91 to 263.14) | -0.66 (-0.68 to -0.64) | <0.001 | 247.87 (150.55 to 365.68) | 41.23 (25.04 to 60.82) | 505.33 (301.23 to 757.79) | 34.28 (20.43 to 51.4) | -0.79 (-0.98 to -0.59) | <0.001 |
| Canada | 15380.24 (11901.19 to 19234.36) | 422.45 (326.89 to 528.31) | 34349.54 (27475.34 to 41632.01) | 564.92 (451.87 to 684.69) | 0.91 (0.81 to 1.02) | <0.001 | 2565.56 (1709.17 to 3566.2) | 70.47 (46.95 to 97.95) | 5441.8 (3881.99 to 7243.74) | 89.5 (63.84 to 119.13) | 1.04 (0.79 to 1.29) | <0.001 |
| Central African Republic | 550.89 (402.32 to 726.34) | 294.75 (215.26 to 388.63) | 1124.01 (813.71 to 1501.72) | 254.39 (184.16 to 339.87) | -0.55 (-0.72 to -0.38) | <0.001 | 96.96 (59.68 to 142.42) | 51.88 (31.93 to 76.2) | 201.44 (124.23 to 295.82) | 45.59 (28.11 to 66.95) | -0.73 (-1.01 to -0.45) | <0.001 |
| Commonwealth of Dominica | 13.87 (10.17 to 18.41) | 242.42 (177.79 to 321.71) | 32.55 (23.81 to 43.18) | 307.96 (225.27 to 408.51) | 1.07 (0.85 to 1.3) | <0.001 | 2.47 (1.53 to 3.65) | 43.2 (26.75 to 63.75) | 5.53 (3.37 to 8.19) | 52.29 (31.9 to 77.49) | 1.17 (0.85 to 1.49) | <0.001 |
| Commonwealth of the Bahamas | 49.39 (35.64 to 66.07) | 225.66 (162.84 to 301.88) | 149.95 (109.37 to 198.56) | 262.52 (191.47 to 347.62) | 0.38 (0.2 to 0.56) | <0.001 | 8.9 (5.49 to 13.23) | 40.67 (25.07 to 60.45) | 26.24 (16.17 to 38.9) | 45.94 (28.31 to 68.1) | 0.19 (-0.06 to 0.43) | 0.128 |
| Cook Islands | 5.97 (4.43 to 7.85) | 310.41 (230.39 to 407.94) | 10.51 (7.82 to 13.63) | 373.55 (277.82 to 484.48) | 0.32 (0.13 to 0.52) | 0.002 | 1.07 (0.68 to 1.54) | 55.67 (35.14 to 79.89) | 1.8 (1.15 to 2.59) | 64.1 (40.98 to 92.05) | 0.02 (-0.26 to 0.31) | 0.872 |
| Czech Republic | 4386.01 (3201.5 to 5826.85) | 293.99 (214.59 to 390.56) | 5908.64 (4355.19 to 7791.19) | 309.97 (228.48 to 408.73) | 0.56 (0.31 to 0.8) | <0.001 | 769.73 (482.36 to 1122.57) | 51.59 (32.33 to 75.24) | 1028.05 (640.35 to 1498.68) | 53.93 (33.59 to 78.62) | 0.8 (0.47 to 1.14) | <0.001 |
| Democratic People's Republic of Korea | 5734.8 (4101.95 to 7629.32) | 239.33 (171.19 to 318.4) | 11586.9 (8384.53 to 15366.6) | 261.3 (189.08 to 346.54) | -0.43 (-0.72 to -0.14) | 0.005 | 1061.24 (651.68 to 1550.89) | 44.29 (27.2 to 64.72) | 2100.55 (1305.37 to 3072.28) | 47.37 (29.44 to 69.28) | -0.16 (-0.5 to 0.18) | 0.342 |
| Democratic Republic of Sao Tome and Principe | 19.25 (14 to 25.39) | 243.62 (177.18 to 321.43) | 36.31 (25.82 to 48.73) | 177.85 (126.45 to 238.7) | -1.22 (-1.41 to -1.03) | <0.001 | 3.2 (1.91 to 4.7) | 40.47 (24.18 to 59.53) | 6.35 (3.68 to 9.46) | 31.11 (18.04 to 46.32) | -1.21 (-1.49 to -0.92) | <0.001 |
| Democratic Republic of Timor-Leste | 179.17 (132.28 to 236.59) | 304.78 (225.02 to 402.45) | 400.3 (296.99 to 526.37) | 334.51 (248.18 to 439.86) | 0.44 (0.27 to 0.61) | <0.001 | 33.75 (21.34 to 48.75) | 57.4 (36.31 to 82.93) | 72.84 (46.68 to 105.31) | 60.87 (39.01 to 88.01) | 1.14 (0.92 to 1.35) | <0.001 |
| Democratic Republic of the Congo | 7364.36 (5401.81 to 9746.04) | 320.11 (234.8 to 423.63) | 18935.16 (13849.84 to 25084.62) | 277.87 (203.24 to 368.11) | -0.35 (-0.58 to -0.13) | 0.003 | 1280.21 (806.87 to 1876.45) | 55.65 (35.07 to 81.56) | 3354.6 (2068.03 to 4893.61) | 49.23 (30.35 to 71.81) | 0.03 (-0.4 to 0.46) | 0.884 |
| Democratic Socialist Republic of Sri Lanka | 5957.6 (4453.93 to 7868.27) | 350.69 (262.18 to 463.16) | 12220.36 (9101.9 to 15969.79) | 385.5 (287.13 to 503.78) | 0.58 (0.46 to 0.7) | <0.001 | 1083.14 (692.02 to 1566.37) | 63.76 (40.74 to 92.2) | 2153.52 (1368.33 to 3088.19) | 67.93 (43.16 to 97.42) | 1.04 (0.9 to 1.17) | <0.001 |
| Dominican Republic | 1363.38 (992.74 to 1800.46) | 253.6 (184.66 to 334.9) | 3286.01 (2367.6 to 4362.62) | 244.12 (175.89 to 324.11) | -0.1 (-0.19 to -0.02) | 0.013 | 242.82 (148.89 to 358) | 45.17 (27.7 to 66.59) | 582.48 (354.65 to 861.38) | 43.27 (26.35 to 63.99) | 0.04 (-0.08 to 0.16) | 0.48 |
| Eastern Republic of Uruguay | 1170.34 (856.93 to 1545.53) | 298.55 (218.6 to 394.25) | 1366.96 (1002.25 to 1807.78) | 281.41 (206.33 to 372.16) | -0.17 (-0.27 to -0.08) | 0.001 | 196.41 (120.91 to 290.05) | 50.1 (30.84 to 73.99) | 229.15 (139.34 to 337.97) | 47.17 (28.69 to 69.58) | 0.16 (0.03 to 0.28) | 0.014 |
| Federal Democratic Republic of Ethiopia | 10518.14 (7655.17 to 13935.53) | 312.43 (227.39 to 413.94) | 19825.18 (14358.34 to 26620.33) | 262.79 (190.32 to 352.86) | -0.5 (-0.52 to -0.47) | <0.001 | 1752.95 (1092.09 to 2527.33) | 52.07 (32.44 to 75.07) | 3369.52 (2061.71 to 4972.17) | 44.66 (27.33 to 65.91) | -0.4 (-0.44 to -0.36) | <0.001 |
| Federal Democratic Republic of Nepal | 3518.16 (2524.03 to 4716.31) | 222.72 (159.78 to 298.57) | 6593.68 (4718.15 to 8853.38) | 217.57 (155.68 to 292.13) | -0.08 (-0.1 to -0.06) | <0.001 | 642.51 (387.4 to 953.65) | 40.67 (24.52 to 60.37) | 1179.4 (710.18 to 1725.8) | 38.92 (23.43 to 56.95) | 1.04 (0.94 to 1.13) | <0.001 |
| Federal Republic of Germany | 39327.85 (28954.88 to 51895.27) | 310.24 (228.41 to 409.38) | 52204.22 (38074.04 to 69044.53) | 351.63 (256.46 to 465.07) | 0.13 (-0.11 to 0.38) | 0.28 | 6904.31 (4251.84 to 10119.15) | 54.46 (33.54 to 79.83) | 9121.86 (5580.52 to 13270.63) | 61.44 (37.59 to 89.39) | 0.15 (-0.15 to 0.44) | 0.313 |
| Federal Republic of Nigeria | 17279.49 (12381.37 to 23027.26) | 218.87 (156.83 to 291.68) | 28145.77 (19841.51 to 38120.1) | 183.18 (129.13 to 248.09) | -0.68 (-0.73 to -0.63) | <0.001 | 2818.08 (1708.73 to 4150.29) | 35.7 (21.64 to 52.57) | 4649.81 (2770.41 to 6912.93) | 30.26 (18.03 to 44.99) | -0.48 (-0.61 to -0.35) | <0.001 |
| Federal Republic of Somalia | 1106.26 (797.81 to 1470.62) | 221.99 (160.09 to 295.1) | 2377.43 (1705.91 to 3189.47) | 191.14 (137.15 to 256.43) | 0.12 (-0.25 to 0.48) | 0.512 | 204.16 (124.57 to 300.64) | 40.97 (25 to 60.33) | 448.89 (272.81 to 665.41) | 36.09 (21.93 to 53.5) | 0.8 (0.35 to 1.25) | 0.001 |
| Federated States of Micronesia | 19.97 (14.65 to 26.36) | 282.67 (207.43 to 373.13) | 40.56 (29.72 to 53.73) | 324.89 (238.05 to 430.45) | 0.93 (0.66 to 1.19) | <0.001 | 3.65 (2.32 to 5.27) | 51.72 (32.82 to 74.64) | 7.29 (4.6 to 10.63) | 58.44 (36.89 to 85.12) | 0.85 (0.45 to 1.25) | <0.001 |
| Federative Republic of Brazil | 49256.85 (36683.28 to 64368.18) | 376.13 (280.12 to 491.52) | 129636.93 (96876.09 to 169961.1) | 416.1 (310.94 to 545.53) | 0.5 (0.4 to 0.6) | <0.001 | 8695.56 (5651.68 to 12335.18) | 66.4 (43.16 to 94.19) | 23361.86 (15125.41 to 33448.78) | 74.98 (48.55 to 107.36) | 0.5 (0.36 to 0.65) | <0.001 |
| French Republic | 17734.41 (12839.61 to 23639.05) | 222.3 (160.94 to 296.31) | 24713.1 (17656.23 to 33095.59) | 238.52 (170.41 to 319.43) | 0.47 (0.34 to 0.6) | <0.001 | 3042.04 (1797.1 to 4573.36) | 38.13 (22.53 to 57.33) | 4192.31 (2527.04 to 6310.28) | 40.46 (24.39 to 60.9) | 0.41 (0.21 to 0.62) | <0.001 |
| Gabonese Republic | 228.85 (169.67 to 302.23) | 329.4 (244.21 to 435.02) | 523.39 (382.69 to 693.77) | 300.73 (219.88 to 398.63) | 0.02 (-0.22 to 0.27) | 0.838 | 39.34 (24.87 to 57.49) | 56.62 (35.8 to 82.75) | 91 (56.99 to 133.47) | 52.29 (32.75 to 76.69) | 0.01 (-0.34 to 0.36) | 0.954 |
| Georgia | 2435.68 (1796.61 to 3220.19) | 344.18 (253.88 to 455.04) | 1863.94 (1372.41 to 2438.06) | 339.16 (249.72 to 443.63) | -0.07 (-0.4 to 0.25) | 0.654 | 412.69 (258.22 to 601.19) | 58.32 (36.49 to 84.95) | 319.18 (202.42 to 464.86) | 58.08 (36.83 to 84.59) | 0.06 (-0.39 to 0.51) | 0.795 |
|  |  |  |  |  |  |  |  |  |  |  |  |  |
| Grand Duchy of Luxembourg | 125.2 (89.29 to 167.94) | 211.51 (150.84 to 283.71) | 237.52 (167.54 to 321.23) | 204.44 (144.21 to 276.49) | -0.16 (-0.3 to -0.01) | 0.038 | 21.82 (13.12 to 33.23) | 36.86 (22.16 to 56.14) | 40.12 (24.01 to 61.4) | 34.54 (20.66 to 52.85) | -0.11 (-0.29 to 0.06) | 0.198 |
| Greenland | 22.93 (16.95 to 30.32) | 306.94 (226.9 to 405.88) | 47.96 (36.47 to 62.01) | 476.25 (362.12 to 615.7) | 1.2 (0.99 to 1.42) | <0.001 | 4.17 (2.66 to 6.05) | 55.84 (35.62 to 80.94) | 7.95 (5.18 to 11.38) | 78.98 (51.48 to 112.96) | 1.51 (1.22 to 1.79) | <0.001 |
| Grenada | 18.42 (13.44 to 24.36) | 294.23 (214.65 to 389.05) | 45.14 (32.85 to 60.18) | 281.71 (205.04 to 375.6) | 0.07 (-0.1 to 0.24) | 0.419 | 3.18 (1.95 to 4.68) | 50.75 (31.1 to 74.71) | 7.76 (4.73 to 11.53) | 48.44 (29.5 to 71.96) | 0.04 (-0.17 to 0.25) | 0.704 |
| Guam | 40.22 (29.34 to 53.05) | 282.05 (205.74 to 372.05) | 84.2 (62.09 to 110.93) | 348.2 (256.78 to 458.75) | 0.75 (0.56 to 0.95) | <0.001 | 7.25 (4.51 to 10.56) | 50.82 (31.66 to 74.03) | 14.56 (9.12 to 21.05) | 60.19 (37.72 to 87.03) | 0.74 (0.41 to 1.06) | <0.001 |
| Hashemite Kingdom of Jordan | 778.89 (573.6 to 1032.19) | 310.46 (228.63 to 411.42) | 4601.33 (3375.45 to 6084.29) | 297.1 (217.94 to 392.85) | -0.6 (-0.79 to -0.4) | <0.001 | 148.46 (94.02 to 212.33) | 59.17 (37.48 to 84.63) | 881.7 (552.88 to 1276.25) | 56.93 (35.7 to 82.4) | -1.2 (-1.5 to -0.91) | <0.001 |
| Hellenic Republic | 4513.29 (3273.17 to 5965.96) | 287.84 (208.75 to 380.49) | 4778.16 (3413.28 to 6411.76) | 266.04 (190.05 to 357) | -0.33 (-0.41 to -0.24) | <0.001 | 779.56 (470 to 1162.6) | 49.72 (29.97 to 74.15) | 822.2 (499.1 to 1223.33) | 45.78 (27.79 to 68.11) | -0.29 (-0.44 to -0.15) | <0.001 |
| Hungary | 4646.09 (3407.84 to 6094.65) | 309 (226.65 to 405.34) | 5141.09 (3771.76 to 6793.88) | 304.46 (223.36 to 402.33) | 0.43 (0.24 to 0.63) | <0.001 | 811.61 (504.13 to 1176.74) | 53.98 (33.53 to 78.26) | 900.74 (568.66 to 1309.87) | 53.34 (33.68 to 77.57) | 0.55 (0.31 to 0.79) | <0.001 |
| Independent State of Papua New Guinea | 986.8 (720.72 to 1303.61) | 298.42 (217.95 to 394.23) | 2743.15 (1998 to 3660.75) | 271.54 (197.78 to 362.37) | -0.3 (-0.4 to -0.2) | <0.001 | 180.06 (111.86 to 260.13) | 54.45 (33.83 to 78.67) | 502.17 (318.56 to 734.78) | 49.71 (31.53 to 72.73) | -0.3 (-0.48 to -0.11) | 0.003 |
| Independent State of Samoa | 37.56 (27.57 to 49.71) | 307.69 (225.84 to 407.23) | 66.01 (47.67 to 87.28) | 297.72 (214.99 to 393.62) | -0.15 (-0.36 to 0.06) | 0.152 | 6.67 (4.19 to 9.65) | 54.67 (34.3 to 79.1) | 11.76 (7.29 to 17.25) | 53.06 (32.87 to 77.81) | -0.47 (-0.82 to -0.12) | 0.01 |
| Ireland | 1012.11 (713.85 to 1371.93) | 237.55 (167.55 to 322.01) | 1899.53 (1310.96 to 2569.92) | 236.57 (163.27 to 320.07) | 0.05 (0.02 to 0.08) | 0.004 | 175.72 (103.6 to 266.78) | 41.24 (24.32 to 62.62) | 326.1 (189.94 to 492.34) | 40.61 (23.66 to 61.32) | 0.35 (0.28 to 0.42) | <0.001 |
| Islamic Republic of Afghanistan | 3684.29 (2731.31 to 4835) | 389.63 (288.85 to 511.32) | 3864.54 (2659.82 to 5309.03) | 188.42 (129.69 to 258.85) | -3.52 (-4.28 to -2.76) | <0.001 | 683.71 (436.86 to 975.85) | 72.31 (46.2 to 103.2) | 803.63 (483.05 to 1190.01) | 39.18 (23.55 to 58.02) | -4.68 (-5.64 to -3.7) | <0.001 |
| Islamic Republic of Iran | 16796.81 (12517.72 to 22037.08) | 398 (296.61 to 522.17) | 41196.17 (30378.05 to 54357.46) | 332.75 (245.37 to 439.06) | -0.47 (-0.73 to -0.2) | 0.001 | 3002.18 (1936.08 to 4264.71) | 71.14 (45.88 to 101.05) | 7489.43 (4824.68 to 10737.76) | 60.49 (38.97 to 86.73) | -0.85 (-1.19 to -0.51) | <0.001 |
| Islamic Republic of Mauritania | 290.44 (208.01 to 388.72) | 212.7 (152.33 to 284.67) | 675.79 (491.12 to 905.97) | 210.41 (152.91 to 282.07) | 0.07 (-0.05 to 0.19) | 0.235 | 50.46 (30.7 to 74.45) | 36.95 (22.48 to 54.52) | 116.08 (71.3 to 172.03) | 36.14 (22.2 to 53.56) | 0.44 (0.25 to 0.63) | <0.001 |
| Islamic Republic of Pakistan | 19970.66 (14342.41 to 26649.9) | 231.68 (166.39 to 309.17) | 44359.64 (31715.94 to 59843.54) | 203.34 (145.38 to 274.32) | -0.47 (-0.54 to -0.4) | <0.001 | 3461.58 (2125.59 to 5105.09) | 40.16 (24.66 to 59.22) | 7726.6 (4729.28 to 11365.74) | 35.42 (21.68 to 52.1) | -0.17 (-0.23 to -0.1) | <0.001 |
| Jamaica | 573.14 (424.42 to 760.6) | 308.33 (228.32 to 409.17) | 1160.55 (851.77 to 1532.52) | 311.39 (228.54 to 411.19) | 0 (-0.17 to 0.17) | 0.987 | 100.02 (61.63 to 147.86) | 53.8 (33.15 to 79.54) | 198.93 (122.84 to 292.79) | 53.38 (32.96 to 78.56) | 0.01 (-0.16 to 0.19) | 0.868 |
| Japan | 62005.15 (45726.33 to 81839.27) | 292.1 (215.41 to 385.54) | 71176.88 (52544.62 to 93758.1) | 329.44 (243.2 to 433.95) | 0.48 (0.24 to 0.71) | <0.001 | 10260.73 (6475.78 to 14879.84) | 48.34 (30.51 to 70.1) | 12266.41 (7810.74 to 17662.67) | 56.77 (36.15 to 81.75) | 0.47 (0.22 to 0.72) | 0.001 |
| Kingdom of Bahrain | 117.82 (86.43 to 157.18) | 246.56 (180.87 to 328.93) | 821.91 (593.56 to 1105.51) | 257.29 (185.81 to 346.07) | 0.48 (0.13 to 0.83) | 0.008 | 23.2 (14.32 to 33.9) | 48.55 (29.97 to 70.93) | 159.97 (99.74 to 233.88) | 50.08 (31.22 to 73.21) | -0.08 (-0.52 to 0.37) | 0.732 |
| Kingdom of Belgium | 4433.32 (3250.14 to 5884.51) | 298.8 (219.06 to 396.61) | 5641.29 (4064.86 to 7497.25) | 295.04 (212.59 to 392.11) | 0.03 (-0.13 to 0.2) | 0.671 | 761.65 (463.17 to 1140.12) | 51.33 (31.22 to 76.84) | 956.67 (579.35 to 1442.11) | 50.03 (30.3 to 75.42) | 0.25 (0.04 to 0.46) | 0.019 |
| Kingdom of Bhutan | 89.78 (63.74 to 120.86) | 207.84 (147.56 to 279.79) | 173.74 (124.27 to 233.41) | 190.99 (136.62 to 256.59) | -0.23 (-0.26 to -0.19) | <0.001 | 16.16 (9.66 to 24.1) | 37.42 (22.35 to 55.79) | 31.44 (19.03 to 46.96) | 34.56 (20.92 to 51.63) | 0.39 (0.33 to 0.45) | <0.001 |
| Kingdom of Cambodia | 2067.16 (1533.86 to 2694.62) | 324.86 (241.05 to 423.46) | 5778.87 (4281.8 to 7600.9) | 331.04 (245.28 to 435.41) | -0.03 (-0.23 to 0.17) | 0.737 | 379.58 (241.69 to 540.45) | 59.65 (37.98 to 84.93) | 1051.72 (670.63 to 1522.27) | 60.25 (38.42 to 87.2) | 0.69 (0.41 to 0.98) | <0.001 |
| Kingdom of Denmark | 1856.63 (1334.92 to 2483.76) | 240.07 (172.61 to 321.16) | 2409.04 (1709.26 to 3249.79) | 253.67 (179.98 to 342.2) | 0.17 (0.06 to 0.28) | 0.004 | 329.72 (198.45 to 487.3) | 42.63 (25.66 to 63.01) | 393.11 (233.86 to 591.28) | 41.39 (24.62 to 62.26) | 0.71 (0.54 to 0.89) | <0.001 |
| Kingdom of Eswatini | 112.77 (81.66 to 151.48) | 237.46 (171.96 to 318.98) | 193.15 (138.1 to 259.16) | 210.45 (150.47 to 282.37) | -0.3 (-0.39 to -0.22) | <0.001 | 20.13 (12.1 to 29.98) | 42.39 (25.48 to 63.13) | 34.93 (20.77 to 52.03) | 38.06 (22.63 to 56.69) | 0.17 (0 to 0.34) | 0.049 |
| Kingdom of Lesotho | 243.27 (177.09 to 322.03) | 253.94 (184.86 to 336.15) | 348.66 (251.92 to 464.52) | 232.34 (167.88 to 309.54) | 0.3 (0.07 to 0.52) | 0.011 | 42.87 (26.17 to 62.87) | 44.75 (27.31 to 65.63) | 62.23 (37.87 to 92.35) | 41.47 (25.24 to 61.54) | 1.07 (0.72 to 1.42) | <0.001 |
| Kingdom of Morocco | 6705.11 (4997.29 to 8752.88) | 339.32 (252.89 to 442.95) | 16724.08 (12361.42 to 21899.18) | 336.65 (248.83 to 440.82) | 0.14 (-0.09 to 0.36) | 0.219 | 1266.28 (806.92 to 1829.03) | 64.08 (40.83 to 92.56) | 3118.83 (1966.17 to 4535.54) | 62.78 (39.58 to 91.3) | 0.54 (0.22 to 0.86) | 0.002 |
| Kingdom of Norway | 1652.55 (1193.86 to 2203.02) | 285.86 (206.51 to 381.08) | 2590.9 (1821.69 to 3473.75) | 289.36 (203.45 to 387.96) | 0.15 (0.06 to 0.24) | 0.001 | 283.7 (174.02 to 423.8) | 49.07 (30.1 to 73.31) | 427.77 (259.08 to 640.79) | 47.77 (28.93 to 71.57) | 0.54 (0.37 to 0.72) | <0.001 |
| Kingdom of Saudi Arabia | 3487.83 (2525.91 to 4644) | 243.82 (176.57 to 324.64) | 13288.43 (9494.51 to 17821.71) | 203.33 (145.28 to 272.69) | -0.55 (-0.62 to -0.48) | <0.001 | 688.97 (422.46 to 1007.31) | 48.16 (29.53 to 70.42) | 2682.25 (1656.11 to 3902.92) | 41.04 (25.34 to 59.72) | -0.74 (-0.82 to -0.66) | <0.001 |
| Kingdom of Spain | 13704.73 (10100.92 to 18036.79) | 257.7 (189.94 to 339.16) | 20436.04 (14585.51 to 27452.35) | 235.16 (167.83 to 315.89) | -0.34 (-0.46 to -0.22) | <0.001 | 2339.66 (1501.67 to 3326.03) | 44 (28.24 to 62.54) | 3445.94 (2045.36 to 5182.76) | 39.65 (23.54 to 59.64) | -0.56 (-0.75 to -0.38) | <0.001 |
| Kingdom of Sweden | 3143.48 (2231.54 to 4277.38) | 243.35 (172.75 to 331.13) | 4409.99 (3056.87 to 5975.11) | 274.14 (190.03 to 371.44) | 0.47 (0.35 to 0.59) | <0.001 | 518.96 (308.89 to 789.07) | 40.17 (23.91 to 61.09) | 713.18 (424.31 to 1065.24) | 44.33 (26.38 to 66.22) | 0.52 (0.32 to 0.72) | <0.001 |
| Kingdom of Thailand | 17558.42 (13137.56 to 22767.92) | 315.13 (235.79 to 408.63) | 44536.96 (33020.47 to 58153.01) | 355.92 (263.89 to 464.74) | 0.69 (0.49 to 0.89) | <0.001 | 3163.37 (2025.64 to 4520.14) | 56.77 (36.36 to 81.13) | 7834.53 (5002.71 to 11255.87) | 62.61 (39.98 to 89.95) | 0.61 (0.32 to 0.9) | <0.001 |
| Kingdom of Tonga | 25.15 (18.65 to 33.14) | 334.24 (247.78 to 440.39) | 32.83 (24.2 to 43.18) | 309.84 (228.43 to 407.55) | -0.57 (-0.73 to -0.41) | <0.001 | 4.45 (2.82 to 6.42) | 59.2 (37.41 to 85.37) | 5.84 (3.67 to 8.43) | 55.13 (34.67 to 79.54) | -0.58 (-0.76 to -0.4) | <0.001 |
| Kingdom of the Netherlands | 5126.86 (3728.99 to 6790.79) | 239.5 (174.2 to 317.23) | 8395.46 (5983.42 to 11153.3) | 290.54 (207.07 to 385.98) | 0.71 (0.62 to 0.81) | <0.001 | 873.14 (535.51 to 1282.07) | 40.79 (25.02 to 59.89) | 1418.3 (870.09 to 2116.13) | 49.08 (30.11 to 73.23) | 0.87 (0.77 to 0.98) | <0.001 |
| Kyrgyz Republic | 1152.73 (853.72 to 1521.55) | 321.39 (238.03 to 424.22) | 2129.31 (1570.75 to 2815.6) | 293.61 (216.59 to 388.25) | -0.27 (-0.82 to 0.28) | 0.316 | 197.74 (124.73 to 288.65) | 55.13 (34.78 to 80.48) | 373.34 (232.04 to 546.23) | 51.48 (32 to 75.32) | -0.65 (-1.36 to 0.05) | 0.069 |
| Lao People's Democratic Republic | 986.91 (725.95 to 1305.49) | 328.67 (241.76 to 434.76) | 2402.74 (1780.18 to 3147.4) | 312.24 (231.34 to 409.01) | -0.11 (-0.26 to 0.04) | 0.131 | 179.8 (113.23 to 260.44) | 59.88 (37.71 to 86.73) | 442.11 (282.09 to 631.49) | 57.45 (36.66 to 82.06) | 0.09 (-0.07 to 0.24) | 0.268 |
| Lebanese Republic | 1193.58 (883.41 to 1555.16) | 384.43 (284.53 to 500.89) | 2144.55 (1587.46 to 2832.5) | 332.65 (246.24 to 439.36) | -0.54 (-0.61 to -0.47) | <0.001 | 221.78 (141.34 to 318.79) | 71.43 (45.52 to 102.68) | 406 (260.51 to 582.92) | 62.98 (40.41 to 90.42) | -0.43 (-0.62 to -0.23) | <0.001 |
| Malaysia | 4666.82 (3455.73 to 6144.22) | 308.26 (228.26 to 405.85) | 13526.08 (10002.1 to 17807.25) | 330.9 (244.69 to 435.64) | 0.61 (0.48 to 0.74) | <0.001 | 865.17 (544.96 to 1244.29) | 57.15 (36 to 82.19) | 2430.91 (1538.71 to 3513.36) | 59.47 (37.64 to 85.95) | 0.79 (0.6 to 0.98) | <0.001 |
| Mongolia | 395.85 (285.6 to 525.78) | 271.63 (195.97 to 360.79) | 1039.62 (765.26 to 1383.52) | 260.49 (191.75 to 346.66) | -0.46 (-0.83 to -0.09) | 0.016 | 70.53 (42.98 to 103.63) | 48.4 (29.49 to 71.11) | 186.82 (115.48 to 271.96) | 46.81 (28.93 to 68.14) | -0.89 (-1.36 to -0.42) | 0.001 |
| Montenegro | 254.49 (188.82 to 334.5) | 322.93 (239.59 to 424.45) | 347.18 (258.93 to 454.67) | 341.83 (254.93 to 447.65) | 0.35 (0.14 to 0.56) | 0.002 | 44.05 (27.59 to 63.6) | 55.89 (35.01 to 80.71) | 59.54 (37.67 to 86.25) | 58.62 (37.09 to 84.92) | 0.17 (-0.15 to 0.5) | 0.283 |
| New Zealand | 1467.52 (1069.52 to 1938.85) | 339.59 (247.49 to 448.65) | 2814.82 (2072.11 to 3727.61) | 364.84 (268.57 to 483.15) | 0.36 (0.25 to 0.46) | <0.001 | 237.57 (147.55 to 348.57) | 54.97 (34.14 to 80.66) | 443.79 (276.91 to 659.65) | 57.52 (35.89 to 85.5) | 0.63 (0.49 to 0.77) | <0.001 |
| North Macedonia | 786.84 (578.9 to 1034.33) | 306.6 (225.58 to 403.04) | 1310.68 (971.34 to 1725.71) | 329.96 (244.53 to 434.44) | 0.33 (0.18 to 0.49) | <0.001 | 137.76 (86.02 to 200.17) | 53.68 (33.52 to 78) | 226.64 (143.11 to 328.58) | 57.06 (36.03 to 82.72) | 0.2 (0.02 to 0.38) | 0.028 |
| Northern Mariana Islands | 11.29 (8.03 to 15.24) | 203.91 (145.15 to 275.33) | 29.95 (22.01 to 39.5) | 325.76 (239.45 to 429.65) | 1.82 (1.46 to 2.19) | <0.001 | 2.2 (1.35 to 3.23) | 39.82 (24.44 to 58.36) | 5.26 (3.34 to 7.63) | 57.23 (36.34 to 83.02) | 2.24 (1.68 to 2.79) | <0.001 |
| Palestine | 367.02 (274.09 to 477.59) | 338.07 (252.48 to 439.92) | 1397.3 (1024.59 to 1854.38) | 301.64 (221.18 to 400.3) | -0.44 (-0.68 to -0.19) | 0.001 | 69.3 (44.33 to 99.61) | 63.83 (40.84 to 91.76) | 266.08 (167.58 to 384.6) | 57.44 (36.18 to 83.02) | -0.89 (-1.15 to -0.64) | <0.001 |
| People's Democratic Republic of Algeria | 5852.43 (4336.71 to 7632.88) | 346.35 (256.65 to 451.71) | 17664.85 (13011.88 to 23409.96) | 319.03 (235 to 422.79) | -0.17 (-0.35 to 0.01) | 0.062 | 1095.58 (695.21 to 1563.78) | 64.84 (41.14 to 92.54) | 3323.85 (2077.31 to 4788.13) | 60.03 (37.52 to 86.47) | -0.17 (-0.4 to 0.06) | 0.148 |
| People's Republic of Bangladesh | 15908.11 (11463.68 to 21347.71) | 202.47 (145.91 to 271.71) | 39491.38 (28436.23 to 52677) | 211.46 (152.27 to 282.07) | 0.29 (0.12 to 0.45) | 0.001 | 2901.86 (1776.44 to 4303.77) | 36.93 (22.61 to 54.78) | 7068.48 (4302.85 to 10455.17) | 37.85 (23.04 to 55.98) | 0.51 (0.37 to 0.65) | <0.001 |
| People's Republic of China | 449164.19 (332464.97 to 592130.67) | 349.26 (258.51 to 460.42) | 1118243 (829442.84 to 1463199.98) | 437.43 (324.46 to 572.37) | 0.66 (0.48 to 0.84) | <0.001 | 77363.64 (49790.74 to 110643.75) | 60.16 (38.72 to 86.03) | 208983.46 (136966.96 to 294533.59) | 81.75 (53.58 to 115.22) | 0.18 (-0.02 to 0.38) | 0.081 |
| Plurinational State of Bolivia | 1021.59 (732.55 to 1368.69) | 214.34 (153.7 to 287.16) | 2886.1 (2070.66 to 3831.43) | 221.03 (158.58 to 293.43) | 0.28 (0.21 to 0.35) | <0.001 | 174.79 (104.64 to 259.65) | 36.67 (21.96 to 54.48) | 489.05 (299.03 to 722.75) | 37.45 (22.9 to 55.35) | 0.39 (0.31 to 0.46) | <0.001 |
| Portuguese Republic | 3676.65 (2646.62 to 4936.25) | 267.47 (192.54 to 359.11) | 4925.74 (3521.89 to 6613.48) | 267.74 (191.43 to 359.47) | 0.04 (-0.04 to 0.12) | 0.261 | 636.03 (387.55 to 953.38) | 46.27 (28.19 to 69.36) | 849.73 (509.12 to 1270.48) | 46.19 (27.67 to 69.06) | 0.07 (-0.04 to 0.18) | 0.196 |
| Principality of Andorra | 18.59 (13.32 to 25.22) | 236.29 (169.28 to 320.48) | 46.94 (33.11 to 63.14) | 248.64 (175.38 to 334.44) | 0.15 (0.02 to 0.28) | 0.028 | 3.27 (1.95 to 4.96) | 41.6 (24.79 to 63.05) | 8.03 (4.79 to 12.01) | 42.53 (25.38 to 63.6) | 0.01 (-0.18 to 0.21) | 0.901 |
| Principality of Monaco | 14.32 (10.39 to 18.97) | 280.14 (203.21 to 371.04) | 20.39 (14.61 to 27.4) | 298.51 (213.91 to 401.05) | 0.1 (0.01 to 0.19) | 0.027 | 2.49 (1.54 to 3.69) | 48.62 (30.03 to 72.14) | 3.43 (2.06 to 5.12) | 50.22 (30.21 to 75) | 0.22 (0.11 to 0.33) | <0.001 |
| Puerto Rico | 1085.7 (790.97 to 1444) | 268.3 (195.47 to 356.85) | 1635.73 (1205.23 to 2164.71) | 319.41 (235.35 to 422.7) | 0.61 (0.59 to 0.63) | <0.001 | 191.09 (117.63 to 279.24) | 47.22 (29.07 to 69.01) | 276.86 (172.68 to 408.4) | 54.06 (33.72 to 79.75) | 0.85 (0.78 to 0.92) | <0.001 |
| Republic of Albania | 956.14 (697.46 to 1275.66) | 291.16 (212.39 to 388.46) | 1619.9 (1206.12 to 2116.43) | 393.16 (292.73 to 513.67) | 0.98 (0.78 to 1.17) | <0.001 | 166.31 (103.12 to 245.36) | 50.64 (31.4 to 74.72) | 269.18 (170.01 to 389.96) | 65.33 (41.26 to 94.65) | 0.88 (0.65 to 1.11) | <0.001 |
| Republic of Angola | 2111.13 (1552.79 to 2805.95) | 292.25 (214.96 to 388.44) | 5727.9 (4186.51 to 7612.35) | 268.63 (196.34 to 357.01) | -0.22 (-0.33 to -0.11) | <0.001 | 373.55 (235.53 to 548.01) | 51.71 (32.6 to 75.86) | 1022.68 (628.28 to 1506.61) | 47.96 (29.47 to 70.66) | 0.22 (-0.01 to 0.45) | 0.059 |
| Republic of Armenia | 1301.59 (955.01 to 1703.89) | 360.54 (264.54 to 471.98) | 1565.54 (1160.37 to 2053.59) | 364.63 (270.26 to 478.3) | 0.29 (-0.28 to 0.87) | 0.307 | 218.96 (138.45 to 319.93) | 60.65 (38.35 to 88.62) | 264.68 (167.33 to 387.78) | 61.65 (38.97 to 90.32) | 0.4 (-0.37 to 1.18) | 0.297 |
| Republic of Austria | 2812.81 (2021.74 to 3781.3) | 253.39 (182.13 to 340.64) | 4245.99 (3028.67 to 5674.76) | 271.1 (193.38 to 362.33) | 0.03 (-0.12 to 0.18) | 0.667 | 491.35 (301.2 to 736.42) | 44.26 (27.13 to 66.34) | 724.97 (439.56 to 1085.91) | 46.29 (28.07 to 69.33) | 0.17 (0.01 to 0.33) | 0.042 |
| Republic of Azerbaijan | 2205.05 (1626.09 to 2911.18) | 336.33 (248.02 to 444.03) | 5000.7 (3682.13 to 6533.41) | 324.88 (239.22 to 424.46) | -0.37 (-1.04 to 0.3) | 0.262 | 373.38 (233.72 to 548.33) | 56.95 (35.65 to 83.64) | 855.61 (534.21 to 1245.25) | 55.59 (34.71 to 80.9) | -0.77 (-1.64 to 0.1) | 0.081 |
| Republic of Belarus | 4480.2 (3313.28 to 5854.95) | 327.69 (242.34 to 428.24) | 5204.42 (3856.82 to 6822.5) | 338.84 (251.1 to 444.19) | 0.22 (-0.2 to 0.63) | 0.294 | 775.63 (491.71 to 1129.36) | 56.73 (35.96 to 82.6) | 895.44 (563.39 to 1301.18) | 58.3 (36.68 to 84.72) | 0.23 (-0.27 to 0.74) | 0.355 |
| Republic of Benin | 600.12 (430.51 to 800.01) | 227.46 (163.18 to 303.22) | 1740.44 (1243.78 to 2319.08) | 200.24 (143.1 to 266.82) | -0.35 (-0.41 to -0.28) | <0.001 | 104.23 (62.32 to 153.59) | 39.51 (23.62 to 58.22) | 303.63 (184.06 to 446.03) | 34.93 (21.18 to 51.32) | -0.4 (-0.55 to -0.25) | <0.001 |
| Republic of Botswana | 212.62 (155.25 to 282.48) | 262.53 (191.69 to 348.79) | 574.67 (414.16 to 767.89) | 224.97 (162.13 to 300.61) | -0.33 (-0.41 to -0.24) | <0.001 | 37.25 (23 to 54.67) | 45.99 (28.4 to 67.51) | 103.43 (63.32 to 151.89) | 40.49 (24.79 to 59.46) | -0.37 (-0.43 to -0.32) | <0.001 |
| Republic of Bulgaria | 4655.06 (3447.72 to 6106.35) | 336.22 (249.02 to 441.05) | 3906.41 (2875.7 to 5126.96) | 320.1 (235.64 to 420.11) | 0.02 (-0.08 to 0.12) | 0.685 | 807.22 (503.8 to 1167.03) | 58.3 (36.39 to 84.29) | 680.23 (425.28 to 983.33) | 55.74 (34.85 to 80.58) | -0.01 (-0.16 to 0.14) | 0.902 |
| Republic of Burundi | 822.1 (600.48 to 1090.16) | 262.97 (192.08 to 348.72) | 2327.54 (1699.95 to 3108.55) | 247.61 (180.85 to 330.7) | 0.13 (-0.09 to 0.35) | 0.231 | 145.88 (89.81 to 213.03) | 46.66 (28.73 to 68.14) | 409.87 (252.88 to 604.98) | 43.6 (26.9 to 64.36) | 0.62 (0.28 to 0.96) | 0.001 |
| Republic of Cabo Verde | 51.77 (38.09 to 68.03) | 287.79 (211.74 to 378.14) | 138.2 (98.16 to 185.25) | 207.4 (147.31 to 278.02) | -1.3 (-1.92 to -0.67) | <0.001 | 8.41 (5.24 to 12.26) | 46.77 (29.15 to 68.14) | 23.6 (14.02 to 35.17) | 35.41 (21.03 to 52.78) | -1.56 (-2.38 to -0.74) | 0.001 |
| Republic of Cameroon | 1536.4 (1100.39 to 2056.28) | 216.74 (155.23 to 290.08) | 4078.28 (2901.08 to 5515.52) | 179.41 (127.62 to 242.64) | -0.54 (-0.61 to -0.48) | <0.001 | 265.01 (160.54 to 394.67) | 37.39 (22.65 to 55.68) | 720.7 (434.51 to 1076.19) | 31.7 (19.11 to 47.34) | -0.54 (-0.72 to -0.36) | <0.001 |
| Republic of Chad | 787.76 (565.3 to 1048.18) | 223.08 (160.09 to 296.83) | 2068.74 (1476.4 to 2753.47) | 200.4 (143.02 to 266.74) | -0.31 (-0.38 to -0.25) | <0.001 | 136.99 (83.09 to 202.76) | 38.79 (23.53 to 57.42) | 359.38 (214.87 to 527.88) | 34.81 (20.81 to 51.14) | 0.28 (0.19 to 0.37) | <0.001 |
| Republic of Chile | 3429.57 (2497.71 to 4569.01) | 260.39 (189.64 to 346.9) | 8158.64 (5944.33 to 10809.42) | 294.68 (214.7 to 390.42) | 0.4 (0.23 to 0.57) | <0.001 | 579.22 (354.38 to 852.26) | 43.98 (26.91 to 64.71) | 1365.2 (839.62 to 2021.77) | 49.31 (30.33 to 73.02) | 0.56 (0.32 to 0.8) | <0.001 |
| Republic of Colombia | 7299.14 (5314.44 to 9713.68) | 271.6 (197.75 to 361.45) | 19849.23 (14591.26 to 26266.59) | 313.1 (230.16 to 414.33) | 0.56 (0.42 to 0.7) | <0.001 | 1316.52 (814.95 to 1932.94) | 48.99 (30.32 to 71.93) | 3474.49 (2154.14 to 5098.84) | 54.81 (33.98 to 80.43) | 0.71 (0.52 to 0.91) | <0.001 |
| Republic of Costa Rica | 673.8 (492.51 to 894.37) | 278.11 (203.28 to 369.15) | 1994.83 (1483.08 to 2635.01) | 315.12 (234.28 to 416.25) | 0.58 (0.37 to 0.78) | <0.001 | 121.06 (75.09 to 178.05) | 49.97 (30.99 to 73.49) | 348.62 (217.35 to 513.88) | 55.07 (34.33 to 81.18) | 0.73 (0.44 to 1.01) | <0.001 |
| Republic of Croatia | 2509.19 (1848.94 to 3291.78) | 332.43 (244.96 to 436.12) | 2593.78 (1922.9 to 3385.98) | 356.84 (264.54 to 465.82) | 0.35 (0.16 to 0.54) | 0.001 | 433.73 (270.6 to 629.36) | 57.46 (35.85 to 83.38) | 442.68 (280.82 to 638.91) | 60.9 (38.63 to 87.9) | 0.27 (0.04 to 0.51) | 0.026 |
| Republic of Cuba | 3177.24 (2306.4 to 4212.47) | 252.21 (183.09 to 334.39) | 5928.05 (4351.33 to 7837) | 292.26 (214.53 to 386.38) | 0 (-0.2 to 0.21) | 0.974 | 553.51 (341.01 to 810.46) | 43.94 (27.07 to 64.34) | 1005.63 (619.46 to 1471.12) | 49.58 (30.54 to 72.53) | 0.14 (-0.05 to 0.34) | 0.142 |
| Republic of Cyprus | 226.39 (160.04 to 303.19) | 238.62 (168.68 to 319.57) | 538.21 (382.39 to 713.82) | 260.04 (184.76 to 344.89) | 0.45 (0.39 to 0.52) | <0.001 | 39.82 (23.57 to 59.57) | 41.97 (24.85 to 62.78) | 93.13 (55.88 to 139.76) | 45 (27 to 67.53) | 0.1 (0 to 0.2) | 0.041 |
| Population of Côte d ivoire | 1586.08 (1131.62 to 2134.91) | 193.25 (137.88 to 260.12) | 4183.15 (2934.5 to 5668.6) | 176.47 (123.79 to 239.14) | -0.27 (-0.32 to -0.22) | <0.001 | 281.3 (167.87 to 418.45) | 34.27 (20.45 to 50.98) | 748.42 (439.85 to 1108.03) | 31.57 (18.56 to 46.74) | -0.21 (-0.4 to -0.03) | 0.026 |
| Republic of Djibouti | 72.6 (52.61 to 96.21) | 241.19 (174.77 to 319.6) | 354.44 (257.44 to 470.1) | 245.44 (178.27 to 325.53) | 0.15 (0.12 to 0.19) | <0.001 | 13.17 (8.06 to 19.27) | 43.76 (26.77 to 64.03) | 63.42 (39.12 to 93.42) | 43.92 (27.09 to 64.69) | 0.63 (0.56 to 0.69) | <0.001 |
| Republic of Ecuador | 1704.7 (1231.14 to 2280.01) | 218.73 (157.97 to 292.55) | 4747.55 (3426.39 to 6306.74) | 228.43 (164.86 to 303.45) | 0.26 (0.21 to 0.31) | <0.001 | 290.58 (174.77 to 434.56) | 37.28 (22.43 to 55.76) | 795.41 (481.43 to 1175.84) | 38.27 (23.16 to 56.58) | 0.37 (0.28 to 0.46) | <0.001 |
| Republic of El Salvador | 1059.13 (769.15 to 1401.4) | 275.39 (200 to 364.39) | 1786.36 (1306.39 to 2351.61) | 287.55 (210.29 to 378.53) | 0.13 (0.11 to 0.14) | <0.001 | 190.57 (117.21 to 279.29) | 49.55 (30.48 to 72.62) | 320.32 (198.38 to 470.07) | 51.56 (31.93 to 75.67) | 0.06 (0.02 to 0.1) | 0.009 |
| Republic of Equatorial Guinea | 89.78 (65.98 to 119.15) | 319.75 (235 to 424.34) | 218.4 (157.49 to 293.64) | 240.55 (173.46 to 323.42) | -0.89 (-0.94 to -0.84) | <0.001 | 15.58 (9.74 to 22.65) | 55.49 (34.7 to 80.67) | 40.03 (24.47 to 59.16) | 44.09 (26.95 to 65.16) | -1.1 (-1.18 to -1.01) | <0.001 |
| Republic of Estonia | 647.29 (471.95 to 861.09) | 302.19 (220.33 to 402.01) | 688.17 (505.31 to 907.28) | 329.48 (241.93 to 434.38) | 0.34 (0.24 to 0.43) | <0.001 | 112.74 (70.14 to 165.87) | 52.63 (32.74 to 77.44) | 117.97 (73.9 to 171.39) | 56.48 (35.38 to 82.06) | 0.36 (0.22 to 0.5) | <0.001 |
| Republic of Fiji | 168.7 (123.9 to 223.19) | 256.37 (188.29 to 339.17) | 367.98 (269.48 to 487.55) | 303.23 (222.06 to 401.76) | 0.63 (0.54 to 0.72) | <0.001 | 31.42 (19.58 to 45.55) | 47.75 (29.76 to 69.23) | 65.77 (40.95 to 95.84) | 54.19 (33.74 to 78.98) | 0.58 (0.45 to 0.72) | <0.001 |
| Republic of Finland | 1919.47 (1354.09 to 2569.41) | 249.76 (176.2 to 334.34) | 2468.94 (1745.91 to 3313.01) | 283.02 (200.14 to 379.78) | 0.7 (0.57 to 0.83) | <0.001 | 329.54 (189.3 to 503.08) | 42.88 (24.63 to 65.46) | 412.55 (243.07 to 623.5) | 47.29 (27.86 to 71.47) | 1.15 (0.95 to 1.35) | <0.001 |
| Republic of Ghana | 2060.11 (1470.97 to 2770.16) | 199.2 (142.24 to 267.86) | 4770.27 (3371.65 to 6437.23) | 175.67 (124.16 to 237.05) | -0.33 (-0.39 to -0.26) | <0.001 | 359.14 (214.76 to 534.68) | 34.73 (20.77 to 51.7) | 842.67 (504.28 to 1263.83) | 31.03 (18.57 to 46.54) | 0.2 (0.06 to 0.33) | 0.006 |
| Republic of Guatemala | 1570.37 (1149.02 to 2076.63) | 284.13 (207.9 to 375.73) | 4025.24 (2965.28 to 5320.67) | 279.44 (205.86 to 369.37) | 0.12 (0.05 to 0.18) | 0.001 | 283.63 (175.62 to 416.58) | 51.32 (31.78 to 75.37) | 726.22 (449.2 to 1066.33) | 50.42 (31.18 to 74.03) | 0.12 (0.05 to 0.2) | 0.001 |
| Republic of Guinea | 1058.45 (764.94 to 1403.6) | 237.96 (171.97 to 315.56) | 1794.61 (1302.51 to 2394.74) | 210.16 (152.53 to 280.43) | -0.28 (-0.33 to -0.23) | <0.001 | 181.06 (110.17 to 268.05) | 40.71 (24.77 to 60.26) | 309.67 (186.13 to 457.03) | 36.26 (21.8 to 53.52) | 0.17 (0.09 to 0.25) | <0.001 |
| Republic of Guinea-Bissau | 119.54 (85.69 to 159.62) | 207.03 (148.4 to 276.45) | 227.21 (160.9 to 306.12) | 177.69 (125.83 to 239.4) | -0.36 (-0.41 to -0.3) | <0.001 | 20.73 (12.35 to 30.81) | 35.91 (21.39 to 53.35) | 40.29 (23.72 to 59.99) | 31.51 (18.55 to 46.91) | -0.19 (-0.28 to -0.1) | <0.001 |
| Republic of Guyana | 129.9 (94.2 to 174.03) | 225.98 (163.88 to 302.77) | 241.47 (175.69 to 323.31) | 260.41 (189.48 to 348.68) | 0.68 (0.46 to 0.89) | <0.001 | 23.6 (14.4 to 34.88) | 41.05 (25.06 to 60.68) | 42.61 (26.06 to 63.16) | 45.96 (28.1 to 68.12) | 1.05 (0.81 to 1.29) | <0.001 |
| Republic of Haiti | 1320.56 (952.55 to 1755.28) | 265.69 (191.65 to 353.15) | 2733.22 (1975.22 to 3624.56) | 229.49 (165.85 to 304.33) | -0.35 (-0.44 to -0.26) | <0.001 | 235.02 (143.73 to 347.89) | 47.28 (28.92 to 69.99) | 491.1 (302.18 to 734.23) | 41.23 (25.37 to 61.65) | -0.28 (-0.42 to -0.13) | <0.001 |
| Republic of Honduras | 845.29 (619.39 to 1119.78) | 278.58 (204.13 to 369.04) | 2483.86 (1818.48 to 3281.55) | 275.22 (201.49 to 363.6) | 0.1 (0.04 to 0.15) | 0.002 | 153.07 (95.03 to 223.53) | 50.44 (31.32 to 73.67) | 450.5 (279.45 to 655.54) | 49.92 (30.96 to 72.63) | 0.53 (0.46 to 0.59) | <0.001 |
| Republic of Iceland | 83.29 (60.29 to 110.92) | 277.42 (200.8 to 369.44) | 156.02 (111.01 to 209.89) | 285.99 (203.48 to 384.74) | 0.42 (0.21 to 0.63) | <0.001 | 14.71 (8.95 to 21.99) | 48.99 (29.81 to 73.25) | 27.01 (16.35 to 40.09) | 49.51 (29.97 to 73.49) | 0.7 (0.42 to 0.98) | <0.001 |
| Republic of India | 166146.89 (118102.11 to 222710.64) | 203.9 (144.94 to 273.32) | 361685.73 (256387.35 to 483647.44) | 209.94 (148.82 to 280.73) | -0.05 (-0.17 to 0.07) | 0.411 | 29317.97 (17855.58 to 43194.13) | 35.98 (21.91 to 53.01) | 62999.64 (38315.45 to 92910.64) | 36.57 (22.24 to 53.93) | 0.47 (0.35 to 0.59) | <0.001 |
| Republic of Indonesia | 55436.21 (40915.92 to 73263.68) | 346.76 (255.93 to 458.27) | 139681.24 (103231.11 to 185042.79) | 347.64 (256.92 to 460.54) | 0 (-0.15 to 0.15) | 0.975 | 9765.02 (6258.14 to 13960.6) | 61.08 (39.15 to 87.33) | 24446.37 (15816.69 to 34913.17) | 60.84 (39.37 to 86.89) | 0.39 (0.21 to 0.57) | <0.001 |
| Republic of Iraq | 3518.85 (2586.46 to 4660.16) | 289.72 (212.96 to 383.7) | 12207.5 (8886.65 to 16244.58) | 268.03 (195.11 to 356.66) | -0.3 (-0.35 to -0.25) | <0.001 | 680.83 (428.91 to 980.68) | 56.06 (35.31 to 80.74) | 2366.74 (1471.24 to 3444.48) | 51.96 (32.3 to 75.63) | -0.52 (-0.61 to -0.44) | <0.001 |
| Republic of Italy | 29053.29 (21388 to 38099.1) | 338.28 (249.03 to 443.61) | 33893.63 (24397.94 to 45106.35) | 308.11 (221.79 to 410.04) | -0.44 (-0.53 to -0.35) | <0.001 | 4953.97 (3077.96 to 7161.15) | 57.68 (35.84 to 83.38) | 5513.98 (3357.12 to 8276.94) | 50.13 (30.52 to 75.24) | -0.37 (-0.43 to -0.3) | <0.001 |
| Republic of Kazakhstan | 4753.48 (3494.43 to 6262.06) | 294.08 (216.19 to 387.41) | 7074.58 (5185.09 to 9407.44) | 291.05 (213.31 to 387.02) | -0.06 (-0.39 to 0.28) | 0.728 | 828.73 (522.17 to 1210.39) | 51.27 (32.3 to 74.88) | 1238.94 (776.25 to 1819.8) | 50.97 (31.93 to 74.87) | -0.4 (-0.84 to 0.04) | 0.072 |
| Republic of Kenya | 3692.98 (2692.49 to 4925.77) | 285.89 (208.44 to 381.32) | 10498.51 (7594.53 to 14099.24) | 257.28 (186.11 to 345.51) | -0.27 (-0.3 to -0.23) | <0.001 | 624.21 (392.71 to 901.31) | 48.32 (30.4 to 69.77) | 1785.56 (1112.47 to 2605.92) | 43.76 (27.26 to 63.86) | 0.47 (0.4 to 0.54) | <0.001 |
| Republic of Kiribati | 14.88 (10.78 to 19.81) | 260.7 (188.93 to 347.21) | 33.86 (24.82 to 44.85) | 289.58 (212.22 to 383.53) | 0.39 (0.18 to 0.61) | 0.001 | 2.75 (1.72 to 3.99) | 48.18 (30.1 to 70) | 6.14 (3.87 to 8.85) | 52.54 (33.12 to 75.64) | 0.41 (0.07 to 0.75) | 0.019 |
| Republic of Korea | 12410 (9046.31 to 16280.53) | 261.73 (190.79 to 343.37) | 41338.73 (30785.41 to 53950.76) | 389 (289.69 to 507.68) | 1.04 (0.83 to 1.25) | <0.001 | 2261.3 (1407.07 to 3248.18) | 47.69 (29.68 to 68.51) | 7463.67 (4722.62 to 10694.04) | 70.23 (44.44 to 100.63) | 0.94 (0.66 to 1.21) | <0.001 |
| Republic of Latvia | 1120.86 (819.15 to 1469.94) | 303.52 (221.82 to 398.05) | 1033.67 (764.59 to 1360.95) | 342.41 (253.28 to 450.83) | 0.26 (0.12 to 0.4) | 0.001 | 196.13 (123.1 to 283.57) | 53.11 (33.33 to 76.79) | 178.13 (112.41 to 258.81) | 59.01 (37.24 to 85.73) | 0.17 (0.01 to 0.33) | 0.033 |
| Republic of Liberia | 355.9 (257.65 to 474.98) | 216.7 (156.88 to 289.2) | 769.16 (541.07 to 1048.47) | 162.42 (114.26 to 221.4) | -0.96 (-1.04 to -0.87) | <0.001 | 61.42 (37.57 to 90.82) | 37.4 (22.87 to 55.3) | 140.12 (82.18 to 209.76) | 29.59 (17.35 to 44.29) | -0.95 (-1.21 to -0.68) | <0.001 |
| Republic of Lithuania | 1445.65 (1065.74 to 1900.91) | 302.94 (223.33 to 398.34) | 1559.31 (1159.53 to 2036.43) | 348.89 (259.44 to 455.65) | 0.25 (0.01 to 0.48) | 0.04 | 252.67 (159.47 to 368.38) | 52.95 (33.42 to 77.19) | 266.67 (169.23 to 385.58) | 59.67 (37.86 to 86.27) | 0.11 (-0.16 to 0.37) | 0.417 |
| Republic of Madagascar | 2103.19 (1549.3 to 2779.01) | 277.81 (204.65 to 367.08) | 5144.82 (3723.15 to 6873.17) | 238.45 (172.56 to 318.55) | -0.32 (-0.54 to -0.1) | 0.006 | 366.95 (228.82 to 530) | 48.47 (30.23 to 70.01) | 912.07 (557.86 to 1341.78) | 42.27 (25.86 to 62.19) | -0.24 (-0.55 to 0.07) | 0.13 |
| Republic of Malawi | 1552.04 (1136.86 to 2051.51) | 258.75 (189.53 to 342.02) | 2854.11 (2052.78 to 3815.47) | 223.7 (160.89 to 299.05) | -0.43 (-0.52 to -0.33) | <0.001 | 276.37 (168.99 to 404.66) | 46.07 (28.17 to 67.46) | 518.01 (318.45 to 764.65) | 40.6 (24.96 to 59.93) | 0.1 (-0.04 to 0.24) | 0.145 |
| Republic of Maldives | 64.05 (47.81 to 83.73) | 388.15 (289.73 to 507.4) | 221.78 (162.94 to 295.12) | 265.32 (194.93 to 353.06) | -1.46 (-1.62 to -1.3) | <0.001 | 11.34 (7.27 to 16.11) | 68.73 (44.03 to 97.64) | 42.01 (26.44 to 61.07) | 50.26 (31.63 to 73.07) | -2 (-2.2 to -1.81) | <0.001 |
| Republic of Mali | 1450.2 (1054.3 to 1917.01) | 232.42 (168.97 to 307.24) | 3169.12 (2276.19 to 4232.13) | 210.33 (151.06 to 280.87) | -0.38 (-0.42 to -0.35) | <0.001 | 248.48 (152.22 to 367.5) | 39.82 (24.4 to 58.9) | 548.56 (329.8 to 804.42) | 36.41 (21.89 to 53.39) | -0.24 (-0.35 to -0.12) | <0.001 |
| Republic of Malta | 114.29 (80.05 to 154.74) | 224.87 (157.51 to 304.47) | 194.4 (135.61 to 262.78) | 266.12 (185.64 to 359.73) | 0.95 (0.76 to 1.14) | <0.001 | 20 (11.75 to 30.47) | 39.35 (23.11 to 59.95) | 32.98 (19.55 to 50.04) | 45.14 (26.77 to 68.51) | 1.38 (1.12 to 1.64) | <0.001 |
| Republic of Mauritius | 334.79 (247.73 to 439.39) | 323.49 (239.37 to 424.57) | 865.4 (648.48 to 1133.05) | 391.59 (293.43 to 512.69) | 1.16 (0.83 to 1.5) | <0.001 | 60.95 (38.75 to 87.17) | 58.89 (37.44 to 84.23) | 152.31 (97.67 to 219.67) | 68.92 (44.19 to 99.4) | 1.22 (0.75 to 1.7) | <0.001 |
| Republic of Moldova | 1525.64 (1117.41 to 2029.83) | 303.95 (222.62 to 404.4) | 1896.85 (1399.56 to 2486.95) | 316.03 (233.18 to 414.35) | 0.44 (0.16 to 0.73) | 0.004 | 265.07 (164.77 to 392.06) | 52.81 (32.83 to 78.11) | 328.38 (204.24 to 479.51) | 54.71 (34.03 to 79.89) | 0.44 (0.07 to 0.82) | 0.022 |
| Republic of Mozambique | 2568.66 (1867.8 to 3413.2) | 269.99 (196.32 to 358.76) | 4297.7 (3106.19 to 5763.82) | 228.64 (165.25 to 306.64) | -0.52 (-0.56 to -0.49) | <0.001 | 454.64 (279.65 to 661.43) | 47.79 (29.39 to 69.52) | 771.06 (472.73 to 1133.35) | 41.02 (25.15 to 60.29) | 0.3 (0.23 to 0.37) | <0.001 |
| Republic of Namibia | 249.66 (182.63 to 330.34) | 264.7 (193.63 to 350.25) | 501.33 (363.05 to 669.6) | 233.09 (168.8 to 311.32) | -0.43 (-0.47 to -0.4) | <0.001 | 43.8 (27.28 to 64.33) | 46.44 (28.92 to 68.21) | 89.1 (54.83 to 132.29) | 41.43 (25.49 to 61.51) | 0.19 (0.14 to 0.24) | <0.001 |
| Republic of Nauru | 2.37 (1.71 to 3.12) | 261.11 (188.49 to 344.03) | 2.41 (1.73 to 3.21) | 249.42 (179.15 to 331.41) | -0.1 (-0.17 to -0.03) | 0.008 | 0.44 (0.28 to 0.64) | 48.74 (30.35 to 70.55) | 0.45 (0.28 to 0.65) | 46.56 (28.55 to 67.45) | -0.17 (-0.28 to -0.06) | 0.005 |
| Republic of Nicaragua | 624.68 (456.4 to 827.23) | 272.06 (198.77 to 360.27) | 1868.37 (1364.35 to 2470.8) | 274.97 (200.79 to 363.63) | 0.14 (0.06 to 0.22) | 0.001 | 113.23 (69.69 to 165.07) | 49.31 (30.35 to 71.89) | 337.22 (209.4 to 492.22) | 49.63 (30.82 to 72.44) | 0.02 (-0.08 to 0.13) | 0.651 |
| Republic of Niue | 0.73 (0.53 to 0.97) | 304.45 (221.04 to 401.9) | 0.94 (0.69 to 1.23) | 344.58 (254.04 to 452.72) | 0.31 (0.2 to 0.42) | <0.001 | 0.13 (0.08 to 0.19) | 54.14 (33.54 to 78.28) | 0.16 (0.1 to 0.23) | 59.78 (37.9 to 86.26) | -0.01 (-0.16 to 0.14) | 0.925 |
| Republic of Palau | 3.88 (2.78 to 5.15) | 264.86 (189.95 to 351.55) | 11.55 (8.38 to 15.37) | 291.36 (211.28 to 387.76) | 0.78 (0.44 to 1.11) | <0.001 | 0.71 (0.44 to 1.03) | 48.29 (29.74 to 70.56) | 2.07 (1.3 to 3) | 52.11 (32.68 to 75.79) | 0.57 (0.07 to 1.07) | 0.028 |
| Republic of Panama | 583.1 (425.7 to 777.32) | 271.47 (198.19 to 361.89) | 1619.85 (1185.74 to 2138.7) | 295.79 (216.52 to 390.53) | 0.25 (0.19 to 0.31) | <0.001 | 104.64 (64.85 to 154.02) | 48.72 (30.19 to 71.7) | 285.36 (177.78 to 417.51) | 52.11 (32.46 to 76.24) | 0.26 (0.2 to 0.31) | <0.001 |
| Republic of Paraguay | 1073.28 (797.52 to 1421.2) | 341.43 (253.71 to 452.11) | 2823.32 (2099.9 to 3691.97) | 347.83 (258.71 to 454.85) | 0.28 (0.16 to 0.4) | <0.001 | 197.11 (125.13 to 286.01) | 62.7 (39.81 to 90.98) | 510.98 (325.64 to 734.32) | 62.95 (40.12 to 90.47) | 0.47 (0.33 to 0.61) | <0.001 |
| Republic of Peru | 3885.76 (2790 to 5163.99) | 221.6 (159.11 to 294.5) | 9821.33 (6976.22 to 13148.06) | 222.91 (158.34 to 298.42) | 0.03 (-0.02 to 0.07) | 0.197 | 649.28 (388.88 to 957.33) | 37.03 (22.18 to 54.6) | 1623.95 (970.34 to 2401.81) | 36.86 (22.02 to 54.51) | 0.04 (-0.04 to 0.12) | 0.338 |
| Republic of Poland | 16767.46 (12425.56 to 22083.03) | 340.85 (252.59 to 448.9) | 22521.99 (16776.02 to 29563.92) | 348.97 (259.94 to 458.09) | 0.71 (0.42 to 0.99) | <0.001 | 2820.17 (1806.97 to 4055.12) | 57.33 (36.73 to 82.43) | 3747.25 (2377.57 to 5403.4) | 58.06 (36.84 to 83.72) | 0.96 (0.54 to 1.38) | <0.001 |
| Republic of Rwanda | 1037.34 (762.83 to 1375.72) | 271.07 (199.34 to 359.5) | 2479.58 (1813.35 to 3307.93) | 249.42 (182.4 to 332.74) | 0.35 (0.05 to 0.65) | 0.024 | 181.22 (113.13 to 264.99) | 47.36 (29.56 to 69.25) | 438.68 (268.63 to 641.9) | 44.13 (27.02 to 64.57) | 0.72 (0.32 to 1.13) | 0.001 |
| Republic of San Marino | 10 (7.2 to 13.25) | 288.64 (207.78 to 382.44) | 16.02 (11.37 to 21.49) | 278.22 (197.47 to 373.33) | -0.28 (-0.36 to -0.2) | <0.001 | 1.72 (1.04 to 2.57) | 49.6 (30.02 to 74.1) | 2.69 (1.6 to 4.12) | 46.74 (27.87 to 71.54) | -0.02 (-0.13 to 0.09) | 0.731 |
| Republic of Senegal | 1059.87 (764.52 to 1418.1) | 222.62 (160.58 to 297.86) | 2423.72 (1742.69 to 3237.11) | 204.31 (146.9 to 272.88) | -0.2 (-0.26 to -0.15) | <0.001 | 182.92 (111.29 to 270.78) | 38.42 (23.38 to 56.88) | 417.22 (249.47 to 623.88) | 35.17 (21.03 to 52.59) | 0.6 (0.41 to 0.79) | <0.001 |
| Republic of Serbia | 4790.7 (3552.21 to 6304.24) | 333.44 (247.24 to 438.79) | 4974.85 (3670.31 to 6501.1) | 338.48 (249.72 to 442.33) | 0.29 (0.17 to 0.42) | <0.001 | 823.82 (519.81 to 1203.68) | 57.34 (36.18 to 83.78) | 856.39 (538.15 to 1238.68) | 58.27 (36.62 to 84.28) | 0.24 (0.08 to 0.39) | 0.003 |
| Republic of Seychelles | 22.04 (16.37 to 29.19) | 345.27 (256.46 to 457.26) | 61.42 (45.36 to 80.36) | 334.33 (246.91 to 437.38) | -0.03 (-0.38 to 0.32) | 0.86 | 3.96 (2.51 to 5.75) | 62.11 (39.29 to 90) | 11.11 (6.98 to 15.87) | 60.5 (37.97 to 86.38) | -0.52 (-0.99 to -0.05) | 0.032 |
| Republic of Sierra Leone | 632.71 (456.9 to 842.54) | 213.87 (154.44 to 284.8) | 1274.34 (907.44 to 1714.06) | 192.8 (137.29 to 259.33) | -0.38 (-0.46 to -0.29) | <0.001 | 110.96 (67.96 to 164.36) | 37.51 (22.97 to 55.56) | 224.91 (133.94 to 335.16) | 34.03 (20.26 to 50.71) | 0.06 (-0.2 to 0.32) | 0.629 |
| Republic of Singapore | 711.06 (522.87 to 931.74) | 202.96 (149.25 to 265.96) | 2883.07 (2135.38 to 3781.16) | 245.27 (181.66 to 321.67) | 0.86 (0.6 to 1.12) | <0.001 | 132.92 (85.12 to 189.64) | 37.94 (24.3 to 54.13) | 516.6 (337.19 to 729.31) | 43.95 (28.69 to 62.04) | 0.83 (0.58 to 1.08) | <0.001 |
| Republic of Slovenia | 837.74 (616.56 to 1103.77) | 298.89 (219.97 to 393.8) | 1316.56 (977.48 to 1730.21) | 342.49 (254.28 to 450.1) | 0.69 (0.53 to 0.85) | <0.001 | 145.18 (89.31 to 212.4) | 51.8 (31.86 to 75.78) | 223.84 (141.54 to 327.54) | 58.23 (36.82 to 85.21) | 0.58 (0.38 to 0.77) | <0.001 |
| Republic of South Africa | 7890.71 (5741.33 to 10543.01) | 278.34 (202.52 to 371.89) | 18119.6 (13163.64 to 24423.99) | 278.71 (202.48 to 375.68) | 0.26 (0.14 to 0.39) | <0.001 | 1332.66 (831.19 to 1942.46) | 47.01 (29.32 to 68.52) | 3016.09 (1879.87 to 4427.95) | 46.39 (28.92 to 68.11) | 0.71 (0.61 to 0.82) | <0.001 |
| Republic of South Sudan | 1192.01 (879.58 to 1585.19) | 291.34 (214.98 to 387.44) | 1922.58 (1395 to 2561.56) | 251.61 (182.56 to 335.23) | -0.61 (-0.78 to -0.44) | <0.001 | 208.54 (130.53 to 301.71) | 50.97 (31.9 to 73.74) | 340.56 (208.11 to 501.07) | 44.57 (27.24 to 65.57) | -0.49 (-0.76 to -0.23) | 0.001 |
| Republic of Sudan | 4405.93 (3241.52 to 5803.34) | 312.8 (230.13 to 412) | 10013.44 (7288.4 to 13301.13) | 285.88 (208.08 to 379.74) | -0.41 (-0.47 to -0.35) | <0.001 | 843.03 (527.16 to 1222.71) | 59.85 (37.43 to 86.81) | 1918.63 (1199.78 to 2786.96) | 54.78 (34.25 to 79.57) | -0.57 (-0.64 to -0.5) | <0.001 |
| Republic of Suriname | 102.26 (75.01 to 134.21) | 283.93 (208.26 to 372.63) | 237.17 (174.33 to 312.66) | 285.6 (209.93 to 376.51) | -0.25 (-0.59 to 0.1) | 0.15 | 18.08 (11.36 to 26.32) | 50.2 (31.55 to 73.08) | 41.24 (25.54 to 60.17) | 49.66 (30.75 to 72.46) | -0.22 (-0.65 to 0.22) | 0.315 |
| Republic of Tajikistan | 1183.14 (873.19 to 1549.61) | 323.45 (238.72 to 423.64) | 2909.62 (2132.53 to 3858.16) | 289.39 (212.1 to 383.73) | -0.55 (-1.05 to -0.05) | 0.032 | 201.28 (127.59 to 294.63) | 55.03 (34.88 to 80.55) | 502.13 (309.68 to 742.75) | 49.94 (30.8 to 73.87) | -0.49 (-1.12 to 0.14) | 0.123 |
| Republic of Trinidad and Tobago | 269.27 (194.22 to 359.37) | 242.44 (174.86 to 323.56) | 660.89 (490.5 to 868.93) | 294.26 (218.39 to 386.89) | 0.95 (0.77 to 1.12) | <0.001 | 48.36 (29.37 to 71.25) | 43.54 (26.45 to 64.15) | 113.85 (71.39 to 167.69) | 50.69 (31.79 to 74.66) | 1.4 (1.17 to 1.64) | <0.001 |
| Republic of Tunisia | 2738.08 (2048.86 to 3576.21) | 390.52 (292.22 to 510.06) | 6380.21 (4756.47 to 8387.01) | 361.14 (269.23 to 474.74) | -0.14 (-0.46 to 0.19) | 0.403 | 510.53 (325.14 to 734.44) | 72.81 (46.37 to 104.75) | 1186.9 (751.45 to 1697.82) | 67.18 (42.53 to 96.1) | -0.28 (-0.7 to 0.13) | 0.176 |
| Republic of Turkey | 19905.99 (14655.13 to 26065.9) | 377.24 (277.73 to 493.97) | 44526.02 (33210.88 to 58475.92) | 354.66 (264.53 to 465.78) | -0.06 (-0.26 to 0.14) | 0.52 | 3758.74 (2377.45 to 5370.97) | 71.23 (45.06 to 101.79) | 8378.53 (5352.47 to 12083.71) | 66.74 (42.63 to 96.25) | -0.11 (-0.43 to 0.22) | 0.517 |
| Republic of Uganda | 2589.29 (1889.39 to 3439.3) | 265.93 (194.05 to 353.23) | 5804.09 (4168.02 to 7737.97) | 234.59 (168.47 to 312.76) | -0.65 (-0.78 to -0.52) | <0.001 | 456.53 (282.49 to 668.81) | 46.89 (29.01 to 68.69) | 1037.88 (635 to 1533.79) | 41.95 (25.67 to 61.99) | -0.23 (-0.41 to -0.05) | 0.014 |
| Republic of Uzbekistan | 4437.45 (3240.23 to 5912.33) | 307.56 (224.58 to 409.79) | 11629.58 (8491.84 to 15470.34) | 280.88 (205.1 to 373.65) | -0.23 (-0.68 to 0.23) | 0.316 | 755 (467.5 to 1113.56) | 52.33 (32.4 to 77.18) | 2015.85 (1244.3 to 2949.96) | 48.69 (30.05 to 71.25) | -0.48 (-1.1 to 0.14) | 0.123 |
| Republic of Vanuatu | 29.72 (21.28 to 39.6) | 259.49 (185.84 to 345.8) | 75.8 (54.62 to 101.27) | 253.58 (182.72 to 338.79) | 0.09 (0.03 to 0.16) | 0.005 | 5.46 (3.37 to 8.06) | 47.65 (29.4 to 70.41) | 13.87 (8.54 to 20.45) | 46.42 (28.57 to 68.43) | 0.25 (0.09 to 0.41) | 0.003 |
| Republic of Yemen | 2761.62 (2031.84 to 3617.18) | 329.71 (242.58 to 431.86) | 6731.07 (4912.22 to 8872.7) | 265.81 (193.98 to 350.38) | -0.54 (-0.62 to -0.45) | <0.001 | 529.93 (334.04 to 762.31) | 63.27 (39.88 to 91.01) | 1319.49 (831.47 to 1910.18) | 52.11 (32.83 to 75.43) | -0.61 (-0.72 to -0.5) | <0.001 |
| Republic of Zambia | 1279.49 (930.14 to 1706.84) | 272.69 (198.24 to 363.77) | 3074.23 (2216.14 to 4104.87) | 221.6 (159.74 to 295.89) | -0.81 (-0.87 to -0.75) | <0.001 | 226.05 (139.21 to 331.98) | 48.18 (29.67 to 70.75) | 562.44 (343.15 to 829.5) | 40.54 (24.74 to 59.79) | -0.57 (-0.63 to -0.5) | <0.001 |
| Republic of Zimbabwe | 1707.83 (1243.93 to 2261.21) | 273.34 (199.09 to 361.92) | 2489.62 (1792.98 to 3342.13) | 217.54 (156.67 to 292.03) | -0.61 (-0.7 to -0.53) | <0.001 | 297.69 (182.36 to 437.02) | 47.65 (29.19 to 69.95) | 452.33 (273.01 to 669.69) | 39.52 (23.85 to 58.52) | -0.36 (-0.51 to -0.21) | <0.001 |
| Republic of the Congo | 469.81 (343.63 to 620.86) | 311.15 (227.58 to 411.2) | 1516.32 (1104.34 to 2007.32) | 275.58 (200.71 to 364.82) | -0.51 (-0.65 to -0.37) | <0.001 | 82.19 (51.19 to 119.95) | 54.44 (33.9 to 79.44) | 272.29 (167.23 to 394.5) | 49.49 (30.39 to 71.7) | -0.67 (-0.89 to -0.45) | <0.001 |
| Republic of the Gambia | 131.5 (94.14 to 176.4) | 200.68 (143.66 to 269.2) | 308.77 (220.34 to 413.42) | 187.39 (133.72 to 250.9) | -0.38 (-0.47 to -0.28) | <0.001 | 23.3 (14.16 to 34.42) | 35.56 (21.61 to 52.52) | 54.48 (32.47 to 80.6) | 33.06 (19.71 to 48.91) | -0.07 (-0.31 to 0.17) | 0.565 |
| Republic of the Marshall Islands | 6.29 (4.54 to 8.41) | 232.15 (167.56 to 310.48) | 16.3 (11.73 to 21.69) | 252.77 (181.92 to 336.29) | 0.85 (0.66 to 1.04) | <0.001 | 1.17 (0.73 to 1.72) | 43.14 (26.85 to 63.38) | 2.98 (1.85 to 4.34) | 46.23 (28.62 to 67.33) | 1.02 (0.72 to 1.32) | <0.001 |
| Republic of the Niger | 1093.21 (787.06 to 1459.38) | 210.81 (151.77 to 281.42) | 2858.53 (2037.12 to 3806.65) | 217.03 (154.67 to 289.02) | 0.08 (-0.06 to 0.22) | 0.245 | 191.66 (116.2 to 285.5) | 36.96 (22.41 to 55.05) | 488.54 (290.54 to 722.42) | 37.09 (22.06 to 54.85) | 0.4 (0.15 to 0.65) | 0.003 |
| Republic of the Philippines | 16011.14 (11823.14 to 21156.46) | 330.13 (243.78 to 436.22) | 43734.66 (32283.27 to 57912.69) | 335.8 (247.87 to 444.66) | 0.09 (-0.02 to 0.2) | 0.09 | 2871.58 (1837.29 to 4093.28) | 59.21 (37.88 to 84.4) | 7689.72 (4930.32 to 10999.95) | 59.04 (37.86 to 84.46) | 0.54 (0.41 to 0.68) | <0.001 |
| Republic of the Union of Myanmar | 11914.08 (8835.62 to 15647.47) | 361.13 (267.82 to 474.29) | 22093.33 (16224.99 to 29160.72) | 328.65 (241.35 to 433.78) | -0.19 (-0.43 to 0.05) | 0.114 | 2162.66 (1359.08 to 3082.72) | 65.55 (41.2 to 93.44) | 4025.06 (2566.5 to 5788.52) | 59.87 (38.18 to 86.11) | 0.31 (0 to 0.62) | 0.05 |
| Romania | 10801.68 (7981.88 to 14166.96) | 328.92 (243.06 to 431.4) | 10435.6 (7678.88 to 13663.69) | 309.15 (227.48 to 404.78) | 0.02 (-0.09 to 0.12) | 0.762 | 1867.18 (1168.33 to 2716.85) | 56.86 (35.58 to 82.73) | 1826.25 (1155.8 to 2641.4) | 54.1 (34.24 to 78.25) | 0.03 (-0.14 to 0.21) | 0.691 |
| Russian Federation | 67221.35 (49510.68 to 88934.97) | 340.25 (250.61 to 450.16) | 79638.11 (58771.31 to 104544.29) | 348.43 (257.13 to 457.4) | 0.4 (0.05 to 0.75) | 0.027 | 11088.82 (7036.61 to 15981.18) | 56.13 (35.62 to 80.89) | 13144.27 (8398.93 to 18926.7) | 57.51 (36.75 to 82.81) | 0.46 (-0.08 to 1.01) | 0.094 |
| Saint Kitts and Nevis | 8.23 (6.01 to 10.81) | 285.3 (208.4 to 374.9) | 29.02 (21.1 to 38.25) | 274.37 (199.45 to 361.67) | 0.35 (-0.14 to 0.84) | 0.158 | 1.42 (0.87 to 2.07) | 49.11 (30.14 to 71.95) | 5 (3.05 to 7.34) | 47.23 (28.86 to 69.43) | 0.32 (-0.34 to 0.98) | 0.332 |
| Saint Lucia | 24.92 (18.13 to 33.23) | 254.48 (185.19 to 339.33) | 85.87 (62.79 to 113.14) | 281.74 (206.03 to 371.21) | 0.28 (0.1 to 0.45) | 0.004 | 4.41 (2.71 to 6.56) | 45.02 (27.65 to 66.98) | 14.83 (9.16 to 21.73) | 48.65 (30.07 to 71.3) | 0.13 (-0.09 to 0.34) | 0.229 |
| Saint Vincent and the Grenadines | 21.27 (15.64 to 28.03) | 277.49 (204.13 to 365.8) | 54.11 (39.43 to 71.94) | 296.64 (216.15 to 394.38) | 0.28 (-0.04 to 0.6) | 0.082 | 3.73 (2.31 to 5.51) | 48.62 (30.14 to 71.95) | 9.3 (5.66 to 13.76) | 51 (31.05 to 75.42) | 0.05 (-0.35 to 0.44) | 0.805 |
| Slovak Republic | 1940.52 (1428.62 to 2563.01) | 300.96 (221.57 to 397.51) | 3002.86 (2216.98 to 3953.17) | 312.85 (230.97 to 411.85) | 0.68 (0.45 to 0.9) | <0.001 | 339.09 (213.09 to 494.93) | 52.59 (33.05 to 76.76) | 521.12 (322.46 to 760.88) | 54.29 (33.6 to 79.27) | 0.75 (0.42 to 1.09) | <0.001 |
| Socialist Republic of Viet Nam | 17224.41 (12805.41 to 22476.15) | 382.95 (284.7 to 499.72) | 46661.25 (34273.18 to 61419.09) | 323.98 (237.97 to 426.45) | -0.45 (-0.95 to 0.05) | 0.076 | 3056.45 (1962.77 to 4403.09) | 67.95 (43.64 to 97.89) | 8446.61 (5369.62 to 12134.24) | 58.65 (37.28 to 84.25) | -0.29 (-0.89 to 0.32) | 0.341 |
| Solomon Islands | 72.42 (52.76 to 95.96) | 295.96 (215.64 to 392.19) | 164.54 (119.14 to 219.15) | 255.2 (184.78 to 339.9) | -0.66 (-0.72 to -0.59) | <0.001 | 13.15 (8.3 to 18.99) | 53.74 (33.93 to 77.62) | 30.65 (19.04 to 44.23) | 47.54 (29.54 to 68.59) | -0.69 (-0.89 to -0.49) | <0.001 |
| State of Eritrea | 527.3 (381.45 to 701.06) | 241.92 (175 to 321.64) | 1130.8 (813.13 to 1514.39) | 211.97 (152.42 to 283.88) | -0.64 (-0.72 to -0.56) | <0.001 | 95.04 (58.25 to 138.36) | 43.6 (26.72 to 63.48) | 207.19 (124.96 to 306.82) | 38.84 (23.42 to 57.51) | -0.26 (-0.44 to -0.08) | 0.005 |
| State of Israel | 1206.74 (868.12 to 1601.06) | 239.67 (172.42 to 317.99) | 2831.68 (2013.63 to 3802.92) | 234.69 (166.89 to 315.19) | 0.3 (0.14 to 0.45) | <0.001 | 211.11 (127.78 to 316.56) | 41.93 (25.38 to 62.87) | 489.24 (293.51 to 740.11) | 40.55 (24.33 to 61.34) | 0.67 (0.44 to 0.9) | <0.001 |
| State of Kuwait | 476.97 (347.49 to 632.99) | 253.69 (184.82 to 336.67) | 2084.81 (1513.59 to 2780.19) | 246.64 (179.06 to 328.91) | -0.36 (-0.66 to -0.06) | 0.02 | 93.9 (58.79 to 136.43) | 49.94 (31.27 to 72.57) | 406.78 (254.42 to 593.5) | 48.12 (30.1 to 70.21) | -0.87 (-1.22 to -0.52) | <0.001 |
| State of Libya | 1071.88 (793.18 to 1412.24) | 325.02 (240.51 to 428.23) | 2848.2 (2085.57 to 3773.05) | 269.85 (197.59 to 357.47) | -1.08 (-1.26 to -0.9) | <0.001 | 204.07 (128.85 to 294.35) | 61.88 (39.07 to 89.25) | 551.69 (349.6 to 797.91) | 52.27 (33.12 to 75.6) | -1.66 (-1.92 to -1.4) | <0.001 |
| State of Qatar | 110.59 (79.12 to 150.01) | 186.97 (133.77 to 253.61) | 1123.78 (795.05 to 1515.47) | 188.09 (133.07 to 253.65) | 0.16 (-0.03 to 0.35) | 0.095 | 22.9 (14.12 to 33.77) | 38.72 (23.86 to 57.09) | 230.84 (139.81 to 338.76) | 38.64 (23.4 to 56.7) | -0.24 (-0.49 to 0.02) | 0.068 |
| Sultanate of Oman | 398.62 (288.25 to 535.33) | 228.4 (165.16 to 306.73) | 1437.04 (1036.86 to 1921.03) | 206.39 (148.92 to 275.9) | -0.15 (-0.23 to -0.07) | <0.001 | 79.81 (49.37 to 117.62) | 45.73 (28.29 to 67.39) | 289.14 (178.25 to 423.11) | 41.53 (25.6 to 60.77) | -0.48 (-0.59 to -0.37) | <0.001 |
| Swiss Confederation | 2477.55 (1775.79 to 3292.98) | 239.74 (171.83 to 318.64) | 4002.28 (2863.19 to 5360.58) | 254.71 (182.22 to 341.16) | 0.11 (0.04 to 0.17) | 0.002 | 431.85 (259.78 to 643.21) | 41.79 (25.14 to 62.24) | 679.01 (405.5 to 1010.55) | 43.21 (25.81 to 64.31) | 0.35 (0.3 to 0.39) | <0.001 |
| Syrian Arab Republic | 2887.28 (2139.72 to 3801.32) | 348.05 (257.93 to 458.23) | 7163.04 (5303.53 to 9415.23) | 335.83 (248.65 to 441.43) | -0.17 (-0.44 to 0.09) | 0.193 | 546.97 (344.62 to 785.15) | 65.94 (41.54 to 94.65) | 1345.27 (851.15 to 1935.4) | 63.07 (39.91 to 90.74) | -0.21 (-0.57 to 0.14) | 0.227 |
| Taiwan (Province of China) | 5502.8 (4007.33 to 7270.19) | 240.02 (174.79 to 317.11) | 12292.77 (9020.33 to 16157.48) | 275.4 (202.08 to 361.98) | 1.15 (0.7 to 1.59) | <0.001 | 937.72 (578.41 to 1356.99) | 40.9 (25.23 to 59.19) | 2190.32 (1386.07 to 3114.12) | 49.07 (31.05 to 69.77) | 0.57 (0.04 to 1.1) | 0.036 |
| Togolese Republic | 388.69 (276.79 to 518.93) | 197.21 (140.44 to 263.29) | 1234.12 (870.14 to 1675.78) | 180.43 (127.22 to 245) | -0.34 (-0.45 to -0.23) | <0.001 | 68.6 (41.28 to 101.01) | 34.81 (20.94 to 51.25) | 218.57 (128.02 to 327.98) | 31.96 (18.72 to 47.95) | 0.01 (-0.18 to 0.2) | 0.917 |
| Tokelau | 0.61 (0.45 to 0.79) | 431.3 (318.69 to 559.62) | 0.56 (0.41 to 0.74) | 317.69 (234.23 to 420.4) | -0.32 (-0.62 to -0.01) | 0.041 | 0.1 (0.07 to 0.15) | 72.67 (46.39 to 105.67) | 0.1 (0.06 to 0.14) | 56.07 (34.98 to 80.92) | -0.93 (-1.35 to -0.52) | <0.001 |
| Turkmenistan | 784.61 (574.43 to 1034.62) | 306.88 (224.67 to 404.67) | 1684.26 (1231.65 to 2247.69) | 268.58 (196.4 to 358.42) | -0.46 (-0.87 to -0.04) | 0.033 | 135.5 (83.11 to 199.23) | 53 (32.51 to 77.92) | 294.68 (179.54 to 432.91) | 46.99 (28.63 to 69.03) | -0.69 (-1.26 to -0.12) | 0.019 |
| Tuvalu | 2.72 (1.99 to 3.59) | 329.64 (241.64 to 435.92) | 4.44 (3.27 to 5.87) | 308.26 (227.04 to 407.89) | 0.12 (-0.07 to 0.3) | 0.202 | 0.48 (0.3 to 0.7) | 58.3 (36.54 to 84.59) | 0.79 (0.5 to 1.16) | 55.19 (34.57 to 80.5) | -0.02 (-0.27 to 0.23) | 0.896 |
| Ukraine | 25875.32 (18883.06 to 34434.27) | 354.46 (258.68 to 471.71) | 24081.18 (17686.02 to 32010.87) | 338.32 (248.47 to 449.72) | -0.04 (-0.26 to 0.18) | 0.7 | 4266.69 (2651.43 to 6250.97) | 58.45 (36.32 to 85.63) | 3981.43 (2501.57 to 5810.04) | 55.94 (35.14 to 81.63) | 0.22 (-0.13 to 0.57) | 0.218 |
| Union of the Comoros | 83.83 (61.69 to 110.1) | 273.72 (201.43 to 359.5) | 192.73 (140.8 to 256.57) | 248.01 (181.18 to 330.17) | -0.57 (-0.65 to -0.48) | <0.001 | 14.64 (9.04 to 21.38) | 47.8 (29.53 to 69.81) | 34 (20.83 to 49.99) | 43.75 (26.8 to 64.32) | -0.27 (-0.42 to -0.11) | 0.001 |
| United Arab Emirates | 354.27 (245.99 to 483.06) | 149.97 (104.13 to 204.49) | 5509.48 (3875.29 to 7472.53) | 170.89 (120.2 to 231.77) | 0.23 (0.04 to 0.42) | 0.017 | 76.88 (45.97 to 114.61) | 32.54 (19.46 to 48.52) | 1143.01 (690.44 to 1701.68) | 35.45 (21.42 to 52.78) | 0.11 (-0.09 to 0.32) | 0.26 |
| United Kingdom of Great Britain and Northern Ireland | 19854.78 (14185.2 to 26400.66) | 242.9 (173.54 to 322.98) | 25308.67 (17364.4 to 34241.86) | 235.49 (161.57 to 318.61) | -0.16 (-0.2 to -0.11) | <0.001 | 3316.02 (2019.74 to 4959.65) | 40.57 (24.71 to 60.67) | 4059.66 (2381.43 to 6236.29) | 37.77 (22.16 to 58.03) | 0.14 (0.07 to 0.21) | <0.001 |
| United Mexican States | 16347.98 (11887.01 to 21716.82) | 258.04 (187.62 to 342.78) | 44747.63 (32624.79 to 59763.15) | 268.05 (195.43 to 358) | 0.11 (0.02 to 0.2) | 0.021 | 2875.29 (1794.32 to 4170.47) | 45.38 (28.32 to 65.83) | 7648.16 (4781.73 to 11195.34) | 45.81 (28.64 to 67.06) | 0.18 (0.09 to 0.27) | <0.001 |
| United Republic of Tanzania | 4503.92 (3297.33 to 5903.29) | 287.73 (210.65 to 377.13) | 10373.26 (7483.01 to 13768.95) | 243.23 (175.46 to 322.85) | -0.6 (-0.67 to -0.53) | <0.001 | 784.9 (491.7 to 1145.25) | 50.14 (31.41 to 73.16) | 1834.06 (1106.17 to 2694.85) | 43 (25.94 to 63.19) | -0.43 (-0.5 to -0.37) | <0.001 |
| United States Virgin Islands | 31.72 (23.01 to 42.71) | 235.54 (170.88 to 317.14) | 49.87 (36.77 to 65.27) | 331.39 (244.39 to 433.77) | 1.1 (0.98 to 1.22) | <0.001 | 5.69 (3.47 to 8.43) | 42.25 (25.74 to 62.6) | 8.36 (5.2 to 12.24) | 55.57 (34.54 to 81.32) | 1.45 (1.31 to 1.59) | <0.001 |

Note: CI, confidence interval; AAPC, average annual percentage change; EOAD, early-onset Alzheimer’s disease and other dementias.

**Supplementary Table S12. Age-standardized Mortality and DALYs of EOAD in men aged 40-64 years and their AAPCs from 1990 to 2021 at the country levels.**

|  | Mortality | | | | | | DALYs | | | | | |
| --- | --- | --- | --- | --- | --- | --- | --- | --- | --- | --- | --- | --- |
| Country | No of people with EOAD in 1990 (000s) | Age standardised rate in 1990 (per 100 000) | No of people with EOAD in 2021 (000s) | Age standardised rate in 2021 (per 100 000) | AAPC (95% CI) | *p* value | No of people with EOAD in 1990 (000s) | Age standardised rate in 1990 (per 100 000) | No of people with EOAD in 2021 (000s) | Age standardised rate in 2021 (per 100 000) | AAPC (95% CI) | *p* value |
| American Samoa | 0.11 (0.02 to 0.36) | 2.72 (0.52 to 8.63) | 0.23 (0.04 to 0.7) | 3.05 (0.57 to 9.45) | 0.29 (0.09 to 0.5) | 0.006 | 5.55 (2.3 to 13.32) | 134.63 (55.81 to 323.31) | 11.31 (4.63 to 26.78) | 152.22 (62.27 to 360.48) | 0.33 (0.16 to 0.51) | <0.001 |
| Antigua and Barbuda | 0.13 (0.03 to 0.4) | 2.54 (0.49 to 7.71) | 0.38 (0.07 to 1.18) | 2.72 (0.52 to 8.45) | 0.42 (0.1 to 0.75) | 0.013 | 6.54 (2.69 to 15.06) | 124.93 (51.34 to 287.84) | 18.87 (7.83 to 44.65) | 135.31 (56.12 to 320.21) | 0.42 (0.14 to 0.71) | 0.005 |
| Arab Republic of Egypt | 141.61 (26.93 to 445.92) | 2.97 (0.57 to 9.37) | 333.76 (62.86 to 1034.47) | 2.87 (0.54 to 8.91) | 0.04 (-0.07 to 0.15) | 0.459 | 7066.87 (2973.64 to 16876.7) | 148.43 (62.46 to 354.48) | 16760.74 (7224.08 to 39118.69) | 144.32 (62.2 to 336.84) | 0.07 (0 to 0.15) | 0.062 |
| Argentine Republic | 83.24 (15.74 to 265.02) | 2.18 (0.41 to 6.95) | 134.46 (25.61 to 426.92) | 2.22 (0.42 to 7.04) | 0.23 (0.16 to 0.31) | <0.001 | 4510.64 (1952.76 to 10313.58) | 118.29 (51.21 to 270.48) | 7089.05 (3050.26 to 16424.24) | 116.88 (50.29 to 270.8) | 0.11 (0.04 to 0.17) | 0.003 |
| Australia | 58.67 (11.28 to 180.44) | 2.61 (0.5 to 8.02) | 118.85 (23.22 to 373.21) | 3.04 (0.59 to 9.54) | 0.83 (0.69 to 0.97) | <0.001 | 2943.4 (1245 to 6839.52) | 130.89 (55.37 to 304.16) | 5816.38 (2389.68 to 13789.04) | 148.61 (61.06 to 352.32) | 0.69 (0.58 to 0.8) | <0.001 |
| Barbados | 0.63 (0.13 to 1.96) | 2.61 (0.52 to 8.08) | 1.62 (0.31 to 5.1) | 3.3 (0.64 to 10.38) | 1.11 (0.81 to 1.41) | <0.001 | 31.34 (13 to 72.96) | 129.28 (53.63 to 300.95) | 78.04 (33.06 to 186.17) | 158.72 (67.24 to 378.63) | 0.98 (0.73 to 1.24) | <0.001 |
| Belize | 0.34 (0.07 to 1.05) | 2.73 (0.52 to 8.41) | 1.17 (0.23 to 3.6) | 2.5 (0.48 to 7.72) | -0.09 (-0.32 to 0.14) | 0.423 | 16.79 (6.94 to 40.21) | 134.67 (55.65 to 322.46) | 58.21 (23.52 to 135.71) | 124.64 (50.36 to 290.62) | -0.09 (-0.31 to 0.13) | 0.424 |
| Bermuda | 0.19 (0.04 to 0.58) | 2.38 (0.45 to 7.29) | 0.37 (0.07 to 1.15) | 3.08 (0.59 to 9.47) | 0.91 (0.72 to 1.09) | <0.001 | 9.7 (4.15 to 22.49) | 122.44 (52.34 to 283.99) | 18.79 (7.88 to 43.31) | 154.65 (64.84 to 356.42) | 0.84 (0.68 to 1) | <0.001 |
| Bolivarian Republic of Venezuela | 40.68 (7.81 to 125.53) | 2.78 (0.53 to 8.59) | 130.54 (25.19 to 417.69) | 3.31 (0.64 to 10.59) | 0.77 (0.55 to 0.98) | <0.001 | 2112.42 (915.15 to 4833.48) | 144.6 (62.65 to 330.87) | 6511.81 (2664.87 to 15836.65) | 165.04 (67.54 to 401.36) | 0.66 (0.48 to 0.84) | <0.001 |
| Bosnia and Herzegovina | 16.83 (3.21 to 51.42) | 2.85 (0.54 to 8.71) | 18.55 (3.52 to 55.95) | 3.14 (0.59 to 9.47) | 0.2 (-0.15 to 0.55) | 0.248 | 867.32 (374.04 to 1996.57) | 146.97 (63.38 to 338.33) | 944.21 (395.12 to 2114.48) | 159.75 (66.85 to 357.74) | 0.27 (-0.02 to 0.56) | 0.066 |
| Brunei Darussalam | 0.35 (0.07 to 1.1) | 1.66 (0.32 to 5.23) | 1.44 (0.27 to 4.55) | 2.17 (0.4 to 6.84) | 1.29 (0.96 to 1.62) | <0.001 | 18.33 (7.68 to 43.68) | 87.33 (36.6 to 208.12) | 73.71 (30.5 to 172.15) | 110.87 (45.88 to 258.93) | 1.17 (0.91 to 1.44) | <0.001 |
| Burkina Faso | 16.47 (3.19 to 51.43) | 2.74 (0.53 to 8.55) | 31.77 (5.78 to 96.16) | 2.15 (0.39 to 6.52) | -0.79 (-0.98 to -0.59) | <0.001 | 777.43 (307.93 to 1912.85) | 129.3 (51.21 to 318.14) | 1538.26 (602.2 to 3655.27) | 104.34 (40.85 to 247.94) | -0.7 (-0.83 to -0.57) | <0.001 |
| Canada | 99.76 (19.33 to 313.86) | 2.74 (0.53 to 8.62) | 209.08 (41.63 to 638.11) | 3.44 (0.68 to 10.49) | 1.04 (0.79 to 1.29) | <0.001 | 5934.08 (2817.45 to 12785.88) | 162.99 (77.39 to 351.19) | 12729.04 (6460.24 to 26479.38) | 209.34 (106.25 to 435.49) | 0.94 (0.81 to 1.08) | <0.001 |
| Central African Republic | 5.51 (1.01 to 17.52) | 2.95 (0.54 to 9.37) | 10.95 (1.98 to 34.94) | 2.48 (0.45 to 7.91) | -0.73 (-1.01 to -0.45) | <0.001 | 270.86 (110.08 to 651.33) | 144.93 (58.9 to 348.49) | 548.97 (220.67 to 1334.97) | 124.24 (49.94 to 302.13) | -0.61 (-0.84 to -0.37) | <0.001 |
| Commonwealth of Dominica | 0.14 (0.03 to 0.45) | 2.53 (0.49 to 7.84) | 0.34 (0.06 to 1.08) | 3.21 (0.61 to 10.26) | 1.17 (0.85 to 1.49) | <0.001 | 7.03 (2.84 to 16.57) | 122.86 (49.69 to 289.49) | 16.44 (6.66 to 40.14) | 155.5 (63.04 to 379.71) | 1.11 (0.84 to 1.37) | <0.001 |
| Commonwealth of the Bahamas | 0.48 (0.09 to 1.49) | 2.18 (0.41 to 6.81) | 1.41 (0.26 to 4.3) | 2.46 (0.45 to 7.52) | 0.19 (-0.06 to 0.43) | 0.128 | 24.07 (9.99 to 57.62) | 109.96 (45.62 to 263.28) | 71.36 (29.18 to 166.08) | 124.92 (51.09 to 290.75) | 0.24 (0.03 to 0.45) | 0.03 |
| Cook Islands | 0.06 (0.01 to 0.2) | 3.34 (0.61 to 10.24) | 0.1 (0.02 to 0.33) | 3.72 (0.7 to 11.58) | 0.02 (-0.26 to 0.31) | 0.872 | 3.11 (1.26 to 7.37) | 161.45 (65.68 to 382.92) | 5.15 (2.14 to 12.05) | 183.14 (76.09 to 428.29) | 0.12 (-0.12 to 0.36) | 0.313 |
| Czech Republic | 39.44 (7.67 to 121.99) | 2.64 (0.51 to 8.18) | 52.01 (9.73 to 158.58) | 2.73 (0.51 to 8.32) | 0.8 (0.47 to 1.14) | <0.001 | 2009 (856.34 to 4657.55) | 134.66 (57.4 to 312.19) | 2685.04 (1148.65 to 6088.92) | 140.86 (60.26 to 319.43) | 0.68 (0.39 to 0.97) | <0.001 |
| Democratic People's Republic of Korea | 61.21 (10.61 to 192.19) | 2.55 (0.44 to 8.02) | 137.55 (25.14 to 435.35) | 3.1 (0.57 to 9.82) | -0.16 (-0.5 to 0.18) | 0.342 | 3016.16 (1187.08 to 7361.74) | 125.88 (49.54 to 307.23) | 6503.75 (2489.18 to 16015.34) | 146.67 (56.14 to 361.17) | -0.24 (-0.55 to 0.07) | 0.128 |
| Democratic Republic of Sao Tome and Principe | 0.2 (0.04 to 0.65) | 2.58 (0.49 to 8.29) | 0.4 (0.08 to 1.3) | 1.97 (0.37 to 6.39) | -1.21 (-1.49 to -0.92) | <0.001 | 9.9 (4.01 to 24.26) | 125.3 (50.71 to 307.07) | 19.44 (7.67 to 47.93) | 95.21 (37.57 to 234.76) | -1.15 (-1.39 to -0.9) | <0.001 |
| Democratic Republic of Timor-Leste | 1.22 (0.22 to 3.78) | 2.08 (0.38 to 6.43) | 3.28 (0.6 to 10.28) | 2.74 (0.51 to 8.59) | 1.14 (0.92 to 1.35) | <0.001 | 71.17 (31.89 to 154.79) | 121.07 (54.25 to 263.3) | 174.41 (75.04 to 395.97) | 145.75 (62.71 to 330.89) | 0.79 (0.61 to 0.98) | <0.001 |
| Democratic Republic of the Congo | 71.08 (13.38 to 224.98) | 3.09 (0.58 to 9.78) | 205.24 (37.58 to 646.93) | 3.01 (0.55 to 9.49) | 0.03 (-0.4 to 0.46) | 0.884 | 3541.17 (1442.29 to 8430.62) | 153.92 (62.69 to 366.46) | 9931.99 (3790.95 to 24368.11) | 145.75 (55.63 to 357.59) | -0.05 (-0.39 to 0.3) | 0.775 |
| Democratic Socialist Republic of Sri Lanka | 45.87 (8.89 to 139.46) | 2.7 (0.52 to 8.21) | 105.61 (18.76 to 329.73) | 3.33 (0.59 to 10.4) | 1.04 (0.9 to 1.17) | <0.001 | 2526.53 (1150.16 to 5529.1) | 148.72 (67.7 to 325.47) | 5505.75 (2284.11 to 12478.72) | 173.68 (72.05 to 393.65) | 0.83 (0.7 to 0.95) | <0.001 |
| Dominican Republic | 13.04 (2.45 to 40.25) | 2.43 (0.45 to 7.49) | 32.73 (5.85 to 99.78) | 2.43 (0.43 to 7.41) | 0.04 (-0.08 to 0.16) | 0.48 | 657.66 (271.69 to 1514.38) | 122.33 (50.54 to 281.69) | 1619.8 (664.17 to 3809.02) | 120.34 (49.34 to 282.98) | -0.01 (-0.11 to 0.09) | 0.787 |
| Eastern Republic of Uruguay | 9.35 (1.76 to 29.24) | 2.38 (0.45 to 7.46) | 11.89 (2.29 to 37.17) | 2.45 (0.47 to 7.65) | 0.16 (0.03 to 0.28) | 0.014 | 504.6 (218.97 to 1149.91) | 128.72 (55.86 to 293.33) | 619.68 (269.71 to 1430.03) | 127.57 (55.52 to 294.39) | 0.02 (-0.08 to 0.13) | 0.672 |
| Federal Democratic Republic of Ethiopia | 106.98 (18.88 to 340.06) | 3.18 (0.56 to 10.1) | 207.19 (38.14 to 632.02) | 2.75 (0.51 to 8.38) | -0.4 (-0.44 to -0.36) | <0.001 | 5244.36 (2179.41 to 12587.27) | 155.78 (64.74 to 373.89) | 10152.68 (4157.98 to 24057.58) | 134.58 (55.11 to 318.89) | -0.41 (-0.44 to -0.37) | <0.001 |
| Federal Democratic Republic of Nepal | 26.37 (4.87 to 83.58) | 1.67 (0.31 to 5.29) | 66.54 (12.13 to 201.55) | 2.2 (0.4 to 6.65) | 1.04 (0.94 to 1.13) | <0.001 | 1452.28 (645.92 to 3200.56) | 91.94 (40.89 to 202.61) | 3266.1 (1290.06 to 7475.59) | 107.77 (42.57 to 246.67) | 0.6 (0.53 to 0.66) | <0.001 |
| Federal Republic of Germany | 321.48 (60.48 to 1006.13) | 2.54 (0.48 to 7.94) | 447.7 (86.67 to 1387.35) | 3.02 (0.58 to 9.34) | 0.15 (-0.15 to 0.44) | 0.313 | 17223.14 (7526.29 to 39305.59) | 135.87 (59.37 to 310.06) | 23414.63 (10322.08 to 53843.21) | 157.71 (69.53 to 362.67) | 0.14 (-0.12 to 0.41) | 0.272 |
| Federal Republic of Nigeria | 167.95 (31.36 to 540.46) | 2.13 (0.4 to 6.85) | 288.16 (52.45 to 947.35) | 1.88 (0.34 to 6.17) | -0.48 (-0.61 to -0.35) | <0.001 | 8431 (3614.36 to 20327.89) | 106.79 (45.78 to 257.49) | 14376.56 (5983.46 to 35880.86) | 93.56 (38.94 to 233.52) | -0.5 (-0.6 to -0.4) | <0.001 |
| Federal Republic of Somalia | 10.58 (1.92 to 34.34) | 2.12 (0.38 to 6.89) | 25.46 (4.45 to 80.55) | 2.05 (0.36 to 6.48) | 0.8 (0.35 to 1.25) | 0.001 | 539.41 (215.11 to 1336.34) | 108.24 (43.16 to 268.16) | 1239.55 (484.02 to 3025.02) | 99.66 (38.92 to 243.21) | 0.51 (0.09 to 0.93) | 0.018 |
| Federated States of Micronesia | 0.24 (0.05 to 0.77) | 3.38 (0.66 to 10.91) | 0.47 (0.09 to 1.49) | 3.76 (0.69 to 11.96) | 0.85 (0.45 to 1.25) | <0.001 | 11.07 (4.32 to 27.76) | 156.68 (61.16 to 393.03) | 21.98 (8.71 to 54.63) | 176.1 (69.77 to 437.67) | 0.87 (0.55 to 1.2) | <0.001 |
| Federative Republic of Brazil | 477.81 (91.96 to 1508.54) | 3.65 (0.7 to 11.52) | 1291.41 (249.56 to 4041.22) | 4.15 (0.8 to 12.97) | 0.5 (0.36 to 0.65) | <0.001 | 23709.69 (10247.57 to 56556.09) | 181.05 (78.25 to 431.87) | 63376.06 (27338.53 to 151204.92) | 203.42 (87.75 to 485.32) | 0.5 (0.38 to 0.62) | <0.001 |
| French Republic | 189.86 (35.97 to 596.22) | 2.38 (0.45 to 7.47) | 252.6 (47.42 to 777.23) | 2.44 (0.46 to 7.5) | 0.41 (0.21 to 0.62) | <0.001 | 9149.6 (3808.36 to 22167.52) | 114.69 (47.74 to 277.87) | 12345.63 (4971.51 to 28748.26) | 119.16 (47.98 to 277.47) | 0.42 (0.26 to 0.59) | <0.001 |
| Gabonese Republic | 2.7 (0.51 to 8.25) | 3.88 (0.74 to 11.87) | 6.18 (1.14 to 19.45) | 3.55 (0.66 to 11.18) | 0.01 (-0.34 to 0.36) | 0.954 | 125.99 (49.96 to 305.75) | 181.34 (71.91 to 440.08) | 289.89 (114.05 to 720.67) | 166.57 (65.53 to 414.08) | 0.04 (-0.26 to 0.34) | 0.779 |
| Georgia | 21.51 (4.13 to 65.81) | 3.04 (0.58 to 9.3) | 17.79 (3.44 to 53.22) | 3.24 (0.63 to 9.68) | 0.06 (-0.39 to 0.51) | 0.795 | 1110.47 (468.93 to 2550.52) | 156.92 (66.26 to 360.41) | 884.47 (379.48 to 2011.11) | 160.94 (69.05 to 365.94) | 0.02 (-0.36 to 0.4) | 0.909 |
| Grand Duchy of Luxembourg | 1.19 (0.22 to 3.63) | 2 (0.38 to 6.13) | 2.3 (0.44 to 7.12) | 1.98 (0.38 to 6.13) | -0.11 (-0.29 to 0.06) | 0.198 | 59.77 (25.24 to 136.97) | 100.96 (42.63 to 231.39) | 114.92 (47.72 to 266.8) | 98.92 (41.07 to 229.65) | -0.11 (-0.27 to 0.05) | 0.173 |
| Greenland | 0.17 (0.03 to 0.52) | 2.25 (0.42 to 6.95) | 0.4 (0.08 to 1.23) | 3.95 (0.76 to 12.23) | 1.51 (1.22 to 1.79) | <0.001 | 9.52 (4.27 to 21.24) | 127.44 (57.12 to 284.27) | 21.13 (9.17 to 47.87) | 209.83 (91.09 to 475.36) | 1.35 (1.1 to 1.6) | <0.001 |
| Grenada | 0.18 (0.04 to 0.56) | 2.95 (0.56 to 8.99) | 0.44 (0.08 to 1.38) | 2.73 (0.51 to 8.6) | 0.04 (-0.17 to 0.25) | 0.704 | 9.08 (3.8 to 20.88) | 145.05 (60.69 to 333.52) | 21.92 (8.8 to 50.7) | 136.78 (54.9 to 316.44) | 0.06 (-0.12 to 0.24) | 0.487 |
| Guam | 0.42 (0.08 to 1.3) | 2.92 (0.55 to 9.13) | 0.86 (0.16 to 2.73) | 3.54 (0.67 to 11.28) | 0.74 (0.41 to 1.06) | <0.001 | 20.46 (8.43 to 48.84) | 143.51 (59.09 to 342.51) | 42.22 (17.47 to 102.29) | 174.59 (72.27 to 423) | 0.73 (0.47 to 1) | <0.001 |
| Hashemite Kingdom of Jordan | 6.61 (1.29 to 20.41) | 2.64 (0.51 to 8.13) | 35.39 (6.64 to 107.21) | 2.29 (0.43 to 6.92) | -1.2 (-1.5 to -0.91) | <0.001 | 351.07 (150.68 to 805.67) | 139.94 (60.06 to 321.13) | 1950.1 (849.1 to 4288.66) | 125.91 (54.82 to 276.91) | -0.95 (-1.18 to -0.71) | <0.001 |
| Hellenic Republic | 41.61 (8 to 129.08) | 2.65 (0.51 to 8.23) | 44.87 (8.62 to 136.51) | 2.5 (0.48 to 7.6) | -0.29 (-0.44 to -0.15) | <0.001 | 2112.92 (906.59 to 4888.16) | 134.76 (57.82 to 311.75) | 2270.68 (978.35 to 5251.79) | 126.43 (54.47 to 292.41) | -0.3 (-0.42 to -0.18) | <0.001 |
| Hungary | 42.24 (8.1 to 132.77) | 2.81 (0.54 to 8.83) | 45.43 (8.56 to 139.33) | 2.69 (0.51 to 8.25) | 0.55 (0.31 to 0.79) | <0.001 | 2143.25 (896.37 to 4894.87) | 142.54 (59.61 to 325.54) | 2338.49 (1014 to 5419.96) | 138.49 (60.05 to 320.97) | 0.5 (0.28 to 0.72) | <0.001 |
| Independent State of Papua New Guinea | 9.58 (1.74 to 30.31) | 2.9 (0.53 to 9.17) | 26.97 (4.91 to 86.52) | 2.67 (0.49 to 8.56) | -0.3 (-0.48 to -0.11) | 0.003 | 481.11 (201.32 to 1151.92) | 145.49 (60.88 to 348.35) | 1355.08 (546.97 to 3203.49) | 134.14 (54.14 to 317.1) | -0.28 (-0.43 to -0.13) | 0.001 |
| Independent State of Samoa | 0.47 (0.09 to 1.45) | 3.84 (0.7 to 11.89) | 0.75 (0.14 to 2.33) | 3.4 (0.63 to 10.5) | -0.47 (-0.82 to -0.12) | 0.01 | 21.49 (8.46 to 53.87) | 176.07 (69.32 to 441.33) | 35.75 (14.23 to 86.32) | 161.22 (64.18 to 389.31) | -0.34 (-0.64 to -0.05) | 0.025 |
| Ireland | 9.45 (1.8 to 29.19) | 2.22 (0.42 to 6.85) | 18.92 (3.65 to 57.34) | 2.36 (0.45 to 7.14) | 0.35 (0.28 to 0.42) | <0.001 | 479.53 (202.38 to 1108.29) | 112.55 (47.5 to 260.13) | 939.13 (398.33 to 2190.26) | 116.96 (49.61 to 272.78) | 0.23 (0.18 to 0.28) | <0.001 |
| Islamic Republic of Afghanistan | 43.27 (7.75 to 130.06) | 4.58 (0.82 to 13.75) | 30.73 (5.31 to 94.04) | 1.5 (0.26 to 4.58) | -4.68 (-5.64 to -3.7) | <0.001 | 2006.28 (779.04 to 4738.83) | 212.17 (82.39 to 501.15) | 1714.63 (686.13 to 3905.07) | 83.6 (33.45 to 190.4) | -4.21 (-5.07 to -3.33) | <0.001 |
| Islamic Republic of Iran | 154.67 (29.62 to 476.35) | 3.66 (0.7 to 11.29) | 352.38 (66.81 to 1083.79) | 2.85 (0.54 to 8.75) | -0.85 (-1.19 to -0.51) | <0.001 | 7846.07 (3428.31 to 17833.96) | 185.91 (81.23 to 422.58) | 18472.97 (8269.59 to 42492.25) | 149.21 (66.8 to 343.22) | -0.67 (-0.96 to -0.37) | <0.001 |
| Islamic Republic of Mauritania | 2.97 (0.55 to 9.32) | 2.17 (0.4 to 6.83) | 8.08 (1.45 to 25.59) | 2.51 (0.45 to 7.97) | 0.44 (0.25 to 0.63) | <0.001 | 146.62 (60.35 to 345.97) | 107.37 (44.2 to 253.36) | 379.01 (148.72 to 933.07) | 118.01 (46.3 to 290.51) | 0.33 (0.17 to 0.48) | <0.001 |
| Islamic Republic of Pakistan | 180.47 (31.77 to 554.88) | 2.09 (0.37 to 6.44) | 449.19 (84.65 to 1433.48) | 2.06 (0.39 to 6.57) | -0.17 (-0.23 to -0.1) | <0.001 | 9284.88 (3984.22 to 21512.24) | 107.71 (46.22 to 249.57) | 22275.27 (8919.8 to 54663.57) | 102.11 (40.89 to 250.58) | -0.26 (-0.33 to -0.2) | <0.001 |
| Jamaica | 4.94 (0.97 to 15.18) | 2.66 (0.52 to 8.16) | 10.3 (1.98 to 32.82) | 2.76 (0.53 to 8.81) | 0.01 (-0.16 to 0.19) | 0.868 | 258.79 (113.2 to 585.19) | 139.22 (60.9 to 314.81) | 532.46 (229 to 1242.26) | 142.86 (61.44 to 333.31) | 0.02 (-0.15 to 0.18) | 0.833 |
| Japan | 587.26 (111.31 to 1865.89) | 2.77 (0.52 to 8.79) | 666.72 (128.4 to 2093.38) | 3.09 (0.59 to 9.69) | 0.47 (0.22 to 0.72) | 0.001 | 29719.32 (13018.92 to 70078.34) | 140 (61.33 to 330.13) | 33788.16 (14963.03 to 79455.17) | 156.39 (69.26 to 367.75) | 0.44 (0.21 to 0.68) | 0.001 |
| Kingdom of Bahrain | 0.98 (0.18 to 3.1) | 2.05 (0.37 to 6.48) | 5.94 (1.08 to 17.83) | 1.86 (0.34 to 5.58) | -0.08 (-0.52 to 0.37) | 0.732 | 52.35 (22.27 to 122.65) | 109.54 (46.61 to 256.67) | 337.51 (145.43 to 743.65) | 105.65 (45.53 to 232.79) | 0.18 (-0.19 to 0.56) | 0.325 |
| Kingdom of Belgium | 39.96 (7.46 to 126.54) | 2.69 (0.5 to 8.53) | 55.09 (10.51 to 170.04) | 2.88 (0.55 to 8.89) | 0.25 (0.04 to 0.46) | 0.019 | 2045.79 (887.45 to 4780.33) | 137.88 (59.81 to 322.19) | 2732.97 (1131.13 to 6402.49) | 142.94 (59.16 to 334.85) | 0.17 (-0.01 to 0.34) | 0.067 |
| Kingdom of Bhutan | 0.87 (0.15 to 2.82) | 2.02 (0.36 to 6.53) | 2.02 (0.35 to 6.28) | 2.22 (0.38 to 6.91) | 0.39 (0.33 to 0.45) | <0.001 | 43.94 (17.4 to 105.06) | 101.71 (40.29 to 243.21) | 95.08 (35.91 to 235.35) | 104.53 (39.47 to 258.73) | 0.18 (0.13 to 0.23) | <0.001 |
| Kingdom of Cambodia | 19.72 (3.77 to 62.11) | 3.1 (0.59 to 9.76) | 67.12 (12.28 to 214.31) | 3.84 (0.7 to 12.28) | 0.69 (0.41 to 0.98) | <0.001 | 994.62 (414.33 to 2333.52) | 156.31 (65.11 to 366.72) | 3165.82 (1274.43 to 7927.78) | 181.35 (73.01 to 454.14) | 0.46 (0.21 to 0.71) | 0.001 |
| Kingdom of Denmark | 18.93 (3.62 to 59.49) | 2.45 (0.47 to 7.69) | 27.24 (5.25 to 82.11) | 2.87 (0.55 to 8.65) | 0.71 (0.54 to 0.89) | <0.001 | 923.78 (378.81 to 2213.7) | 119.45 (48.98 to 286.24) | 1288.49 (518.14 to 3054.38) | 135.68 (54.56 to 321.62) | 0.5 (0.36 to 0.65) | <0.001 |
| Kingdom of Eswatini | 1.11 (0.21 to 3.43) | 2.34 (0.43 to 7.22) | 2.11 (0.37 to 6.55) | 2.3 (0.41 to 7.14) | 0.17 (0 to 0.34) | 0.049 | 55.86 (22.34 to 135.95) | 117.63 (47.05 to 286.27) | 102.02 (39.34 to 251.11) | 111.15 (42.87 to 273.61) | -0.02 (-0.15 to 0.12) | 0.8 |
| Kingdom of Lesotho | 2.1 (0.39 to 6.66) | 2.19 (0.41 to 6.95) | 3.53 (0.65 to 10.71) | 2.35 (0.43 to 7.14) | 1.07 (0.72 to 1.42) | <0.001 | 110.43 (45.23 to 255.89) | 115.27 (47.22 to 267.11) | 173.3 (68.4 to 412.01) | 115.48 (45.58 to 274.55) | 0.72 (0.43 to 1.02) | <0.001 |
| Kingdom of Morocco | 61.14 (11.65 to 184.14) | 3.09 (0.59 to 9.32) | 169.45 (29.98 to 533.45) | 3.41 (0.6 to 10.74) | 0.54 (0.22 to 0.86) | 0.002 | 3116.92 (1335.24 to 7058.7) | 157.74 (67.57 to 357.21) | 8289.5 (3407.51 to 19949.79) | 166.86 (68.59 to 401.58) | 0.39 (0.12 to 0.66) | 0.007 |
| Kingdom of Norway | 14.91 (2.85 to 46.32) | 2.58 (0.49 to 8.01) | 24.43 (4.8 to 74.45) | 2.73 (0.54 to 8.32) | 0.54 (0.37 to 0.72) | <0.001 | 759.59 (332.12 to 1768.47) | 131.39 (57.45 to 305.91) | 1232.14 (536.24 to 2846.9) | 137.61 (59.89 to 317.95) | 0.39 (0.26 to 0.51) | <0.001 |
| Kingdom of Saudi Arabia | 31.92 (5.71 to 98.69) | 2.23 (0.4 to 6.9) | 112.98 (21.04 to 353.8) | 1.73 (0.32 to 5.41) | -0.74 (-0.82 to -0.66) | <0.001 | 1639.18 (683.74 to 3832.44) | 114.59 (47.8 to 267.91) | 6012.83 (2512 to 13919.49) | 92 (38.44 to 212.98) | -0.66 (-0.73 to -0.59) | <0.001 |
| Kingdom of Spain | 142.87 (27.13 to 440.23) | 2.69 (0.51 to 8.28) | 209.97 (39.7 to 638.45) | 2.42 (0.46 to 7.35) | -0.56 (-0.75 to -0.38) | <0.001 | 6931.44 (2876.74 to 16507.09) | 130.34 (54.09 to 310.4) | 10287.83 (4248.55 to 24251.43) | 118.38 (48.89 to 279.06) | -0.44 (-0.59 to -0.29) | <0.001 |
| Kingdom of Sweden | 27.66 (5.23 to 84.51) | 2.14 (0.41 to 6.54) | 38.51 (7.29 to 117.63) | 2.39 (0.45 to 7.31) | 0.52 (0.32 to 0.72) | <0.001 | 1437.3 (619.82 to 3291.89) | 111.27 (47.98 to 254.84) | 2003.07 (882.1 to 4495.81) | 124.52 (54.83 to 279.48) | 0.46 (0.3 to 0.63) | <0.001 |
| Kingdom of Thailand | 177.87 (33.85 to 535.17) | 3.19 (0.61 to 9.6) | 509.03 (94.33 to 1603.29) | 4.07 (0.75 to 12.81) | 0.61 (0.32 to 0.9) | <0.001 | 8788.83 (3573.83 to 20537.57) | 157.74 (64.14 to 368.6) | 24012.97 (9611.24 to 58687.77) | 191.9 (76.81 to 469.01) | 0.64 (0.4 to 0.88) | <0.001 |
| Kingdom of Tonga | 0.28 (0.05 to 0.86) | 3.69 (0.7 to 11.4) | 0.36 (0.07 to 1.15) | 3.44 (0.66 to 10.83) | -0.58 (-0.76 to -0.4) | <0.001 | 13.33 (5.39 to 31.69) | 177.15 (71.63 to 421.17) | 17.5 (6.94 to 42.57) | 165.17 (65.52 to 401.75) | -0.55 (-0.72 to -0.38) | <0.001 |
| Kingdom of the Netherlands | 49.58 (9.58 to 153.71) | 2.32 (0.45 to 7.18) | 85.1 (15.97 to 259.4) | 2.95 (0.55 to 8.98) | 0.87 (0.77 to 0.98) | <0.001 | 2484.94 (1034.87 to 5774.86) | 116.08 (48.34 to 269.77) | 4173.79 (1741.85 to 9671.58) | 144.44 (60.28 to 334.7) | 0.8 (0.71 to 0.89) | <0.001 |
| Kyrgyz Republic | 10.39 (1.96 to 32.87) | 2.9 (0.55 to 9.16) | 18.71 (3.43 to 60.58) | 2.58 (0.47 to 8.35) | -0.65 (-1.36 to 0.05) | 0.069 | 533.52 (227.28 to 1236.49) | 148.75 (63.37 to 344.74) | 975.3 (423.63 to 2282.07) | 134.49 (58.42 to 314.68) | -0.43 (-1.05 to 0.19) | 0.163 |
| Lao People's Democratic Republic | 8.33 (1.52 to 27.11) | 2.77 (0.51 to 9.03) | 21.56 (3.98 to 65.24) | 2.8 (0.52 to 8.48) | 0.09 (-0.07 to 0.24) | 0.268 | 440.13 (187.56 to 1037.9) | 146.58 (62.46 to 345.65) | 1120.18 (480.06 to 2551.8) | 145.57 (62.39 to 331.61) | 0.04 (-0.11 to 0.19) | 0.571 |
| Lebanese Republic | 9.18 (1.8 to 29.25) | 2.96 (0.58 to 9.42) | 16.38 (3.08 to 52.6) | 2.54 (0.48 to 8.16) | -0.43 (-0.62 to -0.23) | <0.001 | 501.87 (228.77 to 1143.22) | 161.64 (73.68 to 368.21) | 892.41 (394.07 to 2012.58) | 138.42 (61.12 to 312.18) | -0.48 (-0.61 to -0.35) | <0.001 |
| Malaysia | 45.94 (8.75 to 143.45) | 3.03 (0.58 to 9.48) | 143.44 (27.69 to 455.75) | 3.51 (0.68 to 11.15) | 0.79 (0.6 to 0.98) | <0.001 | 2292.77 (954.78 to 5376.71) | 151.45 (63.07 to 355.15) | 6952.08 (2879.7 to 16893.11) | 170.08 (70.45 to 413.27) | 0.71 (0.55 to 0.87) | <0.001 |
| Mongolia | 3.36 (0.62 to 10.13) | 2.3 (0.42 to 6.95) | 8.41 (1.56 to 26.32) | 2.11 (0.39 to 6.59) | -0.89 (-1.36 to -0.42) | 0.001 | 177.37 (74.57 to 402.08) | 121.71 (51.17 to 275.91) | 454.03 (196.72 to 1033.36) | 113.76 (49.29 to 258.92) | -0.67 (-1.08 to -0.25) | 0.003 |
| Montenegro | 2.29 (0.43 to 7.12) | 2.91 (0.54 to 9.03) | 3.06 (0.6 to 9.81) | 3.01 (0.59 to 9.66) | 0.17 (-0.15 to 0.5) | 0.283 | 117.73 (49.33 to 271.45) | 149.4 (62.59 to 344.44) | 157.42 (69.62 to 365.67) | 154.99 (68.55 to 360.03) | 0.24 (-0.02 to 0.5) | 0.073 |
| New Zealand | 12.34 (2.39 to 37.64) | 2.86 (0.55 to 8.71) | 24.68 (4.82 to 76.51) | 3.2 (0.62 to 9.92) | 0.63 (0.49 to 0.77) | <0.001 | 646.3 (279.59 to 1460.78) | 149.55 (64.7 to 338.03) | 1273.41 (562.07 to 2900.43) | 165.05 (72.85 to 375.93) | 0.52 (0.4 to 0.63) | <0.001 |
| North Macedonia | 6.93 (1.33 to 21.4) | 2.7 (0.52 to 8.34) | 11.23 (2.09 to 35.14) | 2.83 (0.53 to 8.85) | 0.2 (0.02 to 0.38) | 0.028 | 358.4 (150.72 to 830.35) | 139.66 (58.73 to 323.56) | 586.43 (253.09 to 1328.93) | 147.63 (63.72 to 334.55) | 0.26 (0.1 to 0.42) | 0.002 |
| Northern Mariana Islands | 0.1 (0.02 to 0.32) | 1.81 (0.33 to 5.8) | 0.3 (0.06 to 0.93) | 3.31 (0.63 to 10.15) | 2.24 (1.68 to 2.79) | <0.001 | 5.31 (2.22 to 12.74) | 96.01 (40.16 to 230.18) | 15.09 (6.26 to 36.09) | 164.17 (68.07 to 392.53) | 2.03 (1.58 to 2.48) | <0.001 |
| Palestine | 3.3 (0.6 to 10.15) | 3.04 (0.55 to 9.35) | 11.42 (2.24 to 37.12) | 2.47 (0.48 to 8.01) | -0.89 (-1.15 to -0.64) | <0.001 | 169.84 (71.3 to 399.05) | 156.44 (65.67 to 367.58) | 610.88 (264.7 to 1424.54) | 131.87 (57.14 to 307.51) | -0.7 (-0.94 to -0.45) | <0.001 |
| People's Democratic Republic of Algeria | 55.32 (10.64 to 173.4) | 3.27 (0.63 to 10.26) | 168.21 (32.42 to 510.32) | 3.04 (0.59 to 9.22) | -0.17 (-0.4 to 0.06) | 0.148 | 2787.69 (1190.07 to 6537.05) | 164.98 (70.43 to 386.86) | 8487.16 (3523.67 to 19560.13) | 153.28 (63.64 to 353.26) | -0.15 (-0.35 to 0.05) | 0.136 |
| People's Republic of Bangladesh | 170.83 (32.59 to 536.73) | 2.17 (0.41 to 6.83) | 457.99 (84.92 to 1402.13) | 2.45 (0.45 to 7.51) | 0.51 (0.37 to 0.65) | <0.001 | 8216.44 (3262.23 to 19878.64) | 104.58 (41.52 to 253.01) | 21516.01 (8236.29 to 52817.32) | 115.21 (44.1 to 282.82) | 0.45 (0.31 to 0.58) | <0.001 |
| People's Republic of China | 4883.01 (913.18 to 14785.2) | 3.8 (0.71 to 11.5) | 10009.87 (1819.51 to 32851.91) | 3.92 (0.71 to 12.85) | 0.18 (-0.02 to 0.38) | 0.081 | 235584.23 (97900.54 to 563813.09) | 183.18 (76.12 to 438.4) | 520445.4 (228842.61 to 1251826.87) | 203.59 (89.52 to 489.69) | 0.36 (0.17 to 0.54) | 0.001 |
| Plurinational State of Bolivia | 10.91 (2 to 34.08) | 2.29 (0.42 to 7.15) | 31.54 (5.85 to 99.34) | 2.42 (0.45 to 7.61) | 0.39 (0.31 to 0.46) | <0.001 | 529.42 (206.05 to 1275.12) | 111.08 (43.23 to 267.53) | 1518.21 (595.34 to 3708.37) | 116.27 (45.59 to 284) | 0.34 (0.27 to 0.4) | <0.001 |
| Portuguese Republic | 35.73 (6.86 to 111.49) | 2.6 (0.5 to 8.11) | 47.77 (9.3 to 144.66) | 2.6 (0.51 to 7.86) | 0.07 (-0.04 to 0.18) | 0.196 | 1776.88 (747.74 to 4171.41) | 129.27 (54.4 to 303.47) | 2377.06 (1005.3 to 5480.43) | 129.2 (54.64 to 297.89) | 0.07 (-0.03 to 0.16) | 0.161 |
| Principality of Andorra | 0.17 (0.03 to 0.57) | 2.22 (0.42 to 7.22) | 0.42 (0.07 to 1.31) | 2.22 (0.39 to 6.94) | 0.01 (-0.18 to 0.21) | 0.901 | 8.89 (3.68 to 21.69) | 112.93 (46.78 to 275.67) | 21.71 (9.02 to 50.24) | 114.97 (47.76 to 266.09) | 0.06 (-0.1 to 0.22) | 0.418 |
| Principality of Monaco | 0.14 (0.03 to 0.43) | 2.66 (0.52 to 8.42) | 0.19 (0.04 to 0.59) | 2.85 (0.54 to 8.64) | 0.22 (0.11 to 0.33) | <0.001 | 6.84 (2.95 to 16.27) | 133.82 (57.78 to 318.25) | 9.78 (4.05 to 22.06) | 143.21 (59.28 to 322.93) | 0.17 (0.07 to 0.26) | 0.002 |
| Puerto Rico | 10.37 (1.97 to 32.04) | 2.56 (0.49 to 7.92) | 16.5 (3.11 to 49.29) | 3.22 (0.61 to 9.62) | 0.85 (0.78 to 0.92) | <0.001 | 520.84 (216.84 to 1212.06) | 128.71 (53.59 to 299.53) | 804.21 (330.8 to 1874.4) | 157.04 (64.59 to 366.02) | 0.72 (0.67 to 0.77) | <0.001 |
| Republic of Albania | 8.46 (1.57 to 25.73) | 2.58 (0.48 to 7.83) | 14.57 (2.74 to 46.73) | 3.54 (0.67 to 11.34) | 0.88 (0.65 to 1.11) | <0.001 | 438.06 (184.23 to 1005.89) | 133.4 (56.1 to 306.31) | 744.6 (326.02 to 1756.02) | 180.72 (79.13 to 426.2) | 0.92 (0.71 to 1.13) | <0.001 |
| Republic of Angola | 20.22 (3.74 to 66.09) | 2.8 (0.52 to 9.15) | 62.14 (11.21 to 189.98) | 2.91 (0.53 to 8.91) | 0.22 (-0.01 to 0.45) | 0.059 | 1017.09 (421.44 to 2527.21) | 140.8 (58.34 to 349.85) | 3004.17 (1169.55 to 7154.29) | 140.89 (54.85 to 335.53) | 0.08 (-0.1 to 0.26) | 0.37 |
| Republic of Armenia | 11.05 (2.11 to 34.34) | 3.06 (0.58 to 9.51) | 14.36 (2.82 to 43.84) | 3.34 (0.66 to 10.21) | 0.4 (-0.37 to 1.18) | 0.297 | 580.31 (251.36 to 1309.62) | 160.75 (69.63 to 362.77) | 726.52 (317.9 to 1660.21) | 169.21 (74.04 to 386.68) | 0.4 (-0.27 to 1.07) | 0.233 |
| Republic of Austria | 26.35 (5.03 to 81.62) | 2.37 (0.45 to 7.35) | 40.63 (7.72 to 127.28) | 2.59 (0.49 to 8.13) | 0.17 (0.01 to 0.33) | 0.042 | 1334.91 (554.82 to 3126.62) | 120.26 (49.98 to 281.66) | 2045.88 (857.37 to 4856.31) | 130.63 (54.74 to 310.07) | 0.12 (-0.03 to 0.27) | 0.109 |
| Republic of Azerbaijan | 18.97 (3.56 to 60.65) | 2.89 (0.54 to 9.25) | 43.16 (8.49 to 132.04) | 2.8 (0.55 to 8.58) | -0.77 (-1.64 to 0.1) | 0.081 | 999.2 (435.19 to 2356.4) | 152.4 (66.38 to 359.42) | 2261.47 (981.44 to 5075.18) | 146.92 (63.76 to 329.72) | -0.55 (-1.3 to 0.22) | 0.153 |
| Republic of Belarus | 38.71 (7.49 to 120.24) | 2.83 (0.55 to 8.79) | 47.88 (8.95 to 147.14) | 3.12 (0.58 to 9.58) | 0.23 (-0.27 to 0.74) | 0.355 | 2013.02 (864.75 to 4618.85) | 147.24 (63.25 to 337.83) | 2410.75 (1011.12 to 5492.8) | 156.96 (65.83 to 357.62) | 0.25 (-0.21 to 0.71) | 0.273 |
| Republic of Benin | 6.16 (1.18 to 19.55) | 2.34 (0.45 to 7.41) | 17.86 (3.32 to 53.71) | 2.06 (0.38 to 6.18) | -0.4 (-0.55 to -0.25) | <0.001 | 302.2 (122.13 to 730.75) | 114.54 (46.29 to 276.97) | 885.28 (336.62 to 2066.67) | 101.85 (38.73 to 237.78) | -0.35 (-0.46 to -0.24) | <0.001 |
| Republic of Botswana | 2.04 (0.38 to 6.51) | 2.52 (0.47 to 8.04) | 5.61 (1.01 to 18.07) | 2.19 (0.4 to 7.07) | -0.37 (-0.43 to -0.32) | <0.001 | 102.59 (42.65 to 244.81) | 126.67 (52.66 to 302.28) | 281.44 (113.69 to 698.66) | 110.18 (44.51 to 273.51) | -0.35 (-0.41 to -0.29) | <0.001 |
| Republic of Bulgaria | 41.77 (7.99 to 130.45) | 3.02 (0.58 to 9.42) | 34.43 (6.72 to 105.19) | 2.82 (0.55 to 8.62) | -0.01 (-0.16 to 0.14) | 0.902 | 2125.19 (915.49 to 4928.27) | 153.5 (66.12 to 355.96) | 1771.01 (755.24 to 4081.44) | 145.12 (61.89 to 334.44) | 0.01 (-0.11 to 0.13) | 0.854 |
| Republic of Burundi | 7.6 (1.38 to 24.31) | 2.43 (0.44 to 7.78) | 24.71 (4.63 to 74.52) | 2.63 (0.49 to 7.93) | 0.62 (0.28 to 0.96) | 0.001 | 388.77 (161.96 to 932.17) | 124.36 (51.81 to 298.18) | 1204.11 (472.5 to 2842.77) | 128.1 (50.27 to 302.42) | 0.46 (0.18 to 0.75) | 0.002 |
| Republic of Cabo Verde | 0.57 (0.11 to 1.75) | 3.17 (0.59 to 9.72) | 1.63 (0.3 to 5.21) | 2.45 (0.45 to 7.81) | -1.56 (-2.38 to -0.74) | 0.001 | 27.27 (10.82 to 64.65) | 151.58 (60.12 to 359.37) | 76.8 (29.48 to 188.41) | 115.26 (44.25 to 282.75) | -1.37 (-2.09 to -0.64) | 0.001 |
| Republic of Cameroon | 18.28 (3.47 to 57.07) | 2.58 (0.49 to 8.05) | 48.82 (8.47 to 153) | 2.15 (0.37 to 6.73) | -0.54 (-0.72 to -0.36) | <0.001 | 854.6 (327.45 to 2112.15) | 120.56 (46.19 to 297.96) | 2306.62 (868.39 to 5716.21) | 101.47 (38.2 to 251.47) | -0.5 (-0.64 to -0.36) | <0.001 |
| Republic of Chad | 7.52 (1.37 to 23.48) | 2.13 (0.39 to 6.65) | 23.8 (4.12 to 74.28) | 2.31 (0.4 to 7.2) | 0.28 (0.19 to 0.37) | <0.001 | 379.78 (155.79 to 880.45) | 107.55 (44.12 to 249.33) | 1130.46 (435.79 to 2786.23) | 109.51 (42.22 to 269.91) | 0.09 (0.01 to 0.17) | 0.026 |
| Republic of Chile | 26.79 (5.15 to 84.64) | 2.03 (0.39 to 6.43) | 68.76 (13.2 to 215.04) | 2.48 (0.48 to 7.77) | 0.56 (0.32 to 0.8) | <0.001 | 1465.65 (655.35 to 3315.08) | 111.28 (49.76 to 251.69) | 3630.85 (1576.81 to 8375.9) | 131.14 (56.95 to 302.53) | 0.48 (0.28 to 0.69) | <0.001 |
| Republic of Colombia | 65.76 (12.52 to 206.91) | 2.45 (0.47 to 7.7) | 190.92 (36.5 to 588.3) | 3.01 (0.58 to 9.28) | 0.71 (0.52 to 0.91) | <0.001 | 3390.37 (1427.7 to 7992.9) | 126.16 (53.13 to 297.42) | 9589.95 (4023.6 to 21971.55) | 151.27 (63.47 to 346.58) | 0.65 (0.49 to 0.82) | <0.001 |
| Republic of Costa Rica | 6.04 (1.14 to 18.77) | 2.49 (0.47 to 7.75) | 18.88 (3.61 to 57.81) | 2.98 (0.57 to 9.13) | 0.73 (0.44 to 1.01) | <0.001 | 311.79 (134.29 to 719.17) | 128.69 (55.43 to 296.84) | 949.41 (399.22 to 2202.09) | 149.98 (63.06 to 347.86) | 0.66 (0.42 to 0.9) | <0.001 |
| Republic of Croatia | 21.86 (4.17 to 67.99) | 2.9 (0.55 to 9.01) | 22.68 (4.37 to 70.08) | 3.12 (0.6 to 9.64) | 0.27 (0.04 to 0.51) | 0.026 | 1136.8 (498.34 to 2635.28) | 150.61 (66.02 to 349.14) | 1168.51 (511.98 to 2652.67) | 160.76 (70.43 to 364.94) | 0.3 (0.1 to 0.51) | 0.005 |
| Republic of Cuba | 29.44 (5.55 to 90.74) | 2.34 (0.44 to 7.2) | 58.06 (11.21 to 178.83) | 2.86 (0.55 to 8.82) | 0.14 (-0.05 to 0.34) | 0.142 | 1500.69 (639.29 to 3498.18) | 119.13 (50.75 to 277.69) | 2900.53 (1198.44 to 6776.15) | 143 (59.08 to 334.07) | 0.09 (-0.11 to 0.29) | 0.351 |
| Republic of Cyprus | 2.5 (0.46 to 7.81) | 2.64 (0.48 to 8.23) | 5.32 (1.03 to 16.35) | 2.57 (0.5 to 7.9) | 0.1 (0 to 0.2) | 0.041 | 119.54 (47.49 to 283.94) | 126 (50.06 to 299.27) | 263.54 (107.02 to 620.86) | 127.33 (51.71 to 299.97) | 0.21 (0.13 to 0.29) | <0.001 |
| Population of Côte d ivoire | 17.14 (3.24 to 55.34) | 2.09 (0.39 to 6.74) | 47.56 (8.28 to 148.45) | 2.01 (0.35 to 6.26) | -0.21 (-0.4 to -0.03) | 0.026 | 829.45 (326.33 to 2076.25) | 101.06 (39.76 to 252.97) | 2279.89 (863.75 to 5713.06) | 96.18 (36.44 to 241.01) | -0.19 (-0.32 to -0.06) | 0.006 |
| Republic of Djibouti | 0.7 (0.13 to 2.22) | 2.31 (0.42 to 7.36) | 3.94 (0.65 to 12.21) | 2.73 (0.45 to 8.46) | 0.63 (0.56 to 0.69) | <0.001 | 35.47 (14.32 to 86.7) | 117.84 (47.56 to 288.01) | 190.31 (74.73 to 461.65) | 131.79 (51.75 to 319.68) | 0.45 (0.4 to 0.5) | <0.001 |
| Republic of Ecuador | 15.87 (2.99 to 49.54) | 2.04 (0.38 to 6.36) | 45.7 (8.19 to 143.86) | 2.2 (0.39 to 6.92) | 0.37 (0.28 to 0.46) | <0.001 | 808.68 (344.14 to 1904.11) | 103.76 (44.16 to 244.32) | 2296.34 (932.94 to 5510.1) | 110.49 (44.89 to 265.12) | 0.33 (0.25 to 0.4) | <0.001 |
| Republic of El Salvador | 10.2 (1.86 to 31.6) | 2.65 (0.48 to 8.22) | 17.19 (3.23 to 54) | 2.77 (0.52 to 8.69) | 0.06 (0.02 to 0.1) | 0.009 | 510.65 (213.85 to 1198.7) | 132.78 (55.6 to 311.69) | 856.47 (352.69 to 2039.42) | 137.86 (56.77 to 328.28) | 0.07 (0.05 to 0.1) | <0.001 |
| Republic of Equatorial Guinea | 0.93 (0.17 to 2.96) | 3.3 (0.61 to 10.56) | 2.17 (0.37 to 6.89) | 2.39 (0.41 to 7.59) | -1.1 (-1.18 to -1.01) | <0.001 | 45.26 (17.9 to 113.06) | 161.21 (63.74 to 402.66) | 108.87 (42.8 to 264.77) | 119.91 (47.14 to 291.62) | -0.97 (-1.04 to -0.91) | <0.001 |
| Republic of Estonia | 5.55 (1.05 to 17.47) | 2.59 (0.49 to 8.15) | 6.19 (1.21 to 18.74) | 2.97 (0.58 to 8.97) | 0.36 (0.22 to 0.5) | <0.001 | 289.3 (125.34 to 675.68) | 135.06 (58.52 to 315.44) | 316.24 (135.56 to 710.22) | 151.41 (64.9 to 340.04) | 0.36 (0.25 to 0.48) | <0.001 |
| Republic of Fiji | 1.61 (0.29 to 5.12) | 2.44 (0.44 to 7.79) | 3.43 (0.61 to 10.61) | 2.83 (0.5 to 8.75) | 0.58 (0.45 to 0.72) | <0.001 | 81.95 (33.63 to 197.69) | 124.54 (51.1 to 300.43) | 174.02 (70.75 to 404.16) | 143.4 (58.3 to 333.04) | 0.57 (0.46 to 0.69) | <0.001 |
| Republic of Finland | 19.62 (3.79 to 60.82) | 2.55 (0.49 to 7.91) | 27.06 (5.26 to 82.78) | 3.1 (0.6 to 9.49) | 1.15 (0.95 to 1.35) | <0.001 | 959.51 (393.58 to 2281.18) | 124.85 (51.21 to 296.83) | 1284.57 (527.37 to 3032.75) | 147.25 (60.45 to 347.65) | 0.95 (0.79 to 1.12) | <0.001 |
| Republic of Ghana | 19.71 (3.55 to 63.58) | 1.91 (0.34 to 6.15) | 53.55 (10.04 to 164.04) | 1.97 (0.37 to 6.04) | 0.2 (0.06 to 0.33) | 0.006 | 998.12 (399.58 to 2380.86) | 96.51 (38.64 to 230.22) | 2569.02 (989.34 to 6091.52) | 94.6 (36.43 to 224.32) | 0.04 (-0.07 to 0.15) | 0.485 |
| Republic of Guatemala | 14.62 (2.76 to 45.15) | 2.65 (0.5 to 8.17) | 37.41 (7.14 to 116.65) | 2.6 (0.5 to 8.1) | 0.12 (0.05 to 0.2) | 0.001 | 738.97 (310.7 to 1735.51) | 133.71 (56.22 to 314.01) | 1891.41 (782.61 to 4491.52) | 131.31 (54.33 to 311.81) | 0.11 (0.05 to 0.17) | 0.001 |
| Republic of Guinea | 10.63 (1.85 to 32.53) | 2.39 (0.42 to 7.31) | 20.45 (3.59 to 65.44) | 2.4 (0.42 to 7.66) | 0.17 (0.09 to 0.25) | <0.001 | 524.2 (204.49 to 1214.95) | 117.85 (45.97 to 273.15) | 972.44 (385.19 to 2458.6) | 113.88 (45.11 to 287.91) | 0.03 (-0.03 to 0.09) | 0.361 |
| Republic of Guinea-Bissau | 1.5 (0.27 to 4.88) | 2.59 (0.47 to 8.45) | 2.98 (0.55 to 9.24) | 2.33 (0.43 to 7.23) | -0.19 (-0.28 to -0.1) | <0.001 | 68.56 (26.04 to 176.26) | 118.73 (45.1 to 305.27) | 136.63 (50.04 to 338.08) | 106.85 (39.13 to 264.39) | -0.2 (-0.28 to -0.13) | <0.001 |
| Republic of Guyana | 1.13 (0.21 to 3.56) | 1.96 (0.36 to 6.19) | 2.37 (0.45 to 7.51) | 2.55 (0.48 to 8.1) | 1.05 (0.81 to 1.29) | <0.001 | 58.57 (24.98 to 136.63) | 101.89 (43.45 to 237.7) | 116.96 (47.01 to 280.12) | 126.14 (50.7 to 302.1) | 0.9 (0.69 to 1.11) | <0.001 |
| Republic of Haiti | 14.08 (2.64 to 42.56) | 2.83 (0.53 to 8.56) | 29.28 (5.12 to 94.72) | 2.46 (0.43 to 7.95) | -0.28 (-0.42 to -0.13) | <0.001 | 677.07 (277.93 to 1591.44) | 136.22 (55.92 to 320.19) | 1415.54 (574.37 to 3508.32) | 118.85 (48.23 to 294.57) | -0.27 (-0.39 to -0.16) | <0.001 |
| Republic of Honduras | 9.48 (1.74 to 29.26) | 3.12 (0.57 to 9.64) | 31.28 (5.96 to 95.55) | 3.47 (0.66 to 10.59) | 0.53 (0.46 to 0.59) | <0.001 | 450.99 (181.13 to 1089.36) | 148.63 (59.69 to 359.01) | 1427.55 (544.27 to 3493.19) | 158.18 (60.31 to 387.05) | 0.37 (0.31 to 0.43) | <0.001 |
| Republic of Iceland | 0.74 (0.14 to 2.24) | 2.48 (0.47 to 7.48) | 1.47 (0.29 to 4.39) | 2.69 (0.52 to 8.05) | 0.7 (0.42 to 0.98) | <0.001 | 38.32 (16.06 to 86.82) | 127.62 (53.5 to 289.17) | 73.84 (31.68 to 166.85) | 135.35 (58.06 to 305.85) | 0.58 (0.35 to 0.81) | <0.001 |
| Republic of India | 1492.87 (281.72 to 4841.33) | 1.83 (0.35 to 5.94) | 3691.13 (682.8 to 11914.56) | 2.14 (0.4 to 6.92) | 0.47 (0.35 to 0.59) | <0.001 | 76623.31 (33474.66 to 185799.03) | 94.03 (41.08 to 228.02) | 180975.02 (76146.81 to 440864.65) | 105.05 (44.2 to 255.9) | 0.3 (0.18 to 0.41) | <0.001 |
| Republic of Indonesia | 408.85 (73.83 to 1301.8) | 2.56 (0.46 to 8.14) | 1195.79 (212.44 to 3641.56) | 2.98 (0.53 to 9.06) | 0.39 (0.21 to 0.57) | <0.001 | 22946.96 (10470.75 to 51812.92) | 143.54 (65.5 to 324.1) | 63273.7 (27611.16 to 143105.11) | 157.48 (68.72 to 356.16) | 0.25 (0.09 to 0.41) | 0.003 |
| Republic of Iraq | 33.37 (5.85 to 105.06) | 2.75 (0.48 to 8.65) | 109.77 (20.37 to 353.3) | 2.41 (0.45 to 7.76) | -0.52 (-0.61 to -0.44) | <0.001 | 1686.74 (688.35 to 3999.34) | 138.88 (56.68 to 329.29) | 5622.16 (2287.59 to 13425.45) | 123.44 (50.23 to 294.77) | -0.47 (-0.54 to -0.39) | <0.001 |
| Republic of Italy | 273.89 (52.47 to 859.9) | 3.19 (0.61 to 10.01) | 330.74 (63.98 to 1022.16) | 3.01 (0.58 to 9.29) | -0.37 (-0.43 to -0.3) | <0.001 | 13732.99 (6089.19 to 32423.45) | 159.9 (70.9 to 377.52) | 16484.59 (7125.71 to 38092.54) | 149.85 (64.78 to 346.28) | -0.37 (-0.43 to -0.31) | <0.001 |
| Republic of Kazakhstan | 39.97 (7.53 to 124.65) | 2.47 (0.47 to 7.71) | 60.22 (11.05 to 189.36) | 2.48 (0.45 to 7.79) | -0.4 (-0.84 to 0.04) | 0.072 | 2110.78 (910.03 to 4893.6) | 130.59 (56.3 to 302.75) | 3163.62 (1347.19 to 7254.58) | 130.15 (55.42 to 298.45) | -0.23 (-0.61 to 0.15) | 0.219 |
| Republic of Kenya | 29.2 (5.36 to 88.07) | 2.26 (0.41 to 6.82) | 102.14 (17.83 to 308.67) | 2.5 (0.44 to 7.56) | 0.47 (0.4 to 0.54) | <0.001 | 1586.6 (704.78 to 3521.56) | 122.83 (54.56 to 272.62) | 5140.18 (2220.2 to 11731.78) | 125.96 (54.41 to 287.5) | 0.2 (0.15 to 0.25) | <0.001 |
| Republic of Kiribati | 0.16 (0.03 to 0.5) | 2.76 (0.54 to 8.8) | 0.37 (0.07 to 1.13) | 3.13 (0.57 to 9.64) | 0.41 (0.07 to 0.75) | 0.019 | 7.65 (3.14 to 18.7) | 134.15 (55.02 to 327.66) | 17.66 (7.13 to 42.21) | 151.05 (60.96 to 360.96) | 0.42 (0.13 to 0.7) | 0.005 |
| Republic of Korea | 120.24 (22.87 to 374.92) | 2.54 (0.48 to 7.91) | 375.84 (73.38 to 1129.51) | 3.54 (0.69 to 10.63) | 0.94 (0.66 to 1.21) | <0.001 | 6063.44 (2421.05 to 14321.8) | 127.88 (51.06 to 302.05) | 19212.59 (8171.63 to 43431.4) | 180.79 (76.9 to 408.69) | 0.96 (0.72 to 1.21) | <0.001 |
| Republic of Latvia | 9.6 (1.84 to 29.49) | 2.6 (0.5 to 7.98) | 9.16 (1.77 to 27.34) | 3.03 (0.59 to 9.06) | 0.17 (0.01 to 0.33) | 0.033 | 500.2 (215.39 to 1148.34) | 135.45 (58.33 to 310.96) | 469.9 (201.63 to 1039.92) | 155.66 (66.79 to 344.48) | 0.24 (0.09 to 0.38) | 0.002 |
| Republic of Liberia | 3.66 (0.7 to 11.98) | 2.23 (0.43 to 7.3) | 7.84 (1.37 to 25.36) | 1.66 (0.29 to 5.35) | -0.95 (-1.21 to -0.68) | <0.001 | 179.18 (72.69 to 445.63) | 109.1 (44.26 to 271.34) | 388.61 (151.55 to 975.77) | 82.06 (32 to 206.05) | -0.89 (-1.08 to -0.7) | <0.001 |
| Republic of Lithuania | 12.67 (2.4 to 39.19) | 2.66 (0.5 to 8.21) | 14.33 (2.7 to 43.58) | 3.21 (0.6 to 9.75) | 0.11 (-0.16 to 0.37) | 0.417 | 655.45 (285.08 to 1494.14) | 137.35 (59.74 to 313.1) | 724.52 (305.64 to 1622.18) | 162.11 (68.39 to 362.96) | 0.19 (-0.06 to 0.43) | 0.131 |
| Republic of Madagascar | 16.91 (3.15 to 52.04) | 2.23 (0.42 to 6.87) | 42.5 (7.97 to 133.35) | 1.97 (0.37 to 6.18) | -0.24 (-0.55 to 0.07) | 0.13 | 910.99 (384.98 to 2012.35) | 120.33 (50.85 to 265.82) | 2280.5 (959.88 to 5256.39) | 105.69 (44.49 to 243.62) | -0.23 (-0.5 to 0.03) | 0.084 |
| Republic of Malawi | 14.59 (2.58 to 46.94) | 2.43 (0.43 to 7.83) | 30.9 (5.69 to 97.48) | 2.42 (0.45 to 7.64) | 0.1 (-0.04 to 0.24) | 0.145 | 741.58 (311.36 to 1786.1) | 123.63 (51.91 to 297.77) | 1505.34 (591.47 to 3673.89) | 117.99 (46.36 to 287.96) | -0.07 (-0.18 to 0.05) | 0.262 |
| Republic of Maldives | 0.57 (0.11 to 1.81) | 3.43 (0.65 to 11) | 1.76 (0.33 to 5.37) | 2.11 (0.4 to 6.42) | -2 (-2.2 to -1.81) | <0.001 | 29.56 (13.02 to 69.4) | 179.13 (78.88 to 420.55) | 97.09 (42.66 to 218.3) | 116.15 (51.03 to 261.16) | -1.71 (-1.88 to -1.54) | <0.001 |
| Republic of Mali | 16.33 (3.18 to 50.53) | 2.62 (0.51 to 8.1) | 37.55 (6.88 to 115.35) | 2.49 (0.46 to 7.66) | -0.24 (-0.35 to -0.12) | <0.001 | 773.88 (302.13 to 1855.06) | 124.03 (48.42 to 297.31) | 1771.47 (688.67 to 4273.78) | 117.57 (45.71 to 283.64) | -0.25 (-0.33 to -0.16) | <0.001 |
| Republic of Malta | 1.09 (0.21 to 3.32) | 2.15 (0.4 to 6.54) | 2.04 (0.4 to 6.31) | 2.79 (0.54 to 8.64) | 1.38 (1.12 to 1.64) | <0.001 | 55.27 (22.69 to 125.46) | 108.75 (44.64 to 246.86) | 98.25 (39.46 to 230.58) | 134.5 (54.02 to 315.65) | 1.16 (0.94 to 1.39) | <0.001 |
| Republic of Mauritius | 3.03 (0.59 to 9.51) | 2.93 (0.57 to 9.19) | 7.77 (1.54 to 23.67) | 3.52 (0.7 to 10.71) | 1.22 (0.75 to 1.7) | <0.001 | 155.54 (64.33 to 358.65) | 150.29 (62.16 to 346.54) | 397.92 (174.47 to 914.64) | 180.06 (78.95 to 413.87) | 1.18 (0.78 to 1.58) | <0.001 |
| Republic of Moldova | 13.55 (2.6 to 42.12) | 2.7 (0.52 to 8.39) | 17.55 (3.36 to 54.32) | 2.92 (0.56 to 9.05) | 0.44 (0.07 to 0.82) | 0.022 | 697.93 (303.18 to 1617.48) | 139.05 (60.4 to 322.25) | 886.12 (382.46 to 2066.88) | 147.64 (63.72 to 344.36) | 0.45 (0.13 to 0.78) | 0.007 |
| Republic of Mozambique | 24.66 (4.7 to 78.41) | 2.59 (0.49 to 8.24) | 50.57 (9.47 to 156.69) | 2.69 (0.5 to 8.34) | 0.3 (0.23 to 0.37) | <0.001 | 1239.15 (527.54 to 2939.22) | 130.25 (55.45 to 308.94) | 2381.65 (935.97 to 5874.99) | 126.7 (49.79 to 312.55) | 0.04 (-0.02 to 0.1) | 0.174 |
| Republic of Namibia | 2.35 (0.44 to 7.44) | 2.49 (0.47 to 7.88) | 5.69 (1 to 18.08) | 2.64 (0.47 to 8.4) | 0.19 (0.14 to 0.24) | <0.001 | 118.77 (49.55 to 283.24) | 125.93 (52.53 to 300.31) | 272.02 (105.49 to 683.1) | 126.47 (49.05 to 317.6) | -0.01 (-0.04 to 0.03) | 0.767 |
| Republic of Nauru | 0.03 (0 to 0.08) | 2.92 (0.54 to 9.16) | 0.03 (0 to 0.08) | 2.68 (0.48 to 8.52) | -0.17 (-0.28 to -0.06) | 0.005 | 1.27 (0.49 to 3.11) | 139.86 (54.25 to 342.77) | 1.26 (0.5 to 3.11) | 130.33 (51.27 to 322) | -0.15 (-0.24 to -0.05) | 0.005 |
| Republic of Nicaragua | 5.54 (1.06 to 17.26) | 2.41 (0.46 to 7.52) | 16.43 (2.92 to 49.11) | 2.42 (0.43 to 7.23) | 0.02 (-0.08 to 0.13) | 0.651 | 287.86 (123.37 to 667.97) | 125.37 (53.73 to 290.91) | 851.01 (360.2 to 1951.6) | 125.24 (53.01 to 287.22) | 0.05 (-0.04 to 0.14) | 0.295 |
| Republic of Niue | 0.01 (0 to 0.03) | 3.81 (0.72 to 11.56) | 0.01 (0 to 0.03) | 4.04 (0.77 to 12.37) | -0.01 (-0.16 to 0.14) | 0.925 | 0.42 (0.16 to 1.02) | 174.57 (68.04 to 424.26) | 0.51 (0.21 to 1.23) | 188.44 (76.08 to 453.84) | 0.09 (-0.04 to 0.22) | 0.154 |
| Republic of Palau | 0.04 (0.01 to 0.14) | 3.02 (0.57 to 9.62) | 0.12 (0.02 to 0.39) | 3.02 (0.55 to 9.75) | 0.57 (0.07 to 1.07) | 0.028 | 2.09 (0.82 to 5.14) | 142.8 (56.04 to 351.29) | 5.88 (2.35 to 14.35) | 148.36 (59.38 to 362.09) | 0.65 (0.23 to 1.06) | 0.003 |
| Republic of Panama | 5.27 (1 to 16.18) | 2.45 (0.47 to 7.53) | 14.69 (2.76 to 45.81) | 2.68 (0.5 to 8.37) | 0.26 (0.2 to 0.31) | <0.001 | 271.22 (115.43 to 628.41) | 126.27 (53.74 to 292.56) | 754.48 (320.33 to 1737.33) | 137.77 (58.49 to 317.24) | 0.24 (0.19 to 0.3) | <0.001 |
| Republic of Paraguay | 10.22 (1.98 to 31.75) | 3.25 (0.63 to 10.1) | 28.51 (5.21 to 89.51) | 3.51 (0.64 to 11.03) | 0.47 (0.33 to 0.61) | <0.001 | 513.42 (215.24 to 1189.4) | 163.33 (68.47 to 378.37) | 1399.18 (574.04 to 3326.47) | 172.38 (70.72 to 409.82) | 0.39 (0.27 to 0.52) | <0.001 |
| Republic of Peru | 38.69 (7.36 to 118.05) | 2.21 (0.42 to 6.73) | 97.74 (18.94 to 298.15) | 2.22 (0.43 to 6.77) | 0.04 (-0.04 to 0.12) | 0.338 | 1924.71 (791.22 to 4531.76) | 109.76 (45.12 to 258.44) | 4877.13 (2032.13 to 11428.08) | 110.7 (46.12 to 259.38) | 0.04 (-0.02 to 0.1) | 0.223 |
| Republic of Poland | 146.12 (27.67 to 465.44) | 2.97 (0.56 to 9.46) | 205.43 (39.03 to 637.8) | 3.18 (0.6 to 9.88) | 0.96 (0.54 to 1.38) | <0.001 | 7518.81 (3353.32 to 17398.49) | 152.84 (68.17 to 353.67) | 10359.43 (4541.02 to 23754.17) | 160.52 (70.36 to 368.07) | 0.86 (0.51 to 1.2) | <0.001 |
| Republic of Rwanda | 10.85 (2.05 to 34.97) | 2.83 (0.53 to 9.14) | 29.7 (5.3 to 92.84) | 2.99 (0.53 to 9.34) | 0.72 (0.32 to 1.13) | 0.001 | 528.68 (212.99 to 1292.95) | 138.15 (55.66 to 337.87) | 1389.79 (514.09 to 3342.61) | 139.8 (51.71 to 336.23) | 0.62 (0.27 to 0.97) | 0.001 |
| Republic of San Marino | 0.08 (0.02 to 0.26) | 2.34 (0.44 to 7.4) | 0.14 (0.02 to 0.42) | 2.4 (0.4 to 7.29) | -0.02 (-0.13 to 0.09) | 0.731 | 4.35 (1.89 to 9.87) | 125.56 (54.43 to 284.97) | 7.22 (2.98 to 15.91) | 125.48 (51.8 to 276.37) | -0.14 (-0.23 to -0.04) | 0.007 |
| Republic of Senegal | 11.86 (2.24 to 37.34) | 2.49 (0.47 to 7.84) | 33.1 (5.72 to 104.2) | 2.79 (0.48 to 8.78) | 0.6 (0.41 to 0.79) | <0.001 | 565.77 (222.81 to 1373.29) | 118.84 (46.8 to 288.45) | 1488.82 (560.63 to 3752.89) | 125.5 (47.26 to 316.35) | 0.36 (0.22 to 0.51) | <0.001 |
| Republic of Serbia | 44.59 (8.54 to 137.1) | 3.1 (0.59 to 9.54) | 45.53 (8.83 to 138.51) | 3.1 (0.6 to 9.42) | 0.24 (0.08 to 0.39) | 0.003 | 2245.14 (984.87 to 5226.37) | 156.27 (68.55 to 363.77) | 2303.42 (1004.74 to 5196.12) | 156.72 (68.36 to 353.54) | 0.25 (0.12 to 0.38) | 0.001 |
| Republic of Seychelles | 0.22 (0.04 to 0.71) | 3.51 (0.67 to 11.09) | 0.54 (0.1 to 1.63) | 2.96 (0.55 to 8.86) | -0.52 (-0.99 to -0.05) | 0.032 | 11.02 (4.55 to 26.62) | 172.55 (71.31 to 417) | 28.32 (12.03 to 64.42) | 154.16 (65.51 to 350.65) | -0.3 (-0.71 to 0.11) | 0.143 |
| Republic of Sierra Leone | 5.67 (1.05 to 18.22) | 1.92 (0.35 to 6.16) | 12.64 (2.27 to 39.16) | 1.91 (0.34 to 5.92) | 0.06 (-0.2 to 0.32) | 0.629 | 292.55 (123.52 to 687.08) | 98.89 (41.75 to 232.25) | 632.51 (248.7 to 1498.25) | 95.7 (37.63 to 226.68) | -0.07 (-0.26 to 0.12) | 0.457 |
| Republic of Singapore | 6.43 (1.21 to 19.97) | 1.83 (0.35 to 5.7) | 24.09 (4.65 to 73.31) | 2.05 (0.4 to 6.24) | 0.83 (0.58 to 1.08) | <0.001 | 334.36 (140.65 to 778.86) | 95.44 (40.15 to 222.32) | 1283.47 (566.32 to 2910.73) | 109.19 (48.18 to 247.62) | 0.81 (0.59 to 1.04) | <0.001 |
| Republic of Slovenia | 7.53 (1.41 to 23.38) | 2.69 (0.5 to 8.34) | 11.67 (2.25 to 35.16) | 3.04 (0.59 to 9.15) | 0.58 (0.38 to 0.77) | <0.001 | 384.91 (163.97 to 908.27) | 137.33 (58.5 to 324.05) | 599.17 (260.23 to 1336.73) | 155.87 (67.7 to 347.74) | 0.62 (0.45 to 0.79) | <0.001 |
| Republic of South Africa | 61.24 (11.45 to 192) | 2.16 (0.4 to 6.77) | 161.42 (30.12 to 511.53) | 2.48 (0.46 to 7.87) | 0.71 (0.61 to 0.82) | <0.001 | 3351.98 (1506 to 7625.37) | 118.24 (53.12 to 268.98) | 8322.7 (3581.55 to 19809.39) | 128.02 (55.09 to 304.7) | 0.51 (0.41 to 0.62) | <0.001 |
| Republic of South Sudan | 11.62 (2.14 to 35.74) | 2.84 (0.52 to 8.74) | 19.98 (3.36 to 61.09) | 2.61 (0.44 to 7.99) | -0.49 (-0.76 to -0.23) | 0.001 | 578.36 (235.71 to 1384.85) | 141.36 (57.61 to 338.47) | 983.36 (391.72 to 2352.54) | 128.69 (51.26 to 307.87) | -0.49 (-0.72 to -0.26) | <0.001 |
| Republic of Sudan | 40.81 (7.49 to 133.34) | 2.9 (0.53 to 9.47) | 90.31 (16.31 to 280.96) | 2.58 (0.47 to 8.02) | -0.57 (-0.64 to -0.5) | <0.001 | 2076.41 (877.45 to 5046.52) | 147.41 (62.29 to 358.27) | 4677.53 (1983.92 to 10803.33) | 133.54 (56.64 to 308.43) | -0.49 (-0.55 to -0.42) | <0.001 |
| Republic of Suriname | 0.93 (0.18 to 2.86) | 2.57 (0.5 to 7.94) | 2.29 (0.43 to 6.8) | 2.75 (0.52 to 8.19) | -0.22 (-0.65 to 0.22) | 0.315 | 47.59 (20.29 to 109.35) | 132.14 (56.34 to 303.62) | 114.45 (48.31 to 260.76) | 137.83 (58.17 to 314.02) | -0.22 (-0.61 to 0.18) | 0.272 |
| Republic of Tajikistan | 10.79 (2.04 to 33.23) | 2.95 (0.56 to 9.08) | 29.02 (5.34 to 93.4) | 2.89 (0.53 to 9.29) | -0.49 (-1.12 to 0.14) | 0.123 | 553.92 (240.18 to 1262.99) | 151.43 (65.66 to 345.28) | 1445.63 (597.18 to 3501.48) | 143.78 (59.4 to 348.26) | -0.45 (-1.01 to 0.12) | 0.118 |
| Republic of Trinidad and Tobago | 2.41 (0.46 to 7.52) | 2.17 (0.41 to 6.77) | 6.58 (1.21 to 21.02) | 2.93 (0.54 to 9.36) | 1.4 (1.17 to 1.64) | <0.001 | 124.21 (52.43 to 290.72) | 111.83 (47.2 to 261.75) | 322.9 (132.1 to 770.01) | 143.77 (58.82 to 342.85) | 1.18 (0.98 to 1.38) | <0.001 |
| Republic of Tunisia | 25.05 (4.61 to 73.63) | 3.57 (0.66 to 10.5) | 58.58 (10.95 to 184.77) | 3.32 (0.62 to 10.46) | -0.28 (-0.7 to 0.13) | 0.176 | 1279.24 (544.79 to 2907.62) | 182.45 (77.7 to 414.7) | 2970.18 (1267.8 to 6909.06) | 168.12 (71.76 to 391.08) | -0.21 (-0.58 to 0.16) | 0.246 |
| Republic of Turkey | 172.21 (32.07 to 523.79) | 3.26 (0.61 to 9.93) | 381.06 (73.53 to 1208.83) | 3.04 (0.59 to 9.63) | -0.11 (-0.43 to 0.22) | 0.517 | 9004.95 (3947.55 to 20365.07) | 170.65 (74.81 to 385.94) | 20022.08 (8651.63 to 45519.85) | 159.48 (68.91 to 362.58) | -0.08 (-0.35 to 0.18) | 0.518 |
| Republic of Uganda | 23.53 (4.41 to 73.4) | 2.42 (0.45 to 7.54) | 63.07 (11.45 to 193.73) | 2.55 (0.46 to 7.83) | -0.23 (-0.41 to -0.05) | 0.014 | 1206.19 (495.03 to 2821.15) | 123.88 (50.84 to 289.74) | 3067.71 (1194.52 to 7371.55) | 123.99 (48.28 to 297.95) | -0.33 (-0.49 to -0.17) | <0.001 |
| Republic of Uzbekistan | 40.68 (7.72 to 128.31) | 2.82 (0.53 to 8.89) | 105.09 (20.19 to 327.02) | 2.54 (0.49 to 7.9) | -0.48 (-1.1 to 0.14) | 0.123 | 2082.98 (881.97 to 4916.84) | 144.37 (61.13 to 340.79) | 5420.46 (2260.53 to 12429.14) | 130.92 (54.6 to 300.19) | -0.34 (-0.87 to 0.2) | 0.211 |
| Republic of Vanuatu | 0.32 (0.06 to 0.99) | 2.78 (0.5 to 8.62) | 0.85 (0.16 to 2.7) | 2.85 (0.53 to 9.02) | 0.25 (0.09 to 0.41) | 0.003 | 15.45 (6.13 to 37.08) | 134.9 (53.51 to 323.78) | 40.82 (16.04 to 101.58) | 136.55 (53.68 to 339.82) | 0.2 (0.09 to 0.31) | 0.001 |
| Republic of Yemen | 26.89 (4.81 to 84.75) | 3.21 (0.57 to 10.12) | 63.58 (11.17 to 201.05) | 2.51 (0.44 to 7.94) | -0.61 (-0.72 to -0.5) | <0.001 | 1342.44 (546.54 to 3209.41) | 160.28 (65.25 to 383.18) | 3225.47 (1377.56 to 7731.64) | 127.37 (54.4 to 305.32) | -0.57 (-0.67 to -0.48) | <0.001 |
| Republic of Zambia | 12.06 (2.26 to 37.81) | 2.57 (0.48 to 8.06) | 31.82 (5.62 to 98.85) | 2.29 (0.41 to 7.13) | -0.57 (-0.63 to -0.5) | <0.001 | 611.87 (258.4 to 1455.87) | 130.4 (55.07 to 310.28) | 1581.25 (633.57 to 3814.76) | 113.98 (45.67 to 274.98) | -0.61 (-0.66 to -0.56) | <0.001 |
| Republic of Zimbabwe | 17.14 (3.43 to 51.49) | 2.74 (0.55 to 8.24) | 27.38 (4.75 to 83.72) | 2.39 (0.42 to 7.32) | -0.36 (-0.51 to -0.21) | <0.001 | 851.81 (348.65 to 1974.54) | 136.33 (55.8 to 316.03) | 1327.42 (512.34 to 3203.77) | 115.99 (44.77 to 279.94) | -0.43 (-0.55 to -0.32) | <0.001 |
| Republic of the Congo | 5.22 (0.99 to 16.34) | 3.46 (0.65 to 10.82) | 16.47 (3 to 51.27) | 2.99 (0.54 to 9.32) | -0.67 (-0.89 to -0.45) | <0.001 | 249.85 (99.47 to 605.69) | 165.47 (65.88 to 401.15) | 802.4 (318.97 to 1945.46) | 145.83 (57.97 to 353.58) | -0.56 (-0.75 to -0.37) | <0.001 |
| Republic of the Gambia | 1.36 (0.25 to 4.28) | 2.07 (0.39 to 6.53) | 3.81 (0.7 to 11.85) | 2.31 (0.43 to 7.19) | -0.07 (-0.31 to 0.17) | 0.565 | 67.03 (26.9 to 162.02) | 102.28 (41.06 to 247.25) | 177.39 (65.48 to 442.39) | 107.66 (39.74 to 268.48) | -0.16 (-0.34 to 0.02) | 0.073 |
| Republic of the Marshall Islands | 0.08 (0.01 to 0.24) | 2.82 (0.52 to 8.88) | 0.2 (0.03 to 0.61) | 3.04 (0.53 to 9.51) | 1.02 (0.72 to 1.32) | <0.001 | 3.55 (1.4 to 8.81) | 131.11 (51.5 to 325.24) | 9.13 (3.53 to 22.73) | 141.55 (54.71 to 352.46) | 0.93 (0.68 to 1.17) | <0.001 |
| Republic of the Niger | 11.37 (2.18 to 35.63) | 2.19 (0.42 to 6.87) | 32.74 (5.64 to 102.3) | 2.49 (0.43 to 7.77) | 0.4 (0.15 to 0.65) | 0.003 | 555.57 (222.16 to 1328.94) | 107.13 (42.84 to 256.27) | 1550.42 (629.97 to 3819.07) | 117.72 (47.83 to 289.96) | 0.3 (0.1 to 0.5) | 0.005 |
| Republic of the Philippines | 122.85 (23.29 to 386.6) | 2.53 (0.48 to 7.97) | 386.27 (71.09 to 1235.6) | 2.97 (0.55 to 9.49) | 0.54 (0.41 to 0.68) | <0.001 | 6785.87 (3088.01 to 15558.26) | 139.92 (63.67 to 320.79) | 20143.43 (8759.76 to 47300.12) | 154.66 (67.26 to 363.17) | 0.36 (0.24 to 0.48) | <0.001 |
| Republic of the Union of Myanmar | 88.83 (16.74 to 278.25) | 2.69 (0.51 to 8.43) | 194.55 (36.14 to 590.96) | 2.89 (0.54 to 8.79) | 0.31 (0 to 0.62) | 0.05 | 4922.87 (2185.95 to 10797.94) | 149.22 (66.26 to 327.3) | 10142.24 (4418.22 to 23207.48) | 150.87 (65.72 to 345.22) | 0.15 (-0.12 to 0.42) | 0.272 |
| Romania | 98.39 (18.89 to 299.29) | 3 (0.58 to 9.11) | 93.05 (18.33 to 291.39) | 2.76 (0.54 to 8.63) | 0.03 (-0.14 to 0.21) | 0.691 | 5002.04 (2147.94 to 11486.14) | 152.32 (65.41 to 349.76) | 4769.7 (2068.88 to 10999.84) | 141.3 (61.29 to 325.86) | 0.04 (-0.1 to 0.18) | 0.587 |
| Russian Federation | 578.24 (109.23 to 1833.53) | 2.93 (0.55 to 9.28) | 711.14 (134.85 to 2231.46) | 3.11 (0.59 to 9.76) | 0.46 (-0.08 to 1.01) | 0.094 | 29925.82 (13323.86 to 69553.36) | 151.48 (67.44 to 352.06) | 36222.09 (15959.36 to 84654.6) | 158.48 (69.82 to 370.38) | 0.46 (0.01 to 0.9) | 0.043 |
| Saint Kitts and Nevis | 0.09 (0.02 to 0.27) | 3.03 (0.57 to 9.43) | 0.3 (0.06 to 0.96) | 2.87 (0.55 to 9.11) | 0.32 (-0.34 to 0.98) | 0.332 | 4.17 (1.72 to 9.8) | 144.58 (59.49 to 339.82) | 14.67 (5.91 to 35.9) | 138.74 (55.9 to 339.39) | 0.35 (-0.21 to 0.92) | 0.214 |
| Saint Lucia | 0.24 (0.05 to 0.74) | 2.5 (0.47 to 7.51) | 0.81 (0.16 to 2.53) | 2.67 (0.52 to 8.31) | 0.13 (-0.09 to 0.34) | 0.229 | 12.14 (4.97 to 28) | 124.02 (50.71 to 285.94) | 41.09 (16.63 to 94.64) | 134.83 (54.58 to 310.53) | 0.19 (0 to 0.38) | 0.054 |
| Saint Vincent and the Grenadines | 0.22 (0.04 to 0.7) | 2.91 (0.56 to 9.08) | 0.54 (0.1 to 1.66) | 2.94 (0.57 to 9.11) | 0.05 (-0.35 to 0.44) | 0.805 | 10.81 (4.43 to 25.87) | 141.04 (57.77 to 337.53) | 26.41 (11.02 to 62.6) | 144.8 (60.43 to 343.19) | 0.15 (-0.21 to 0.5) | 0.407 |
| Slovak Republic | 18.49 (3.52 to 57.29) | 2.87 (0.55 to 8.89) | 27.42 (5.23 to 84.9) | 2.86 (0.55 to 8.85) | 0.75 (0.42 to 1.09) | <0.001 | 924.19 (397.13 to 2155.24) | 143.34 (61.59 to 334.26) | 1391.32 (600.32 to 3225.89) | 144.95 (62.54 to 336.08) | 0.71 (0.43 to 0.99) | <0.001 |
| Socialist Republic of Viet Nam | 184.27 (33.06 to 580.49) | 4.1 (0.73 to 12.91) | 542.97 (100.59 to 1677.44) | 3.77 (0.7 to 11.65) | -0.29 (-0.89 to 0.32) | 0.341 | 8897.24 (3754.89 to 21553.3) | 197.81 (83.48 to 479.2) | 25665.8 (9880.99 to 61345.82) | 178.2 (68.61 to 425.94) | -0.28 (-0.83 to 0.27) | 0.305 |
| Solomon Islands | 0.78 (0.13 to 2.57) | 3.18 (0.55 to 10.49) | 1.78 (0.31 to 5.46) | 2.76 (0.48 to 8.47) | -0.69 (-0.89 to -0.49) | <0.001 | 37.83 (14.88 to 94.06) | 154.59 (60.83 to 384.4) | 87.05 (34.73 to 210.44) | 135.02 (53.86 to 326.38) | -0.63 (-0.77 to -0.49) | <0.001 |
| State of Eritrea | 5 (0.92 to 16.32) | 2.29 (0.42 to 7.49) | 12.55 (2.15 to 40.55) | 2.35 (0.4 to 7.6) | -0.26 (-0.44 to -0.08) | 0.005 | 255.32 (103.54 to 625.11) | 117.14 (47.5 to 286.8) | 604.77 (225.22 to 1509.89) | 113.37 (42.22 to 283.03) | -0.39 (-0.52 to -0.26) | <0.001 |
| State of Israel | 11.07 (2.13 to 34.12) | 2.2 (0.42 to 6.78) | 27.16 (5.28 to 83.17) | 2.25 (0.44 to 6.89) | 0.67 (0.44 to 0.9) | <0.001 | 566.72 (238.34 to 1304.32) | 112.56 (47.34 to 259.05) | 1360.75 (555.36 to 3193.39) | 112.78 (46.03 to 264.67) | 0.51 (0.32 to 0.71) | <0.001 |
| State of Kuwait | 3.85 (0.71 to 11.8) | 2.05 (0.38 to 6.27) | 15.4 (2.89 to 48.31) | 1.82 (0.34 to 5.72) | -0.87 (-1.22 to -0.52) | <0.001 | 208.96 (88.33 to 467.13) | 111.14 (46.98 to 248.45) | 865.79 (378.37 to 1945) | 102.43 (44.76 to 230.1) | -0.63 (-0.94 to -0.32) | <0.001 |
| State of Libya | 11.23 (2.02 to 34.63) | 3.41 (0.61 to 10.5) | 27.06 (4.85 to 84.39) | 2.56 (0.46 to 8) | -1.66 (-1.92 to -1.4) | <0.001 | 548.81 (221.59 to 1277.87) | 166.41 (67.19 to 387.48) | 1370.75 (562.67 to 3263.07) | 129.87 (53.31 to 309.15) | -1.42 (-1.64 to -1.19) | <0.001 |
| State of Qatar | 0.87 (0.15 to 2.71) | 1.48 (0.25 to 4.58) | 7.96 (1.38 to 24.41) | 1.33 (0.23 to 4.09) | -0.24 (-0.49 to 0.02) | 0.068 | 48.48 (19.96 to 110.47) | 81.96 (33.75 to 186.76) | 461.61 (196.32 to 1035.29) | 77.26 (32.86 to 173.28) | -0.06 (-0.28 to 0.15) | 0.541 |
| Sultanate of Oman | 3.69 (0.66 to 11.59) | 2.11 (0.38 to 6.64) | 12.24 (2.24 to 39.81) | 1.76 (0.32 to 5.72) | -0.48 (-0.59 to -0.37) | <0.001 | 189.32 (79.04 to 442.22) | 108.47 (45.29 to 253.39) | 653.59 (272.59 to 1548.62) | 93.87 (39.15 to 222.42) | -0.32 (-0.41 to -0.23) | <0.001 |
| Swiss Confederation | 22.88 (4.33 to 71.58) | 2.21 (0.42 to 6.93) | 39.4 (7.63 to 117.09) | 2.51 (0.49 to 7.45) | 0.35 (0.3 to 0.39) | <0.001 | 1160.14 (482.75 to 2708.82) | 112.26 (46.71 to 262.12) | 1952.46 (816.19 to 4424.05) | 124.26 (51.94 to 281.55) | 0.26 (0.21 to 0.31) | <0.001 |
| Syrian Arab Republic | 24.96 (4.67 to 80.08) | 3.01 (0.56 to 9.65) | 63.05 (10.86 to 202.76) | 2.96 (0.51 to 9.51) | -0.21 (-0.57 to 0.14) | 0.227 | 1304.73 (569.36 to 3041.73) | 157.28 (68.63 to 366.67) | 3271.65 (1446.34 to 7658.13) | 153.39 (67.81 to 359.05) | -0.19 (-0.49 to 0.12) | 0.216 |
| Taiwan (Province of China) | 62.69 (11.96 to 194.12) | 2.73 (0.52 to 8.47) | 130.64 (24.92 to 408.68) | 2.93 (0.56 to 9.16) | 0.57 (0.04 to 1.1) | 0.036 | 2968.09 (1203.01 to 7223.53) | 129.46 (52.47 to 315.07) | 6334.75 (2603.34 to 14994.27) | 141.92 (58.32 to 335.92) | 0.79 (0.32 to 1.26) | 0.002 |
| Togolese Republic | 4.08 (0.76 to 13.04) | 2.07 (0.38 to 6.62) | 14.79 (2.56 to 46.94) | 2.16 (0.37 to 6.86) | 0.01 (-0.18 to 0.2) | 0.917 | 200.27 (80.2 to 492.48) | 101.61 (40.69 to 249.87) | 699.34 (267.2 to 1754.23) | 102.24 (39.07 to 256.47) | -0.07 (-0.23 to 0.09) | 0.375 |
| Tokelau | 0.01 (0 to 0.03) | 6.38 (1.22 to 19.33) | 0.01 (0 to 0.02) | 3.77 (0.71 to 11.63) | -0.93 (-1.35 to -0.52) | <0.001 | 0.38 (0.15 to 0.94) | 271.19 (103.09 to 665.32) | 0.31 (0.12 to 0.75) | 175.64 (67.6 to 426.67) | -0.69 (-1.05 to -0.33) | 0.001 |
| Turkmenistan | 6.92 (1.31 to 21.28) | 2.71 (0.51 to 8.32) | 14.9 (2.73 to 47.98) | 2.38 (0.44 to 7.65) | -0.69 (-1.26 to -0.12) | 0.019 | 360.55 (151.84 to 819.68) | 141.02 (59.39 to 320.6) | 777.31 (331.86 to 1833.42) | 123.95 (52.92 to 292.36) | -0.55 (-1.04 to -0.06) | 0.03 |
| Tuvalu | 0.03 (0.01 to 0.1) | 4 (0.75 to 12.7) | 0.05 (0.01 to 0.16) | 3.52 (0.64 to 11.41) | -0.02 (-0.27 to 0.23) | 0.896 | 1.52 (0.6 to 3.8) | 184.13 (73.34 to 460.44) | 2.4 (0.95 to 5.99) | 166.71 (66.23 to 415.96) | 0.05 (-0.17 to 0.26) | 0.666 |
| Ukraine | 211.72 (39.8 to 662.78) | 2.9 (0.55 to 9.08) | 222.24 (37.6 to 710.96) | 3.12 (0.53 to 9.99) | 0.22 (-0.13 to 0.57) | 0.218 | 11185.75 (4959.79 to 25537.25) | 153.23 (67.94 to 349.83) | 11187.26 (4509.73 to 27086.48) | 157.17 (63.36 to 380.54) | 0.14 (-0.14 to 0.42) | 0.332 |
| Union of the Comoros | 0.84 (0.16 to 2.6) | 2.75 (0.53 to 8.48) | 2.2 (0.39 to 6.75) | 2.84 (0.51 to 8.68) | -0.27 (-0.42 to -0.11) | 0.001 | 41.66 (17.16 to 98.34) | 136.04 (56.05 to 321.12) | 105.58 (40.06 to 251.96) | 135.87 (51.55 to 324.23) | -0.33 (-0.46 to -0.2) | <0.001 |
| United Arab Emirates | 3.04 (0.53 to 9.72) | 1.29 (0.23 to 4.12) | 43.47 (7.8 to 134.48) | 1.35 (0.24 to 4.17) | 0.11 (-0.09 to 0.32) | 0.26 | 165.27 (66.66 to 394.29) | 69.96 (28.22 to 166.91) | 2433.14 (988.97 to 5677.79) | 75.47 (30.67 to 176.11) | 0.12 (-0.07 to 0.31) | 0.192 |
| United Kingdom of Great Britain and Northern Ireland | 188.05 (35.88 to 587.63) | 2.3 (0.44 to 7.19) | 253.28 (48.59 to 777.68) | 2.36 (0.45 to 7.24) | 0.14 (0.07 to 0.21) | <0.001 | 9450.68 (4121.36 to 22111.8) | 115.62 (50.42 to 270.51) | 12495.36 (5277.76 to 29551.99) | 116.26 (49.11 to 274.97) | 0.02 (-0.02 to 0.07) | 0.329 |
| United Mexican States | 136.23 (25.51 to 430.83) | 2.15 (0.4 to 6.8) | 384.11 (72.39 to 1212.32) | 2.3 (0.43 to 7.26) | 0.18 (0.09 to 0.27) | <0.001 | 7217.6 (3220.18 to 16647.8) | 113.92 (50.83 to 262.77) | 20143.04 (8962.59 to 46740.87) | 120.66 (53.69 to 279.99) | 0.15 (0.07 to 0.24) | 0.001 |
| United Republic of Tanzania | 46.42 (8.69 to 148.8) | 2.97 (0.55 to 9.51) | 112.96 (21.08 to 348.65) | 2.65 (0.49 to 8.17) | -0.43 (-0.5 to -0.37) | <0.001 | 2281.26 (925.35 to 5636.8) | 145.74 (59.12 to 360.1) | 5488.25 (2136.82 to 13137.92) | 128.69 (50.1 to 308.05) | -0.45 (-0.52 to -0.39) | <0.001 |
| United States Virgin Islands | 0.32 (0.06 to 0.99) | 2.35 (0.44 to 7.37) | 0.53 (0.1 to 1.66) | 3.55 (0.67 to 11.01) | 1.45 (1.31 to 1.59) | <0.001 | 15.74 (6.39 to 38.17) | 116.89 (47.44 to 283.42) | 25.46 (10.17 to 60.54) | 169.18 (67.59 to 402.3) | 1.24 (1.12 to 1.36) | <0.001 |
| United States of America | 1079.31 (204.94 to 3448.93) | 3.44 (0.65 to 10.99) | 2198.08 (420.81 to 6910.87) | 4.28 (0.82 to 13.46) | 1.17 (0.9 to 1.44) | <0.001 | 56062.06 (25508.74 to 130104.4) | 178.62 (81.27 to 414.53) | 111544.79 (50246.5 to 258173.59) | 217.28 (97.88 to 502.91) | 1.03 (0.81 to 1.24) | <0.001 |

Note: CI, confidence interval; AAPC, average annual percentage change; EOAD, early-onset Alzheimer’s disease and other dementias; DALYs, disability-adjusted life years.

**Supplementary Table S13. Age-standardized prevalence and incidence of EOAD in women aged 40-64 years and their AAPCs from 1990 to 2021 at the country levels.**

|  | Prevalence | | | | | | Incidence | | | | | |
| --- | --- | --- | --- | --- | --- | --- | --- | --- | --- | --- | --- | --- |
| Country | No of people with EOAD in 1990 (000s) | Age standardised rate in 1990 (per 100 000) | No of people with EOAD in 2021 (000s) | Age standardised rate in 2021 (per 100 000) | AAPC (95% CI) | *p* value | No of people with EOAD in 1990 (000s) | Age standardised rate in 1990 (per 100 000) | No of people with EOAD in 2021 (000s) | Age standardised rate in 2021 (per 100 000) | AAPC (95% CI) | *p* value |
| American Samoa | 11.71 (8.67 to 15.38) | 328.46 (243.2 to 431.24) | 25.76 (19.18 to 33.87) | 367.71 (273.8 to 483.58) | 0.47 (0.24 to 0.7) | <0.001 | 2.17 (1.38 to 3.13) | 60.83 (38.78 to 87.83) | 4.67 (2.99 to 6.77) | 66.64 (42.63 to 96.63) | 0.38 (0.19 to 0.57) | <0.001 |
| Antigua and Barbuda | 17.24 (12.64 to 22.65) | 295.76 (216.78 to 388.53) | 47.3 (34.56 to 62.33) | 310.42 (226.85 to 409.11) | 0.26 (0 to 0.53) | 0.054 | 3.02 (1.87 to 4.43) | 51.76 (32.04 to 76.05) | 8.2 (5.1 to 12.09) | 53.82 (33.5 to 79.34) | 0.21 (0 to 0.43) | 0.052 |
| Arab Republic of Egypt | 15408.36 (11561.87 to 20004.28) | 331.75 (248.93 to 430.7) | 34860.11 (26065.86 to 45494.39) | 329.74 (246.56 to 430.33) | 0.1 (-0.01 to 0.21) | 0.083 | 2931.62 (1887.69 to 4183.42) | 63.12 (40.64 to 90.07) | 6779.03 (4335.35 to 9663.19) | 64.12 (41.01 to 91.4) | 0.15 (0.06 to 0.24) | 0.002 |
| Argentine Republic | 14969.85 (11151.4 to 19409.44) | 369.04 (274.91 to 478.49) | 21760.12 (16062.71 to 28624.72) | 336.6 (248.47 to 442.79) | -0.15 (-0.21 to -0.09) | <0.001 | 2509.42 (1559.85 to 3630.93) | 61.86 (38.45 to 89.51) | 3636.93 (2262.03 to 5346.37) | 56.26 (34.99 to 82.7) | -0.2 (-0.25 to -0.15) | <0.001 |
| Australia | 8379.95 (6228.54 to 11008.36) | 381.7 (283.71 to 501.43) | 15830.69 (11689.44 to 20865.27) | 388.7 (287.02 to 512.31) | 0.38 (0.25 to 0.51) | <0.001 | 1446.15 (898.61 to 2102.89) | 65.87 (40.93 to 95.79) | 2699.61 (1664.03 to 3982.58) | 66.28 (40.86 to 97.79) | 0.29 (0.17 to 0.41) | <0.001 |
| Barbados | 90.41 (66.51 to 118.5) | 320.88 (236.04 to 420.59) | 196.27 (147.82 to 254.83) | 357.92 (269.57 to 464.71) | 0.58 (0.35 to 0.82) | <0.001 | 15.69 (9.84 to 23.06) | 55.68 (34.91 to 81.83) | 33.28 (20.97 to 48.06) | 60.69 (38.23 to 87.63) | 0.46 (0.27 to 0.65) | <0.001 |
| Belize | 34.21 (25.33 to 44.93) | 298.64 (221.07 to 392.19) | 132.75 (97 to 175.6) | 276.63 (202.12 to 365.93) | -0.18 (-0.42 to 0.07) | 0.156 | 6.08 (3.79 to 8.89) | 53.04 (33.12 to 77.56) | 23.78 (14.79 to 35.1) | 49.55 (30.81 to 73.14) | -0.17 (-0.37 to 0.04) | 0.103 |
| Bermuda | 26.53 (19.69 to 34.91) | 314.06 (233.06 to 413.15) | 48.14 (35.72 to 63.22) | 391.1 (290.15 to 513.61) | 0.88 (0.7 to 1.06) | <0.001 | 4.6 (2.85 to 6.8) | 54.46 (33.71 to 80.53) | 8.04 (5.11 to 11.82) | 65.33 (41.54 to 96.04) | 0.73 (0.59 to 0.88) | <0.001 |
| Bolivarian Republic of Venezuela | 5211.8 (3897.45 to 6782.75) | 346.65 (259.23 to 451.14) | 15849.68 (11794.22 to 20654.24) | 384.88 (286.4 to 501.55) | 0.62 (0.46 to 0.78) | <0.001 | 932.43 (602.13 to 1344.88) | 62.02 (40.05 to 89.45) | 2737.65 (1734.16 to 3960.53) | 66.48 (42.11 to 96.17) | 0.47 (0.35 to 0.6) | <0.001 |
| Bosnia and Herzegovina | 2469.31 (1854.17 to 3250.73) | 396.85 (297.99 to 522.43) | 2643.48 (1985.99 to 3408.74) | 433.69 (325.82 to 559.24) | 0.35 (0.13 to 0.58) | 0.003 | 427.26 (273.12 to 620.28) | 68.67 (43.89 to 99.69) | 452.65 (290.35 to 646.36) | 74.26 (47.63 to 106.04) | 0.31 (0.14 to 0.49) | 0.001 |
| Brunei Darussalam | 43.61 (31.56 to 57.84) | 253.59 (183.52 to 336.36) | 183.8 (135.93 to 243.57) | 298.88 (221.04 to 396.08) | 0.75 (0.48 to 1.03) | <0.001 | 8.25 (5.13 to 12.01) | 47.96 (29.82 to 69.85) | 33.05 (20.78 to 48.42) | 53.75 (33.8 to 78.73) | 0.56 (0.33 to 0.79) | <0.001 |
| Burkina Faso | 1885.83 (1392.13 to 2482) | 275.59 (203.44 to 362.71) | 3856.01 (2811.33 to 5150.04) | 245.62 (179.08 to 328.05) | -0.37 (-0.41 to -0.34) | <0.001 | 346.9 (217.56 to 501.97) | 50.7 (31.79 to 73.36) | 710.4 (440.33 to 1041.61) | 45.25 (28.05 to 66.35) | -0.36 (-0.39 to -0.34) | <0.001 |
| Canada | 19905.5 (15654.02 to 24803.77) | 541.66 (425.97 to 674.95) | 40849.31 (32320.98 to 50106.87) | 646.32 (511.38 to 792.79) | 0.62 (0.49 to 0.75) | <0.001 | 3294.2 (2251.56 to 4550.65) | 89.64 (61.27 to 123.83) | 6409.7 (4483.36 to 8641.9) | 101.41 (70.94 to 136.73) | 0.37 (0.25 to 0.5) | <0.001 |
| Central African Republic | 832.87 (611.65 to 1099.84) | 402.06 (295.27 to 530.93) | 1654.82 (1223.5 to 2170.62) | 367.72 (271.87 to 482.33) | -0.41 (-0.52 to -0.3) | <0.001 | 162.44 (102.29 to 236.54) | 78.42 (49.38 to 114.19) | 329.57 (205.65 to 476.87) | 73.23 (45.7 to 105.96) | -0.38 (-0.47 to -0.29) | <0.001 |
| Commonwealth of Dominica | 23.17 (17.11 to 30.54) | 331.61 (244.83 to 437.11) | 32.44 (23.96 to 42.61) | 335.82 (247.99 to 441.01) | -0.28 (-0.55 to -0.01) | 0.043 | 4.01 (2.49 to 5.82) | 57.36 (35.63 to 83.26) | 5.57 (3.52 to 8.19) | 57.69 (36.46 to 84.72) | -0.26 (-0.48 to -0.03) | 0.029 |
| Commonwealth of the Bahamas | 62.67 (45.82 to 83.58) | 262.24 (191.76 to 349.78) | 191.2 (141.04 to 252.06) | 302.74 (223.32 to 399.12) | 0.35 (0.17 to 0.54) | 0.001 | 11.28 (6.86 to 16.77) | 47.22 (28.69 to 70.2) | 33.45 (20.82 to 48.96) | 52.96 (32.96 to 77.52) | 0.29 (0.13 to 0.44) | 0.001 |
| Cook Islands | 6.23 (4.66 to 8.18) | 362.48 (270.8 to 475.81) | 12.59 (9.42 to 16.44) | 430.09 (321.97 to 561.73) | 0.26 (0.09 to 0.43) | 0.004 | 1.14 (0.72 to 1.65) | 66.06 (42.04 to 95.86) | 2.22 (1.42 to 3.19) | 75.79 (48.52 to 109.14) | 0.2 (0.06 to 0.34) | 0.008 |
| Czech Republic | 6143.25 (4584.85 to 8015.66) | 386.28 (288.29 to 504.02) | 7057.51 (5233.47 to 9298.1) | 381.71 (283.06 to 502.89) | 0.42 (0.16 to 0.67) | 0.002 | 1072.02 (687.99 to 1534.86) | 67.41 (43.26 to 96.51) | 1232.3 (784.34 to 1790.26) | 66.65 (42.42 to 96.83) | 0.31 (0.1 to 0.53) | 0.005 |
| Democratic People's Republic of Korea | 9307.48 (6849.22 to 12337.31) | 335.59 (246.95 to 444.83) | 15564.32 (11494.39 to 20488.32) | 349.44 (258.07 to 460) | -0.54 (-0.82 to -0.27) | <0.001 | 1725.36 (1097.45 to 2491.55) | 62.21 (39.57 to 89.83) | 2854.63 (1817.94 to 4094.17) | 64.09 (40.82 to 91.92) | -0.48 (-0.71 to -0.24) | <0.001 |
| Democratic Republic of Sao Tome and Principe | 23.55 (17.39 to 31.08) | 279.6 (206.49 to 368.93) | 43.49 (31.41 to 58.19) | 220.55 (159.3 to 295.1) | -0.86 (-1.07 to -0.66) | <0.001 | 4.2 (2.64 to 6.11) | 49.82 (31.31 to 72.55) | 7.97 (4.84 to 11.76) | 40.43 (24.53 to 59.62) | -0.75 (-0.91 to -0.58) | <0.001 |
| Democratic Republic of Timor-Leste | 193.08 (142.79 to 254.71) | 347.75 (257.18 to 458.76) | 439.19 (329.02 to 576.14) | 393.07 (294.48 to 515.65) | 0.84 (0.57 to 1.12) | <0.001 | 36.97 (23.78 to 53.11) | 66.58 (42.83 to 95.65) | 81.24 (52.54 to 116.52) | 72.71 (47.02 to 104.28) | 0.65 (0.43 to 0.88) | <0.001 |
| Democratic Republic of the Congo | 11311.62 (8391.81 to 14931.27) | 411.36 (305.18 to 542.99) | 25037.53 (18608.82 to 32937.94) | 369.58 (274.68 to 486.19) | -0.36 (-0.51 to -0.21) | <0.001 | 2182.54 (1380.1 to 3160.14) | 79.37 (50.19 to 114.92) | 4835.05 (3075.92 to 6980.17) | 71.37 (45.4 to 103.03) | -0.36 (-0.49 to -0.23) | <0.001 |
| Democratic Socialist Republic of Sri Lanka | 6376.57 (4736.7 to 8393.6) | 377.53 (280.44 to 496.95) | 15367.27 (11583.6 to 20056.81) | 441.58 (332.85 to 576.33) | 0.77 (0.66 to 0.88) | <0.001 | 1165.83 (749.67 to 1669.54) | 69.02 (44.39 to 98.85) | 2703.16 (1756.37 to 3881.11) | 77.67 (50.47 to 111.52) | 0.6 (0.5 to 0.69) | <0.001 |
| Dominican Republic | 1564.6 (1155.42 to 2067.06) | 281.37 (207.79 to 371.73) | 3888.75 (2873.96 to 5174.41) | 290.64 (214.8 to 386.73) | 0.07 (0 to 0.15) | 0.053 | 278.52 (173.8 to 407.42) | 50.09 (31.26 to 73.27) | 693.35 (432.49 to 1010.67) | 51.82 (32.32 to 75.54) | 0.03 (-0.03 to 0.09) | 0.352 |
| Eastern Republic of Uruguay | 1667.7 (1254.32 to 2179.74) | 393.36 (295.86 to 514.14) | 1946.65 (1450.89 to 2548.73) | 365.39 (272.33 to 478.4) | -0.2 (-0.28 to -0.11) | <0.001 | 276.32 (175.17 to 398.45) | 65.18 (41.32 to 93.98) | 320.74 (200.48 to 468.88) | 60.2 (37.63 to 88.01) | -0.23 (-0.3 to -0.16) | <0.001 |
| Federal Democratic Republic of Ethiopia | 11387.44 (8363.03 to 15181.15) | 357.41 (262.49 to 476.49) | 23506.81 (17190.73 to 31319.95) | 314.91 (230.3 to 419.58) | -0.2 (-0.28 to -0.12) | <0.001 | 2040.53 (1295.91 to 2942.73) | 64.05 (40.67 to 92.36) | 4256.75 (2671.25 to 6170.82) | 57.03 (35.79 to 82.67) | -0.19 (-0.26 to -0.13) | <0.001 |
| Federal Democratic Republic of Nepal | 4030.14 (2946.18 to 5371.95) | 268.52 (196.29 to 357.92) | 9127.45 (6671.93 to 12042.74) | 257.13 (187.95 to 339.25) | -0.12 (-0.14 to -0.1) | <0.001 | 737.87 (461.16 to 1078.7) | 49.16 (30.73 to 71.87) | 1639.55 (1024.03 to 2389.47) | 46.19 (28.85 to 67.31) | -0.21 (-0.23 to -0.19) | <0.001 |
| Federal Republic of Germany | 54710.83 (40947.17 to 71388.16) | 431.46 (322.91 to 562.98) | 68852.46 (51683.49 to 89469.32) | 468.16 (351.42 to 608.34) | 0.07 (-0.16 to 0.31) | 0.547 | 9070.83 (5646.57 to 13240.24) | 71.53 (44.53 to 104.41) | 11346.12 (7144.04 to 16409.27) | 77.15 (48.58 to 111.57) | 0.09 (-0.11 to 0.3) | 0.35 |
| Federal Republic of Nigeria | 13939.43 (10141.23 to 18548.2) | 256.4 (186.53 to 341.17) | 43351.26 (31476.83 to 57778.34) | 239.86 (174.16 to 319.69) | -0.28 (-0.45 to -0.12) | 0.001 | 2432.54 (1527.15 to 3545.24) | 44.74 (28.09 to 65.21) | 7414.71 (4593.84 to 10829.57) | 41.03 (25.42 to 59.92) | -0.33 (-0.46 to -0.19) | <0.001 |
| Federal Republic of Somalia | 1452.8 (1071.42 to 1922.26) | 281.45 (207.57 to 372.4) | 3711.17 (2747.96 to 4897.41) | 273.65 (202.63 to 361.13) | 0.45 (0.19 to 0.71) | 0.001 | 281.75 (178.82 to 408.06) | 54.58 (34.64 to 79.05) | 716.37 (454.7 to 1045.54) | 52.82 (33.53 to 77.1) | 0.35 (0.13 to 0.57) | 0.003 |
| Federated States of Micronesia | 22.79 (16.95 to 30.05) | 352.1 (261.94 to 464.39) | 48.43 (36.21 to 63.42) | 396.19 (296.23 to 518.81) | 0.72 (0.44 to 0.99) | <0.001 | 4.22 (2.71 to 6.11) | 65.19 (41.9 to 94.38) | 8.87 (5.72 to 12.76) | 72.53 (46.79 to 104.41) | 0.62 (0.39 to 0.85) | <0.001 |
| Federative Republic of Brazil | 60679.61 (45418.86 to 78879.32) | 439.92 (329.28 to 571.87) | 160149.26 (120599.12 to 208634.7) | 471.02 (354.7 to 613.63) | 0.34 (0.26 to 0.42) | <0.001 | 10977.41 (7160.18 to 15482) | 79.59 (51.91 to 112.24) | 29047.01 (18865.35 to 41240.6) | 85.43 (55.49 to 121.3) | 0.33 (0.24 to 0.42) | <0.001 |
| French Republic | 24294.29 (17860.07 to 32069.7) | 298.17 (219.2 to 393.6) | 32728.53 (23646.12 to 43706.42) | 303.2 (219.06 to 404.91) | 0.3 (0.14 to 0.46) | 0.001 | 3933.88 (2366.84 to 5825.05) | 48.28 (29.05 to 71.49) | 5267.73 (3142.58 to 7937.32) | 48.8 (29.11 to 73.53) | 0.23 (0.09 to 0.37) | 0.003 |
| Gabonese Republic | 351.79 (261.43 to 456.06) | 465.64 (346.04 to 603.65) | 673.46 (494.75 to 880.66) | 377.02 (276.98 to 493.02) | -0.82 (-1.07 to -0.58) | <0.001 | 66.74 (42.22 to 95.51) | 88.34 (55.89 to 126.42) | 129.4 (82.06 to 187.61) | 72.44 (45.94 to 105.03) | -0.77 (-0.98 to -0.55) | <0.001 |
| Georgia | 3512.11 (2639.86 to 4598.62) | 432.67 (325.22 to 566.53) | 2736.12 (2061.93 to 3595.41) | 444.85 (335.24 to 584.56) | 0.05 (-0.28 to 0.38) | 0.758 | 598.43 (383.72 to 858.11) | 73.72 (47.27 to 105.71) | 465.62 (298.73 to 669.5) | 75.7 (48.57 to 108.85) | 0.06 (-0.22 to 0.33) | 0.673 |
| Grand Duchy of Luxembourg | 165.95 (120.94 to 219.11) | 285.98 (208.41 to 377.58) | 286.16 (206.42 to 381.97) | 259.54 (187.22 to 346.45) | -0.33 (-0.5 to -0.16) | <0.001 | 27.66 (16.89 to 41.38) | 47.67 (29.1 to 71.31) | 46.73 (28.25 to 69.56) | 42.39 (25.62 to 63.09) | -0.41 (-0.56 to -0.25) | <0.001 |
| Greenland | 23.26 (17.54 to 30.4) | 437.36 (329.81 to 571.52) | 48.8 (37.27 to 63.48) | 567.75 (433.61 to 738.47) | 0.66 (0.26 to 1.07) | 0.002 | 4.07 (2.66 to 5.86) | 76.45 (49.95 to 110.25) | 8.07 (5.28 to 11.53) | 93.92 (61.41 to 134.17) | 0.54 (0.22 to 0.87) | 0.002 |
| Grenada | 24.9 (18.67 to 32.45) | 350.82 (263.12 to 457.22) | 47.2 (34.98 to 62.7) | 314.15 (232.86 to 417.33) | -0.25 (-0.39 to -0.11) | 0.001 | 4.28 (2.72 to 6.28) | 60.28 (38.31 to 88.55) | 8.23 (5.16 to 12.03) | 54.78 (34.36 to 80.08) | -0.22 (-0.34 to -0.11) | <0.001 |
| Guam | 41.42 (30.62 to 54.82) | 340.14 (251.49 to 450.15) | 98.66 (73.89 to 129.38) | 413.49 (309.71 to 542.26) | 0.72 (0.54 to 0.89) | <0.001 | 7.55 (4.8 to 10.92) | 62.02 (39.45 to 89.68) | 17.38 (11.19 to 25.07) | 72.83 (46.89 to 105.06) | 0.57 (0.43 to 0.71) | <0.001 |
| Hashemite Kingdom of Jordan | 769.55 (568.08 to 1014.58) | 339.07 (250.3 to 447.03) | 4531.72 (3366.25 to 5945.31) | 358.8 (266.52 to 470.72) | -0.15 (-0.29 to -0.01) | 0.032 | 148.5 (93.59 to 214.87) | 65.43 (41.24 to 94.67) | 868.41 (558.19 to 1253.03) | 68.76 (44.2 to 99.21) | -0.11 (-0.23 to 0.01) | 0.064 |
| Hellenic Republic | 6315.85 (4659.71 to 8281.6) | 387.26 (285.71 to 507.79) | 6739.62 (4925.95 to 8911.58) | 356.69 (260.7 to 471.64) | -0.36 (-0.44 to -0.28) | <0.001 | 1036.87 (636.45 to 1538.52) | 63.58 (39.02 to 94.34) | 1106.83 (682.19 to 1650.39) | 58.58 (36.1 to 87.35) | -0.36 (-0.42 to -0.29) | <0.001 |
| Hungary | 6778.25 (5069.97 to 8842.51) | 402.17 (300.82 to 524.65) | 6795.54 (5101.82 to 8864.69) | 386.33 (290.04 to 503.96) | 0.34 (0.17 to 0.52) | <0.001 | 1186.11 (764.7 to 1712.38) | 70.38 (45.37 to 101.6) | 1193.02 (770.21 to 1709.93) | 67.82 (43.79 to 97.21) | 0.27 (0.12 to 0.42) | 0.001 |
| Independent State of Papua New Guinea | 1047.05 (778.01 to 1373.62) | 349.88 (259.98 to 459.01) | 2809.79 (2072.24 to 3734.21) | 298.75 (220.33 to 397.04) | -0.5 (-0.63 to -0.38) | <0.001 | 194.67 (124.76 to 278.98) | 65.05 (41.69 to 93.22) | 531.01 (337.62 to 768.63) | 56.46 (35.9 to 81.72) | -0.47 (-0.57 to -0.36) | <0.001 |
| Independent State of Samoa | 42.91 (32.06 to 56.47) | 359.8 (268.81 to 473.52) | 71.3 (53.02 to 93.96) | 354.19 (263.4 to 466.74) | -0.11 (-0.28 to 0.07) | 0.216 | 7.8 (4.97 to 11.23) | 65.39 (41.69 to 94.21) | 12.95 (8.28 to 18.6) | 64.32 (41.13 to 92.41) | -0.11 (-0.24 to 0.03) | 0.121 |
| Ireland | 1334.49 (961.79 to 1777.91) | 317.89 (229.11 to 423.52) | 2512.26 (1793.47 to 3374.38) | 304.47 (217.36 to 408.96) | -0.01 (-0.06 to 0.05) | 0.76 | 223.01 (133.45 to 332.22) | 53.12 (31.79 to 79.14) | 413.7 (244.3 to 620.1) | 50.14 (29.61 to 75.15) | -0.08 (-0.13 to -0.03) | 0.002 |
| Islamic Republic of Afghanistan | 3951.04 (2957.6 to 5164.91) | 395 (295.68 to 516.35) | 5421.54 (3904 to 7291.71) | 254.96 (183.59 to 342.91) | -2.18 (-2.69 to -1.67) | <0.001 | 753.57 (486.76 to 1087.71) | 75.34 (48.66 to 108.74) | 1095.7 (679.11 to 1589.23) | 51.53 (31.94 to 74.74) | -1.85 (-2.28 to -1.42) | <0.001 |
| Islamic Republic of Iran | 16438.79 (12271.57 to 21561.46) | 422.2 (315.17 to 553.77) | 47774.69 (35587.33 to 62784.02) | 395.36 (294.5 to 519.57) | 0 (-0.21 to 0.21) | 0.992 | 3004.72 (1941.73 to 4312.51) | 77.17 (49.87 to 110.76) | 8760.02 (5630.15 to 12502.13) | 72.49 (46.59 to 103.46) | -0.02 (-0.2 to 0.16) | 0.802 |
| Islamic Republic of Mauritania | 355.34 (258.02 to 473.8) | 251.25 (182.44 to 335.01) | 843.57 (615.82 to 1121.29) | 243.58 (177.82 to 323.77) | -0.05 (-0.19 to 0.09) | 0.465 | 65.92 (40.49 to 96.75) | 46.61 (28.63 to 68.41) | 155.55 (95.41 to 226.29) | 44.92 (27.55 to 65.34) | -0.08 (-0.2 to 0.03) | 0.149 |
| Islamic Republic of Pakistan | 19304.07 (13950.95 to 25677.53) | 243.19 (175.75 to 323.48) | 46733.35 (33427.92 to 62881.66) | 224.2 (160.37 to 301.68) | -0.29 (-0.31 to -0.26) | <0.001 | 3427.59 (2151.78 to 4982.41) | 43.18 (27.11 to 62.77) | 8269.68 (5156.66 to 12103.4) | 39.67 (24.74 to 58.07) | -0.3 (-0.32 to -0.28) | <0.001 |
| Jamaica | 691.21 (513.93 to 907.09) | 359.17 (267.05 to 471.34) | 1339.97 (993.68 to 1760.77) | 352.07 (261.08 to 462.63) | -0.11 (-0.32 to 0.1) | 0.309 | 120.71 (76.25 to 175.64) | 62.72 (39.62 to 91.27) | 231.98 (145.23 to 335.56) | 60.95 (38.16 to 88.17) | -0.14 (-0.31 to 0.03) | 0.102 |
| Japan | 87669.15 (65693.24 to 114534.61) | 404.45 (303.07 to 528.39) | 91962.25 (68620.75 to 120020.3) | 429.96 (320.83 to 561.15) | 0.29 (0.06 to 0.51) | 0.013 | 14747.77 (9472.28 to 20996.23) | 68.04 (43.7 to 96.86) | 15724.07 (10095.95 to 22613.04) | 73.52 (47.2 to 105.73) | 0.4 (0.21 to 0.58) | <0.001 |
| Kingdom of Bahrain | 100.38 (74.36 to 132.55) | 351.08 (260.08 to 463.61) | 533.22 (394.38 to 705.65) | 343.2 (253.84 to 454.18) | -0.05 (-0.52 to 0.44) | 0.848 | 19.22 (12.04 to 27.7) | 67.22 (42.11 to 96.89) | 101.95 (64.07 to 147.86) | 65.62 (41.24 to 95.16) | -0.07 (-0.47 to 0.34) | 0.736 |
| Kingdom of Belgium | 6192.13 (4618.69 to 8055.87) | 415.31 (309.78 to 540.32) | 7436.2 (5493.53 to 9812.91) | 391.43 (289.17 to 516.54) | -0.07 (-0.25 to 0.1) | 0.413 | 1009.76 (624.31 to 1460.58) | 67.73 (41.87 to 97.96) | 1208.24 (740.93 to 1784.91) | 63.6 (39 to 93.96) | -0.12 (-0.27 to 0.04) | 0.137 |
| Kingdom of Bhutan | 99.16 (71.67 to 131.74) | 253.61 (183.3 to 336.92) | 199.97 (145.03 to 266.64) | 243.62 (176.69 to 324.84) | -0.07 (-0.12 to -0.02) | 0.004 | 17.84 (11.08 to 26.28) | 45.63 (28.34 to 67.21) | 35.75 (21.92 to 52.61) | 43.56 (26.7 to 64.09) | -0.1 (-0.13 to -0.06) | <0.001 |
| Kingdom of Cambodia | 3051.94 (2279.74 to 3984.44) | 379.68 (283.61 to 495.69) | 8398.01 (6330.65 to 10988.27) | 418.2 (315.25 to 547.19) | 0.44 (0.26 to 0.62) | <0.001 | 566.54 (364.09 to 817.33) | 70.48 (45.3 to 101.68) | 1527.66 (986.28 to 2160.29) | 76.07 (49.11 to 107.58) | 0.34 (0.19 to 0.49) | <0.001 |
| Kingdom of Denmark | 2169.92 (1581.11 to 2877.98) | 279.85 (203.91 to 371.17) | 2702.35 (1938.55 to 3621.37) | 285.76 (204.99 to 382.94) | 0.08 (-0.02 to 0.17) | 0.112 | 364.87 (216.82 to 542.19) | 47.06 (27.96 to 69.93) | 423.59 (247.81 to 643.19) | 44.79 (26.2 to 68.01) | -0.16 (-0.28 to -0.05) | 0.005 |
| Kingdom of Eswatini | 148 (108.51 to 195.8) | 286.91 (210.35 to 379.58) | 311 (230.87 to 410.8) | 305.92 (227.1 to 404.09) | 0.31 (0.26 to 0.35) | <0.001 | 28.1 (17.46 to 40.86) | 54.48 (33.85 to 79.21) | 57.49 (36.27 to 83.89) | 56.55 (35.67 to 82.52) | 0.22 (0.19 to 0.26) | <0.001 |
| Kingdom of Lesotho | 492.51 (363.53 to 644.12) | 338.27 (249.68 to 442.4) | 617.44 (459.68 to 809.98) | 346.68 (258.1 to 454.79) | 0.29 (0.21 to 0.36) | <0.001 | 91.81 (57.57 to 132.33) | 63.06 (39.54 to 90.89) | 113.68 (71.37 to 163.76) | 63.83 (40.07 to 91.95) | 0.24 (0.17 to 0.3) | <0.001 |
| Kingdom of Morocco | 7850.66 (5832.67 to 10303.6) | 381.16 (283.18 to 500.25) | 18948.38 (14270.69 to 24667.47) | 379.09 (285.51 to 493.51) | -0.01 (-0.19 to 0.18) | 0.942 | 1504.51 (964.08 to 2170.56) | 73.05 (46.81 to 105.38) | 3575.1 (2295.68 to 5166.67) | 71.53 (45.93 to 103.37) | -0.06 (-0.23 to 0.1) | 0.435 |
| Kingdom of Norway | 2103.19 (1553.28 to 2774.12) | 368.97 (272.5 to 486.67) | 3071.17 (2226.22 to 4085.91) | 360.32 (261.19 to 479.37) | 0.06 (-0.03 to 0.15) | 0.164 | 349.22 (218.58 to 512.06) | 61.27 (38.35 to 89.83) | 493.23 (302.85 to 732.03) | 57.87 (35.53 to 85.88) | -0.08 (-0.15 to 0) | 0.061 |
| Kingdom of Saudi Arabia | 2518.63 (1847.31 to 3310.7) | 302.92 (222.18 to 398.18) | 10419.43 (7568.85 to 14079.3) | 256.67 (186.45 to 346.83) | -0.69 (-0.76 to -0.62) | <0.001 | 495.94 (311.17 to 719.8) | 59.65 (37.43 to 86.57) | 2079.16 (1287.41 to 3063.12) | 51.22 (31.71 to 75.46) | -0.61 (-0.67 to -0.56) | <0.001 |
| Kingdom of Spain | 21106.51 (15779 to 27324.37) | 379.61 (283.79 to 491.44) | 29001.16 (21281.52 to 38283.14) | 330.79 (242.74 to 436.66) | -0.47 (-0.58 to -0.35) | <0.001 | 3518.83 (2271.51 to 4991.56) | 63.29 (40.85 to 89.78) | 4670.95 (2821.38 to 6905.41) | 53.28 (32.18 to 78.76) | -0.48 (-0.58 to -0.38) | <0.001 |
| Kingdom of Sweden | 4747.97 (3465.46 to 6278.76) | 370.53 (270.45 to 490) | 6361.05 (4673.44 to 8442.8) | 405.95 (298.25 to 538.81) | 0.51 (0.4 to 0.61) | <0.001 | 788.08 (487.78 to 1145.75) | 61.5 (38.07 to 89.42) | 1013.7 (631.49 to 1483.06) | 64.69 (40.3 to 94.65) | 0.44 (0.33 to 0.55) | <0.001 |
| Kingdom of Thailand | 23015.66 (17386.62 to 29830.89) | 390.92 (295.31 to 506.67) | 60315.94 (45514.37 to 78654.73) | 444.76 (335.61 to 579.98) | 0.71 (0.51 to 0.91) | <0.001 | 4119.05 (2694.53 to 5828) | 69.96 (45.77 to 98.99) | 10654.84 (6942.88 to 15128.06) | 78.57 (51.2 to 111.55) | 0.71 (0.54 to 0.87) | <0.001 |
| Kingdom of Tonga | 30.64 (22.79 to 40.2) | 368.43 (274.06 to 483.33) | 39.14 (29.1 to 51.34) | 352.49 (262.01 to 462.32) | -0.31 (-0.38 to -0.25) | <0.001 | 5.59 (3.57 to 8.04) | 67.22 (42.92 to 96.62) | 7.14 (4.56 to 10.23) | 64.29 (41.06 to 92.1) | -0.3 (-0.35 to -0.24) | <0.001 |
| Kingdom of the Netherlands | 6872.18 (5159.2 to 8957.14) | 325.06 (244.03 to 423.68) | 11103.09 (8118.73 to 14626.7) | 382.88 (279.97 to 504.39) | 0.7 (0.55 to 0.85) | <0.001 | 1096.77 (676.67 to 1602.43) | 51.88 (32.01 to 75.8) | 1829.36 (1129.15 to 2690.69) | 63.08 (38.94 to 92.79) | 0.88 (0.69 to 1.08) | <0.001 |
| Kyrgyz Republic | 1611.92 (1208 to 2097.3) | 407.58 (305.45 to 530.31) | 2940.1 (2192.78 to 3835.17) | 366.54 (273.37 to 478.13) | -0.29 (-0.84 to 0.26) | 0.284 | 276.45 (175.84 to 397.12) | 69.9 (44.46 to 100.41) | 515.47 (326.69 to 742.32) | 64.26 (40.73 to 92.55) | -0.21 (-0.65 to 0.23) | 0.346 |
| Lao People's Democratic Republic | 1230.5 (920.31 to 1619.43) | 377.95 (282.67 to 497.4) | 2891.66 (2163.04 to 3797.1) | 372.33 (278.51 to 488.91) | -0.13 (-0.27 to 0.02) | 0.077 | 228.3 (148.71 to 328.13) | 70.12 (45.68 to 100.78) | 539.65 (346.28 to 766.61) | 69.48 (44.59 to 98.71) | -0.09 (-0.21 to 0.03) | 0.141 |
| Lebanese Republic | 1356.52 (1013.07 to 1765.9) | 428.32 (319.87 to 557.58) | 3176.19 (2382.53 to 4145.66) | 422.75 (317.12 to 551.79) | -0.12 (-0.18 to -0.06) | <0.001 | 255.93 (163.63 to 368.46) | 80.81 (51.67 to 116.34) | 598.27 (382.8 to 861.42) | 79.63 (50.95 to 114.66) | -0.11 (-0.15 to -0.06) | <0.001 |
| Malaysia | 6102.78 (4511.93 to 7995.46) | 411.6 (304.31 to 539.26) | 17258.1 (12892.57 to 22455.34) | 446.35 (333.44 to 580.77) | 0.59 (0.42 to 0.76) | <0.001 | 1143.09 (734.59 to 1637.25) | 77.1 (49.54 to 110.42) | 3112.28 (1981.28 to 4467.13) | 80.49 (51.24 to 115.53) | 0.47 (0.33 to 0.61) | <0.001 |
| Mongolia | 467.28 (344.77 to 617.78) | 324.49 (239.42 to 429) | 1503.18 (1113.72 to 1968.19) | 338.62 (250.88 to 443.37) | -0.11 (-0.49 to 0.27) | 0.556 | 84.09 (52.47 to 122.06) | 58.39 (36.43 to 84.76) | 268.93 (167.24 to 385.56) | 60.58 (37.67 to 86.85) | -0.06 (-0.37 to 0.25) | 0.692 |
| Montenegro | 340.51 (255 to 443.77) | 401.15 (300.41 to 522.8) | 435.96 (329.29 to 567.1) | 412.77 (311.77 to 536.94) | 0.19 (0.04 to 0.34) | 0.012 | 59.54 (38.11 to 85.68) | 70.14 (44.89 to 100.94) | 75.52 (48.52 to 108.36) | 71.51 (45.94 to 102.59) | 0.14 (0.03 to 0.25) | 0.016 |
| New Zealand | 1888.45 (1409.75 to 2462.58) | 440.21 (328.62 to 574.04) | 3816.04 (2833.74 to 5014.41) | 473.98 (351.97 to 622.83) | 0.37 (0.25 to 0.49) | <0.001 | 313.36 (195.8 to 453.39) | 73.05 (45.64 to 105.69) | 615.61 (388.47 to 902.63) | 76.46 (48.25 to 112.11) | 0.26 (0.15 to 0.37) | <0.001 |
| North Macedonia | 1001.21 (746.8 to 1309.55) | 374.52 (279.35 to 489.86) | 1502.98 (1133.25 to 1955.97) | 396.35 (298.85 to 515.81) | 0.29 (0.17 to 0.42) | <0.001 | 176.76 (112.31 to 256.33) | 66.12 (42.01 to 95.89) | 262.97 (168.4 to 376.2) | 69.35 (44.41 to 99.21) | 0.24 (0.14 to 0.35) | <0.001 |
| Northern Mariana Islands | 7.42 (5.37 to 10.01) | 235.7 (170.73 to 317.93) | 31.06 (23.14 to 41) | 390.1 (290.63 to 514.92) | 1.96 (1.47 to 2.44) | <0.001 | 1.46 (0.9 to 2.14) | 46.41 (28.74 to 67.98) | 5.59 (3.6 to 8.08) | 70.22 (45.15 to 101.43) | 1.56 (1.18 to 1.95) | <0.001 |
| Palestine | 538.88 (400.86 to 703.16) | 403.23 (299.96 to 526.16) | 1572.84 (1174.66 to 2056.54) | 352.79 (263.48 to 461.29) | -0.71 (-0.86 to -0.56) | <0.001 | 101.52 (64.8 to 146.54) | 75.97 (48.49 to 109.65) | 298.85 (190.55 to 431.23) | 67.03 (42.74 to 96.73) | -0.65 (-0.78 to -0.52) | <0.001 |
| People's Democratic Republic of Algeria | 7113.61 (5330.35 to 9366.33) | 402.81 (301.83 to 530.37) | 19980.31 (14821.19 to 26195.17) | 363.91 (269.95 to 477.11) | -0.36 (-0.55 to -0.17) | <0.001 | 1342.23 (852.95 to 1943.98) | 76 (48.3 to 110.08) | 3790.9 (2416.8 to 5455.64) | 69.05 (44.02 to 99.37) | -0.35 (-0.51 to -0.18) | <0.001 |
| People's Republic of Bangladesh | 16180.36 (11658.6 to 21408.96) | 243.39 (175.37 to 322.04) | 48444.54 (35212.92 to 64433.62) | 244.88 (177.99 to 325.7) | 0 (-0.1 to 0.1) | 0.972 | 2946.61 (1817.35 to 4304.06) | 44.32 (27.34 to 64.74) | 8726.86 (5413.3 to 12807.44) | 44.11 (27.36 to 64.74) | -0.03 (-0.12 to 0.05) | 0.403 |
| People's Republic of China | 491163.58 (367912.18 to 642065.07) | 421.51 (315.74 to 551.01) | 1292588.98 (973360.43 to 1696466.6) | 516.98 (389.31 to 678.52) | 0.67 (0.47 to 0.87) | <0.001 | 87858.32 (57013.07 to 124651.24) | 75.4 (48.93 to 106.97) | 244108.07 (158482.16 to 346587.84) | 97.63 (63.39 to 138.62) | 0.72 (0.57 to 0.88) | <0.001 |
| Plurinational State of Bolivia | 1280.13 (937.31 to 1700.31) | 252.2 (184.66 to 334.98) | 3559.29 (2584.64 to 4734.58) | 265.41 (192.73 to 353.06) | 0.34 (0.27 to 0.41) | <0.001 | 222.78 (136.7 to 327.88) | 43.89 (26.93 to 64.6) | 613.2 (379.06 to 902.06) | 45.73 (28.27 to 67.27) | 0.28 (0.22 to 0.34) | <0.001 |
| Portuguese Republic | 5571.34 (4126 to 7308.63) | 364.17 (269.7 to 477.73) | 7220.91 (5274.96 to 9538.54) | 354.99 (259.32 to 468.93) | -0.05 (-0.11 to 0.02) | 0.165 | 914.25 (562.06 to 1355.79) | 59.76 (36.74 to 88.62) | 1185.51 (726.31 to 1763.53) | 58.28 (35.71 to 86.7) | -0.06 (-0.11 to 0) | 0.063 |
| Principality of Andorra | 22.23 (16.15 to 29.53) | 325.43 (236.51 to 432.34) | 55.79 (40 to 74.5) | 321.3 (230.37 to 429.02) | -0.11 (-0.27 to 0.05) | 0.171 | 3.75 (2.25 to 5.55) | 54.83 (32.91 to 81.23) | 9.19 (5.58 to 13.64) | 52.94 (32.13 to 78.53) | -0.17 (-0.3 to -0.04) | 0.012 |
| Principality of Monaco | 19.73 (14.5 to 25.94) | 361.68 (265.76 to 475.39) | 26.18 (19.17 to 34.62) | 370.55 (271.38 to 490.02) | -0.02 (-0.11 to 0.07) | 0.676 | 3.3 (2.01 to 4.86) | 60.41 (36.79 to 89.1) | 4.25 (2.58 to 6.31) | 60.1 (36.57 to 89.32) | -0.09 (-0.17 to -0.01) | 0.038 |
| Puerto Rico | 1414.67 (1046.78 to 1864.16) | 307.58 (227.59 to 405.31) | 2157.95 (1618.22 to 2813.43) | 373.05 (279.75 to 486.37) | 0.71 (0.68 to 0.75) | <0.001 | 246.91 (154.92 to 363.24) | 53.68 (33.68 to 78.98) | 363.32 (227.96 to 528.57) | 62.81 (39.41 to 91.38) | 0.58 (0.55 to 0.61) | <0.001 |
| Republic of Albania | 1012.09 (751.95 to 1319.69) | 361.16 (268.33 to 470.93) | 2074.7 (1567.72 to 2684.71) | 462.8 (349.71 to 598.88) | 0.81 (0.6 to 1.01) | <0.001 | 176.92 (113.59 to 254.13) | 63.13 (40.53 to 90.68) | 350.67 (225.16 to 502.23) | 78.22 (50.23 to 112.03) | 0.7 (0.53 to 0.87) | <0.001 |
| Republic of Angola | 2535.55 (1877.3 to 3340.08) | 370.37 (274.22 to 487.89) | 8518.31 (6296.54 to 11219.52) | 351.78 (260.03 to 463.33) | -0.12 (-0.22 to -0.01) | 0.041 | 495.66 (309.67 to 718.88) | 72.4 (45.23 to 105.01) | 1653.6 (1050.21 to 2408.17) | 68.29 (43.37 to 99.45) | -0.15 (-0.24 to -0.05) | 0.004 |
| Republic of Armenia | 1712.48 (1296.72 to 2226.71) | 434.17 (328.76 to 564.54) | 2263.99 (1705.58 to 2952.12) | 452.71 (341.05 to 590.31) | 0.34 (-0.2 to 0.88) | 0.207 | 290.89 (188.02 to 421.18) | 73.75 (47.67 to 106.78) | 380.68 (246.33 to 549.91) | 76.12 (49.26 to 109.96) | 0.29 (-0.15 to 0.72) | 0.186 |
| Republic of Austria | 4125.32 (3049.18 to 5452.19) | 358.08 (264.67 to 473.26) | 5675.2 (4128.83 to 7511.61) | 358.8 (261.03 to 474.9) | -0.11 (-0.25 to 0.03) | 0.11 | 688.4 (428.23 to 1022.27) | 59.75 (37.17 to 88.73) | 925.21 (569.73 to 1374.74) | 58.49 (36.02 to 86.91) | -0.15 (-0.27 to -0.04) | 0.012 |
| Republic of Azerbaijan | 2946.51 (2206.76 to 3844.08) | 408.58 (306.01 to 533.05) | 6651.01 (4967.85 to 8706.46) | 399.37 (298.3 to 522.79) | -0.43 (-1.1 to 0.25) | 0.206 | 503.24 (317.27 to 726.14) | 69.78 (44 to 100.69) | 1138.05 (728.38 to 1668.03) | 68.34 (43.74 to 100.16) | -0.35 (-0.88 to 0.19) | 0.197 |
| Republic of Belarus | 7290.76 (5461.53 to 9574.39) | 447.16 (334.97 to 587.22) | 8040.41 (6057.14 to 10496.72) | 450.47 (339.36 to 588.09) | 0.12 (-0.27 to 0.52) | 0.531 | 1246.4 (793.98 to 1783.58) | 76.44 (48.7 to 109.39) | 1378.57 (880.55 to 1993.94) | 77.24 (49.33 to 111.71) | 0.12 (-0.21 to 0.45) | 0.454 |
| Republic of Benin | 803.23 (591.6 to 1070.61) | 267.15 (196.76 to 356.07) | 2192.92 (1606.39 to 2892.18) | 234.07 (171.47 to 308.71) | -0.44 (-0.47 to -0.4) | <0.001 | 147.98 (91.56 to 215.5) | 49.22 (30.45 to 71.67) | 404.51 (250.37 to 587.67) | 43.18 (26.72 to 62.73) | -0.42 (-0.45 to -0.39) | <0.001 |
| Republic of Botswana | 313.45 (231.52 to 416.34) | 344.45 (254.41 to 457.51) | 838.31 (618.18 to 1103.16) | 310.96 (229.31 to 409.21) | -0.15 (-0.31 to 0.01) | 0.066 | 58.38 (36.74 to 85.04) | 64.15 (40.38 to 93.45) | 156.5 (99.06 to 225.92) | 58.05 (36.75 to 83.8) | -0.17 (-0.3 to -0.03) | 0.017 |
| Republic of Bulgaria | 6090.82 (4555.71 to 7935.64) | 421.94 (315.6 to 549.74) | 4969.76 (3702.87 to 6509.37) | 408.18 (304.13 to 534.64) | 0.12 (0 to 0.24) | 0.045 | 1065.62 (689.98 to 1533.02) | 73.82 (47.8 to 106.2) | 867.43 (555.1 to 1252.32) | 71.24 (45.59 to 102.86) | 0.06 (-0.04 to 0.16) | 0.212 |
| Republic of Burundi | 1228.71 (912.34 to 1622.58) | 335.46 (249.09 to 443) | 2520.72 (1859.29 to 3313.24) | 304.28 (224.44 to 399.94) | -0.1 (-0.31 to 0.11) | 0.322 | 232.66 (148.67 to 337.92) | 63.52 (40.59 to 92.26) | 476.7 (297.57 to 686.47) | 57.54 (35.92 to 82.86) | -0.12 (-0.3 to 0.06) | 0.183 |
| Republic of Cabo Verde | 91.74 (67.84 to 121.06) | 343.32 (253.88 to 453.02) | 194.57 (143.15 to 255.47) | 298.85 (219.88 to 392.4) | -0.69 (-1.22 to -0.16) | 0.012 | 15.95 (10.05 to 23.34) | 59.68 (37.6 to 87.34) | 33.95 (21.17 to 49.64) | 52.14 (32.52 to 76.24) | -0.63 (-1.07 to -0.2) | 0.006 |
| Republic of Cameroon | 1905.39 (1384.87 to 2512.6) | 260.34 (189.22 to 343.3) | 5148.08 (3753.78 to 6853.99) | 225.34 (164.31 to 300) | -0.49 (-0.58 to -0.4) | <0.001 | 350.64 (218.83 to 510.78) | 47.91 (29.9 to 69.79) | 957.06 (588.6 to 1407.96) | 41.89 (25.76 to 61.63) | -0.46 (-0.53 to -0.39) | <0.001 |
| Republic of Chad | 1085.75 (797.52 to 1434.82) | 265.45 (194.98 to 350.79) | 2174.14 (1573.51 to 2914.01) | 217.27 (157.24 to 291.2) | -0.65 (-0.68 to -0.62) | <0.001 | 200.55 (125.43 to 292.57) | 49.03 (30.67 to 71.53) | 409.49 (247.34 to 601.56) | 40.92 (24.72 to 60.12) | -0.59 (-0.61 to -0.57) | <0.001 |
| Republic of Chile | 4925.07 (3658.94 to 6419.76) | 344.31 (255.8 to 448.8) | 11382.45 (8425.3 to 14927.56) | 382.5 (283.13 to 501.63) | 0.36 (0.17 to 0.54) | 0.001 | 820.54 (515.67 to 1189.19) | 57.36 (36.05 to 83.14) | 1866.17 (1170.3 to 2713.22) | 62.71 (39.33 to 91.18) | 0.32 (0.15 to 0.49) | 0.001 |
| Republic of Colombia | 8523.53 (6290.05 to 11241.91) | 310.13 (228.87 to 409.04) | 26476.43 (19702.86 to 34864.5) | 368.96 (274.57 to 485.85) | 0.62 (0.48 to 0.77) | <0.001 | 1528.89 (974.84 to 2224.46) | 55.63 (35.47 to 80.94) | 4593.1 (2888 to 6661.43) | 64.01 (40.25 to 92.83) | 0.51 (0.4 to 0.62) | <0.001 |
| Republic of Costa Rica | 796.64 (591.01 to 1045.29) | 322.68 (239.39 to 423.39) | 2638.35 (1952.91 to 3434.33) | 369.77 (273.7 to 481.32) | 0.61 (0.4 to 0.83) | <0.001 | 142.27 (89.23 to 206.84) | 57.63 (36.14 to 83.78) | 456.76 (287.66 to 665.15) | 64.02 (40.32 to 93.22) | 0.49 (0.32 to 0.66) | <0.001 |
| Republic of Croatia | 3534.23 (2665.82 to 4594.64) | 443 (334.15 to 575.92) | 3336.3 (2511.23 to 4340.33) | 450.61 (339.17 to 586.21) | 0.18 (0 to 0.36) | 0.055 | 609.09 (392.85 to 886.25) | 76.35 (49.24 to 111.09) | 569.15 (363.28 to 817.27) | 76.87 (49.07 to 110.38) | 0.14 (-0.01 to 0.28) | 0.068 |
| Republic of Cuba | 3469.38 (2552.59 to 4604.66) | 271.96 (200.09 to 360.95) | 6947.62 (5098.04 to 9157.9) | 329.64 (241.89 to 434.51) | 0.15 (-0.03 to 0.34) | 0.103 | 601.73 (375.33 to 885.63) | 47.17 (29.42 to 69.42) | 1178.84 (741.22 to 1713.11) | 55.93 (35.17 to 81.28) | 0.09 (-0.08 to 0.25) | 0.28 |
| Republic of Cyprus | 317.37 (231.27 to 421.64) | 319.96 (233.15 to 425.08) | 730.84 (529.91 to 978.19) | 314.94 (228.35 to 421.53) | 0.07 (0.02 to 0.12) | 0.007 | 53.14 (31.92 to 79.57) | 53.57 (32.18 to 80.22) | 121.51 (73.2 to 180.48) | 52.36 (31.54 to 77.77) | 0.03 (-0.02 to 0.09) | 0.181 |
| Population of Côte d ivoire | 1645.02 (1196.55 to 2198.47) | 235.75 (171.48 to 315.06) | 4400.7 (3167.65 to 5842.27) | 222.26 (159.99 to 295.07) | -0.21 (-0.27 to -0.14) | <0.001 | 308.71 (191.39 to 454.48) | 44.24 (27.43 to 65.13) | 822.18 (493.29 to 1201.1) | 41.52 (24.91 to 60.66) | -0.21 (-0.26 to -0.16) | <0.001 |
| Republic of Djibouti | 78.04 (57.41 to 103.33) | 291.94 (214.78 to 386.56) | 343.03 (251.89 to 454.77) | 284.49 (208.9 to 377.17) | -0.13 (-0.21 to -0.05) | 0.003 | 15.05 (9.45 to 21.83) | 56.3 (35.36 to 81.66) | 65.98 (41.18 to 95.9) | 54.72 (34.15 to 79.54) | -0.12 (-0.19 to -0.05) | 0.001 |
| Republic of Ecuador | 2025.24 (1483.5 to 2683.53) | 256.79 (188.1 to 340.26) | 6023.65 (4422.07 to 7997.43) | 274.84 (201.76 to 364.9) | 0.32 (0.27 to 0.38) | <0.001 | 350.5 (218.7 to 513.13) | 44.44 (27.73 to 65.06) | 1022 (632.94 to 1503.41) | 46.63 (28.88 to 68.6) | 0.25 (0.2 to 0.3) | <0.001 |
| Republic of El Salvador | 1374.74 (1013.35 to 1804.15) | 321.11 (236.7 to 421.41) | 2890.86 (2140.42 to 3792.93) | 335.66 (248.53 to 440.41) | 0.14 (0.11 to 0.17) | <0.001 | 245.92 (152.16 to 355.01) | 57.44 (35.54 to 82.92) | 512.39 (323.22 to 746.25) | 59.49 (37.53 to 86.65) | 0.12 (0.1 to 0.15) | <0.001 |
| Republic of Equatorial Guinea | 129.83 (95.8 to 170.03) | 388.11 (286.38 to 508.29) | 354.31 (260.39 to 467.64) | 330.32 (242.76 to 435.98) | -0.5 (-0.56 to -0.45) | <0.001 | 25.25 (15.89 to 36.28) | 75.49 (47.49 to 108.46) | 69.44 (43.79 to 100.34) | 64.74 (40.82 to 93.55) | -0.47 (-0.52 to -0.41) | <0.001 |
| Republic of Estonia | 1060.6 (794.27 to 1386.63) | 414.16 (310.16 to 541.47) | 950.25 (718.08 to 1236.71) | 430.2 (325.09 to 559.89) | 0.25 (0.16 to 0.34) | <0.001 | 182.82 (116.61 to 264.04) | 71.39 (45.53 to 103.1) | 162.28 (104.28 to 234.73) | 73.47 (47.21 to 106.27) | 0.2 (0.13 to 0.27) | <0.001 |
| Republic of Fiji | 197.07 (145.36 to 262.71) | 301.76 (222.58 to 402.26) | 432.29 (322.7 to 570.42) | 368.09 (274.77 to 485.7) | 0.7 (0.61 to 0.79) | <0.001 | 37.2 (23.38 to 54) | 56.97 (35.8 to 82.69) | 78.29 (49.4 to 112.93) | 66.66 (42.06 to 96.16) | 0.54 (0.47 to 0.62) | <0.001 |
| Republic of Finland | 2498.57 (1830.26 to 3296.96) | 319.88 (234.32 to 422.1) | 3015.25 (2188.46 to 3977.49) | 350.78 (254.6 to 462.73) | 0.63 (0.51 to 0.76) | <0.001 | 413.09 (249.87 to 618.41) | 52.89 (31.99 to 79.17) | 484.11 (289.31 to 726.32) | 56.32 (33.66 to 84.5) | 0.48 (0.37 to 0.6) | <0.001 |
| Republic of Ghana | 2438.3 (1774.79 to 3274.29) | 234.35 (170.58 to 314.7) | 7053.57 (5093.68 to 9325.08) | 224.36 (162.02 to 296.61) | -0.13 (-0.2 to -0.05) | 0.002 | 450.71 (275.57 to 661.94) | 43.32 (26.49 to 63.62) | 1299.61 (797.63 to 1898.77) | 41.34 (25.37 to 60.4) | -0.13 (-0.19 to -0.06) | <0.001 |
| Republic of Guatemala | 1749.95 (1293.47 to 2305.99) | 309.93 (229.08 to 408.4) | 5276.24 (3931.17 to 6926.18) | 316.66 (235.93 to 415.68) | 0.26 (0.19 to 0.33) | <0.001 | 318.13 (200.06 to 463.63) | 56.34 (35.43 to 82.11) | 949.36 (599.25 to 1366.47) | 56.98 (35.96 to 82.01) | 0.21 (0.15 to 0.27) | <0.001 |
| Republic of Guinea | 1250.14 (913.48 to 1650.61) | 268.9 (196.49 to 355.04) | 2164.56 (1550.96 to 2864.22) | 230.86 (165.42 to 305.48) | -0.52 (-0.56 to -0.49) | <0.001 | 230.99 (143.27 to 337.1) | 49.69 (30.82 to 72.51) | 404.23 (244.95 to 590.08) | 43.11 (26.12 to 62.93) | -0.49 (-0.52 to -0.47) | <0.001 |
| Republic of Guinea-Bissau | 158.94 (114.5 to 212.39) | 236.22 (170.17 to 315.67) | 312.81 (224.2 to 417.37) | 211.76 (151.78 to 282.54) | -0.33 (-0.35 to -0.31) | <0.001 | 29.59 (17.85 to 43.67) | 43.97 (26.53 to 64.91) | 58.65 (35.53 to 85.86) | 39.7 (24.05 to 58.13) | -0.3 (-0.32 to -0.29) | <0.001 |
| Republic of Guyana | 151.28 (109.91 to 201.64) | 257.93 (187.39 to 343.8) | 290.52 (213.96 to 385.42) | 296.66 (218.48 to 393.56) | 0.63 (0.43 to 0.83) | <0.001 | 27.6 (17.17 to 40.48) | 47.06 (29.27 to 69.02) | 51.62 (32.01 to 75.93) | 52.72 (32.69 to 77.53) | 0.5 (0.34 to 0.66) | <0.001 |
| Republic of Haiti | 1407.81 (1027.05 to 1858.47) | 281.69 (205.51 to 371.87) | 3263.77 (2374.21 to 4320.32) | 246.35 (179.21 to 326.1) | -0.35 (-0.43 to -0.26) | <0.001 | 254.1 (156.91 to 373.69) | 50.84 (31.4 to 74.77) | 596.37 (366.18 to 871.95) | 45.01 (27.64 to 65.81) | -0.33 (-0.4 to -0.27) | <0.001 |
| Republic of Honduras | 982.78 (728.03 to 1294.65) | 313.82 (232.47 to 413.41) | 3144.89 (2336.78 to 4139.28) | 306.23 (227.54 to 403.06) | 0.07 (0.01 to 0.13) | 0.029 | 179.11 (112.43 to 259.53) | 57.19 (35.9 to 82.87) | 571.31 (361.95 to 829.79) | 55.63 (35.24 to 80.8) | 0.05 (0 to 0.1) | 0.042 |
| Republic of Iceland | 102.25 (75.69 to 135.45) | 345.75 (255.92 to 458) | 190.43 (139.02 to 253.1) | 356.63 (260.35 to 474.01) | 0.32 (0.12 to 0.53) | 0.003 | 17.28 (10.65 to 25.57) | 58.43 (36.01 to 86.45) | 31.32 (18.96 to 46.69) | 58.65 (35.51 to 87.45) | 0.17 (-0.01 to 0.36) | 0.066 |
| Republic of India | 183548.79 (134172.06 to 241835.83) | 253.65 (185.42 to 334.2) | 445481.36 (324942 to 591511.17) | 263.81 (192.42 to 350.28) | -0.03 (-0.11 to 0.06) | 0.533 | 32515.04 (20508.87 to 47013.75) | 44.93 (28.34 to 64.97) | 76069.6 (47429.87 to 110521.27) | 45.05 (28.09 to 65.45) | -0.09 (-0.15 to -0.02) | 0.01 |
| Republic of Indonesia | 66902.62 (49555.15 to 88227.61) | 406.26 (300.92 to 535.76) | 164866.51 (122832.09 to 216378.41) | 411.18 (306.34 to 539.65) | -0.09 (-0.25 to 0.06) | 0.238 | 11849.96 (7644.11 to 16848.46) | 71.96 (46.42 to 102.31) | 29062.1 (18661.45 to 41252.36) | 72.48 (46.54 to 102.88) | -0.08 (-0.2 to 0.05) | 0.208 |
| Republic of Iraq | 4104.85 (3040.94 to 5392.74) | 353.36 (261.78 to 464.23) | 13716.15 (10176.82 to 18062.79) | 335.33 (248.8 to 441.6) | -0.13 (-0.2 to -0.07) | <0.001 | 795.06 (503.71 to 1146.73) | 68.44 (43.36 to 98.72) | 2643.09 (1665.62 to 3799.5) | 64.62 (40.72 to 92.89) | -0.16 (-0.22 to -0.1) | <0.001 |
| Republic of Italy | 43235.1 (32507.55 to 56184.17) | 475.68 (357.65 to 618.15) | 47396.16 (34832.03 to 62498.05) | 416.07 (305.78 to 548.65) | -0.51 (-0.6 to -0.42) | <0.001 | 7226.74 (4601.34 to 10337.19) | 79.51 (50.62 to 113.73) | 7520.35 (4646.11 to 11055.81) | 66.02 (40.79 to 97.06) | -0.62 (-0.74 to -0.51) | <0.001 |
| Republic of Kazakhstan | 7156.08 (5353.73 to 9348.88) | 386.12 (288.87 to 504.43) | 10032.91 (7508.75 to 13126.2) | 367.99 (275.41 to 481.45) | -0.06 (-0.37 to 0.25) | 0.705 | 1243.68 (791.73 to 1787.39) | 67.1 (42.72 to 96.44) | 1752.08 (1104.61 to 2549.88) | 64.26 (40.52 to 93.52) | -0.04 (-0.28 to 0.21) | 0.742 |
| Republic of Kenya | 4599.76 (3385.84 to 6069.48) | 350.6 (258.07 to 462.63) | 13312.59 (9800.76 to 17720.39) | 331.98 (244.4 to 441.9) | -0.14 (-0.2 to -0.07) | <0.001 | 831.1 (531.9 to 1195.61) | 63.35 (40.54 to 91.13) | 2396.54 (1535.93 to 3454.91) | 59.76 (38.3 to 86.16) | -0.15 (-0.2 to -0.11) | <0.001 |
| Republic of Kiribati | 20.7 (15.41 to 27.3) | 344.67 (256.55 to 454.61) | 48.16 (35.91 to 63.26) | 367.72 (274.16 to 483.02) | 0.22 (0.04 to 0.4) | 0.021 | 3.85 (2.46 to 5.52) | 64.09 (40.98 to 91.97) | 8.88 (5.68 to 12.76) | 67.77 (43.34 to 97.44) | 0.19 (0.04 to 0.33) | 0.013 |
| Republic of Korea | 18450.76 (13555.61 to 24254.73) | 375.84 (276.12 to 494.06) | 52839.13 (39592.87 to 68608.01) | 509.48 (381.75 to 661.52) | 0.72 (0.47 to 0.96) | <0.001 | 3317.77 (2087.43 to 4725.37) | 67.58 (42.52 to 96.25) | 9593.53 (6209.09 to 13698.68) | 92.5 (59.87 to 132.08) | 0.69 (0.47 to 0.91) | <0.001 |
| Republic of Latvia | 1868.57 (1405.9 to 2447.9) | 416.56 (313.42 to 545.71) | 1517.29 (1145.54 to 1970.5) | 445.8 (336.57 to 578.96) | 0.2 (0.09 to 0.32) | 0.001 | 323.83 (206.65 to 469.89) | 72.19 (46.07 to 104.75) | 259.72 (167.48 to 372.58) | 76.31 (49.21 to 109.47) | 0.18 (0.08 to 0.27) | 0.001 |
| Republic of Liberia | 374.83 (274.49 to 498.61) | 256.11 (187.55 to 340.68) | 818.02 (585.03 to 1103.87) | 196.41 (140.47 to 265.05) | -0.96 (-1.02 to -0.91) | <0.001 | 69.15 (43.14 to 101.4) | 47.25 (29.48 to 69.28) | 156.31 (94.71 to 230.47) | 37.53 (22.74 to 55.34) | -0.83 (-0.88 to -0.78) | <0.001 |
| Republic of Lithuania | 2365.08 (1774.23 to 3079.31) | 408.92 (306.76 to 532.41) | 2323.53 (1748.59 to 3031.52) | 454.27 (341.86 to 592.68) | 0.17 (-0.03 to 0.38) | 0.092 | 408.92 (267.19 to 590.34) | 70.7 (46.2 to 102.07) | 396.4 (255.18 to 568.28) | 77.5 (49.89 to 111.1) | 0.16 (-0.01 to 0.33) | 0.066 |
| Republic of Madagascar | 2607.2 (1932.34 to 3427.49) | 332.5 (246.43 to 437.11) | 6496.75 (4768.52 to 8665.96) | 286.42 (210.23 to 382.05) | -0.45 (-0.61 to -0.28) | <0.001 | 492.38 (311.52 to 718.7) | 62.79 (39.73 to 91.66) | 1231.27 (771.41 to 1788.57) | 54.28 (34.01 to 78.85) | -0.42 (-0.56 to -0.27) | <0.001 |
| Republic of Malawi | 2075.38 (1539.88 to 2737.21) | 326.61 (242.34 to 430.76) | 3940.87 (2911.4 to 5194.31) | 297.14 (219.51 to 391.64) | -0.1 (-0.2 to -0.01) | 0.031 | 392.74 (249.2 to 564.54) | 61.81 (39.22 to 88.84) | 749.99 (474.74 to 1090.41) | 56.55 (35.79 to 82.21) | -0.11 (-0.19 to -0.03) | 0.012 |
| Republic of Maldives | 55.29 (41.19 to 72.59) | 400.79 (298.6 to 526.17) | 198.68 (148.82 to 260.11) | 388.16 (290.76 to 508.2) | -0.48 (-0.77 to -0.19) | 0.002 | 10.21 (6.51 to 14.53) | 73.97 (47.19 to 105.32) | 36.69 (23.65 to 52.89) | 71.68 (46.21 to 103.33) | -0.39 (-0.63 to -0.14) | 0.003 |
| Republic of Mali | 1660.23 (1220.52 to 2206.01) | 261.76 (192.43 to 347.81) | 3542.92 (2605.32 to 4687.21) | 239.15 (175.86 to 316.4) | -0.27 (-0.3 to -0.25) | <0.001 | 305.8 (190.82 to 446.56) | 48.21 (30.08 to 70.41) | 657.62 (409.12 to 968.04) | 44.39 (27.62 to 65.34) | -0.25 (-0.27 to -0.23) | <0.001 |
| Republic of Malta | 173.17 (125.4 to 230.19) | 313.52 (227.04 to 416.76) | 251.09 (179.91 to 332.96) | 352.5 (252.57 to 467.43) | 0.73 (0.57 to 0.9) | <0.001 | 28.84 (17.55 to 42.72) | 52.22 (31.77 to 77.34) | 40.64 (24 to 60.99) | 57.06 (33.7 to 85.62) | 0.57 (0.42 to 0.73) | <0.001 |
| Republic of Mauritius | 427.53 (319.43 to 562.39) | 397.02 (296.63 to 522.26) | 1079.38 (810.92 to 1410.37) | 482.19 (362.26 to 630.05) | 1.1 (0.8 to 1.39) | <0.001 | 77.8 (49.85 to 111.68) | 72.25 (46.3 to 103.71) | 190.52 (123.19 to 272.21) | 85.11 (55.03 to 121.6) | 0.93 (0.68 to 1.17) | <0.001 |
| Republic of Moldova | 2371.97 (1788.32 to 3100.33) | 393.37 (296.58 to 514.17) | 2825.65 (2118.95 to 3691.1) | 418.05 (313.49 to 546.09) | 0.48 (0.17 to 0.78) | 0.004 | 411.92 (268.26 to 594.46) | 68.31 (44.49 to 98.59) | 486.18 (309.98 to 706.26) | 71.93 (45.86 to 104.49) | 0.42 (0.17 to 0.67) | 0.002 |
| Republic of Mozambique | 3307.24 (2461.7 to 4343.91) | 320.05 (238.22 to 420.36) | 6279.57 (4649.97 to 8281.61) | 293.95 (217.67 to 387.67) | -0.25 (-0.29 to -0.2) | <0.001 | 629.03 (400.26 to 909.27) | 60.87 (38.73 to 87.99) | 1194.72 (753.43 to 1718.54) | 55.93 (35.27 to 80.45) | -0.24 (-0.28 to -0.2) | <0.001 |
| Republic of Namibia | 344.49 (256.76 to 451.65) | 345.27 (257.34 to 452.68) | 780.22 (576.52 to 1022.32) | 319.35 (235.97 to 418.44) | -0.16 (-0.24 to -0.09) | <0.001 | 64.01 (40.56 to 92.78) | 64.16 (40.65 to 92.99) | 144.58 (90.48 to 209.55) | 59.18 (37.03 to 85.77) | -0.18 (-0.24 to -0.11) | <0.001 |
| Republic of Nauru | 2.22 (1.62 to 2.92) | 303.91 (222.5 to 401.13) | 3.27 (2.43 to 4.3) | 318.73 (237 to 419.46) | 0.54 (0.37 to 0.71) | <0.001 | 0.42 (0.27 to 0.61) | 57.79 (36.69 to 83.1) | 0.61 (0.39 to 0.88) | 59.84 (38.35 to 86.2) | 0.42 (0.29 to 0.56) | <0.001 |
| Republic of Nicaragua | 752.5 (558.95 to 995.66) | 308.36 (229.05 to 408.01) | 2523.89 (1872.76 to 3336.17) | 326.84 (242.52 to 432.02) | 0.28 (0.18 to 0.39) | <0.001 | 135.91 (85.93 to 197.08) | 55.69 (35.21 to 80.76) | 449.24 (285.41 to 653.73) | 58.18 (36.96 to 84.66) | 0.24 (0.15 to 0.32) | <0.001 |
| Republic of Niue | 0.92 (0.68 to 1.2) | 367.42 (270.4 to 482.05) | 1.04 (0.77 to 1.36) | 400.13 (298.01 to 523.02) | 0.16 (0.1 to 0.23) | <0.001 | 0.17 (0.11 to 0.24) | 66.27 (42.3 to 96.02) | 0.18 (0.12 to 0.27) | 70.82 (44.99 to 102.43) | 0.12 (0.06 to 0.17) | <0.001 |
| Republic of Palau | 4.69 (3.47 to 6.22) | 331.59 (245.53 to 439.62) | 13.33 (9.91 to 17.4) | 400.12 (297.39 to 522.42) | 0.93 (0.68 to 1.18) | <0.001 | 0.86 (0.54 to 1.26) | 60.78 (38.43 to 88.78) | 2.37 (1.52 to 3.37) | 71.17 (45.6 to 101.21) | 0.77 (0.57 to 0.97) | <0.001 |
| Republic of Panama | 635.66 (469.29 to 840.03) | 309.85 (228.76 to 409.47) | 1898.65 (1410.99 to 2498.34) | 344.32 (255.89 to 453.08) | 0.31 (0.25 to 0.38) | <0.001 | 113.88 (71.17 to 165.24) | 55.51 (34.69 to 80.54) | 331.45 (206.18 to 478.55) | 60.11 (37.39 to 86.79) | 0.24 (0.19 to 0.29) | <0.001 |
| Republic of Paraguay | 1215 (912.43 to 1596.44) | 391.43 (293.95 to 514.32) | 3208.41 (2407.59 to 4187.2) | 396.67 (297.66 to 517.68) | 0.17 (0.05 to 0.3) | 0.008 | 222.58 (143.42 to 321.4) | 71.71 (46.2 to 103.54) | 579.39 (377.55 to 834.54) | 71.63 (46.68 to 103.18) | 0.11 (0 to 0.21) | 0.043 |
| Republic of Peru | 4551.45 (3329.57 to 6069.42) | 257.02 (188.02 to 342.75) | 12258.6 (8949.48 to 16225.52) | 265.43 (193.78 to 351.32) | 0.11 (0.06 to 0.16) | <0.001 | 774.33 (473.7 to 1142.1) | 43.73 (26.75 to 64.5) | 2052.18 (1260.6 to 3009.74) | 44.43 (27.3 to 65.17) | 0.06 (0.02 to 0.09) | 0.002 |
| Republic of Poland | 23185.43 (17490.7 to 30087.43) | 433.09 (326.72 to 562.02) | 28926.07 (21760.82 to 37627.03) | 436.46 (328.34 to 567.74) | 0.65 (0.37 to 0.93) | <0.001 | 3946.27 (2553.38 to 5602.48) | 73.71 (47.7 to 104.65) | 4886.87 (3148.53 to 6990.49) | 73.74 (47.51 to 105.48) | 0.5 (0.28 to 0.73) | <0.001 |
| Republic of Rwanda | 1626.09 (1207.98 to 2138.39) | 347.1 (257.85 to 456.45) | 3892.6 (2887.07 to 5124.15) | 329.08 (244.07 to 433.19) | 0.19 (-0.04 to 0.42) | 0.096 | 303.66 (192.71 to 435.11) | 64.82 (41.14 to 92.88) | 730.42 (461.78 to 1056) | 61.75 (39.04 to 89.27) | 0.16 (-0.03 to 0.36) | 0.095 |
| Republic of San Marino | 12.13 (8.92 to 16.13) | 356.15 (261.73 to 473.57) | 20.94 (14.96 to 28.04) | 318.67 (227.59 to 426.66) | -0.63 (-0.77 to -0.49) | <0.001 | 1.99 (1.22 to 2.96) | 58.43 (35.78 to 86.84) | 3.4 (2.01 to 5.09) | 51.7 (30.65 to 77.51) | -0.63 (-0.74 to -0.51) | <0.001 |
| Republic of Senegal | 1257.43 (918.49 to 1675.11) | 254.19 (185.67 to 338.62) | 3148.62 (2276.34 to 4193.03) | 245.57 (177.54 to 327.03) | -0.09 (-0.15 to -0.04) | 0.002 | 232.12 (144.42 to 338.57) | 46.92 (29.19 to 68.44) | 573.73 (350.39 to 836.18) | 44.75 (27.33 to 65.22) | -0.15 (-0.19 to -0.1) | <0.001 |
| Republic of Serbia | 6311.29 (4727.93 to 8262.23) | 417.8 (312.98 to 546.95) | 6278.72 (4676.9 to 8153.35) | 418.55 (311.77 to 543.52) | 0.22 (0.08 to 0.36) | 0.002 | 1091.56 (693.66 to 1582.19) | 72.26 (45.92 to 104.74) | 1089.87 (694.98 to 1569.67) | 72.65 (46.33 to 104.64) | 0.19 (0.08 to 0.31) | 0.002 |
| Republic of Seychelles | 29.77 (22.26 to 38.47) | 465.7 (348.23 to 601.8) | 71.57 (53.31 to 93.31) | 441.82 (329.1 to 575.96) | -0.44 (-0.87 to -0.01) | 0.046 | 5.32 (3.44 to 7.56) | 83.2 (53.75 to 118.22) | 12.83 (8.26 to 18.21) | 79.17 (50.98 to 112.4) | -0.37 (-0.73 to -0.01) | 0.045 |
| Republic of Sierra Leone | 716.46 (525.24 to 944.99) | 259.72 (190.4 to 342.57) | 1403.78 (1015.38 to 1868.77) | 224.87 (162.66 to 299.36) | -0.54 (-0.59 to -0.49) | <0.001 | 133.12 (82 to 192.75) | 48.26 (29.73 to 69.87) | 263.4 (163.03 to 384.07) | 42.19 (26.12 to 61.52) | -0.5 (-0.54 to -0.46) | <0.001 |
| Republic of Singapore | 948.61 (697.73 to 1234.33) | 256.95 (188.99 to 334.34) | 3104.63 (2330.17 to 3996.48) | 296.32 (222.41 to 381.45) | 0.64 (0.39 to 0.9) | <0.001 | 178.49 (115.66 to 250.57) | 48.35 (31.33 to 67.87) | 552.74 (368.02 to 772.47) | 52.76 (35.13 to 73.73) | 0.45 (0.23 to 0.68) | <0.001 |
| Republic of Slovenia | 1247.48 (940.4 to 1612.38) | 418.67 (315.61 to 541.13) | 1577.62 (1196.17 to 2044.8) | 430.34 (326.29 to 557.78) | 0.31 (0.13 to 0.5) | 0.002 | 214.04 (137.99 to 307.84) | 71.84 (46.31 to 103.31) | 268.94 (173.84 to 385.77) | 73.36 (47.42 to 105.23) | 0.27 (0.12 to 0.42) | 0.001 |
| Republic of South Africa | 12257.89 (9030.86 to 16103.82) | 382.17 (281.56 to 502.07) | 27805.91 (20559.67 to 36730.97) | 384.3 (284.15 to 507.66) | 0.14 (-0.05 to 0.33) | 0.132 | 2176.21 (1395.13 to 3127.8) | 67.85 (43.5 to 97.52) | 4841.12 (3089.57 to 7008.74) | 66.91 (42.7 to 96.87) | 0.08 (-0.08 to 0.23) | 0.322 |
| Republic of South Sudan | 971.56 (709.69 to 1288.03) | 300.86 (219.77 to 398.86) | 2160.8 (1582.1 to 2867.3) | 279.74 (204.82 to 371.2) | -0.39 (-0.48 to -0.3) | <0.001 | 187.23 (116.68 to 274.41) | 57.98 (36.13 to 84.98) | 415.17 (260.39 to 606.15) | 53.75 (33.71 to 78.47) | -0.36 (-0.43 to -0.29) | <0.001 |
| Republic of Sudan | 4483.42 (3292.83 to 5925.11) | 331.89 (243.76 to 438.61) | 9932.44 (7262.88 to 13239.65) | 287.71 (210.38 to 383.51) | -0.57 (-0.62 to -0.52) | <0.001 | 878.17 (554.31 to 1269.43) | 65.01 (41.03 to 93.97) | 1957.61 (1220.13 to 2842.22) | 56.71 (35.34 to 82.33) | -0.53 (-0.57 to -0.49) | <0.001 |
| Republic of Suriname | 117.53 (86.49 to 154.85) | 309.47 (227.74 to 407.73) | 282.89 (209.42 to 371.77) | 327.18 (242.21 to 429.98) | -0.06 (-0.28 to 0.16) | 0.561 | 20.95 (13.06 to 30.44) | 55.16 (34.4 to 80.15) | 49.4 (30.88 to 72) | 57.14 (35.71 to 83.27) | -0.09 (-0.27 to 0.09) | 0.294 |
| Republic of Tajikistan | 1452.99 (1088.41 to 1909.71) | 399.73 (299.43 to 525.37) | 3560.05 (2633.53 to 4670.96) | 345.2 (255.36 to 452.92) | -0.61 (-1.21 to -0.01) | 0.047 | 249.94 (157.64 to 361.7) | 68.76 (43.37 to 99.51) | 621.74 (390.91 to 908.35) | 60.29 (37.9 to 88.08) | -0.51 (-0.99 to -0.03) | 0.037 |
| Republic of Trinidad and Tobago | 311.13 (227.53 to 413.31) | 278.08 (203.36 to 369.41) | 754.49 (562.37 to 988.01) | 342.65 (255.4 to 448.71) | 0.94 (0.76 to 1.12) | <0.001 | 56.02 (35.06 to 81.98) | 50.07 (31.34 to 73.27) | 130.56 (81.99 to 191.77) | 59.29 (37.24 to 87.09) | 0.77 (0.62 to 0.92) | <0.001 |
| Republic of Tunisia | 3105.86 (2329.14 to 4085.98) | 441.22 (330.88 to 580.46) | 7696.23 (5767.63 to 10027.54) | 420.96 (315.47 to 548.48) | -0.12 (-0.38 to 0.14) | 0.344 | 583.59 (374.74 to 842.95) | 82.91 (53.24 to 119.75) | 1430.97 (912.81 to 2060.47) | 78.27 (49.93 to 112.7) | -0.16 (-0.39 to 0.06) | 0.156 |
| Republic of Turkey | 23491.81 (17526.63 to 30428.27) | 444.62 (331.72 to 575.91) | 53866.47 (40719.28 to 70637.46) | 430.66 (325.55 to 564.75) | -0.04 (-0.19 to 0.11) | 0.558 | 4417.42 (2805.09 to 6300.75) | 83.61 (53.09 to 119.25) | 10059.46 (6385.1 to 14432.8) | 80.43 (51.05 to 115.39) | -0.08 (-0.22 to 0.05) | 0.198 |
| Republic of Uganda | 3263.8 (2398.93 to 4263.76) | 327.27 (240.54 to 427.53) | 8425.28 (6188.81 to 11124.02) | 301.66 (221.58 to 398.28) | -0.36 (-0.42 to -0.3) | <0.001 | 616.86 (390.84 to 886.97) | 61.85 (39.19 to 88.94) | 1592.87 (1004.48 to 2310.99) | 57.03 (35.96 to 82.74) | -0.33 (-0.38 to -0.28) | <0.001 |
| Republic of Uzbekistan | 5789.62 (4267.25 to 7637.5) | 383.53 (282.68 to 505.94) | 15141.89 (11277.29 to 19904.54) | 340.13 (253.32 to 447.11) | -0.26 (-0.76 to 0.24) | 0.297 | 992.81 (625.37 to 1431.12) | 65.77 (41.43 to 94.8) | 2657.19 (1681.2 to 3848.61) | 59.69 (37.76 to 86.45) | -0.19 (-0.58 to 0.21) | 0.344 |
| Republic of Vanuatu | 29.2 (21.37 to 39.03) | 298.91 (218.8 to 399.59) | 90.45 (66.17 to 119.09) | 304.72 (222.92 to 401.22) | 0.23 (0.16 to 0.31) | <0.001 | 5.46 (3.43 to 8.02) | 55.87 (35.06 to 82.07) | 16.75 (10.65 to 24.09) | 56.43 (35.87 to 81.16) | 0.17 (0.11 to 0.22) | <0.001 |
| Republic of Yemen | 2817.83 (2075.95 to 3712.28) | 372.43 (274.37 to 490.64) | 8060.6 (5973.58 to 10654.41) | 316.38 (234.47 to 418.19) | -0.3 (-0.37 to -0.22) | <0.001 | 546.61 (349.24 to 794.81) | 72.24 (46.16 to 105.05) | 1579.79 (995.1 to 2286.2) | 62.01 (39.06 to 89.73) | -0.3 (-0.37 to -0.23) | <0.001 |
| Republic of Zambia | 1470.8 (1081.93 to 1941.43) | 305.2 (224.51 to 402.86) | 3711.1 (2731.71 to 4899.88) | 283.53 (208.71 to 374.36) | -0.29 (-0.33 to -0.25) | <0.001 | 281.07 (176.31 to 403.75) | 58.32 (36.59 to 83.78) | 712.57 (448.24 to 1030.85) | 54.44 (34.25 to 78.76) | -0.26 (-0.29 to -0.22) | <0.001 |
| Republic of Zimbabwe | 1957.04 (1450.79 to 2600.84) | 313.54 (232.43 to 416.68) | 3978.76 (2942.68 to 5265.64) | 300.22 (222.04 to 397.32) | 0.21 (0.09 to 0.33) | 0.001 | 368.93 (234.54 to 534.99) | 59.11 (37.58 to 85.71) | 746.08 (464.68 to 1083.52) | 56.3 (35.06 to 81.76) | 0.15 (0.05 to 0.24) | 0.005 |
| Republic of the Congo | 669.66 (496.26 to 882.41) | 387.63 (287.26 to 510.78) | 1633.32 (1200.85 to 2138) | 311.72 (229.18 to 408.04) | -0.7 (-0.8 to -0.6) | <0.001 | 129.78 (82.03 to 190.18) | 75.12 (47.48 to 110.08) | 318.48 (203.65 to 451.14) | 60.78 (38.87 to 86.1) | -0.7 (-0.8 to -0.6) | <0.001 |
| Republic of the Gambia | 121.63 (88.18 to 162.18) | 233.27 (169.13 to 311.06) | 358.71 (257.1 to 477.02) | 219.15 (157.07 to 291.43) | -0.28 (-0.37 to -0.2) | <0.001 | 22.94 (14.14 to 33.89) | 43.99 (27.12 to 65) | 67.09 (40.89 to 98.68) | 40.99 (24.98 to 60.29) | -0.3 (-0.37 to -0.23) | <0.001 |
| Republic of the Marshall Islands | 6.91 (5.08 to 9.18) | 290.47 (213.67 to 385.66) | 19.36 (14.29 to 25.64) | 304.24 (224.57 to 402.97) | 0.68 (0.44 to 0.92) | <0.001 | 1.29 (0.83 to 1.88) | 54.4 (34.71 to 79.19) | 3.6 (2.26 to 5.24) | 56.55 (35.49 to 82.32) | 0.56 (0.36 to 0.76) | <0.001 |
| Republic of the Niger | 1117.53 (814.94 to 1487.85) | 233.65 (170.38 to 311.07) | 3654.31 (2668.13 to 4815.2) | 249.07 (181.86 to 328.2) | 0.18 (0.13 to 0.23) | <0.001 | 210.8 (129.82 to 309.08) | 44.07 (27.14 to 64.62) | 671.43 (414.52 to 981.68) | 45.76 (28.25 to 66.91) | 0.1 (0.06 to 0.15) | <0.001 |
| Republic of the Philippines | 19505.45 (14574.08 to 25599.95) | 398.72 (297.91 to 523.3) | 52991.63 (39556.97 to 69575.58) | 413.82 (308.91 to 543.33) | 0.15 (0.04 to 0.26) | 0.009 | 3555.89 (2298.56 to 5046.43) | 72.69 (46.99 to 103.16) | 9461.17 (6150.12 to 13498.57) | 73.88 (48.03 to 105.41) | 0.07 (-0.01 to 0.15) | 0.1 |
| Republic of the Union of Myanmar | 15668.18 (11761.62 to 20574.74) | 449.3 (337.28 to 590.01) | 33021.24 (24699.54 to 43191.4) | 423.76 (316.97 to 554.28) | -0.13 (-0.38 to 0.12) | 0.301 | 2879.75 (1848.33 to 4109.22) | 82.58 (53 to 117.84) | 6028.18 (3854.76 to 8606.23) | 77.36 (49.47 to 110.44) | -0.16 (-0.37 to 0.05) | 0.125 |
| Romania | 14229.78 (10635.56 to 18578.12) | 412.89 (308.6 to 539.06) | 13285.55 (10033.43 to 17299.56) | 393.33 (297.05 to 512.17) | 0.11 (-0.01 to 0.23) | 0.066 | 2468.95 (1574.63 to 3560.6) | 71.64 (45.69 to 103.31) | 2320.58 (1479.87 to 3336.36) | 68.7 (43.81 to 98.78) | 0.07 (-0.02 to 0.16) | 0.102 |
| Russian Federation | 106830.42 (79979.51 to 139253.23) | 453.34 (339.4 to 590.93) | 119901.31 (90426.92 to 156250.37) | 449.11 (338.71 to 585.27) | 0.31 (-0.03 to 0.66) | 0.069 | 17856.35 (11582.94 to 25410.77) | 75.77 (49.15 to 107.83) | 20061.53 (12974.16 to 28522.17) | 75.14 (48.6 to 106.84) | 0.26 (-0.01 to 0.53) | 0.055 |
| Saint Kitts and Nevis | 11.05 (8.17 to 14.31) | 345.12 (255.12 to 446.85) | 32.95 (24.25 to 43.26) | 325.65 (239.66 to 427.55) | 0.21 (-0.32 to 0.74) | 0.435 | 1.89 (1.18 to 2.76) | 58.96 (36.97 to 86.28) | 5.67 (3.53 to 8.22) | 56.02 (34.93 to 81.22) | 0.17 (-0.26 to 0.59) | 0.428 |
| Saint Lucia | 33.09 (24.38 to 43.68) | 304.35 (224.26 to 401.78) | 98.57 (72.86 to 129.38) | 321.27 (237.46 to 421.69) | 0.06 (-0.13 to 0.25) | 0.502 | 5.83 (3.62 to 8.57) | 53.66 (33.33 to 78.83) | 17.15 (10.81 to 25.08) | 55.88 (35.23 to 81.75) | 0.03 (-0.13 to 0.19) | 0.698 |
| Saint Vincent and the Grenadines | 26.85 (20.08 to 35.01) | 329.55 (246.45 to 429.68) | 55.31 (41.2 to 72.73) | 325.55 (242.46 to 428.07) | 0.01 (-0.29 to 0.3) | 0.971 | 4.69 (2.94 to 6.8) | 57.52 (36.1 to 83.41) | 9.64 (5.99 to 13.97) | 56.74 (35.28 to 82.22) | -0.02 (-0.26 to 0.22) | 0.882 |
| Slovak Republic | 2820.13 (2112.78 to 3680.83) | 393.7 (294.95 to 513.85) | 3866.62 (2902.58 to 5034.66) | 400.76 (300.84 to 521.82) | 0.56 (0.37 to 0.76) | <0.001 | 492.54 (314.31 to 709) | 68.76 (43.88 to 98.98) | 669.16 (428.88 to 965.34) | 69.36 (44.45 to 100.05) | 0.43 (0.28 to 0.59) | <0.001 |
| Socialist Republic of Viet Nam | 25351.83 (19067.96 to 33152.31) | 472.49 (355.37 to 617.87) | 65579.44 (49455.69 to 85524.49) | 435.91 (328.74 to 568.49) | -0.2 (-0.64 to 0.25) | 0.38 | 4510.44 (2914.45 to 6453.75) | 84.06 (54.32 to 120.28) | 11728.53 (7573.15 to 16774.02) | 77.96 (50.34 to 111.5) | -0.18 (-0.54 to 0.18) | 0.32 |
| Solomon Islands | 71.79 (52.99 to 94.56) | 332.33 (245.33 to 437.74) | 185.77 (136.78 to 245.96) | 295.1 (217.28 to 390.72) | -0.56 (-0.61 to -0.5) | <0.001 | 13.34 (8.48 to 19.36) | 61.78 (39.24 to 89.61) | 35.32 (22.28 to 50.96) | 56.11 (35.39 to 80.96) | -0.46 (-0.51 to -0.41) | <0.001 |
| State of Eritrea | 750.58 (554.12 to 992.45) | 309.77 (228.69 to 409.59) | 1728.26 (1273.93 to 2280.34) | 302.92 (223.29 to 399.69) | -0.13 (-0.18 to -0.08) | <0.001 | 142.76 (90.27 to 207.72) | 58.92 (37.25 to 85.73) | 325.47 (204.31 to 474.93) | 57.05 (35.81 to 83.24) | -0.14 (-0.18 to -0.1) | <0.001 |
| State of Israel | 1815.75 (1333.1 to 2377.04) | 331.31 (243.24 to 433.72) | 3867.26 (2790.11 to 5148.02) | 311.96 (225.07 to 415.27) | 0.22 (0.05 to 0.39) | 0.012 | 302.96 (185.07 to 446.5) | 55.28 (33.77 to 81.47) | 639.48 (387.91 to 946.55) | 51.58 (31.29 to 76.36) | 0.11 (-0.03 to 0.25) | 0.128 |
| State of Kuwait | 286.34 (211.16 to 378.69) | 295.4 (217.84 to 390.67) | 1865.99 (1365.9 to 2472.6) | 280.3 (205.18 to 371.43) | -0.48 (-0.65 to -0.3) | <0.001 | 56.58 (35.34 to 82.29) | 58.37 (36.46 to 84.89) | 367.32 (228.51 to 536.09) | 55.18 (34.33 to 80.53) | -0.44 (-0.58 to -0.29) | <0.001 |
| State of Libya | 1002.04 (745.17 to 1310.52) | 393.8 (292.85 to 515.03) | 3077.33 (2257.84 to 4066.63) | 316.14 (231.95 to 417.77) | -0.99 (-1.16 to -0.82) | <0.001 | 190.65 (121.39 to 272.16) | 74.93 (47.71 to 106.96) | 594.63 (372.07 to 862.22) | 61.09 (38.22 to 88.58) | -0.89 (-1.04 to -0.74) | <0.001 |
| State of Qatar | 45.45 (33.07 to 61.01) | 233.18 (169.64 to 312.99) | 475.98 (344.41 to 633.15) | 242.7 (175.61 to 322.84) | 0.33 (0.17 to 0.49) | <0.001 | 9.27 (5.71 to 13.56) | 47.54 (29.3 to 69.58) | 95.68 (59.39 to 139.92) | 48.78 (30.28 to 71.34) | 0.24 (0.11 to 0.37) | 0.001 |
| Sultanate of Oman | 319 (234.65 to 423.16) | 337.84 (248.5 to 448.15) | 997.9 (718.93 to 1318) | 281.12 (202.53 to 371.29) | -0.43 (-0.52 to -0.33) | <0.001 | 61.7 (39.07 to 89.84) | 65.35 (41.38 to 95.15) | 195.92 (119.79 to 285.31) | 55.19 (33.75 to 80.37) | -0.4 (-0.49 to -0.32) | <0.001 |
| Swiss Confederation | 3780.55 (2790.04 to 4982.15) | 361.26 (266.61 to 476.09) | 5548.27 (4068.04 to 7317.2) | 360.9 (264.61 to 475.96) | -0.05 (-0.12 to 0.02) | 0.131 | 632.72 (392.93 to 934.77) | 60.46 (37.55 to 89.32) | 899.58 (553.82 to 1329.24) | 58.51 (36.02 to 86.46) | -0.15 (-0.19 to -0.1) | <0.001 |
| Syrian Arab Republic | 3090.09 (2315.43 to 4068.7) | 390.52 (292.62 to 514.2) | 8140.69 (6075.31 to 10715.92) | 381.49 (284.7 to 502.17) | -0.2 (-0.43 to 0.03) | 0.087 | 592 (379.07 to 846.98) | 74.82 (47.91 to 107.04) | 1539.17 (982.72 to 2214.27) | 72.13 (46.05 to 103.77) | -0.22 (-0.42 to -0.02) | 0.03 |
| Taiwan (Province of China) | 5849.1 (4321.97 to 7641.19) | 280.2 (207.04 to 366.05) | 16641.51 (12360.21 to 21515.84) | 356.1 (264.49 to 460.4) | 1.31 (0.95 to 1.67) | <0.001 | 1029.22 (649.66 to 1477.72) | 49.3 (31.12 to 70.79) | 3027.28 (1918.82 to 4264.64) | 64.78 (41.06 to 91.26) | 1.43 (1.08 to 1.77) | <0.001 |
| Togolese Republic | 518.02 (374.56 to 695.6) | 225.56 (163.09 to 302.88) | 1734.16 (1256.86 to 2317.68) | 228.21 (165.4 to 305) | -0.03 (-0.09 to 0.03) | 0.274 | 97.66 (59.81 to 143.63) | 42.52 (26.04 to 62.54) | 321.37 (197.02 to 472.03) | 42.29 (25.93 to 62.12) | -0.07 (-0.11 to -0.02) | 0.006 |
| Tokelau | 0.67 (0.5 to 0.87) | 426.08 (317.56 to 554) | 0.64 (0.48 to 0.84) | 371.26 (275.91 to 486.74) | -0.03 (-0.21 to 0.14) | 0.683 | 0.12 (0.08 to 0.17) | 75.73 (48.59 to 108.38) | 0.12 (0.07 to 0.17) | 67.11 (42.74 to 96.41) | -0.04 (-0.19 to 0.11) | 0.581 |
| Turkmenistan | 1043.64 (777.52 to 1363.42) | 385.67 (287.33 to 503.85) | 2281.13 (1686.66 to 3003.41) | 354.55 (262.15 to 466.82) | -0.15 (-0.62 to 0.33) | 0.534 | 181.13 (115.08 to 260.4) | 66.94 (42.53 to 96.23) | 397.45 (248.75 to 572.91) | 61.77 (38.66 to 89.05) | -0.15 (-0.52 to 0.23) | 0.43 |
| Tuvalu | 3.86 (2.87 to 5.09) | 361.45 (269.07 to 476.71) | 5.43 (4.04 to 7.11) | 398.6 (296.76 to 522.02) | 0.57 (0.42 to 0.72) | <0.001 | 0.71 (0.45 to 1.03) | 66.14 (42.36 to 96.15) | 0.98 (0.63 to 1.41) | 71.86 (46.32 to 103.33) | 0.48 (0.36 to 0.61) | <0.001 |
| Ukraine | 40480.48 (30103.32 to 52985.28) | 459.66 (341.83 to 601.65) | 37402.84 (27902.43 to 48807.65) | 446.06 (332.76 to 582.08) | 0.02 (-0.18 to 0.23) | 0.817 | 6807.93 (4327.85 to 9771.66) | 77.3 (49.14 to 110.96) | 6243.55 (3989.8 to 8933.44) | 74.46 (47.58 to 106.54) | -0.01 (-0.17 to 0.15) | 0.866 |
| Union of the Comoros | 103.58 (77.08 to 137.31) | 322.26 (239.81 to 427.17) | 257.68 (190.81 to 338.19) | 323.17 (239.31 to 424.13) | -0.06 (-0.11 to -0.01) | 0.022 | 19.49 (12.45 to 28.23) | 60.64 (38.72 to 87.83) | 47.93 (30.44 to 68.87) | 60.11 (38.18 to 86.37) | -0.08 (-0.12 to -0.03) | 0.002 |
| United Arab Emirates | 156.9 (112.59 to 209.86) | 229.92 (164.99 to 307.53) | 1756.54 (1262.07 to 2390.49) | 197.82 (142.13 to 269.21) | -0.17 (-0.36 to 0.02) | 0.084 | 32.05 (19.77 to 47.13) | 46.97 (28.98 to 69.06) | 364.17 (222.51 to 536.68) | 41.01 (25.06 to 60.44) | -0.18 (-0.33 to -0.03) | 0.019 |
| United Kingdom of Great Britain and Northern Ireland | 26595.12 (19647.94 to 34928.8) | 319.35 (235.93 to 419.42) | 34941.76 (24619.73 to 46591.83) | 315.94 (222.61 to 421.28) | -0.04 (-0.11 to 0.02) | 0.199 | 4260.22 (2636.08 to 6300.18) | 51.16 (31.65 to 75.65) | 5487.81 (3299.72 to 8171.41) | 49.62 (29.84 to 73.88) | -0.11 (-0.17 to -0.05) | 0.001 |
| United Mexican States | 20027.38 (14744.36 to 26606.01) | 299.94 (220.82 to 398.47) | 57188.47 (41936.44 to 75970.86) | 310.11 (227.4 to 411.95) | 0.14 (0.03 to 0.24) | 0.01 | 3547.64 (2249.29 to 5123.71) | 53.13 (33.69 to 76.74) | 9664.84 (6159.7 to 14021.55) | 52.41 (33.4 to 76.03) | 0.04 (-0.06 to 0.14) | 0.393 |
| United Republic of Tanzania | 5785.02 (4266.81 to 7620.86) | 344.55 (254.13 to 453.89) | 13453.63 (9880.74 to 17703.32) | 291.99 (214.45 to 384.23) | -0.69 (-0.76 to -0.63) | <0.001 | 1089.25 (686.85 to 1571.62) | 64.87 (40.91 to 93.6) | 2566.94 (1622.47 to 3693.24) | 55.71 (35.21 to 80.16) | -0.63 (-0.69 to -0.58) | <0.001 |
| United States Virgin Islands | 37.91 (27.57 to 51.06) | 261.24 (189.97 to 351.86) | 60.08 (44.76 to 78.61) | 382.91 (285.22 to 500.96) | 1.26 (1.19 to 1.34) | <0.001 | 6.81 (4.22 to 10.1) | 46.96 (29.07 to 69.63) | 10.07 (6.29 to 14.71) | 64.18 (40.11 to 93.74) | 1.03 (0.97 to 1.08) | <0.001 |
| United States of America | 163694.37 (123368.06 to 212005.63) | 490.98 (370.03 to 635.89) | 325724.63 (246769.03 to 420633.57) | 609.27 (461.59 to 786.8) | 1.05 (0.9 to 1.2) | <0.001 | 26606.53 (17235.58 to 38203.25) | 79.8 (51.7 to 114.59) | 53135.77 (34492.45 to 75570.74) | 99.39 (64.52 to 141.36) | 0.99 (0.89 to 1.1) | <0.001 |

Note: CI, confidence interval; AAPC, average annual percentage change; EOAD, early-onset Alzheimer’s disease and other dementias.

**Supplementary Table S14. Age-standardized Mortality and DALYs of EOAD in women aged 40-64 years and their AAPCs from 1990 to 2021 at the country levels.**

|  | Mortality |  |  |  |  |  | DALYs |  |  |  |  |  |
| --- | --- | --- | --- | --- | --- | --- | --- | --- | --- | --- | --- | --- |
| Country | No of people with EOAD in 1990 (000s) | Age standardised rate in 1990 (per 100 000) | No of people with EOAD in 2021 (000s) | Age standardised rate in 2021 (per 100 000) | AAPC (95% CI) | *p* value | No of people with EOAD in 1990 (000s) | Age standardised rate in 1990 (per 100 000) | No of people with EOAD in 2021 (000s) | Age standardised rate in 2021 (per 100 000) | AAPC (95% CI) | *p* value |
| American Samoa | 0.13 (0.02 to 0.41) | 3.76 (0.7 to 11.53) | 0.26 (0.05 to 0.81) | 3.66 (0.7 to 11.55) | 0.1 (-0.26 to 0.46) | 0.564 | 6.56 (2.7 to 15.33) | 184.04 (75.82 to 430.01) | 13.09 (5.63 to 30.7) | 186.87 (80.39 to 438.31) | 0.22 (-0.08 to 0.52) | 0.153 |
| Antigua and Barbuda | 0.16 (0.03 to 0.51) | 2.74 (0.54 to 8.72) | 0.41 (0.08 to 1.3) | 2.72 (0.52 to 8.52) | 0.12 (-0.23 to 0.47) | 0.489 | 8.38 (3.64 to 19.75) | 143.8 (62.52 to 338.76) | 22.28 (9.8 to 50.76) | 146.24 (64.33 to 333.15) | 0.18 (-0.12 to 0.49) | 0.235 |
| Arab Republic of Egypt | 151.81 (29.14 to 475.5) | 3.27 (0.63 to 10.24) | 324.13 (62.06 to 1006.88) | 3.07 (0.59 to 9.52) | -0.26 (-0.45 to -0.06) | 0.01 | 7797.57 (3404.68 to 18300.44) | 167.88 (73.3 to 394.02) | 16972.27 (7255.05 to 39014.01) | 160.54 (68.63 to 369.03) | -0.12 (-0.27 to 0.02) | 0.099 |
| Argentine Republic | 121.79 (23.76 to 366.84) | 3 (0.59 to 9.04) | 189.34 (36.78 to 585.54) | 2.93 (0.57 to 9.06) | 0.13 (0.05 to 0.21) | 0.002 | 6750.97 (3071.85 to 15225.01) | 166.43 (75.73 to 375.33) | 10219.74 (4587.8 to 22997.76) | 158.09 (70.97 to 355.75) | 0 (-0.06 to 0.07) | 0.99 |
| Australia | 72.3 (14.42 to 219.34) | 3.29 (0.66 to 9.99) | 149.17 (29.67 to 454.78) | 3.66 (0.73 to 11.17) | 0.72 (0.54 to 0.91) | <0.001 | 3894.08 (1777.34 to 8638.14) | 177.37 (80.96 to 393.46) | 7740 (3445.73 to 17378.96) | 190.04 (84.6 to 426.71) | 0.57 (0.42 to 0.73) | <0.001 |
| Barbados | 0.81 (0.16 to 2.47) | 2.87 (0.55 to 8.77) | 1.75 (0.34 to 5.35) | 3.19 (0.62 to 9.75) | 0.72 (0.4 to 1.04) | <0.001 | 43.01 (19.05 to 97.52) | 152.66 (67.62 to 346.12) | 92.36 (40.94 to 206.45) | 168.43 (74.66 to 376.48) | 0.64 (0.38 to 0.91) | <0.001 |
| Belize | 0.3 (0.06 to 0.95) | 2.62 (0.5 to 8.26) | 1.18 (0.23 to 3.76) | 2.46 (0.47 to 7.83) | -0.18 (-0.49 to 0.13) | 0.244 | 16.2 (7.05 to 36.49) | 141.42 (61.56 to 318.52) | 63.42 (27.93 to 146.77) | 132.15 (58.2 to 305.85) | -0.17 (-0.44 to 0.11) | 0.226 |
| Bermuda | 0.22 (0.04 to 0.68) | 2.64 (0.52 to 7.99) | 0.39 (0.08 to 1.17) | 3.2 (0.63 to 9.51) | 0.73 (0.46 to 1) | <0.001 | 12.25 (5.59 to 27.04) | 144.97 (66.21 to 320.02) | 21.72 (10.01 to 45.85) | 176.43 (81.32 to 372.43) | 0.77 (0.55 to 0.99) | <0.001 |
| Bolivarian Republic of Venezuela | 44.76 (8.7 to 138) | 2.98 (0.58 to 9.18) | 146.27 (28.49 to 455.36) | 3.55 (0.69 to 11.06) | 0.88 (0.64 to 1.12) | <0.001 | 2437.91 (1096.2 to 5558.76) | 162.15 (72.91 to 369.73) | 7700.62 (3337.55 to 17607.91) | 187 (81.05 to 427.58) | 0.76 (0.56 to 0.96) | <0.001 |
| Bosnia and Herzegovina | 22.48 (4.33 to 69.55) | 3.61 (0.7 to 11.18) | 23.56 (4.57 to 68.18) | 3.87 (0.75 to 11.19) | 0.11 (-0.17 to 0.4) | 0.411 | 1190.32 (531.57 to 2649.01) | 191.3 (85.43 to 425.73) | 1244.74 (551 to 2674.38) | 204.21 (90.4 to 438.76) | 0.21 (-0.04 to 0.45) | 0.098 |
| Brunei Darussalam | 0.4 (0.08 to 1.28) | 2.34 (0.45 to 7.47) | 1.7 (0.32 to 5.17) | 2.77 (0.52 to 8.41) | 0.8 (0.41 to 1.2) | <0.001 | 21.59 (9.24 to 51.92) | 125.54 (53.74 to 301.94) | 90.45 (39.55 to 204.67) | 147.08 (64.32 to 332.82) | 0.77 (0.44 to 1.1) | <0.001 |
| Burkina Faso | 21.69 (4.23 to 64.97) | 3.17 (0.62 to 9.49) | 39.8 (7.45 to 122.49) | 2.54 (0.47 to 7.8) | -0.75 (-0.98 to -0.53) | <0.001 | 1052.36 (431.06 to 2461.52) | 153.79 (62.99 to 359.72) | 2016.87 (830.82 to 4668.84) | 128.47 (52.92 to 297.4) | -0.59 (-0.74 to -0.44) | <0.001 |
| Canada | 113.9 (22.51 to 349.4) | 3.1 (0.61 to 9.51) | 233.25 (46.03 to 706.03) | 3.69 (0.73 to 11.17) | 0.87 (0.61 to 1.13) | <0.001 | 7537.23 (3889.68 to 15467.77) | 205.1 (105.85 to 420.91) | 15356.19 (8110.23 to 30522.51) | 242.97 (128.32 to 482.93) | 0.71 (0.6 to 0.83) | <0.001 |
| Central African Republic | 9.37 (1.81 to 28.21) | 4.52 (0.87 to 13.62) | 18.01 (3.11 to 55.44) | 4 (0.69 to 12.32) | -0.59 (-0.78 to -0.39) | <0.001 | 454.78 (186.78 to 1068.26) | 219.54 (90.17 to 515.69) | 886.83 (366.2 to 2080.07) | 197.06 (81.37 to 462.21) | -0.51 (-0.67 to -0.34) | <0.001 |
| Commonwealth of Dominica | 0.21 (0.04 to 0.66) | 3.06 (0.58 to 9.5) | 0.28 (0.06 to 0.88) | 2.93 (0.59 to 9.13) | -0.53 (-0.85 to -0.22) | 0.002 | 11.25 (4.89 to 25.72) | 160.95 (69.93 to 368.19) | 15.24 (6.81 to 34.65) | 157.73 (70.47 to 358.66) | -0.41 (-0.71 to -0.12) | 0.007 |
| Commonwealth of the Bahamas | 0.56 (0.1 to 1.73) | 2.32 (0.44 to 7.23) | 1.65 (0.31 to 5.13) | 2.61 (0.49 to 8.12) | 0.18 (-0.05 to 0.42) | 0.117 | 30 (13.12 to 66.95) | 125.55 (54.89 to 280.17) | 89.17 (39.21 to 206.39) | 141.2 (62.08 to 326.79) | 0.24 (0.04 to 0.45) | 0.023 |
| Cook Islands | 0.07 (0.01 to 0.21) | 4.02 (0.79 to 12.41) | 0.12 (0.02 to 0.36) | 3.98 (0.79 to 12.36) | -0.32 (-0.56 to -0.07) | 0.013 | 3.41 (1.42 to 8.13) | 198.49 (82.65 to 472.61) | 6.1 (2.7 to 14.01) | 208.34 (92.39 to 478.61) | -0.12 (-0.33 to 0.1) | 0.272 |
| Czech Republic | 56.76 (11.24 to 173.37) | 3.57 (0.71 to 10.9) | 62.04 (12.17 to 185.44) | 3.36 (0.66 to 10.03) | 0.51 (0.18 to 0.84) | 0.004 | 2960.78 (1314.54 to 6613.5) | 186.17 (82.66 to 415.85) | 3303.5 (1488.05 to 7227.03) | 178.67 (80.48 to 390.88) | 0.44 (0.14 to 0.74) | 0.005 |
| Democratic People's Republic of Korea | 97.89 (16.94 to 302.9) | 3.53 (0.61 to 10.92) | 186.58 (34.73 to 583.02) | 4.19 (0.78 to 13.09) | -0.19 (-0.48 to 0.11) | 0.209 | 4948.14 (2103.79 to 11588.34) | 178.41 (75.85 to 417.83) | 9025 (3654.84 to 21487.07) | 202.63 (82.06 to 482.42) | -0.28 (-0.56 to -0.01) | 0.046 |
| Democratic Republic of Sao Tome and Principe | 0.27 (0.05 to 0.79) | 3.17 (0.6 to 9.4) | 0.52 (0.1 to 1.65) | 2.65 (0.51 to 8.38) | -0.85 (-1.11 to -0.59) | <0.001 | 12.97 (5.47 to 29.94) | 154.02 (64.91 to 355.42) | 25.04 (10.15 to 61.58) | 127 (51.46 to 312.28) | -0.81 (-1.04 to -0.59) | <0.001 |
| Democratic Republic of Timor-Leste | 1.57 (0.28 to 5.01) | 2.83 (0.51 to 9.03) | 4.44 (0.83 to 13.94) | 3.98 (0.75 to 12.47) | 1.76 (1.4 to 2.13) | <0.001 | 88.05 (39.8 to 201.61) | 158.58 (71.68 to 363.11) | 225.54 (97.26 to 531.04) | 201.86 (87.04 to 475.28) | 1.35 (1.03 to 1.66) | <0.001 |
| Democratic Republic of the Congo | 130.17 (24.37 to 397.69) | 4.73 (0.89 to 14.46) | 335.68 (58.31 to 1011.68) | 4.95 (0.86 to 14.93) | 0.2 (-0.11 to 0.5) | 0.197 | 6253.18 (2517.12 to 15022.07) | 227.4 (91.54 to 546.29) | 15421.97 (5969.31 to 37305.92) | 227.64 (88.11 to 550.67) | 0.05 (-0.21 to 0.3) | 0.717 |
| Democratic Socialist Republic of Sri Lanka | 53.31 (9.94 to 162.9) | 3.16 (0.59 to 9.64) | 132.45 (23.54 to 400.83) | 3.81 (0.68 to 11.52) | 0.9 (0.78 to 1.02) | <0.001 | 2943.4 (1340.7 to 6547.34) | 174.27 (79.38 to 387.64) | 7173 (3236.15 to 15671.27) | 206.12 (92.99 to 450.31) | 0.81 (0.7 to 0.92) | <0.001 |
| Dominican Republic | 13.97 (2.66 to 44.23) | 2.51 (0.48 to 7.95) | 35.39 (6.46 to 109.64) | 2.64 (0.48 to 8.19) | 0.2 (0.09 to 0.3) | 0.001 | 752.7 (325.74 to 1722.69) | 135.36 (58.58 to 309.8) | 1874.48 (813.53 to 4236.12) | 140.1 (60.8 to 316.61) | 0.13 (0.04 to 0.22) | 0.006 |
| Eastern Republic of Uruguay | 13.76 (2.61 to 42.85) | 3.25 (0.62 to 10.11) | 17.21 (3.37 to 53.18) | 3.23 (0.63 to 9.98) | 0.05 (-0.08 to 0.19) | 0.417 | 759.3 (349.24 to 1703.91) | 179.1 (82.38 to 401.91) | 921.56 (414.17 to 2063.42) | 172.98 (77.74 to 387.31) | -0.05 (-0.16 to 0.06) | 0.324 |
| Federal Democratic Republic of Ethiopia | 117.7 (23.23 to 375.15) | 3.69 (0.73 to 11.77) | 261.05 (47.84 to 792.27) | 3.5 (0.64 to 10.61) | -0.01 (-0.1 to 0.08) | 0.835 | 5909.77 (2598.88 to 14214.44) | 185.49 (81.57 to 446.14) | 12836.74 (5402.62 to 30120.01) | 171.97 (72.38 to 403.51) | -0.06 (-0.14 to 0.02) | 0.156 |
| Federal Democratic Republic of Nepal | 35.69 (6.68 to 111.8) | 2.38 (0.45 to 7.45) | 104.76 (18.95 to 327.19) | 2.95 (0.53 to 9.22) | 0.88 (0.79 to 0.97) | <0.001 | 1900.01 (821.26 to 4400) | 126.59 (54.72 to 293.16) | 5043.97 (2023.7 to 12122.43) | 142.09 (57.01 to 341.5) | 0.49 (0.43 to 0.55) | <0.001 |
| Federal Republic of Germany | 455.07 (88.15 to 1383.05) | 3.59 (0.7 to 10.91) | 579.66 (116.11 to 1750.26) | 3.94 (0.79 to 11.9) | -0.03 (-0.33 to 0.26) | 0.814 | 24958.73 (11372.57 to 54381.69) | 196.83 (89.69 to 428.86) | 31519.29 (14705.14 to 68938.48) | 214.31 (99.99 to 468.74) | 0.01 (-0.24 to 0.27) | 0.924 |
| Federal Republic of Nigeria | 119.81 (21.8 to 380.06) | 2.2 (0.4 to 6.99) | 462.9 (80.88 to 1566.63) | 2.56 (0.45 to 8.67) | 0.57 (0.26 to 0.88) | 0.001 | 6464.28 (2920.25 to 14918.35) | 118.9 (53.71 to 274.4) | 23138.61 (9676.2 to 58237.99) | 128.03 (53.54 to 322.23) | 0.26 (0.02 to 0.51) | 0.036 |
| Federal Republic of Somalia | 12.82 (2.42 to 39.9) | 2.48 (0.47 to 7.73) | 39.24 (7.41 to 118.9) | 2.89 (0.55 to 8.77) | 1.22 (0.92 to 1.53) | <0.001 | 692.42 (296.54 to 1599.33) | 134.14 (57.45 to 309.84) | 1954.53 (833.89 to 4490.12) | 144.12 (61.49 to 331.09) | 0.89 (0.6 to 1.18) | <0.001 |
| Federated States of Micronesia | 0.28 (0.05 to 0.88) | 4.34 (0.8 to 13.57) | 0.53 (0.1 to 1.65) | 4.36 (0.8 to 13.48) | 0.39 (-0.07 to 0.86) | 0.096 | 13.3 (5.39 to 33.16) | 205.48 (83.29 to 512.33) | 26.21 (11 to 62.06) | 214.43 (89.96 to 507.72) | 0.51 (0.13 to 0.89) | 0.011 |
| Federative Republic of Brazil | 631.45 (123.71 to 1963.38) | 4.58 (0.9 to 14.23) | 1773.88 (352.63 to 5463.75) | 5.22 (1.04 to 16.07) | 0.49 (0.34 to 0.65) | <0.001 | 31423.72 (14029.46 to 72862.81) | 227.82 (101.71 to 528.25) | 86185.81 (37959.96 to 202055.01) | 253.49 (111.65 to 594.28) | 0.44 (0.32 to 0.56) | <0.001 |
| French Republic | 264.29 (51.21 to 807.9) | 3.24 (0.63 to 9.92) | 333.36 (65.61 to 994.75) | 3.09 (0.61 to 9.22) | 0.17 (-0.08 to 0.42) | 0.181 | 13012.91 (5461.98 to 30207.02) | 159.71 (67.04 to 370.74) | 16794.12 (7281.41 to 38231.09) | 155.58 (67.46 to 354.18) | 0.21 (0.01 to 0.42) | 0.041 |
| Gabonese Republic | 4.35 (0.82 to 12.93) | 5.75 (1.08 to 17.12) | 8.07 (1.38 to 25.32) | 4.52 (0.77 to 14.17) | -1.09 (-1.4 to -0.79) | <0.001 | 203.76 (83.02 to 478.49) | 269.71 (109.89 to 633.34) | 384.95 (155.61 to 936.09) | 215.51 (87.12 to 524.05) | -0.96 (-1.23 to -0.68) | <0.001 |
| Georgia | 31.46 (6.17 to 95.94) | 3.88 (0.76 to 11.82) | 26.42 (5.14 to 79.64) | 4.3 (0.84 to 12.95) | 0.23 (-0.22 to 0.67) | 0.312 | 1664.08 (757.22 to 3774.61) | 205.01 (93.29 to 465.01) | 1352.75 (604.46 to 2981.65) | 219.94 (98.28 to 484.77) | 0.17 (-0.22 to 0.55) | 0.383 |
| Grand Duchy of Luxembourg | 1.56 (0.3 to 4.85) | 2.68 (0.52 to 8.36) | 2.38 (0.47 to 7.18) | 2.16 (0.43 to 6.51) | -0.75 (-0.95 to -0.56) | <0.001 | 81.38 (35.55 to 183.18) | 140.23 (61.27 to 315.66) | 131.16 (58.14 to 288.61) | 118.96 (52.74 to 261.77) | -0.56 (-0.74 to -0.38) | <0.001 |
| Greenland | 0.18 (0.03 to 0.55) | 3.31 (0.62 to 10.28) | 0.36 (0.07 to 1.08) | 4.21 (0.8 to 12.51) | 0.45 (-0.1 to 1) | 0.106 | 10.09 (4.71 to 21.91) | 189.7 (88.53 to 412.04) | 20.96 (10.12 to 44.34) | 243.8 (117.69 to 515.82) | 0.58 (0.11 to 1.05) | 0.018 |
| Grenada | 0.24 (0.05 to 0.73) | 3.32 (0.64 to 10.27) | 0.42 (0.08 to 1.28) | 2.76 (0.54 to 8.49) | -0.37 (-0.57 to -0.16) | 0.001 | 12.22 (5.43 to 27.67) | 172.24 (76.5 to 389.9) | 22.34 (9.51 to 50.41) | 148.72 (63.3 to 335.54) | -0.3 (-0.47 to -0.14) | 0.001 |
| Guam | 0.44 (0.09 to 1.37) | 3.63 (0.71 to 11.27) | 1.01 (0.2 to 3.06) | 4.23 (0.83 to 12.84) | 0.61 (0.32 to 0.91) | <0.001 | 22.23 (9.58 to 53.03) | 182.54 (78.66 to 435.49) | 51.25 (22.42 to 118.69) | 214.8 (93.95 to 497.45) | 0.63 (0.4 to 0.87) | <0.001 |
| Hashemite Kingdom of Jordan | 7.09 (1.39 to 22) | 3.12 (0.61 to 9.69) | 34.67 (6.51 to 105.4) | 2.75 (0.52 to 8.35) | -0.98 (-1.21 to -0.74) | <0.001 | 375.47 (161.74 to 872.42) | 165.43 (71.26 to 384.39) | 1979.35 (912.84 to 4300.04) | 156.72 (72.27 to 340.46) | -0.64 (-0.82 to -0.46) | <0.001 |
| Hellenic Republic | 57.51 (11.18 to 176.04) | 3.53 (0.69 to 10.79) | 61.17 (11.92 to 182.98) | 3.24 (0.63 to 9.68) | -0.44 (-0.58 to -0.3) | <0.001 | 3038.14 (1380.35 to 7001.91) | 186.29 (84.64 to 429.33) | 3233.33 (1453.48 to 7181.3) | 171.12 (76.93 to 380.07) | -0.4 (-0.51 to -0.28) | <0.001 |
| Hungary | 62.43 (12 to 192.87) | 3.7 (0.71 to 11.44) | 60.93 (11.76 to 185.66) | 3.46 (0.67 to 10.55) | 0.42 (0.21 to 0.64) | <0.001 | 3260.12 (1439.88 to 7437.95) | 193.43 (85.43 to 441.32) | 3222.84 (1451.64 to 7124.08) | 183.22 (82.53 to 405) | 0.37 (0.18 to 0.57) | <0.001 |
| Independent State of Papua New Guinea | 11.09 (2.04 to 35.46) | 3.71 (0.68 to 11.85) | 28.54 (5.1 to 88) | 3.03 (0.54 to 9.36) | -0.65 (-0.84 to -0.46) | <0.001 | 557.55 (235.52 to 1364.67) | 186.31 (78.7 to 456.01) | 1465.68 (607.47 to 3431.45) | 155.84 (64.59 to 364.85) | -0.57 (-0.73 to -0.42) | <0.001 |
| Independent State of Samoa | 0.55 (0.1 to 1.67) | 4.64 (0.88 to 13.99) | 0.91 (0.17 to 2.78) | 4.5 (0.85 to 13.82) | -0.15 (-0.42 to 0.11) | 0.255 | 25.94 (10.28 to 60.53) | 217.52 (86.24 to 507.61) | 42.61 (16.78 to 105.63) | 211.64 (83.37 to 524.72) | -0.13 (-0.36 to 0.09) | 0.243 |
| Ireland | 12.33 (2.41 to 38.07) | 2.94 (0.57 to 9.07) | 22.41 (4.44 to 67.63) | 2.72 (0.54 to 8.2) | -0.06 (-0.14 to 0.02) | 0.16 | 645.63 (287.29 to 1454) | 153.8 (68.44 to 346.36) | 1193.56 (545.42 to 2643.77) | 144.65 (66.1 to 320.41) | -0.04 (-0.1 to 0.03) | 0.235 |
| Islamic Republic of Afghanistan | 65.47 (11.8 to 206.04) | 6.55 (1.18 to 20.6) | 70.71 (12.51 to 222.5) | 3.33 (0.59 to 10.46) | -2.9 (-3.52 to -2.27) | <0.001 | 2828.7 (1042.91 to 7293.16) | 282.79 (104.26 to 729.12) | 3394.36 (1280.07 to 8444.53) | 159.63 (60.2 to 397.12) | -2.64 (-3.21 to -2.07) | <0.001 |
| Islamic Republic of Iran | 151.63 (29.63 to 469.64) | 3.89 (0.76 to 12.06) | 403.67 (75.5 to 1226) | 3.34 (0.62 to 10.15) | -0.36 (-0.63 to -0.1) | 0.009 | 7957.03 (3635.99 to 18315.7) | 204.36 (93.38 to 470.41) | 21893.62 (10279.74 to 48468.52) | 181.18 (85.07 to 401.1) | -0.21 (-0.44 to 0.02) | 0.074 |
| Islamic Republic of Mauritania | 3.94 (0.75 to 12.23) | 2.79 (0.53 to 8.65) | 10.33 (1.78 to 32.74) | 2.98 (0.51 to 9.45) | 0.09 (-0.1 to 0.28) | 0.338 | 193.5 (80.89 to 463.67) | 136.82 (57.2 to 327.84) | 490.94 (198.62 to 1214.79) | 141.76 (57.35 to 350.77) | 0.06 (-0.1 to 0.23) | 0.435 |
| Islamic Republic of Pakistan | 203.26 (38.15 to 631.04) | 2.56 (0.48 to 7.95) | 556.92 (102.03 to 1759.53) | 2.67 (0.49 to 8.44) | 0.02 (-0.07 to 0.11) | 0.636 | 10167.14 (4250.14 to 23734.65) | 128.08 (53.54 to 299) | 26640.25 (10472.59 to 65565.68) | 127.81 (50.24 to 314.55) | -0.09 (-0.15 to -0.02) | 0.008 |
| Jamaica | 5.4 (1.01 to 16.83) | 2.81 (0.53 to 8.74) | 10.69 (2 to 33.11) | 2.81 (0.52 to 8.7) | -0.03 (-0.26 to 0.2) | 0.771 | 304.71 (141.44 to 666.89) | 158.34 (73.49 to 346.53) | 597.74 (278.13 to 1332.72) | 157.05 (73.08 to 350.16) | -0.06 (-0.27 to 0.16) | 0.596 |
| Japan | 798.03 (153.14 to 2462.3) | 3.68 (0.71 to 11.36) | 815.19 (162.51 to 2476.72) | 3.81 (0.76 to 11.58) | 0.25 (-0.03 to 0.52) | 0.082 | 42485.08 (19751.18 to 95549.39) | 196 (91.12 to 440.8) | 43838.53 (20441.76 to 95958) | 204.96 (95.57 to 448.64) | 0.24 (-0.01 to 0.49) | 0.056 |
| Kingdom of Bahrain | 0.91 (0.18 to 2.87) | 3.2 (0.63 to 10.03) | 3.95 (0.73 to 12.01) | 2.54 (0.47 to 7.73) | -1.05 (-1.63 to -0.47) | 0.001 | 48.43 (20.66 to 113.27) | 169.39 (72.26 to 396.19) | 227.26 (102.66 to 498.54) | 146.27 (66.07 to 320.87) | -0.6 (-1.13 to -0.08) | 0.025 |
| Kingdom of Belgium | 55.49 (10.56 to 168.33) | 3.72 (0.71 to 11.29) | 68.25 (13.31 to 204.17) | 3.59 (0.7 to 10.75) | -0.05 (-0.29 to 0.19) | 0.65 | 2939.84 (1345.82 to 6555.92) | 197.18 (90.27 to 439.71) | 3584.06 (1617.49 to 7971.69) | 188.66 (85.14 to 419.62) | -0.06 (-0.26 to 0.15) | 0.564 |
| Kingdom of Bhutan | 1.03 (0.19 to 3.21) | 2.63 (0.48 to 8.2) | 2.67 (0.48 to 8.34) | 3.25 (0.58 to 10.17) | 0.73 (0.65 to 0.81) | <0.001 | 51.65 (21.02 to 120.12) | 132.09 (53.77 to 307.21) | 122.32 (47.39 to 302.91) | 149.02 (57.74 to 369.03) | 0.45 (0.39 to 0.51) | <0.001 |
| Kingdom of Cambodia | 30.2 (5.94 to 96.16) | 3.76 (0.74 to 11.96) | 100.1 (17.58 to 318.14) | 4.98 (0.88 to 15.84) | 1.16 (0.92 to 1.4) | <0.001 | 1555.83 (673.71 to 3713.66) | 193.55 (83.81 to 462) | 4798.79 (1929.85 to 11724.85) | 238.97 (96.1 to 583.87) | 0.9 (0.69 to 1.11) | <0.001 |
| Kingdom of Denmark | 21.91 (4.23 to 68.48) | 2.83 (0.54 to 8.83) | 28.42 (5.61 to 85.5) | 3.01 (0.59 to 9.04) | 0.38 (0.23 to 0.53) | <0.001 | 1107.72 (495.36 to 2567.7) | 142.86 (63.88 to 331.15) | 1421.6 (603.4 to 3287.81) | 150.33 (63.81 to 347.67) | 0.25 (0.13 to 0.38) | <0.001 |
| Kingdom of Eswatini | 1.53 (0.29 to 4.74) | 2.96 (0.56 to 9.19) | 3.63 (0.62 to 11.02) | 3.57 (0.61 to 10.84) | 0.82 (0.7 to 0.95) | <0.001 | 77.47 (32.5 to 179.81) | 150.19 (63.01 to 348.57) | 173.87 (69.95 to 410.51) | 171.02 (68.8 to 403.8) | 0.6 (0.5 to 0.69) | <0.001 |
| Kingdom of Lesotho | 4.79 (0.92 to 15.04) | 3.29 (0.63 to 10.33) | 6.93 (1.23 to 21.15) | 3.89 (0.69 to 11.87) | 0.88 (0.73 to 1.04) | <0.001 | 248.02 (105.79 to 569.42) | 170.35 (72.66 to 391.09) | 335.62 (135.78 to 799.09) | 188.44 (76.24 to 448.67) | 0.62 (0.5 to 0.74) | <0.001 |
| Kingdom of Morocco | 79.59 (15.05 to 241.9) | 3.86 (0.73 to 11.74) | 204.06 (35.07 to 647.18) | 4.08 (0.7 to 12.95) | 0.2 (-0.05 to 0.44) | 0.107 | 4023.98 (1721.9 to 9204.2) | 195.37 (83.6 to 446.88) | 10032.93 (4192.9 to 23997.3) | 200.72 (83.89 to 480.1) | 0.12 (-0.1 to 0.33) | 0.27 |
| Kingdom of Norway | 19.31 (3.69 to 59.04) | 3.39 (0.65 to 10.36) | 26.94 (5.31 to 81.83) | 3.16 (0.62 to 9.6) | 0.09 (-0.07 to 0.26) | 0.258 | 1012.06 (465.63 to 2260.09) | 177.55 (81.69 to 396.5) | 1446.19 (675.74 to 3171.64) | 169.67 (79.28 to 372.11) | 0.08 (-0.04 to 0.2) | 0.174 |
| Kingdom of Saudi Arabia | 24.47 (4.46 to 77.47) | 2.94 (0.54 to 9.32) | 90.04 (16.9 to 283.01) | 2.22 (0.42 to 6.97) | -1.06 (-1.13 to -0.99) | <0.001 | 1261.63 (540.55 to 2999.47) | 151.74 (65.01 to 360.75) | 4927.78 (2121.64 to 11211.22) | 121.39 (52.26 to 276.18) | -0.87 (-0.94 to -0.81) | <0.001 |
| Kingdom of Spain | 214.37 (41.63 to 653.07) | 3.86 (0.75 to 11.75) | 288.25 (57.69 to 853.02) | 3.29 (0.66 to 9.73) | -0.71 (-0.87 to -0.54) | <0.001 | 10863.11 (4723.24 to 24482.07) | 195.38 (84.95 to 440.32) | 14730.81 (6436.97 to 32876.73) | 168.02 (73.42 to 375) | -0.6 (-0.73 to -0.46) | <0.001 |
| Kingdom of Sweden | 37.35 (7.18 to 113.58) | 2.91 (0.56 to 8.86) | 46.91 (9.21 to 138.54) | 2.99 (0.59 to 8.84) | 0.23 (0.09 to 0.38) | 0.003 | 2102.4 (976.25 to 4656.26) | 164.07 (76.19 to 363.38) | 2726.94 (1288.94 to 5674.96) | 174.03 (82.26 to 362.17) | 0.35 (0.22 to 0.47) | <0.001 |
| Kingdom of Thailand | 241 (46.36 to 743.82) | 4.09 (0.79 to 12.63) | 682.37 (131.76 to 2014.4) | 5.03 (0.97 to 14.85) | 0.44 (0.13 to 0.74) | 0.006 | 12188.94 (5308.96 to 28025.24) | 207.03 (90.17 to 476) | 33236.8 (14429.02 to 77016.76) | 245.08 (106.4 to 567.91) | 0.53 (0.29 to 0.78) | <0.001 |
| Kingdom of Tonga | 0.36 (0.07 to 1.12) | 4.31 (0.81 to 13.44) | 0.41 (0.08 to 1.26) | 3.71 (0.72 to 11.37) | -0.63 (-0.7 to -0.56) | <0.001 | 17.31 (7.11 to 41.95) | 208.14 (85.5 to 504.35) | 20.66 (8.65 to 48.07) | 186.06 (77.87 to 432.89) | -0.53 (-0.59 to -0.46) | <0.001 |
| Kingdom of the Netherlands | 65.31 (12.83 to 200.62) | 3.09 (0.61 to 9.49) | 102.43 (20.14 to 309.03) | 3.53 (0.69 to 10.66) | 0.54 (0.37 to 0.71) | <0.001 | 3408.86 (1508.02 to 7822.58) | 161.24 (71.33 to 370.01) | 5376 (2427.82 to 11748.16) | 185.39 (83.72 to 405.12) | 0.58 (0.43 to 0.73) | <0.001 |
| Kyrgyz Republic | 14.62 (2.78 to 45.69) | 3.7 (0.7 to 11.55) | 26.09 (5.02 to 81.89) | 3.25 (0.63 to 10.21) | -0.62 (-1.31 to 0.06) | 0.073 | 771.36 (349.18 to 1771.38) | 195.04 (88.29 to 447.9) | 1397.82 (629.77 to 3198.16) | 174.27 (78.51 to 398.72) | -0.43 (-1.03 to 0.18) | 0.161 |
| Lao People's Democratic Republic | 11.07 (2.05 to 35.3) | 3.4 (0.63 to 10.84) | 27.81 (5.24 to 86.15) | 3.58 (0.67 to 11.09) | 0.04 (-0.06 to 0.15) | 0.392 | 593.71 (262.88 to 1402.93) | 182.36 (80.74 to 430.91) | 1457.6 (618.4 to 3370.83) | 187.68 (79.62 to 434.03) | -0.01 (-0.12 to 0.11) | 0.896 |
| Lebanese Republic | 11.27 (2.14 to 35.37) | 3.56 (0.68 to 11.17) | 22.76 (4.52 to 69.71) | 3.03 (0.6 to 9.28) | -0.61 (-0.75 to -0.47) | <0.001 | 618.21 (276.98 to 1397.9) | 195.2 (87.46 to 441.38) | 1324.19 (619.09 to 2855.34) | 176.25 (82.4 to 380.05) | -0.41 (-0.5 to -0.32) | <0.001 |
| Malaysia | 60.2 (11.73 to 185.34) | 4.06 (0.79 to 12.5) | 182.31 (34.79 to 551.04) | 4.71 (0.9 to 14.25) | 0.75 (0.52 to 0.99) | <0.001 | 3095.54 (1354.69 to 7107.37) | 208.78 (91.37 to 479.36) | 9103.42 (3971.12 to 20978.1) | 235.44 (102.71 to 542.56) | 0.68 (0.48 to 0.89) | <0.001 |
| Mongolia | 4.23 (0.77 to 13.1) | 2.94 (0.53 to 9.1) | 11.91 (2.3 to 37.21) | 2.68 (0.52 to 8.38) | -0.97 (-1.48 to -0.46) | 0.001 | 225 (97.7 to 521.54) | 156.25 (67.85 to 362.17) | 672.19 (300.73 to 1505.16) | 151.42 (67.74 to 339.06) | -0.56 (-1 to -0.12) | 0.015 |
| Montenegro | 3.06 (0.57 to 8.8) | 3.6 (0.67 to 10.37) | 3.87 (0.77 to 12.05) | 3.66 (0.73 to 11.41) | 0.06 (-0.16 to 0.27) | 0.58 | 162.6 (73.12 to 352.92) | 191.56 (86.14 to 415.77) | 205.33 (94.36 to 462.13) | 194.41 (89.34 to 437.55) | 0.11 (-0.06 to 0.28) | 0.208 |
| New Zealand | 15.53 (3.02 to 48.01) | 3.62 (0.71 to 11.19) | 31.98 (6.37 to 97.02) | 3.97 (0.79 to 12.05) | 0.5 (0.33 to 0.66) | <0.001 | 850.79 (386.17 to 1891.5) | 198.32 (90.02 to 440.92) | 1746.27 (813.01 to 3808.46) | 216.9 (100.98 to 473.04) | 0.44 (0.31 to 0.58) | <0.001 |
| North Macedonia | 9.07 (1.69 to 27.85) | 3.39 (0.63 to 10.42) | 13.33 (2.55 to 40.01) | 3.51 (0.67 to 10.55) | 0.17 (0.03 to 0.3) | 0.017 | 479.21 (212.58 to 1090.05) | 179.26 (79.52 to 407.75) | 707.57 (319.27 to 1576.63) | 186.59 (84.2 to 415.77) | 0.22 (0.1 to 0.34) | 0.001 |
| Northern Mariana Islands | 0.07 (0.01 to 0.22) | 2.24 (0.42 to 7.04) | 0.3 (0.06 to 0.96) | 3.82 (0.73 to 12.04) | 2.19 (1.49 to 2.9) | <0.001 | 3.74 (1.6 to 8.79) | 119 (50.86 to 279.24) | 15.83 (6.86 to 37.34) | 198.82 (86.17 to 468.94) | 2.07 (1.49 to 2.66) | <0.001 |
| Palestine | 4.92 (0.92 to 15.58) | 3.68 (0.69 to 11.65) | 12.33 (2.34 to 37.93) | 2.77 (0.53 to 8.51) | -1.3 (-1.45 to -1.15) | <0.001 | 260.33 (117.67 to 596.62) | 194.8 (88.05 to 446.44) | 693.35 (313.2 to 1540.61) | 155.52 (70.25 to 345.57) | -1.04 (-1.18 to -0.89) | <0.001 |
| People's Democratic Republic of Algeria | 69.07 (12.98 to 210.14) | 3.91 (0.73 to 11.9) | 191.73 (36.73 to 599.46) | 3.49 (0.67 to 10.92) | -0.42 (-0.62 to -0.21) | <0.001 | 3572.62 (1554.65 to 8299.41) | 202.3 (88.03 to 469.96) | 9938.49 (4323.39 to 22924.1) | 181.01 (78.74 to 417.53) | -0.38 (-0.58 to -0.18) | <0.001 |
| People's Republic of Bangladesh | 177.62 (33.47 to 532.28) | 2.67 (0.5 to 8.01) | 624.61 (115.41 to 1943.96) | 3.16 (0.58 to 9.83) | 0.49 (0.38 to 0.6) | <0.001 | 8730.59 (3538.26 to 20126.81) | 131.33 (53.22 to 302.76) | 29044.12 (11386.88 to 70607.16) | 146.81 (57.56 to 356.9) | 0.33 (0.23 to 0.43) | <0.001 |
| People's Republic of China | 5096.33 (972.71 to 14964.74) | 4.37 (0.83 to 12.84) | 10957.45 (2059.27 to 33692.65) | 4.38 (0.82 to 13.48) | 0.19 (-0.03 to 0.41) | 0.094 | 257424 (114145.96 to 580481.81) | 220.92 (97.96 to 498.16) | 603380.39 (271921.39 to 1341698.93) | 241.33 (108.76 to 536.63) | 0.38 (0.18 to 0.59) | <0.001 |
| Plurinational State of Bolivia | 13.33 (2.45 to 41.73) | 2.63 (0.48 to 8.22) | 37.61 (7.1 to 116.6) | 2.8 (0.53 to 8.7) | 0.38 (0.31 to 0.46) | <0.001 | 670.09 (285.91 to 1588.56) | 132.01 (56.33 to 312.96) | 1878.89 (802.67 to 4390.93) | 140.11 (59.85 to 327.43) | 0.37 (0.3 to 0.44) | <0.001 |
| Portuguese Republic | 53.34 (10.42 to 162.04) | 3.49 (0.68 to 10.59) | 68.43 (13.59 to 206.32) | 3.36 (0.67 to 10.14) | -0.08 (-0.17 to 0) | 0.058 | 2751.6 (1233.21 to 6241.61) | 179.86 (80.61 to 407.99) | 3545.42 (1557.5 to 7952.54) | 174.3 (76.57 to 390.96) | -0.06 (-0.14 to 0.01) | 0.105 |
| Principality of Andorra | 0.21 (0.04 to 0.64) | 3.14 (0.6 to 9.33) | 0.5 (0.09 to 1.64) | 2.9 (0.53 to 9.42) | -0.34 (-0.53 to -0.14) | 0.001 | 11.11 (4.9 to 24.4) | 162.67 (71.71 to 357.24) | 26.87 (11.58 to 61.97) | 154.71 (66.7 to 356.85) | -0.23 (-0.41 to -0.06) | 0.01 |
| Principality of Monaco | 0.19 (0.03 to 0.57) | 3.48 (0.6 to 10.38) | 0.22 (0.04 to 0.65) | 3.13 (0.58 to 9.27) | -0.42 (-0.54 to -0.31) | <0.001 | 9.78 (4.29 to 21.65) | 179.21 (78.55 to 396.78) | 12.05 (5.35 to 25.9) | 170.55 (75.78 to 366.63) | -0.27 (-0.36 to -0.17) | <0.001 |
| Puerto Rico | 12.55 (2.43 to 38.34) | 2.73 (0.53 to 8.34) | 19.72 (3.86 to 60.11) | 3.41 (0.67 to 10.39) | 0.89 (0.81 to 0.97) | <0.001 | 674.39 (302.41 to 1511.63) | 146.63 (65.75 to 328.66) | 1037.84 (460.14 to 2251.9) | 179.42 (79.55 to 389.3) | 0.78 (0.72 to 0.84) | <0.001 |
| Republic of Albania | 9.56 (1.82 to 29.13) | 3.41 (0.65 to 10.39) | 19.06 (3.63 to 59.59) | 4.25 (0.81 to 13.29) | 0.63 (0.37 to 0.9) | <0.001 | 499.87 (216.32 to 1143.49) | 178.38 (77.19 to 408.05) | 1002.86 (450.73 to 2329.26) | 223.71 (100.54 to 519.59) | 0.7 (0.47 to 0.93) | <0.001 |
| Republic of Angola | 25.84 (4.91 to 77.62) | 3.77 (0.72 to 11.34) | 102.83 (18.25 to 312.06) | 4.25 (0.75 to 12.89) | 0.45 (0.24 to 0.67) | <0.001 | 1310.74 (553.86 to 3048.01) | 191.46 (80.9 to 445.23) | 4908.57 (2014.8 to 11647.19) | 202.71 (83.21 to 481) | 0.26 (0.09 to 0.44) | 0.005 |
| Republic of Armenia | 14.69 (2.89 to 44.34) | 3.73 (0.73 to 11.24) | 20.13 (3.9 to 60.86) | 4.02 (0.78 to 12.17) | 0.39 (-0.31 to 1.1) | 0.262 | 794.27 (355.31 to 1741.34) | 201.37 (90.08 to 441.49) | 1062.87 (476.2 to 2339.91) | 212.53 (95.22 to 467.89) | 0.39 (-0.22 to 1.01) | 0.202 |
| Republic of Austria | 38.7 (7.49 to 117.37) | 3.36 (0.65 to 10.19) | 51.89 (10.06 to 157.25) | 3.28 (0.64 to 9.94) | -0.08 (-0.25 to 0.08) | 0.305 | 2014.79 (899.81 to 4540.79) | 174.89 (78.1 to 394.15) | 2743.87 (1242.23 to 6173.68) | 173.47 (78.54 to 390.31) | -0.08 (-0.22 to 0.06) | 0.251 |
| Republic of Azerbaijan | 25.35 (4.88 to 76.72) | 3.51 (0.68 to 10.64) | 57.19 (10.86 to 177.68) | 3.43 (0.65 to 10.67) | -0.85 (-1.69 to 0) | 0.049 | 1379.62 (617.09 to 3012.1) | 191.31 (85.57 to 417.68) | 3098.26 (1372.71 to 7006.35) | 186.04 (82.43 to 420.71) | -0.62 (-1.37 to 0.13) | 0.1 |
| Republic of Belarus | 65.34 (12.75 to 199.65) | 4.01 (0.78 to 12.25) | 74.48 (14.44 to 231.61) | 4.17 (0.81 to 12.98) | 0.14 (-0.34 to 0.63) | 0.552 | 3442.16 (1537.86 to 7616.53) | 211.12 (94.32 to 467.14) | 3875.61 (1734.55 to 8859.31) | 217.14 (97.18 to 496.35) | 0.16 (-0.27 to 0.59) | 0.46 |
| Republic of Benin | 8.81 (1.67 to 28.01) | 2.93 (0.56 to 9.32) | 22.71 (4.25 to 72.96) | 2.42 (0.45 to 7.79) | -0.73 (-0.84 to -0.62) | <0.001 | 432.43 (181.03 to 1034.51) | 143.82 (60.21 to 344.07) | 1146.8 (465.29 to 2716.5) | 122.41 (49.67 to 289.96) | -0.59 (-0.67 to -0.51) | <0.001 |
| Republic of Botswana | 3.28 (0.58 to 10.17) | 3.61 (0.64 to 11.17) | 8.39 (1.48 to 25.94) | 3.11 (0.55 to 9.62) | -0.23 (-0.52 to 0.06) | 0.121 | 165.02 (68.58 to 390.54) | 181.34 (75.37 to 429.16) | 426.27 (173.9 to 1012.37) | 158.12 (64.51 to 375.53) | -0.21 (-0.44 to 0.03) | 0.087 |
| Republic of Bulgaria | 55.14 (10.52 to 169.72) | 3.82 (0.73 to 11.76) | 44.88 (9.04 to 138.37) | 3.69 (0.74 to 11.36) | 0.12 (-0.05 to 0.29) | 0.158 | 2908.67 (1318.38 to 6536.97) | 201.5 (91.33 to 452.85) | 2367.82 (1055.03 to 5220.24) | 194.48 (86.65 to 428.76) | 0.11 (-0.03 to 0.26) | 0.114 |
| Republic of Burundi | 10.66 (1.98 to 34.31) | 2.91 (0.54 to 9.37) | 25.03 (4.52 to 78.07) | 3.02 (0.55 to 9.42) | 0.33 (0.05 to 0.61) | 0.022 | 575.86 (257.88 to 1358.72) | 157.22 (70.41 to 370.96) | 1285.25 (544.91 to 3027.81) | 155.14 (65.78 to 365.49) | 0.19 (-0.06 to 0.43) | 0.131 |
| Republic of Cabo Verde | 0.85 (0.15 to 2.49) | 3.17 (0.57 to 9.32) | 1.9 (0.36 to 6.01) | 2.92 (0.56 to 9.23) | -0.91 (-1.62 to -0.2) | 0.014 | 44.71 (20.05 to 98.56) | 167.32 (75.02 to 368.81) | 97.18 (42.06 to 228.02) | 149.26 (64.6 to 350.23) | -0.78 (-1.39 to -0.16) | 0.015 |
| Republic of Cameroon | 22.83 (4.55 to 71.92) | 3.12 (0.62 to 9.83) | 58.34 (10.16 to 188.05) | 2.55 (0.44 to 8.23) | -0.72 (-0.97 to -0.48) | <0.001 | 1090.63 (439.66 to 2673.96) | 149.01 (60.07 to 365.35) | 2852.9 (1115.74 to 7124.35) | 124.87 (48.84 to 311.84) | -0.62 (-0.8 to -0.43) | <0.001 |
| Republic of Chad | 10.93 (2.09 to 34.1) | 2.67 (0.51 to 8.34) | 24.16 (4.33 to 75.9) | 2.41 (0.43 to 7.59) | -0.31 (-0.35 to -0.26) | <0.001 | 555.83 (238.11 to 1298.27) | 135.89 (58.21 to 317.4) | 1191.84 (475.36 to 2862.57) | 119.1 (47.5 to 286.06) | -0.42 (-0.45 to -0.38) | <0.001 |
| Republic of Chile | 39.39 (7.52 to 123.61) | 2.75 (0.53 to 8.64) | 97.15 (18.59 to 292.22) | 3.26 (0.62 to 9.82) | 0.46 (0.2 to 0.73) | 0.001 | 2202.8 (1006.29 to 4980.48) | 154 (70.35 to 348.18) | 5263.79 (2422.08 to 11600.44) | 176.89 (81.39 to 389.82) | 0.42 (0.19 to 0.64) | 0.001 |
| Republic of Colombia | 72.44 (13.66 to 222.91) | 2.64 (0.5 to 8.11) | 242.91 (46.66 to 729.24) | 3.39 (0.65 to 10.16) | 0.88 (0.64 to 1.11) | <0.001 | 3967.32 (1765.93 to 8799.69) | 144.35 (64.25 to 320.18) | 12859.23 (5610.67 to 28618.28) | 179.2 (78.19 to 398.81) | 0.77 (0.58 to 0.96) | <0.001 |
| Republic of Costa Rica | 6.97 (1.36 to 21.2) | 2.82 (0.55 to 8.59) | 23.98 (4.68 to 73.36) | 3.36 (0.66 to 10.28) | 0.71 (0.41 to 1.02) | <0.001 | 375.64 (168.75 to 852.12) | 152.15 (68.35 to 345.15) | 1268.72 (575.96 to 2891.02) | 177.81 (80.72 to 405.18) | 0.67 (0.41 to 0.93) | <0.001 |
| Republic of Croatia | 31.57 (6.1 to 96.7) | 3.96 (0.76 to 12.12) | 29.07 (5.7 to 89.06) | 3.93 (0.77 to 12.03) | -0.01 (-0.21 to 0.2) | 0.949 | 1672.4 (769.23 to 3726.05) | 209.63 (96.42 to 467.05) | 1552.13 (699.64 to 3415.05) | 209.63 (94.49 to 461.24) | 0.08 (-0.1 to 0.26) | 0.389 |
| Republic of Cuba | 31.67 (6.15 to 96.27) | 2.48 (0.48 to 7.55) | 66.66 (12.91 to 212.5) | 3.16 (0.61 to 10.08) | 0.31 (0.11 to 0.5) | 0.003 | 1680.05 (738.33 to 3776.63) | 131.7 (57.88 to 296.04) | 3481.72 (1542.21 to 8128.82) | 165.2 (73.17 to 385.69) | 0.25 (0.06 to 0.45) | 0.012 |
| Republic of Cyprus | 3.7 (0.69 to 11.55) | 3.73 (0.7 to 11.64) | 6.8 (1.32 to 20.17) | 2.93 (0.57 to 8.69) | -0.84 (-0.95 to -0.74) | <0.001 | 178.89 (73.84 to 426.09) | 180.35 (74.44 to 429.57) | 356.4 (155.43 to 801.48) | 153.59 (66.98 to 345.38) | -0.5 (-0.57 to -0.42) | <0.001 |
| Population of Côte d ivoire | 16.43 (3.1 to 52.94) | 2.35 (0.44 to 7.59) | 46.42 (7.85 to 150.75) | 2.34 (0.4 to 7.61) | 0.04 (-0.18 to 0.27) | 0.695 | 835.37 (349.51 to 2010.64) | 119.72 (50.09 to 288.15) | 2331.82 (940.65 to 5770.46) | 117.77 (47.51 to 291.44) | -0.03 (-0.19 to 0.13) | 0.74 |
| Republic of Djibouti | 0.76 (0.14 to 2.34) | 2.83 (0.53 to 8.77) | 3.79 (0.62 to 11.54) | 3.15 (0.52 to 9.57) | 0.26 (0.12 to 0.41) | 0.001 | 39.47 (16.57 to 91.4) | 147.65 (62 to 341.91) | 188.22 (75.74 to 442.75) | 156.1 (62.81 to 367.19) | 0.12 (0 to 0.24) | 0.043 |
| Republic of Ecuador | 18.26 (3.46 to 55.56) | 2.32 (0.44 to 7.04) | 54.07 (10.05 to 168.96) | 2.47 (0.46 to 7.71) | 0.36 (0.27 to 0.45) | <0.001 | 976.45 (425.23 to 2246.7) | 123.81 (53.92 to 284.87) | 2890.02 (1264.1 to 6558.37) | 131.86 (57.68 to 299.24) | 0.34 (0.26 to 0.41) | <0.001 |
| Republic of El Salvador | 12.94 (2.44 to 39.66) | 3.02 (0.57 to 9.26) | 25.71 (4.82 to 80.03) | 2.99 (0.56 to 9.29) | -0.06 (-0.14 to 0.01) | 0.079 | 677.97 (301.01 to 1551.4) | 158.36 (70.31 to 362.38) | 1377.05 (595.93 to 3129.77) | 159.89 (69.2 to 363.4) | 0.02 (-0.03 to 0.07) | 0.482 |
| Republic of Equatorial Guinea | 1.4 (0.26 to 4.34) | 4.19 (0.77 to 12.97) | 4.04 (0.6 to 12.35) | 3.76 (0.56 to 11.52) | -0.32 (-0.37 to -0.26) | <0.001 | 69.03 (28.39 to 163.41) | 206.34 (84.88 to 488.49) | 196.72 (79.2 to 472.08) | 183.4 (73.84 to 440.12) | -0.35 (-0.39 to -0.31) | <0.001 |
| Republic of Estonia | 9.47 (1.84 to 29.5) | 3.7 (0.72 to 11.52) | 7.76 (1.56 to 22.62) | 3.51 (0.71 to 10.24) | -0.07 (-0.21 to 0.08) | 0.346 | 500.52 (228.06 to 1141.54) | 195.45 (89.06 to 445.76) | 424.54 (189.43 to 910.18) | 192.2 (85.76 to 412.06) | 0.06 (-0.05 to 0.18) | 0.248 |
| Republic of Fiji | 2.09 (0.38 to 6.39) | 3.2 (0.59 to 9.79) | 4.1 (0.74 to 12.64) | 3.49 (0.63 to 10.76) | 0.32 (0.15 to 0.5) | 0.001 | 105.74 (44.63 to 245.54) | 161.91 (68.34 to 375.98) | 213.49 (92.42 to 486.66) | 181.78 (78.7 to 414.38) | 0.44 (0.3 to 0.57) | <0.001 |
| Republic of Finland | 25.07 (4.92 to 77.09) | 3.21 (0.63 to 9.87) | 30.4 (5.98 to 91.92) | 3.54 (0.7 to 10.69) | 0.76 (0.56 to 0.96) | <0.001 | 1270.4 (551.84 to 2944.21) | 162.65 (70.65 to 376.94) | 1536.19 (681.59 to 3534.85) | 178.71 (79.29 to 411.23) | 0.68 (0.52 to 0.84) | <0.001 |
| Republic of Ghana | 25.68 (4.8 to 81.85) | 2.47 (0.46 to 7.87) | 82.83 (15.11 to 251.03) | 2.63 (0.48 to 7.98) | 0.09 (-0.17 to 0.35) | 0.487 | 1288.09 (518.73 to 3061.37) | 123.8 (49.86 to 294.24) | 4004.8 (1611.98 to 9479.56) | 127.38 (51.27 to 301.53) | 0.03 (-0.15 to 0.22) | 0.733 |
| Republic of Guatemala | 15.82 (2.93 to 48.47) | 2.8 (0.52 to 8.58) | 47.26 (9.06 to 145.8) | 2.84 (0.54 to 8.75) | 0.26 (0.15 to 0.38) | <0.001 | 840.93 (370.75 to 1909.77) | 148.93 (65.66 to 338.23) | 2513.9 (1110.21 to 5673.64) | 150.87 (66.63 to 340.51) | 0.25 (0.16 to 0.34) | <0.001 |
| Republic of Guinea | 13.08 (2.44 to 40.27) | 2.81 (0.53 to 8.66) | 23.82 (4.22 to 72.33) | 2.54 (0.45 to 7.71) | -0.43 (-0.47 to -0.39) | <0.001 | 655.57 (275.68 to 1548.81) | 141.01 (59.3 to 333.15) | 1175.45 (484.1 to 2809.82) | 125.37 (51.63 to 299.68) | -0.44 (-0.47 to -0.4) | <0.001 |
| Republic of Guinea-Bissau | 1.87 (0.37 to 6.01) | 2.78 (0.55 to 8.93) | 3.94 (0.72 to 12.5) | 2.67 (0.48 to 8.46) | -0.09 (-0.13 to -0.05) | <0.001 | 90.22 (35.35 to 224.42) | 134.08 (52.53 to 333.54) | 186.17 (73.06 to 461.69) | 126.03 (49.46 to 312.54) | -0.15 (-0.19 to -0.12) | <0.001 |
| Republic of Guyana | 1.25 (0.25 to 3.88) | 2.13 (0.43 to 6.61) | 2.66 (0.5 to 8.58) | 2.71 (0.52 to 8.76) | 1.01 (0.76 to 1.25) | <0.001 | 68.58 (30.4 to 156.92) | 116.93 (51.84 to 267.55) | 139.54 (59.16 to 325.62) | 142.49 (60.41 to 332.51) | 0.85 (0.63 to 1.06) | <0.001 |
| Republic of Haiti | 14.1 (2.59 to 42.52) | 2.82 (0.52 to 8.51) | 32.78 (5.87 to 105.71) | 2.47 (0.44 to 7.98) | -0.3 (-0.4 to -0.19) | <0.001 | 717.64 (302.77 to 1643.5) | 143.6 (60.58 to 328.85) | 1669.54 (698.77 to 4055.44) | 126.02 (52.74 to 306.1) | -0.31 (-0.4 to -0.22) | <0.001 |
| Republic of Honduras | 10.5 (2.04 to 32.16) | 3.35 (0.65 to 10.27) | 39.02 (7.25 to 121.83) | 3.8 (0.71 to 11.86) | 0.52 (0.47 to 0.57) | <0.001 | 526.21 (220.47 to 1219.16) | 168.03 (70.4 to 389.3) | 1843.48 (741.02 to 4586) | 179.51 (72.16 to 446.56) | 0.35 (0.3 to 0.39) | <0.001 |
| Republic of Iceland | 0.94 (0.18 to 2.87) | 3.17 (0.62 to 9.72) | 1.53 (0.3 to 4.52) | 2.86 (0.56 to 8.46) | -0.05 (-0.36 to 0.25) | 0.723 | 49.4 (22.19 to 112) | 167.04 (75.02 to 378.72) | 84.94 (40.17 to 184.6) | 159.07 (75.23 to 345.72) | 0.1 (-0.15 to 0.36) | 0.403 |
| Republic of India | 1954.51 (367.93 to 6214.27) | 2.7 (0.51 to 8.59) | 5686.55 (1076.48 to 17512.21) | 3.37 (0.64 to 10.37) | 0.57 (0.45 to 0.69) | <0.001 | 96687.78 (41726.88 to 234335.88) | 133.62 (57.66 to 323.84) | 263725.12 (109981.02 to 646831.08) | 156.17 (65.13 to 383.04) | 0.37 (0.28 to 0.47) | <0.001 |
| Republic of Indonesia | 547.88 (104.57 to 1764.28) | 3.33 (0.63 to 10.71) | 1472.05 (283.31 to 4710.37) | 3.67 (0.71 to 11.75) | 0.01 (-0.13 to 0.15) | 0.94 | 30624.83 (14158.21 to 70534.2) | 185.97 (85.97 to 428.31) | 79287.26 (36433.86 to 183533.98) | 197.74 (90.87 to 457.73) | -0.02 (-0.16 to 0.12) | 0.776 |
[truncated: 24,253 more chars]
